# scGraph2Vec: a deep generative model for gene embedding augmented by graph neural network and single-cell omics data

**DOI:** 10.1093/gigascience/giae108

**Published:** 2024-12-20

**Authors:** Shiqi Lin, Peilin Jia

**Affiliations:** National Genomics Data Center, China National Center for Bioinformation, Beijing 100101, China; Beijing Institute of Genomics, Chinese Academy of Sciences, Beijing 100101, China; College of Life Sciences, University of Chinese Academy of Sciences, Beijing 100049, China; National Genomics Data Center, China National Center for Bioinformation, Beijing 100101, China; Beijing Institute of Genomics, Chinese Academy of Sciences, Beijing 100101, China

**Keywords:** gene embedding, gene regulatory network, single-cell RNA-seq, tissue specificity, complex disease

## Abstract

**Background:**

Exploring the cellular processes of genes from the aspects of biological networks is of great interest to understanding the properties of complex diseases and biological systems. Biological networks, such as protein–protein interaction networks and gene regulatory networks, provide insights into the molecular basis of cellular processes and often form functional clusters in different tissue and disease contexts.

**Results:**

We present scGraph2Vec, a deep learning framework for generating informative gene embeddings. scGraph2Vec extends the variational graph autoencoder framework and integrates single-cell datasets and gene–gene interaction networks. We demonstrate that the gene embeddings are biologically interpretable and enable the identification of gene clusters representing functional or tissue-specific cellular processes. By comparing similar tools, we showed that scGraph2Vec clearly distinguished different gene clusters and aggregated more biologically functional genes. scGraph2Vec can be widely applied in diverse biological contexts. We illustrated that the embeddings generated by scGraph2Vec can infer disease-associated genes from genome-wide association study data (e.g., COVID-19 and Alzheimer's disease), identify additional driver genes in lung adenocarcinoma, and reveal regulatory genes responsible for maintaining or transitioning melanoma cell states.

**Conclusions:**

scGraph2Vec not only reconstructs tissue-specific gene networks but also obtains a latent representation of genes implying their biological functions.

## Background

Our understanding of the molecular property and regulatory mechanism is highly incomplete, especially at the cell type and tissue resolution. The common and tissue-specific processes are often controlled by different gene regulatory programs, which alter the expression of genes in different biological conditions [1, 2]. Comparison of gene regulatory networks in different tissues shows that the edges of the network (e.g., the links between transcription factors to target genes) have higher tissue specificity than the nodes of the network (e.g., genes) because the links among genes are heavily regulated by their functional roles and the tissue contexts [1]. In the disease environment, the topology of molecular networks helps identify novel genes and pathways associated with diseases [3].

In recent years, huge amounts of omics data from various human tissues and organs have been accumulated [4–7]. Many methods have been developed to decode the dynamic regulatory links among genes [8]. Network embeddings hold substantial promise for analyzing gene regulatory programs under various conditions. For example, the Set2Gaussian [9] method identified the gene set embeddings based on the topology of the protein–protein interaction (PPI) network and used the resultant embeddings for tumor stratification and clinical prognosis. Methods such as SAUCIE [10], scVI [11], LDVAE [12], scGNN [13], and scVAE [14] used deep neural networks to aggregate and represent cell type–specific gene regulatory signals from single-cell transcriptome data, enabling highly accurate downstream analysis. Additionally, scETM [15], siVAE [16], and SIMBA [17] confirmed the broad potential of learning cell and gene embeddings simultaneously for studying cell heterogeneity, identifying gene expression features, and integrating omics data. Furthermore, scapGNN [18] inferred stable gene–cell association networks from sparse single-cell profile data.

Deep learning approaches have proved advantageous in many human genetics and genomics studies [8]. Variational graph autoencoder (VGAE) [19] is a type of graph neural network (GNN) designed to efficiently learn latent representations of graph-structured data, enabling tasks like link prediction and node clustering by leveraging both graph topology and node features [20, 21]. Given the strong ability of VGAE to generate graph embeddings, several methods have been developed based on this framework, including ARGA [22], ARGVA [22], SIG-VAE [23], and DGVAE [24], among others. ARGA and ARGVA [22] incorporate an adversarial regularized embedding framework to improve the efficiency in handling real-world graph data. SIG-VAE [23] enhances VGAE’s flexibility using a hierarchical variational framework, enabling it to capture graph dependency structure and produce more interpretable latent representations. DGVAE [24] introduces graph cluster memberships as latent factors and a new GNN variant, leading to better performance in graph generation and clustering tasks. These methods demonstrated the adaptability and interpretability of VGAE for diverse real-world graph modeling challenges.

In this work, we developed an extended VGAE approach [19, 25]—namely, scGraph2Vec—that integrates single-cell datasets with gene–gene interaction networks to generate highly informative gene embeddings. We showed that the resultant gene embeddings recapitulate high-dimensional biological information derived from the structures of gene–gene interaction networks and gene expression patterns across cells. Compared to 13 existing embedding tools, scGraph2Vec demonstrated promising performance in gene embedding and cluster prediction on benchmark single-cell RNA sequencing (scRNA-seq) datasets. These gene embeddings help us to understand the functional clusters of genes, elucidate the influence of regulatory genes on biological processes in specific tissue environments, and infer more disease-related genes to explain disease risk. In summary, scGraph2Vec can be used on a wide range of gene networks and single-cell datasets for different biological problems.

## Methods

### Data collection and preprocessing

We collected the scRNA-seq data from 6 healthy human tissues, which are brain [26], heart [27], kidney [28], liver [29], lung [30], and peripheral blood mononuclear cells (PBMCs) [31] (Supplementary Table S1). We obtained paired single-cell ATAC sequencing (scATAC-seq) and scRNA-seq data from 4 mid-gestation human cerebral cortex samples (GEO accession ID: GSE162170) [32]. The lung adenocarcinoma (LUAD) tumor tissues and their distal nonmalignant lung tissues were downloaded from EBI ArrayExpress (accession ID: E-MTAB-6149 and E-MTAB-6653) [33]. The scRNA-seq and bulk RNA-seq datasets from human melanoma cell lines with and without perturbations (*SOX10* knockdown) were downloaded from Scope (Scope session: Wouters_Human_Melanoma) and GEO (accession ID: GSE134432) [34]. For each dataset, we filtered for genes with a nonzero expression value in at least 3 cells and for cells with at least 200 expressed genes. The originally downloaded count data were transformed to log (counts per million + 1) values and scaled by all cells.

We used 3 representative interaction networks: the physical interaction network, the biological pathways, and the transcriptional regulatory network. The physical interaction network was downloaded from the BioGRID database (release v.4.4.210) [35], which contained 977,356 interactions among 19,752 genes. We also extracted the Reactome [36] dataset from PathwayCommons (v14, [37]) [38] to construct interaction networks that characterize biological pathways such as signaling or metabolism, which contained 326,439 interactions among 10,800 genes. The transcriptional regulatory network from the TRRUST database (v2, [39]) [40] contained 8,427 interactions among 2,862 genes. To explore the impact of different network features on embedding, we divided the nodes of each network into 20 groups evenly based on degree and took the top 100 genes of each group as hub genes. The hub gene and its neighbor genes were formed into a gene cluster, and then we measured the aggregation of these gene clusters in the 2-dimensional embeddings using the Hopkins statistic.

### scGraph2Vec model design

scGraph2Vec takes 2 input matrices: the adjacency matrix *A* from the gene–gene interaction network and the node feature matrix *X* from the single-cell gene profile. We first refine *A* by including a node community matrix and generate an enhanced adjacency matrix *A_n_*. scGraph2Vec (RRID: SCR_025322) directs *A_n_* and *X* to go through a multilayer graph convolutional network (GCN) to generate the low-dimensional vectors *Z* and reconstructs the graph structure of the network through the decoder process.

Specifically, we first calculate the primary assignments of node communities by using the Louvain greedy algorithm [41] based on the interaction network. Let $M \in {\{ {0,1} \}}^{n \times k}$ represent the membership matrix, where *n* is the total number of nodes and *k* is the total number of communities. Each element in *M* indicates whether a node *i* (*i* = 1, …, *n*) belongs to a community ${C}_j$ (*j* = 1, …, *k*), that is, ${M}_{ij} = 1$ if $i \in {C}_j$ and 0 otherwise. Subsequently, the node community matrix ${A}_c$ is calculated as follows:


(1) \begin{eqnarray*}
{A}_c = M{M}^T - {I}_n
\end{eqnarray*}

where ${I}_n$ is the identity matrix. Then, we calculate a new adjacency matrix ${A}_n$ by


(2) \begin{eqnarray*}
{A}_n = A + \lambda {A}_c
\end{eqnarray*}

where the hyperparameter λ > 0 is introduced to balance the contribution of the original adjacency matrix *A* and the derived community matrix *A_c_*. To alleviate the computational cost, we require that in each community ${C}_j$, each node $i \in {C}_j$ only connects to a predefined number (denoted by *s*) of nodes in ${C}_j$, instead of connecting to all nodes in ${C}_j$.Thus, *s* is a hyperparameter that can tune the sparsity of ${A}_n$.

The encoder includes multiple convolutional layers. We design 2 alternative structures: one with 3 layers (256-64-16) and the other with 2 layers (64-16). The encoder generates the embedding matrix *Z*:


(3) \begin{eqnarray*}
Z = GCN\left( {{A}_n,X} \right)
\end{eqnarray*}

The decoder reconstructs the connectivity structure of the network using *Z*, which relies on the inner product decoder followed by the nonlinear process using the sigmoid activation function:


(4) \begin{eqnarray*}
{\hat{A}} = \sigma \left( {Z{Z}^T} \right)
\end{eqnarray*}

where ${\mathrm{\sigma }}( x ) = \frac{1}{{1 + {e}^{ - x}}}$.

The optimization strategy for reconstruction typically uses the evidence lower bound (ELBO) loss ${L}_{\textit{VGAE}}\ $ to assess the similarity between the initial and the reconstructed graph structure:


(5) \begin{eqnarray*}
{L}_{{VGAE}} = {E}_{q\left( {Z\left| {A,X} \right.} \right)}\left[ {\log p(A\left| {Z,X)} \right.} \right] - KL\left( {q\left( {Z{\mathrm{|}}A,X} \right)\|p\left( Z \right)} \right)
\end{eqnarray*}

where $KL( {q( {Z{\mathrm{|}}A,X} )\|p( Z )} )$ is the Kullback–Leibler divergence between $q( \cdot )$ and $p( \cdot )$. More description of ${L}_{\textit{VGAE}}$ can found in the original VGAE article [19]. In scGraph2Vec, we include a complemental loss inspired by modularity for community detection. Modularity is designed to measure the strength of community structure in networks by comparing the density of connections inside and outside communities [42, 43]. To capture the global community structure, we soften the calculation of specific communities in the traditional modular formula and add a global regularizer [25]:


(6) \begin{eqnarray*}
{L}_M = \frac{\beta }{{2m}}\mathop \sum \limits_{i,j = 1}^n \left( {{A}_{ij} - \frac{{{d}_i{d}_j}}{{2m}}} \right){e}^{ - \gamma \|{z}_i - {z}_j\|_2^2}
\end{eqnarray*}

Here, the L2 distance $\|{z}_i - {z}_j\|_2^2$ is the soft counterpart of modularity, which replaces the original community indicator ${\mathrm{\delta }}( {{\mathrm{i}},{\mathrm{\ j}}} ) \in \{ {0,1} \}$, where ${\mathrm{\delta }}( {{\mathrm{i}},{\mathrm{\ j}}} ) = 1$ if nodes *i* and *j* belong to the same community and 0 otherwise. The ${d}_i = \mathop \sum \limits_{j = 1}^n {A}_{i,j}\ $ (*n* is the total number of nodes) is the degree for the *i*th node, and 2*m* is the sum of the degrees of all nodes. Therefore, the loss function does not involve the “exact” modularity and is independent of the community information associated with nodes. The hyperparameter $\beta > 0$ balances the relative importance of the global community structure and the pairwise node connectivity. Overall, the loss function used in scGraph2Vec is $L = \ {L}_{\textit{VGAE}} + {L}_M$. Our optimization goal is to maximize the graph similarity and modularity score to obtain gene embeddings.

### Training details

scGraph2Vec was implemented using TensorFlow (v.1.15.0). The genes in the feature matrix were matched with those in the interaction network, where genes not annotated in the interaction network were removed and genes with missing feature values were filled with 0. The positive edges (i.e., the element values of the matrix being 1) of the initial adjacency matrix were split by 90%, 5%, and 5%, and the same number of negative edges (i.e., the element values of the matrix being 0) was randomly selected to form the training set, validation set, and test set, respectively. For hyperparameter selection in scGraph2Vec, we considered standard VGAE parameters, including learning rate, training iterations, encoding layers and their dimensions, plus new parameters that were specifically designed for scGraph2Vec (i.e., λ, s, β, and γ). The optimal hyperparameters were determined by combining evaluation metrics, prioritizing a higher silhouette coefficient, a greater number of clusters, and a shorter running time. We first tested λ (0.001, 0.1, 1), β (0.5, 5, 10), γ (0.1, 1), and s (2, 10). The optimal settings were $\lambda = 1,\beta = 10,\ \gamma = 0.1,s = 10$. Subsequently, we tested a 2-layer encoder with 5 possible dimensions (i.e., 64-16, 128-16, 256-16, 128-32, and 256-32), as well as a 3-layer encoder with 2 possible dimensions (256-64-16 and 512-64-16). We tested 500, 600, and 1,000 epochs, using the Adam optimizer and a learning rate of 1 × 10^−4^. The model training was repeated 10 times in each tissue.

### Gene cluster identification

The 16-dimensional gene embeddings output by scGraph2Vec were reduced to a 2-dimensional representation for visualization by using *t*-distributed stochastic neighbor embedding (t-SNE) [44]. Then the hierarchical clustering method was used to identify gene clusters. The total number of clusters was determined according to the silhouette coefficient. We restricted each gene cluster to contain more than 10 genes. In each replication, the output with the maximum silhouette coefficient was selected.

### Benchmark with known embedding methods

We adopt a unified benchmark framework to compare scGraph2Vec with other embedding calculation methods, including ARGA [22], ARGVA [22], SIG-VAE [23], DGVAE [24], scVI [11], LDVAE [12], siVAE [16], scVAE [14], scGNN [13], scapGNN [18], scETM [15], SAUCIE [10], and SIMBA [17]. Notably, to the best of our knowledge, there is currently no method specifically designed for integrating gene interaction networks with single-cell omics data to generate gene embeddings. Therefore, we selected representative VGAE-based methods capable of generating gene embeddings based on single-cell data, excluding those unable to generate embeddings for all genes within a reasonable time frame [45] or lacking available source code [46]. We used each method to extract the embeddings of all genes in the brain and PBMC datasets, then reduced these embeddings to 2 dimensions for visualization using t-SNE. Hierarchical clustering was utilized to identify gene clusters. Considering the sparsity of single-cell data, all models assumed that data followed a zero-inflated negative binomial distribution. For the models within the unfixed VAE framework (including ARGA, ARGVA, SIG-VAE, DGVAE, scVAE, siVAE, scVI, and LDVAE), we configured their structures to match that of scGraph2Vec, including the same encoder–decoder layer numbers and dimensions (256-64-16). Optimization for all models involved scanning hyperparameters without recommended values and adjusting the learning rate (1 × 10^−3^, 1 × 10^−4^, 1 × 10^−5^) and the number of epochs (50, 100, 200, 400, 600), while other parameters were set to their defaults. Specifically, for SAUCIE, we tested parameter combinations that influenced clustering results, lambda_c (0.1, 0.2, 0.3) and lambda_d (0.5, 0.7, 0.9), and then directly used the gene embeddings and clusters provided by SAUCIE. We modified the “ConNetGNN” function of scapGNN to output gene embeddings learned from the hidden layer. For scETM, we followed the recommendation of training at least 6,000 epochs and testing models at 6,000, 9,000, and 12,000 epochs. scGNN extracted embeddings from all genes and tested both models obtained with or without the left truncated mixture Gaussian (LTMG). The final selection of the optimal model parameters was based on the convergence of the loss function.

The effectiveness of different methods in distinguishing gene clusters was evaluated using the silhouette coefficient and Davies–Bouldin index (DBI). A higher silhouette coefficient or lower DBI indicates better separation of distinct clusters. Furthermore, to estimate the biological meaning of gene clusters, we collected 50 hallmark gene sets from the MSigDB database [47, 48] as the ground truth. These gene sets represent well-defined biological states or processes. The similarity of 2 gene sets is measured by the Jaccard index.

### Benchmark with SCENIC and LIGER

We implemented the Python workflow pySCENIC (v.0.12.1) [49] to analyze gene clusters constructed by transcription factors and their potential target genes. The workflow first builds a gene coexpression network from scRNA-seq data, then uses transcription factor binding motifs to identify clusters with regulators and remove unsupported genes. We used motif data from the cisTarget Human database v9 and ran pySCENIC with default settings. Additionally, we implemented the LIGER software (v.2.0.1) [50]. LIGER utilized integrative nonnegative matrix factorization (iNMF) to identify factors that are either shared between datasets or specific to each. These factors were then used to cluster cells jointly and identify marker genes for all clusters. We applied both methods to the brain and PBMC datasets.

### Estimating biological implications of gene clusters

We explored the biological implications of clusters based on topology, annotated gene sets, and expression patterns. First, we examined whether gene clusters could reflect closely connected genes in gene–gene interaction networks. Specifically, for each cluster, we divided all connections in the BioGRID network into intracluster and out-of-cluster connections. For each gene, we calculated 2 closeness centrality values: the internal closeness centrality based on the intracluster connections and the external closeness centrality based on the out-of-cluster connections. Then, we averaged these centrality values across all genes in each cluster to determine the overall internal and external closeness centrality. Wilcoxon rank-sum test was employed to compare the internal and external closeness centrality for each cluster.

For annotated gene sets, we downloaded 50 hallmark gene sets [51] and curated a set of 397 housekeeping genes [52] (set name: HSIAO_HOUSEKEEPING_GENES) from the MSigDB database. Fisher’s exact test was employed to identify clusters enriched for the annotated gene sets.

For gene expression patterns, we calculated an expression score for each gene cluster using the “AddModuleScore” function from the Seurat software (v.4.3.0.1) [53].

### Identification of cell-type specificity and tissue specificity

For cell-type specificity, we used the gene set enrichment analysis (GSEA) [47] to examine whether cluster genes were significantly enriched in differentially expressed genes (DEGs) across cell types. We identified DEGs for each cell type using the “FindAllMarkers” function of the Seurat software [54] and ranked all genes by their average log2 (fold change). The predefined gene sets were derived from gene clusters identified by scGraph2Vec. We used the R package GSEABase (v.1.56.0) for GSEA and enrichplot (v.1.14.2) for visualization.

We then applied Fisher’s exact test to identify tissue-specific clusters. Specifically, clusters that significantly overlapped with other clusters from all other tissues were identified first (Bonferroni corrected *P* < 0.05). Clusters with significant overlap in less than 5% of the total clusters were considered tissue specific.

### Identification of disease-candidate genes

**Figure 1:**
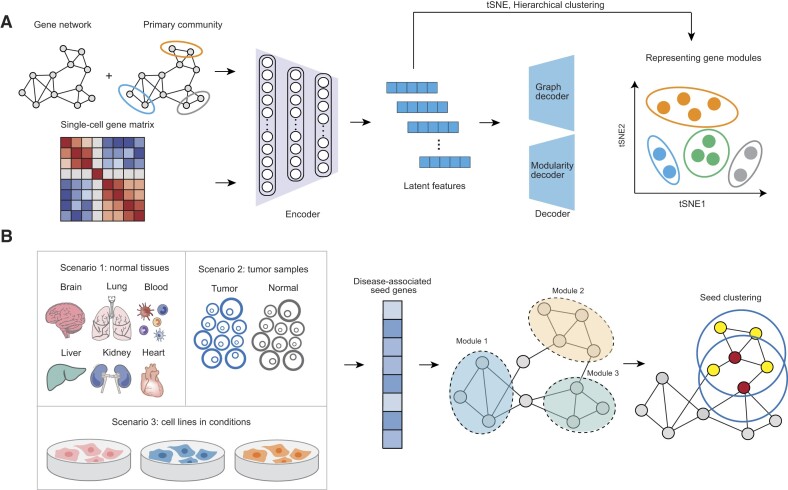
Schematic description of scGraph2Vec. (A) Feature extraction. The input of scGraph2Vec is an adjacency matrix from the gene interaction network and a feature matrix from single-cell data. The membership matrix derived from the primary community is calculated by the adjacency matrix using the Louvain algorithm and is added to the adjacency matrix. The model adopts the modified VGAE framework. The encoder is composed of a 3-layer GCN network, and the decoder considers both the reconstruction of the original dimension and the detection of the node community. The latent features were extracted as model output and reduced to 2 dimensions by t-SNE. Gene clusters were obtained using a hierarchical clustering algorithm. (B) The framework to infer novel candidate genes. The input scRNA-seq data can be derived from normal tissues, case-control tumor samples, or cell lines in different states. Starting from seed genes, defined as GWAS-significant genes or known driver genes, we can identify novel candidate genes by searching for neighboring genes in the embedding space from related tissues.

We developed a framework to identify novel candidate genes using the gene embeddings (Fig. 1B). The framework can be applied in various conditions. Here we demonstrated it to identify disease-candidate genes from genome-wide association study (GWAS) data, cancer-driver genes from tumor tissue scRNA-seq data, and regulatory genes from melanoma scRNA-seq data. In each case, scGraph2Vec was first applied to the corresponding single-cell omics data to generate gene embeddings. Next, a set of ground-truth genes was used as seed genes. Each seed gene can form a cluster if it contains more than 6 genes within a default radius of 0.5 in the embedding space. The cluster is expanded if any of its component genes has neighbor genes located within a radius of 0.5 until no further genes can be added. We excluded genes that are not direct interactors of the seed genes. Genes in the cluster are thus considered candidates for further analyses.

We demonstrated the framework to infer disease-candidate genes from GWAS summary statistics using COVID-19 and Alzheimer's disease (AD) as examples. For COVID-19, we downloaded GWAS data from Host Genetics Initiative (HGI, release 7, access date: 8 April 2022, file name: A2_ALL_eur_leave23andme) [55], including 13,769 severe COVID-19 patients and 1,072,442 healthy individuals. For AD, we downloaded GWAS data from a study conducted using 71,880 clinically diagnosed AD or AD-by-proxy cases and 383,378 controls [56]. In both cases, we used the genome-wide significant genes identified by MAGMA [57] (Bonferroni correction) as seed genes. Gene embeddings were generated using the scRNA-seq data in the disease-relevant tissues (e.g., lung for COVID-19 and brain for AD). We collected bulk RNA-seq data from those with disease and healthy individuals for validation [58, 59].

The framework can also be used to identify candidate driver genes (Fig. 1B). To this end, we downloaded scRNA-seq data from a non–small cell lung cancer (NSCLC) patient [33], including data from the tumor core sample and the distal normal tissue. The gene embeddings were generated using scGraph2Vec for tumor or normal, respectively. A total of 18 LUAD-driver genes were collected from previous studies [60] and used as seed genes. To validate the candidate genes identified from our framework, we utilized the bulk RNA-seq data from The Cancer Genome Atlas (TCGA), including 541 LUAD cases and 59 normal controls [61]. Transcript per million (TPM) normalized gene expression data were utilized to identify DEGs using the R package DESeq2 (version 1.34.0) [62] (|log2(FC)| > 0.5 and Benjamini–Hochberg [BH] adjusted *P*, or *P*_BH_ < 0.05). The univariate Cox proportional hazards regression analysis was applied to identify prognosis-related genes (*P* < 0.05). The Kaplan–Meier curves and the log-rank tests were implemented using the R package survival (v.3.5.1).

Furthermore, we applied the framework to identify regulatory genes using the melanoma scRNA-seq dataset. This dataset includes 9 patient-derived cultures and the A375 cell line, categorized into the stable melanocytic (MM001, MM011, MM031, A375), intermediate (MM057, MM074, MM087), and mesenchymal-like (MM029, MM047, MM099) states [34]. We applied scGraph2Vec to generate gene embeddings and gene clusters for each state. For the resultant clusters, we investigated their state specificity following the same approach as for tissue-specific clusters and calculated AUCell scores using the AUCell R package (v.1.25.2) [63]. Furthermore, we collected gene signatures representing 14 crucial functional states of cancer cells, including stemness, invasion, metastasis, proliferation, epithelial–mesenchymal transition (EMT), angiogenesis, apoptosis, cell cycle, differentiation, DNA damage, DNA repair, hypoxia, inflammation, and quiescence [64]. Then we applied Fisher’s exact test to identify gene clusters significantly enriched in these 14 functional states. We identified candidate genes located near *SOX10* in the gene embeddings of both the melanoma and intermediate cell states, with a clustering radius of 0.8 and a minimum of 6 genes per cluster. To validate the *SOX10* neighboring genes, we downloaded the scRNA-seq data from the same study for *SOX10* knockdown (KD) experiments for 3 intermediate samples (MM057, MM074, and MM087), each generated at multiple time points (24, 48, and 72 hours post-*SOX10* knockdown, plus a control with *SOX10*). Additionally, bulk RNA-seq data were collected from melanoma and intermediate cell lines following *SOX10* negative control and 72 hours after knockdown. We compared AUCell scores [63] of *SOX10* neighboring genes before and after *SOX10* knockdown using scRNA-seq data (*t*-test, *P* < 0.05) and performed differential expression analysis on bulk RNA-seq data from normal and *SOX10*-KD samples using DESeq2 (|log2(FC)| > 1 and *P* < 0.05) [62].

We performed pathway enrichment analysis using the R packages clusterProfiler (v.4.6.2) [65] and ReactomePA (v.1.42.0) [66], with KEGG [67], WikiPathways [68], and Reactome [69] as reference databases. ClueGO (v.2.5.10) [70] was used for pathway annotation and STRING [71] to analyze protein–protein interactions.

## Results

### Overview of scGraph2Vec

scGraph2Vec was built on a VGAE framework with extensions for the task of generating informative embeddings. It took a gene–gene interaction network and a gene–feature matrix as the input and generated gene embeddings as the output (Fig. 1A). scGraph2Vec had 3 major improvements to the standard VGAE framework. First, instead of using the standard adjacency matrix *A*, we generated an enhanced adjacency matrix *A_n_* by combining *a priori* primary community information with *A*. We used the Louvain algorithm [41] to construct a gene membership matrix, which provided the prior community information. The enhanced adjacency matrix *A_n_* thus informed the encoder with information on primary assignments for gene clusters. Second, link prediction and community detection are simultaneously implemented during the model optimization process to enhance the representation of gene communities in the embedding space. Lastly, a modularity-inspired method was implemented to optimize the loss function to reduce the impact of local pairwise connections on community structure [42, 43]. By iteratively maximizing the joint of graph likelihood and modularity scores, scGraph2Vec generated latent features representing various information of genes.

The working network was downloaded from BioGRID [35], including 19,752 genes and 977,356 interactions. Notably, the interactions were either physical (98.3%) or genetic (1.7%). Thus, it has no annotation about tissue specificity or cell-type specificity and remains the same for all applications. The gene feature matrix was constructed using the scRNA-seq [72, 73] or the scATAC-seq [74, 75] data in the gene-by-cell format. We collected scRNA-seq data for 6 human tissues, which are brain [26], heart [27], kidney [28], liver [29], lung [30], and PBMCs [31]. All scRNA-seq data were quality-controlled and processed following the same pipeline. On average, each tissue contained 66,395 cells (ranging from 2,638 cells in PBMCs to 287,269 in the heart) and 24,064 genes (ranging from 13,714 genes in PBMCs to 33,694 in the heart and kidney) (Supplementary Table S1). To ensure the same dimensionality of the inputs, the scRNA-seq gene expression matrix is trimmed or imputed to match the 19,752 genes available in the working network.

We carried out a hyperparameter sweep to examine the key hyperparameters of scGraph2Vec. The best hyperparameters were determined by using a combination of assessment parameters, including the silhouette coefficient, the number of clusters, and the running time. We first selected the hyperparameters β = 10, λ = 1, γ = 0.1, and *s* = 10 (pink line, Supplementary Fig. S1A). We next determined the following hyperparameters for model training: learning rate = 1 × 10^−4^, a 3-layer GCN encoder with 256, 64, and 16 neurons, respectively, and epoch time = 600 (blue line, Supplementary Fig. S1B). For larger datasets, we recommend a fast model with a 2-layer GCN encoder (64 and 16 neurons for each layer). The resulting latent feature is a 19,752 genes × 16 vectors matrix.

### scGraph2Vec generates gene embeddings for gene cluster identification

We reduced the latent features to 2 dimensions for visualization using t-SNE [44] and identified gene clusters by using the hierarchical clustering method. Due to the nature of neural network algorithms, such as random initialization, the same algorithm might generate slightly different results, although each output was a faithful approximation of the input graph. Hence, we replicated the model training process 10 times for each dataset, establishing a pool for selecting the best gene clusters (Supplementary Fig. S2A–C).

Next, we measured the stability of the resultant models and gene clusters obtained from the 10 replications. We found that the predicted edges were highly consistent across 10 replications (69% of the BioGRID edges were identified in all 10 replicates). To assess the stability of gene clustering, we took the fifth replication as the reference because it had the highest silhouette coefficient and then compared its resultant clusters with those from the other replications. We found that the majority of clusters could be replicated except cluster 8 and cluster 10, which were slightly mixed with other clusters (Supplementary Fig. S2C, D).

### Key components of scGraph2Vec are important for gene cluster identification

We further investigated the importance of key components in scGraph2Vec by varying its structure (e.g., excluding the gene feature matrix, randomizing the reference network, and excluding or randomizing the primary community). In each case, we generated gene embeddings using the corresponding scGraph2Vec settings (the original design or those using alternative structures) and conducted hierarchical clustering based on their 2-dimensional latent features to construct gene clusters. Then, we compared the resultant gene clusters to evaluate the models.

**Figure 2:**
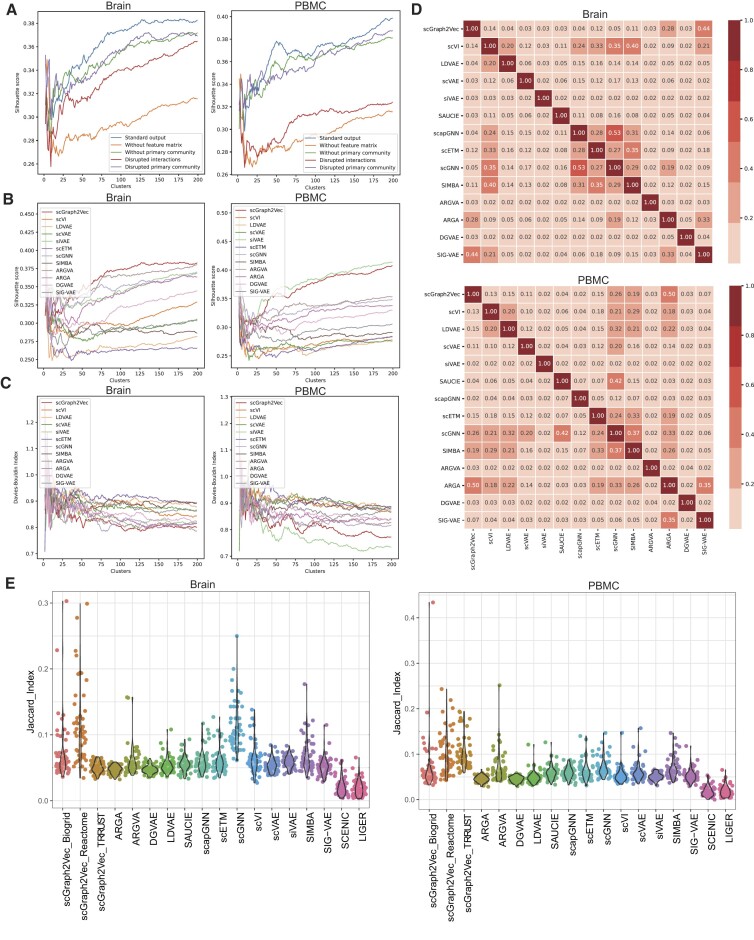
Performance comparison between different methods. (A) Comparison of silhouette coefficients among standard outputs of scGraph2Vec and other variants using brain and PBMC datasets. The x-axis is the number of clusters. The y-axis is the silhouette coefficients. (B, C) Comparison of silhouette coefficients among scGraph2Vec and 11 tools using brain (B) and PBMC (C) datasets. The methods scapGNN and SAUCIE were excluded as they failed to extract clear gene clusters. The x-axis is the number of clusters. The y-axis is the silhouette coefficients and DBI. (D) Comparison of gene clustering similarity across methods using the Jaccard index. (E) Comparison of the similarity between gene clusters from different methods and the MSigDB hallmark gene sets.

The standard output of scGraph2Vec generated the most distinguishable clusters (Fig. 2A). Particularly, when excluding the gene feature matrix, there were hardly any clusters formed based on the resultant embeddings in any tissues tested (Supplementary Figs. S3A, B and S4A, B). Excluding or disrupting the primary community also obscured the formation of clusters (Supplementary Figs. S3D, E and S4D, E), indicating that the primary community of scGraph2Vec played important roles in generating gene clusters with similar behaviors in the network.

Additionally, we found that the reference network was critical to identifying gene clusters. We demonstrated this by randomizing the BioGRID network [35] or using alternative types of reference networks, such as the Reactome [69] network that characterizes biological pathways or the TRRUST [40] network that represents transcription regulatory relationships. In the former case, when using a randomized BioGRID network, we did not find any cluster (Supplementary Figs. S3C and S4C). In the latter case, gene clusters can be distinguished regardless of the type of biological network used (Supplementary Fig. S5). The 3 types of networks are typical scale-free networks with different network characteristics (Supplementary Fig. S6). We evaluated the effect of degree centrality on the clustering of genes in the 2-dimensional embedding space. By comparing gene clusters composed of hub genes with a varying degree of centrality and their neighbor genes, we found that scGraph2Vec is good at capturing small clusters with local connections, which is suitable for different types of biological networks (Supplementary Fig. S7). In contrast, the BioGRID network has a more uniform clustering effect. Thus, we used BioGRID as the main working network in the subsequent analysis.

Then, we compared the gene clusters identified using embedding trained on scRNA-seq and scATAC-seq data from the same brain samples [32]. Notably, when using scATAC-seq data, we constructed the feature matrix based on chromatin accessibility and mapped genomic regions to genes [76]. We observed most clusters could be replicated across different omics (Supplementary Fig. S8). This indicated that scGraph2Vec could detect gene clusters conserved in gene expression and chromatin accessibility through embeddings.

We used 64 logical CPUs and 512 GB RAM for all experiments. The execution time of scGraph2Vec depends on the size of the single-cell dataset and the interaction network. It ranges from 6.5 hours on the smallest dataset (PBMCs: 2,638 cells and 13,714 genes) to 37.3 hours on the largest dataset (heart: 287,269 cells and 33,694 genes). For the same brain dataset, the TRRUST network (the smallest in size: 2,862 genes and 8,427 interactions) takes 0.28 hours, while the BioGRID network (the largest in size: 19,752 genes and 977,356 interactions) takes 7.6 hours.

### Benchmark of scGraph2Vec with existing methods

We benchmarked scGraph2Vec using the brain [26] and PBMC [31] datasets, along with 13 embedding methods: ARGA [22], ARGVA [22], SIG-VAE [23], DGVAE [24], scVAE [14], LDVAE [12], scVI [11], siVAE [16], scapGNN [18], scGNN [13], scETM [15], SAUCIE [10], and SIMBA [17]. We uniformly extracted latent features of all genes for each method based on the same gene expression matrix, where the unfixed VAE variants all used the same-size encoder–decoder (256-64-16) structure. Notably, ARGA, ARGVA, SIG-VAE, and DGVAE were not designed specifically for biological data, and other methods were designed based on the gene expression matrix only (Supplementary Table S2).

Overall, scGraph2Vec showed more competitive results than other embedding methods in identifying gene clusters (Fig. 2B, C and Supplementary Fig. S9). Methods developed for cell embeddings, such as scVI, LDVAE, SAUCIE, and scETM, generally lose effectiveness in generating gene embeddings. Among the methods equipped with gene embedding, only siVAE demonstrated comparable cluster division to scGraph2Vec on the PBMC dataset (Fig. 2B, C). We found that the clusters identified by different methods have low similarities (Fig. 2D). This is counterintuitive since clusters of potentially interacting genes would be expected to be consistently identified by at least some of these methods. The low similarities were also found when we compared 2 commonly used gene clustering methods (SCENIC [49] and LIGER [50]) with scGraph2Vec (Supplementary Fig. S10). Then we introduced the MSigDB hallmark gene set [48] as the ground truth gene set to evaluate the biological meaning of different clusters. scGraph2Vec generated gene clusters that shared the most with the hallmark genes than other competing algorithms, indicating that the clusters by scGraph2Vec were functionally convergent (Fig. 2E).

### scGraph2Vec generated biologically meaningful clusters

We applied scGraph2Vec to generate gene embeddings for 6 representative human tissues and subsequently generated gene clusters (Supplementary Fig. S11 and Supplementary Table S3). As a result, we obtained an average of 134 gene clusters for each tissue, ranging from 108 to 149 (Supplementary Fig. S12). Each cluster contained tens to hundreds of genes, for example, 68 to 270 genes per cluster for the brain (Supplementary Table S3). We evaluated these clusters for their topological characteristics, annotated gene sets, and expression patterns.

**Figure 3:**
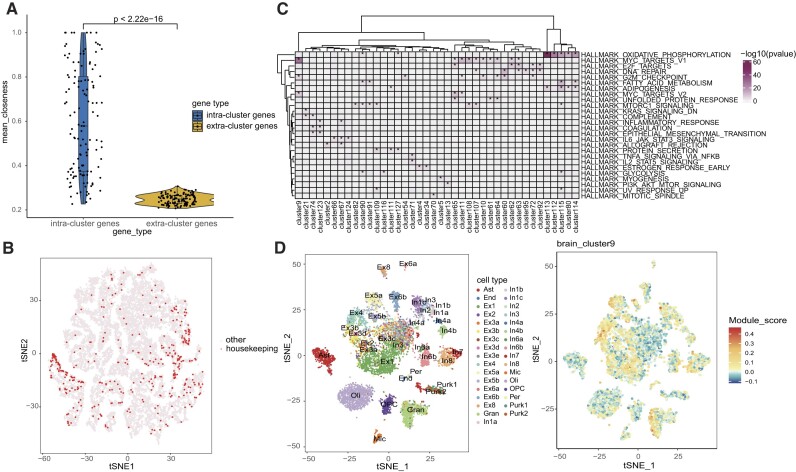
Biological implications of latent features from brain tissue. (A) Comparison of internal and external closeness centralities for all gene clusters using the Wilcoxon rank-sum test. Each dot represents a gene cluster. (B) The distribution of housekeeping genes in brain embeddings. (C) The gene clusters significantly enriched in hallmark gene sets (Fisher’s exact test, *P* < 0.05/50). (D) The left plot is the t-SNE plot of brain scRNA-seq data. The color indicates cell types labeled from the original article. The right plot shows the module score of each cell for brain cluster 9.

To test if the cluster genes were topologically correlated, we examined the closeness centrality of cluster genes. Taking the brain tissue as an example, the internal closeness centrality was significantly higher than the external closeness centrality of the clusters (*P* < 2.22 × 10^−16^, Fig. 3A). Thus, cluster genes were topologically related.

We next explored the biological implications of the clusters identified in different tissues using housekeeping genes and hallmark gene sets, as these genes were known to play critical functions in cells [51, 52]. We found that housekeeping genes tended to cluster together (Fig. 3B and Supplementary Fig. S13). As for the 50 hallmark gene sets, 26 were enriched in 40 gene clusters (Fig. 3C). The brain cluster 9 contained the highest number of housekeeping genes, with 59 genes, and enriched in hallmark gene sets related to MYC targets V1 (*P* = 6.83 × 10^−29^), MYC targets V2 (*P* = 1.42 × 10^−7^), and G2M checkpoint (*P* = 3.97 × 10^−5^).

To represent gene cluster expression across cell types, we computed the module score for each cluster at the single-cell level [26] (Fig. 3D). We observed highly variable expression patterns of clusters among different cell types (the top 20 highly variable clusters, Supplementary Fig. S14). Among them, brain cluster 9 had high module scores in most neurons and cerebellar granule cells but had low module scores in other cell types (Fig. 3D).

### scGraph2Vec generated clusters with cell-type and tissue specificity

**Figure 4:**
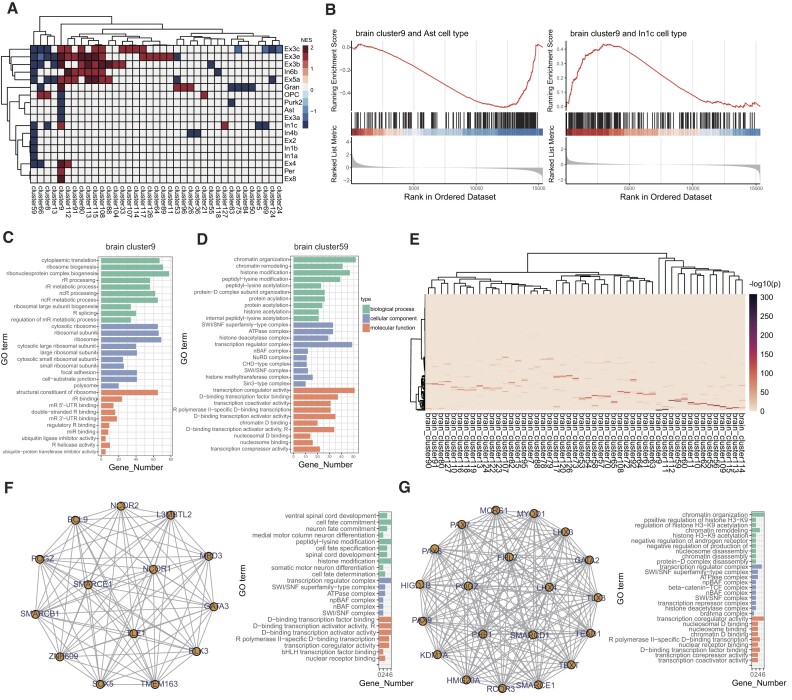
Tissue-specific clusters and functional analysis. (A) The heatmap of normalized enrichment score (NES) from GSEA results. Only significant enriched clusters and cell types are highlighted in color (*P_BH_* < 0.05). (B) The GSEA enrichment results for cluster 9 and 2 cell types (i.e., Ast and In1c). (C) GO enrichment for cluster 9. (D) GO enrichment for cluster 59. (E) The heatmap plot shows the brain-specific gene clusters. (F) The predicted subnetwork (left) and GO enrichment (right) of *SMARCE1* neighbor genes from embeddings of brain tissue. (G) The predicted subnetwork (left) and GO enrichment (right) of *SMARCE1* neighbor genes from embeddings of lung tissue.

Next, we tested if the cluster genes were enriched with cell type–specific genes using GSEA [47]. To this end, we defined cell type–specific genes as the DEGs for each cell type using the original scRNA-seq data [26]. Taking the brain tissue as an example, we found a total of 37 clusters significantly enriched with at least 1 cell type (*P*_BH_ < 0.05, Fig. 4A and Supplementary Table S4). Among them, cluster 9 was enriched with upregulated DEGs of the largest number of associated cell types, including excitatory neuronal (Ex) subtype 3c (Ex3c), Ex5a, Ex8, Ex3b, Ex4, Ex3e, inhibitory neuronal (In) subtype 1c (In1c), pericytes (Per), and cerebellar granule cells (Gran) (Fig. 4A, B and Supplementary Fig. S15). Gene Ontology (GO )enrichment analyses [65] showed that cluster 9 was related to functions such as cytoplasmic translation (*P*_BH_ = 1.02 × 10^−8^), ribosome biogenesis (*P*_BH_ = 8.75 × 10^−8^), ribonucleoprotein complex biogenesis (*P*_BH_ = 7.83 × 10^−5^), and ribonucleoprotein processing (*P*_BH_ = 1.68 × 10^−8^) (Fig. 4C). Another example is cluster 59, which was enriched in multiple excitatory and inhibitory neurons and was particularly related to downregulated DEGs in these cell types (Fig. 4A and Supplementary Fig. S15). Cluster 59 was mainly associated with functions of chromatin organization (*P*_BH_ = 1.59 × 10^−39^), histone modification (*P*_BH_ = 3.44 × 10^−31^), and histone acetylation (*P*_BH_ = 6.57 × 10^−16^) (Fig. 4D).

In the other 5 tissues, we identified 16 to 81 clusters enriched in cell-type DEGs of the corresponding tissue. In the lung tissue, 24 of 120 clusters were significantly enriched in DEGs of 27 lung cell types, including airway smooth muscle, capillary aerocytes, alveolar fibroblasts, and alveolar epithelial type 1 (*P*_BH_ < 0.05; Supplementary Fig. S16). In the heart tissue, 16 of 149 clusters were associated with DEGs of 15 heart cell types, such as cytoplasmic cardiomyocytes I and II, atrial cardiomyocytes, and ventricular cardiomyocytes I and II (*P*_BH_ < 0.05; Supplementary Fig. S17). In liver, 81 of 137 clusters were significantly enriched in DEGs of 19 liver cell types, including hepatocytes, central venous liver sinusoidal endothelial cells, and hepatic stellate cells (*P*_BH_ < 0.05; Supplementary Fig. S18). In addition, we identified a total of 143 clusters in kidney, of which 72 were related to the DEGs of kidney cell types (*P*_BH_ < 0.05; Supplementary Fig. S19). In PBMCs, 7 of 128 clusters were significantly enriched in DEGs of 4 cell types: B cells, CD14^+^ monocytes, memory CD4^+^ T cells, and naive CD4^+^ T cell (*P*_BH_ < 0.05; Supplementary Fig. S20).

Then we investigated the functions of the clusters and observed consistency in function and related cell types (Supplementary Fig. S21). The lung cluster 49 was enriched in cell types of alveolar epithelial type 1, alveolar fibroblasts, and airway smooth muscle. It was associated with functions of the chemokine-mediated signaling pathway (*P*_BH_ = 1.85 × 10^−6^) and negative regulation of endopeptidase activity (*P*_BH_ = 2.08 × 10^−6^). The heart cluster 80 was enriched in atrial cardiomyocytes and was associated with muscle cell development (*P*_BH_ = 1.68 × 10^−8^) and myofibril assembly (*P*_BH_ = 2.80 × 10^−6^). In the liver, several hepatocyte-enriched clusters were associated with distinct metabolic processes: cluster 35 with sulfur compound metabolic process (*P*_BH_ = 0.002) and gluconeogenesis (*P*_BH_ = 0.002), cluster 39 with organic acid catabolic process (*P*_BH_ = 1.31 × 10^−12^) and carboxylic acid catabolic process (*P*_BH_ = 8.30 × 10^−12^), and cluster 40 with nucleotide metabolic process (*P*_BH_ = 3.57 × 10^−7^). The kidney cluster 4 was associated with response to dietary excess (*P*_BH_ = 0.007) and fatty acid transport (*P*_BH_ = 0.007).

Next, we compared the cross-tissue differences in gene clusters. We defined tissue-specific clusters as those with no more than 5% significantly overlapping clusters, where overlap clusters were identified by shared genes with clusters from other tissues using Fisher’s exact test. For the brain tissue, we identified 50 of 127 clusters as brain specific, among which cluster 59, aforementioned as closely associated with the brain and enriched in genes of nBAF complex, was included (Fig. 4D, E). Then, we searched for genes located near *SMARCE1*, a key gene of the nBAF complex in the embedding space using the framework to identify candidate genes. We compared the neighbor genes found in the brain and nonbrain (i.e., lung) and reconstructed the predicted gene subnetwork (Fig. 4F, G). In the brain tissue, 14 genes were found in the neighboring subnetwork of *SMARCE1*, and these genes were enriched with a neuron fate commitment, medial motor column neuron differentiation, and somatic motor neuron differentiation, among others (Fig. 4F). In contrast, in the lung tissue, 20 genes were found adjacent to *SMARCE1*, but they were enriched with general functions related to chromatin organization and histone H3-K9 (Fig. 4G).

Collectively, we elucidated the high-dimensional biological information implied by gene embeddings in 6 representative human tissues and provided a general reference panel of tissue-related gene clusters.

### scGraph2Vec found candidate disease–associated genes

Large-scale GWAS have identified thousands of genetic associations with diseases. Disease-associated genes often interact with each other and jointly disturb multiple pathways or regulatory networks in disease tissues or cell types. Therefore, understanding how genes interact in a context is crucial to understanding the molecular mechanisms of diseases. We next explored the ability of scGraph2Vec to infer disease-associated genes by using the resultant gene embeddings and demonstrated it in COVID-19 and AD.

#### Application in COVID-19

**Figure 5:**
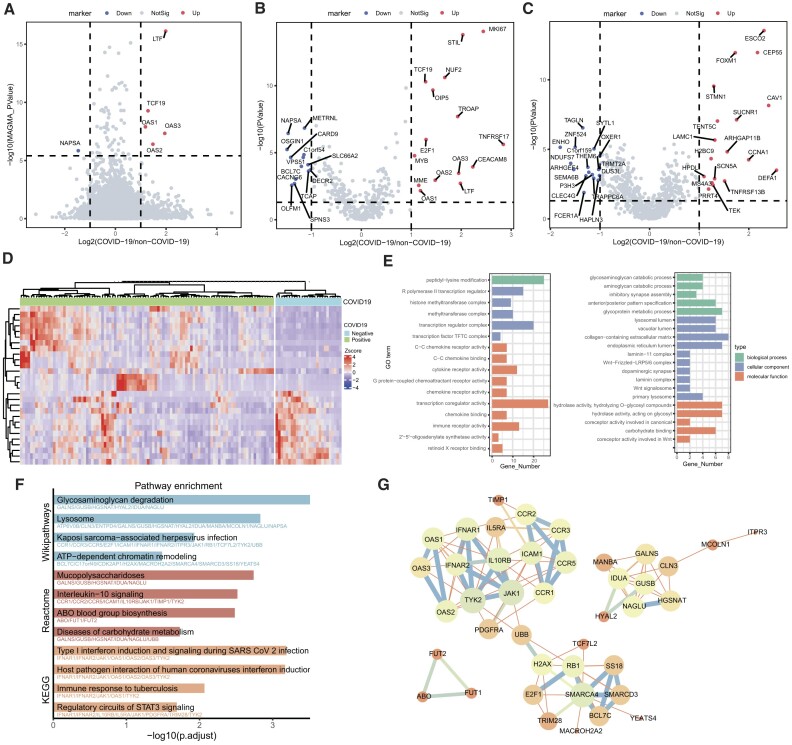
Inference of COVID-19–associated genes. (A) Volcano plot for GWAS-reported genes. The x-axis represents the log2(FC) of genes in COVID-19 and non–COVID-19 groups. The y-axis shows the gene-based *P* value from the MAGMA analysis (Bonferroni correction). The dashed lines represent the thresholds of significantly associated genes (*P* < 2.63 × 10^−6^) and differential expression genes (|log2(FC)| > 1). (B, C) Differential expression analysis of COVID-19–associated genes inferred from lung embeddings (B) and brain embeddings (C). The dashed lines represent the threshold of significant DEGs (*P*_BH_ < 0.05, |log2(FC)| > 1). (D) The expression profiles for COVID-19–associated candidate genes inferred from lung tissue. The samples in the columns were grouped by COVID-19–positive samples and COVID-19–negative samples, and the genes on the rows were clustered according to Ward’s method. (E) GO enrichment of all candidate genes (left) and *CEACAM8* neighbor genes (right) from lung tissue. (F) Pathway enrichment of all candidate genes based on the KEGG, WikiPathways, and Reactome databases. (G) STRING analysis for the candidate genes involved in the enriched pathways.

Using the HGI GWAS summary statistics for COVID-19 severity [55], we calculated gene-based *P* values by using MAGMA [57] and identified 60 significant genes for COVID-19 severity (Bonferroni-corrected threshold *P* < 2.63 × 10^−6^). Next, we used the 60 genes as the seeds and searched for their neighbor genes in the 2-dimensional embedding space of the lung tissue. As a result, we obtained 356 neighbor genes for COVID-19 severity. To validate these genes, we identified DEGs by comparing the bulk RNA-seq data from 102 COVID-19–positive patients and 26 COVID-19–negative individuals [58] (*P*_BH_ < 0.05 and |log2(FC)>1|) following the original study [58]. Notably, for the 60 GWAS-implied genes, only 6 were DEGs (Fig. 5A). In contrast, 28 of 356 neighbor genes were validated to be DEGs. This proportion, although only marginally significant (*P* = 0.067, hypergeometric test; Fig. 5B), indicated that the gene embeddings indeed could be used to identify more disease-associated genes. This was further proved when we conducted the analyses using a disease-irrelevant tissue (i.e., brain), where 35 of the 681 identified neighbor genes were DEGs but were not statistically significant (*P* = 0.83; Fig. 5C). The 28 genes showed distinct expression patterns in COVID-19 and non–COVID-19 samples (Fig. 5D). Importantly, many genes were identified but were missed by the original GWAS results, such as *MKI67, STIL, NUF2, OIP5, TNFRSF17*, and *CEACAM8*. The 356 neighbor genes mainly related to cytokine receptor activity (*P*_BH_ = 4.86 × 10^−6^), C-C chemokine receptor activity (*P*_BH_ = 4.86 × 10^−5^), and peptidyl-lysine modification (*P*_BH_ = 8.23 × 10^−5^), among other functions (Fig. 5E left plot). Pathway enrichment analysis further revealed significant enrichment in glycosaminoglycan degradation (*P*_BH_ = 3.08 × 10^−4^), type I interferon induction and signaling during SARS-CoV-2 infection (*P*_BH_ = 6.46 × 10^−4^), interleukin-10 signaling (*P*_BH_ = 3.03 × 10^−3^), and ABO blood group biosynthesis (*P*_BH_ = 3.29 × 10^−3^) (Fig. 5F). STRING analysis further demonstrated protein–protein interactions among genes involved in these pathways (Fig. 5G). We particularly examined the 8 neighbor genes of the gene *CEACAM8*. These genes were enriched in biological processes such as the glycosaminoglycan (GAG) catabolic process (*P*_BH_ = 3.19 × 10^−4^, Fig. 5E, right plot). GAGs serve as receptors for numerous microbial pathogens to adhere to and invade cells [77]. Recent evidence has shown that the entry process of SARS-CoV-2 into host cells was mediated by the transmembrane spike (S) protein interacting with both cellular heparan sulfate GAG and angiotensin converting enzyme 2 (ACE2) [78, 79]. Thus, GAG derivatives have been promising candidates for SARS-CoV-2 antiviral therapy [80, 81].

#### Application in Alzheimer's disease

**Figure 6:**
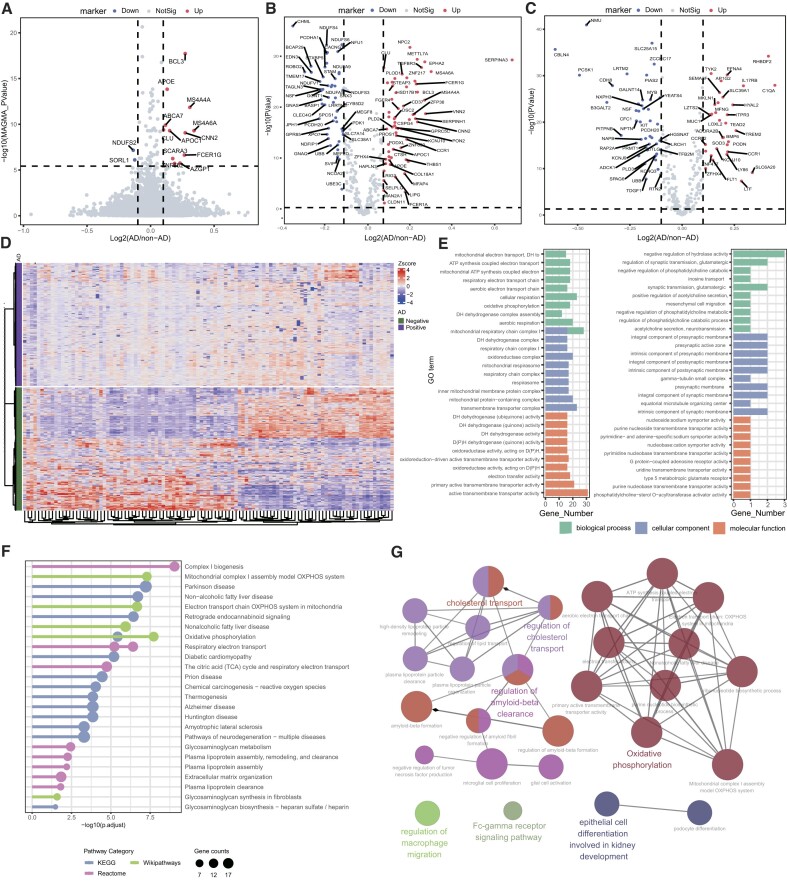
Inference of AD-associated genes. (A) Volcano plot for GWAS-reported genes. The x-axis represents the log2 (FC) of genes in AD and non-AD groups. The y-axis shows the gene-based *P* value by MAGMA (after Bonferroni correction). The dashed lines represent the threshold of significant associated genes (*P* < 3.77 × 10^−6^) and differential expression gene threshold (|fold change| > 1.1). (B, C) Differential expression analysis AD-associated genes inferred from brain embeddings (B) and lung embeddings (C). The dashed lines represent the threshold of significant DEGs (*P*_BH_ < 0.05, |fold change| > 1.1). (D) AD-associated gene expression profiles inferred from brain tissue. The samples in the columns were grouped by AD-positive samples and AD-negative samples, and the genes on the rows were clustered according to Ward’s method. (E) GO analysis of all neighbor genes (left) and *APOE* neighbor genes (right) from brain tissue. (F) Pathway enrichment of all candidate genes based on the KEGG, WikiPathways, and Reactome databases. (G) Pathway annotation for all candidate genes using the ClueGO software.

Using a large-scale GWAS for AD (71,880 cases and 383,378 controls) [56], we identified 65 AD-associated genes by MAGMA at the Bonferroni-corrected threshold *P* < 3.77 × 10^−6^. Fourteen of 65 genes were DEGs (*P*_BH_ < 0.05 and |fold change| > 1.1) between the AD group and the control group using an independent bulk RNA-seq data [59] (postmortem brain tissues of 376 late-onset AD patients and 173 normal samples, Fig. 6A). Using the 65 genes as seed genes and the gene embeddings for the normal brain tissue, we identified 362 neighbor genes for AD. These newly identified AD-candidate genes were significantly enriched with DEGs (112/362, hypergeometric test *P* = 0.011, Fig. 6B). As a negative control, we found 317 neighbor genes near the 65 seed genes using the gene embeddings generated for an irrelevant tissue (i.e., the healthy lung tissue). However, only 78 of 317 genes were DEGs, and this proportion was not statistically significant (*P* = 0.68, hypergeometric test; Fig. 6C). These neighbor genes identified by latent features can well distinguish AD patients from healthy controls (Fig. 6D). Functional enrichment analysis of these genes indicated interesting GO terms that might be important pathogenic causes for AD, such as the mitochondrial electron transport chain (*P*_BH_ = 2.75 × 10^−8^; Fig. 6E, left plot) [82]. Pathway annotation of 362 genes revealed regulatory pathways involved in AD, mainly complex I biogenesis (*P*_BH_ = 9.00 × 10^−10^), cholesterol transport (*P*_BH_ = 1.07 × 10^−4^), regulation of cholesterol transport (*P*_BH_ = 9.48 × 10^−4^), regulation of amyloid-beta clearance (*P*_BH_ = 9.83 × 10^−5^), and oxidative phosphorylation (*P*_BH_ = 3.67 × 10^−9^) (Fig. 6F, G). Furthermore, the newly identified AD-candidate genes can help to better explain the molecular mechanisms of potential targets. For example, we found a causal gene *SERPINA3* that had been verified to be associated with AD but had never been detected by GWAS [83]. Another AD risk gene, *APOC1*, and its 8 neighbor genes (such as *ADORA2A, CDH2*, and *SLC28A2*) were enriched in functions related to negative regulation of hydrolase activity (*P*_BH_ = 0.01), regulation of synaptic transmission, glutamatergic (*P*_BH_ = 0.01), negative regulation of phosphatidylcholine catabolic process (*P*_BH_ = 0.01), and so on (Fig. 6E right plot).

### scGraph2Vec identified candidate driver genes in LUAD

**Figure 7:**
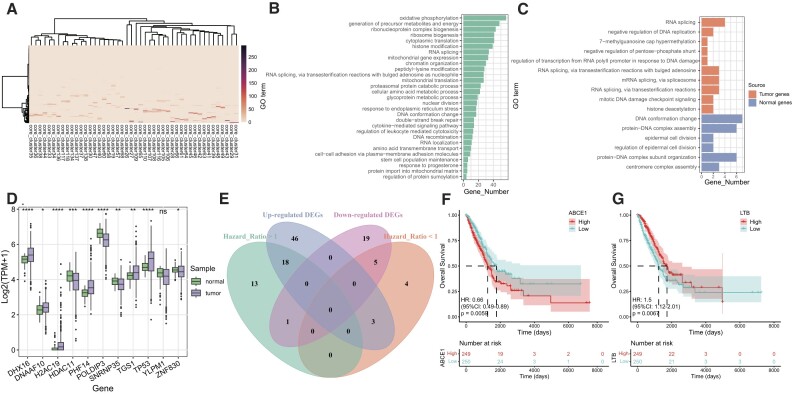
Discovery of additional tumor-driver genes. (A) The heatmap plot of the tumor-specific gene clusters. (B) The most significant GO terms enriched for tumor-specific clusters. Only biological processes were shown. (C) The top 10 GO terms (biological process) enriched in *TP53* neighbor genes (sorted by *P*_BH_). (D) Boxplots showing the expression differences of *TP53* neighbor genes in TCGA LUAD samples. The *P* values were from the Wilcoxon test using log2 (TPM + 1). *P < 0.05, **P < 0.01, ***P < 0.001, ^****^P < 0.0001. (E) Venn diagram showing the overlap of the 4 gene sets: upregulated DEGs, downregulated DEGs, genes with hazard ratio (HR) >1, and genes with HR <1. (F, G) Kaplan–Meier curve based on the survival time (days) of TCGA LUAD samples and the expression levels of *ABCE1* and *LTB* genes. Samples were grouped based on the median of log2 (TPM + 1).

We further illustrated the effectiveness of scGraph2Vec in identifying candidate driver-like genes in cancer. Using scRNA-seq data from LUAD patients [33], we generated embeddings for tumor and normal lung tissues, respectively, and identified gene clusters in each condition (Supplementary Fig. S22A, B and Supplementary Table S3). Notably, in the 2-dimensional embedding space, we observed a partial enrichment of housekeeping genes (Supplementary Fig. S22C, D). By comparing the overlapping genes in the clusters from either normal tissue or tumor, we identified 41 clusters specifically enriched in tumor (Fig. 7A). These tumor-specific clusters were enriched in functions related to oxidative phosphorylation (*P*_BH_ = 1.13 × 10^−87^), double-strand break repair (*P*_BH_ = 1.45 × 10^−7^), cytokine-mediated signaling pathway (*P*_BH_ = 4.14 × 10^−6^), cell–cell adhesion via plasma–membrane adhesion molecules (*P*_BH_ = 0.005), and stem cell population maintenance (*P*_BH_ = 0.004) (Fig. 7B). Then, we used the 18 LUAD-driver genes reported by TCGA as seeds (*ARID1A, BRAF, CDKN2A, EGFR, KEAP1, KRAS, MET, MGA, NF1, PIK3CA, RB1, RBM10, RIT10, SETD2, SMARCA4, STK11, TP53*, and *U2AF1*) [60] and identified 251 neighbor genes in the embeddings of tumor and 505 neighbor genes in normal tissue. Notably, we observed that the enrichment of these neighbor genes aligned with the tissue context of the respective samples. For example, *TP53* neighbor genes in normal tissue are enriched for functions like DNA conformation change (*P*_BH_ = 0.005), protein–DNA complex assembly (*P*_BH_ = 0.005), and epidermal cell division (*P*_BH_ = 0.005). However, *TP53* neighbor genes in tumor are enriched for functions related to tumor development, such as negative regulation of DNA replication (*P*_BH_ = 0.02), 7-methylguanosine cap hypermethylation (*P*_BH_ = 0.02), negative regulation of pentose-phosphate shunt (*P*_BH_ = 0.02), and mitotic DNA damage checkpoint signaling (*P*_BH_ = 0.03) (Fig. 7C).

To validate the neighbor genes, we downloaded bulk RNA-seq data of LUAD samples from TCGA, including expression data for 238 of the 251 neighbor genes identified in the tumor. Among all 238 neighbor genes, 109 were DEGs (|log2(FC)| > 0.5 and *P*_BH_ < 0.05). Specifically, for *TP53*, 9 out of 10 neighbor genes were DEGs (Fig. 7D, Wilcoxon rank-sum test, *P* < 0.05). Subsequently, using the univariate Cox proportional hazards model, we discovered 44 prognosis-related genes (*P* < 0.05), among which 27 genes were DEGs (Fig. 7E). Then we conducted survival analysis using each of the 27 genes to stratify samples by the median of log2 (TPM + 1) as the cutoff value. High expression of 13 genes and low expression of 5 genes were associated with poor prognosis in LUAD patients (Fig. 7F, G and Supplementary Fig. S23). Several were recognized as LUAD-related genes, including *ABCE1* [84], *CCNE1* [85], *CHCHD2* [86], *ERG* [87], *HOXA1* [88], *KRAS* [89], *KRT17* [90], *LHX2* [91], *LTB* [92], and *SHMT2* [93], among others.

### scGraph2Vec revealed regulatory genes underlie cell state transitions in melanoma

**Figure 8:**
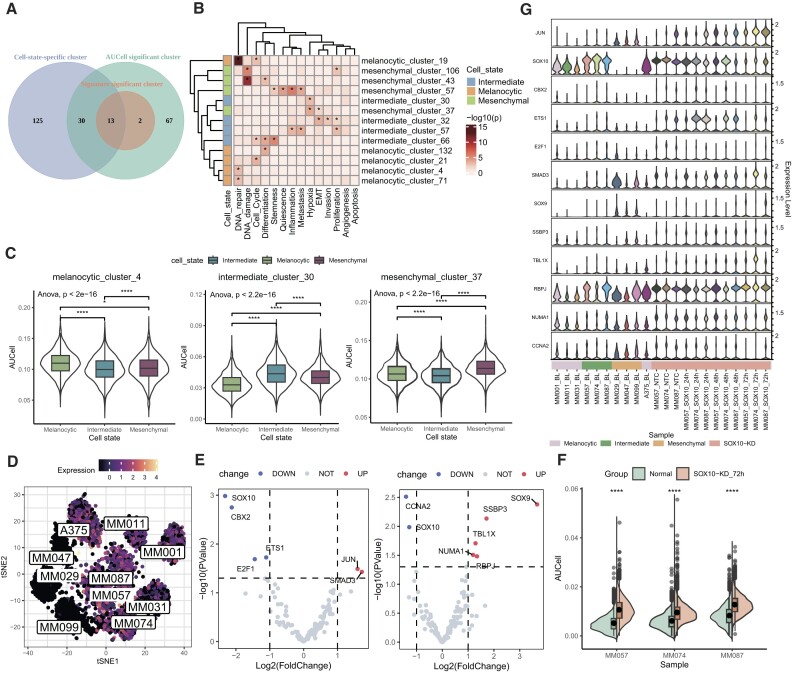
Discovery of regulatory genes for cell state transitions. (A) Venn diagram of the overlapping genes among 3 types of clusters: clusters specific to cell states, clusters with significant differences in AUCell scores between states, and clusters with enriched functional signatures. (B) The enrichment results for 14 functional state signatures and gene clusters from 3 cell states (Fisher’s exact test, *P* < 0.05/14). (C) Cellular activity of gene clusters in 3 states measured by AUCell scores. One-way ANOVA was used to test across multiple cell states and a 2-sample *t*-test was used for comparisons between cell states. *P < 0.05, **P < 0.01, ***P < 0.001, ^****^P < 0.0001. (D) Expression distribution of *SOX10* in different cell lines. (E) Differential expression analysis of *SOX10* neighbor genes inferred from melanocytic embeddings (left) and intermediate embeddings (right). The dashed lines represent the threshold of significant DEGs (*P* < 0.05, |log2(FC)| > 1). (F) Comparison of cell activity (AUCell) changes between normal and *SOX10*-KD samples. The 2-sample *t*-test was used to compare the activity between normal and *SOX10*-KD samples. *P < 0.05, **P < 0.01, ***P < 0.001, ^****^P < 0.0001. (G) Stacked violin plot showing expression of validated *SOX10* neighboring genes in scRNA-seq data.

scGraph2Vec can generate gene embeddings for different cell states and reveal gene regulatory programs underlying cell state stabilization and transitions. We demonstrated it using melanoma scRNA-seq data, including cells of 3 melanocyte cell states. Based on the gene embeddings, we identified 149 clusters for the melanocytic state (62 significantly specific to melanocytic), 149 for the intermediate state (59 specific), and 133 for the mesenchymal state (47 specific) (Supplementary Table S3). Furthermore, using AUCell [63] to quantitatively assess the cluster activities, we found that 120 of 149 melanocytic clusters, 122 of 149 intermediate clusters, and 37 mesenchymal clusters showed significant differences across 3 states (*P* < 0.05 for ANOVA and *t*-test; high activity). Then we identified gene clusters that reflect distinct cellular functional states based on the gene signatures of 14 crucial cancer cell states [64]. In total, we discovered 15 gene clusters that were significantly enriched with these functional signatures (Fisher’s exact test, *P* < 0.05/14). These clusters exhibited notable differences in AUCell activity, with the majority (13 out of 15) being cell state specific (Fig. 8A). We observed that gene clusters in the melanocytic state were primarily associated with the DNA repair, cell cycle, and differentiation, while those in the mesenchymal state were enriched for DNA damage, quiescence, inflammation, metastasis, hypoxia, EMT, and so on (Fig. 8B). The intermediate state, acting as a transitional phase, displayed features of both (Fig. 8B). Among 3 cell states, melanocytic cluster 4, intermediate cluster 30, and mesenchymal cluster 37 showed the most significant increase in AUCell activity (Fig. 8C), each representing a distinct functional state: melanocytic cluster 4 was enriched in DNA repair, intermediate cluster 30 in hypoxia responses, and mesenchymal cluster 37 in EMT and hypoxia responses. Consistent with previous studies, melanocytic cells were characterized by a high proliferation rate, mesenchymal cells exhibited high invasive capacity, and intermediate cells presented with mixed characteristics shared with the other 2 states [94–96]. Also, EMT is a process where melanocytes lose their epithelial characteristics and acquire a mesenchymal phenotype, which enhances melanoma cell motility, invasiveness, and metastatic potential [97, 98].

In addition to identifying state-specific gene clusters, scGraph2Vec can also help identify critical genes during cell state transitions. Previous studies have reported that the transcription factor *SOX10* drives the dynamic transition of melanoma cells from a melanocytic state to a mesenchymal state [34, 99]. *SOX10* was found to be highly expressed in 7 cell cultures at the melanocytic and intermediate states (MM001, MM011, MM031, MM057, MM074, MM087, and A375) (Fig. 8D). Using the embeddings generated by scGraph2Vec, we identified 169 neighbor genes of *SOX10* in the melanocytic state and 182 in the intermediate state, respectively. Using the bulk RNA-seq data generated at 72 hours after *SOX10* knockdown, we identified 555 DEGs (|log2(FC)| > 1 and *P* < 0.05) for the melanocytic state. Five of these DEGs were included in the *SOX10* neighbor genes, which were *E2F1, JUN, SMAD3, ETS1*, and *CBX2* (Fig. 8E, left panel). Similarly, among the 498 DEGs for the intermediate state, 6 were included in our neighbor genes, including those involved in cell differentiation and cell cycle processes, such as *SOX9, CCNA2, TBL1X*, and *NUMA1*, as well as immune signaling-related genes like *RBPJ* (Fig. 8E, right panel). STRING analysis revealed close protein–protein interactions among these genes (Supplementary Fig. 24). We further validated the 182 neighbor genes for the intermediate state where scRNA-seq data were available. As shown in Fig. 8F, *SOX10-*neighbor genes showed significantly increased activity 72 hours after *SOX10* knockdown in the intermediate-state cells compared to the control cells (*t*-test, *P* < 0.05). This was observed in all 3 samples with the intermediate state (Fig. 8F). Most of these neighboring genes were highly expressed in the mesenchymal state (Fig. 8G), with *SOX9, JUN*, and *SMAD3* showing significantly high expression (log2(FC) > 1 and *P*_BH_ < 0.05). In summary, scGraph2Vec can infer neighboring genes of interest in different cell states based on gene embeddings, providing insights into cell state–specific regulatory relationships.

## Discussion

We developed scGraph2Vec based on GNN to represent tissue-specific gene embeddings by integrating heterogeneous information of gene interaction networks and single-cell gene matrices. On benchmark datasets, we showed that scGraph2Vec outperformed competing algorithms by generating gene embeddings that can delineate gene clusters and cluster functionally meaningful genes. Using scRNA-seq data from 6 types of human normal tissues and 3 different types of gene networks, we elucidated that gene embeddings can capture the topological features of various biological networks, cluster sets of co-functioning genes, and reveal tissue- or cell type–specific gene clusters. Additionally, we highlighted application cases in COVID-19, AD, LUAD, and melanoma, showing that scGraph2Vec is versatile in analyzing samples from normal human tissues, case-control tumor samples, and cell lines in different states. By searching for neighboring genes in the embedding space, we identified additional disease-associated genes and revealed gene regulatory programs across diverse biological contexts. Since gene embeddings accurately measure and integrate molecular patterns at different levels, they provide more complete associations between genes and phenotypes in tissues, effectively complementing strategies for studying disease-associated genes.

The precise description of genes in complex multicellular organisms depends on the interaction between genes and gene products in response to the cellular environment. The rapidly accumulated single-cell omics data have provided an unprecedented opportunity to identify the cellular processes of genes under different cell lineages and environmental changes. However, it is challenging to accurately infer the regulatory programs of genes in cell lineages from these data. One is that most of the data have heterogeneous structures and are generated from different experimental designs and technical platforms. In addition, experimental techniques for generating high-throughput tissue-specific gene interactions are currently unfeasible, especially for cell line samples that are not readily available. With few exceptions, existing networks often lack fine-grained regulation, resulting in disease modules that are either overly fragmented or too extensive, making observable disease modules less discernible [100, 101]. Therefore, there is an urgent need to develop comprehensive computational methods to integrate multilevel genome data and provide systematic explanations for tissue-specific regulatory networks. Our approach integrated a gene interaction network with single-cell gene profiles, allowing for extension to various types of gene networks and single-cell data across different biological contexts. This scalability is especially important in an era when there is already a large amount of publicly available gene networks and single-cell omics data. The gene embeddings generated from scGraph2Vec can provide the necessary background panel for gene regulation, identify co-functional gene modules, and contribute to studying pathogenic mechanisms of disease-associated genes.

One limitation of scGraph2Vec is that it relies on the data quality of the gene feature matrix that provides tissue-specific information. In this work, we used publicly available scRNA-seq data from human normal tissues, tumor tissues, and cell lines. The scRNA-seq data are noisy and incomplete, especially since they come from specific sampling sites and different experimental designs. Graph embedding can reduce some technical noises but cannot impute the unknown tissue-specific information. Second, the boundaries of gene clusters are difficult to determine. The regulation pattern of gene sets in a given tissue is ambiguous. The physical distance between genes in the embedding space extracted by scGraph2Vec does not represent the actual distance of the regulatory relationship between genes, because it often depends on the gene interaction network and gene feature matrix used in the model training. Furthermore, scGraph2Vec is better at capturing small clusters with local connections, making it more suitable for scale-free networks with numerous sparse connections, a characteristic of most biological interaction networks. To increase the adaptability and interpretability of our method, we recommend the use of multiple single-relationship gene interaction networks for the analyses. Finally, scGraph2Vec is a unified and highly scalable framework for integrative analysis of various networks and single-cell datasets from a given tissue. We therefore expect this work to be of interest, especially since tissue-specific gene interaction networks are lacking and needed for various types of downstream analysis.

## Availability of Source Code and Requirements

Project name: scGraph2Vec

Project homepage: https://github.com/LPH-BIG/scGraph2Vec

Operating system: Platform independent

Programming language: Python

Other requirements: Python 3.7 or higher, TensorFlow 1.15.0

License: MIT License

RRID: SCR_025322

biotoolsID: scGraph2Vec

## Supplementary Material

giae108_GIGA-D-24-00200_Original_Submission

giae108_GIGA-D-24-00200_Revision_1

giae108_GIGA-D-24-00200_Revision_2

giae108_Response_to_Reviewer_Comments_Original_Submission

giae108_Response_to_Reviewer_Comments_Revision_1

giae108_Reviewer_1_Report_Original_Submission Bin Li -- 7/12/2024 Reviewed

giae108_Reviewer_1_Report_Revision_1 Bin Li -- 10/25/2024 Reviewed

giae108_Reviewer_2_Report_Original_Submission Saptarshi Pyne -- 7/15/2024 Reviewed

giae108_Reviewer_2_Report_Revision_1 Saptarshi Pyne -- 11/2/2024 Reviewed

giae108_Reviewer_3_Report_Original_Submission Yuansong Zeng -- 7/16/2024 Reviewed

giae108_Reviewer_3_Report_Revision_1 Yuansong Zeng -- 11/5/2024 Reviewed

giae108_Supplemental_Files

## Data Availability

The scRNA-seq data used in this article are all publicly available. The liver tissue data and paired scRNA-seq and scATAC-seq data can be accessed from GEO, with the following access numbers: GSE115469 and GSE162170, respectively. Other scRNA-seq datasets for this article are available via the following databases: brain dataset from the human cell landscape [102]; heart dataset from the Single Cell Portal [103], with accession code SCP498; kidney dataset from the Kidney Cell Atlas, specifically the mature kidney dataset [28]; lung dataset from the Human Lung Cell Atlas [30] with Synapse ID: syn21041850; PBMC dataset from the 10X Genomics 3k PBMC supporting dataset; and LUAD dataset from EMBL-EBI database, with accession codes E-MTAB-6149 and E-MTAB-6653. Furthermore, the RNA-seq data from the brain and plasma are available as supplementary data of their respective articles [58, 59]. The RNA-seq data for LUAD are from the TCGA database with project ID TCGA-LUAD [60]. The melanoma datasets were downloaded from the Scope (Scope session: Wouters_Human_Melanoma) and GEO (accession ID: GSE134432). The data and code for transparent and reproducible results are available in Zenodo [104] and GitHub [105]. DOME-ML annotations are available via the DOME Registry [106]. Other data further supporting this work are openly available in the *GigaScience* repository, GigaDB [107].

## Additional Files

**Supplementary Table S1**. Statistics of the datasets used.

**Supplementary Table S2**. Summary of methods used for comparison.

**Supplementary Table S3**. Gene clusters identified by scGraph2Vec in 6 representative human tissues, tumor/normal tissues of LUAD, and 3 melanocyte cell states.

**Supplementary Table S4**. The results of applying GSEA to identify potential enrichment of gene clusters among brain cell type-specific genes.

**Supplementary Fig. S1**. The hyperparameter sweep result.

**Supplementary Fig. S2**. The evaluation of model stability in multiple ways.

**Supplementary Fig. S3**. Performance evaluation of the key components in scGraph2Vec by varying its structure using the brain dataset.

**Supplementary Fig. S4**. Performance evaluation of the key components in scGraph2Vec by varying its structure using the PBMC dataset.

**Supplementary Fig. S5**. Clustering results based on the embeddings obtained by using the Reactome network (left) and the TRRUST network (right), respectively.

**Supplementary Fig. S6**. Characteristics of 3 biological networks.

**Supplementary Fig. S7**. Evaluation of the clustering of genes grouped by varying degrees of centrality.

**Supplementary Fig. S8**. Gene clusters cross-validated using scRNA-seq and scATAC-seq data from the same brain samples.

**Supplementary Fig. S9**. Comparison of gene embedding among methods on the same brain and PBMC datasets.

**Supplementary Fig. S10**. Clustering measurement of SCENIC and LIGER gene clusters in scGraph2Vec 2-dimensional embedding space.

**Supplementary Fig. S11**. Gene clusters in 6 human tissues.

**Supplementary Fig. S12**. The optimal clustering was selected by silhouette coefficient in 10 replications.

**Supplementary Fig. S13**. The distribution of housekeeping genes in latent features from 6 human tissues.

**Supplementary Fig. S14**. Scaled module scores in cells for the top 20 highly variable clusters.

**Supplementary Fig. S15**. GSEA analysis between the gene clusters (cluster 9 and cluster 59) and cell types in the brain dataset.

**Supplementary Fig. S16**. GSEA for lung clusters.

**Supplementary Fig. S17**. GSEA for heart clusters.

**Supplementary Fig. S18**. GSEA for liver clusters.

**Supplementary Fig. S19**. GSEA for kidney clusters.

**Supplementary Fig. S20**. GSEA for PBMC clusters.

**Supplementary Fig. S21**. GO enrichment analysis for cluster genes.

**Supplementary Fig. S22**. Gene clusters in the tumor tissue (A) and the normal tissue (B). Housekeeping genes in latent features from the tumor tissue (C) and the normal tissue (D).

**Supplementary Fig. S23**. Kaplan–Meier survival analysis of 16 out of 18 overlap candidate genes.

**Supplementary Fig. S24**. STRING analysis for the 12 validated *SOX10* neighbor genes.

## Abbreviations

AD: Alzheimer's disease; DEG: differentially expressed gene; DBI: Davies–Bouldin index; Ex: excitatory neuronal; EMT: epithelial–mesenchymal transition; GAG: glycosaminoglycan; GCN: graph convolutional network; GNN: graph neural network; Gran: cerebellar granule cells; GSEA: gene set enrichment analysis; GO: Gene Ontology; GWAS: genome-wide association studies; HR: hazard ratio; In: inhibitory neuronal; iNMF: integrative nonnegative matrix factorization; KD: knockdown; LTMG: Left Truncated Mixture Gaussian; LUAD: lung adenocarcinoma; NSCLC: non–small cell lung cancer; PBMC: peripheral blood mononuclear cell; Per: pericytes; PPI: protein–protein interaction; TCGA: The Cancer Genome Atlas; t-SNE: t-distributed stochastic neighbor embedding; TPM: transcript per million; VGAE: variational graph autoencoder.
