# Supplementary material for: scGraph2Vec: a deep generative model for gene embedding augmented by graph neural network and single-cell omics data
Source: Gigascience. 2024 Dec 20;13:giae108. doi: 10.1093/gigascience/giae108 (PMC11659981; doi:10.1093/gigascience/giae108)
Supplement: giae108_GIGA-D-24-00200_Original_Submission [file giae108_giga-d-24-00200_original_submission.pdf]

GigaScience

scGraph2Vec: a deep generative model for gene embedding augmented by Graph Neural Network and single-cell omics data

--Manuscript Draft--

**Manuscript Number:**	GIGA-D-24-00200
**Full Title:**	scGraph2Vec: a deep generative model for gene embedding augmented by Graph Neural Network and single-cell omics data
**Article Type:**	Technical Note
**Funding Information:**	the Strategic Priority Research Program of the Chinese Academy of Sciences (XDB38010400) — Prof. Peilin Jia<br>the National Natural Science Foundation of China (32270706) — Prof. Peilin Jia
**Abstract:**	Background<br><br>Exploring the cellular processes of genes from the aspects of biological networks is of great interest to understanding the properties of complex diseases and biological systems. Biological networks, such as gene regulatory networks and protein-protein interaction networks, can provide insights into the molecular basis of cellular processes and functions and often form different modules in different tissue and disease contexts.<br><br>Results<br><br>Here, we present scGraph2Vec, a deep-learning framework for generating gene embeddings that are highly informative by incorporating single-cell data patterns of neighbor genes and communities. By extending the variational graph autoencoder (VGAE) framework and incorporating gene interaction networks, scGraph2Vec is more expressive than existing methods in representing high-dimensional gene information and clustering functional modules. We demonstrate that the gene embeddings are biologically interpretable and could reveal functional gene modules representing general or tissue-specific cellular processes. When applied to the genome-wide association study data (e.g., COVID-19 and Alzheimer’s Disease), the embeddings can infer disease-associated genes. Furthermore, by obtaining gene embeddings using lung adenocarcinoma (LUAD) and matched normal samples, we gain insights into the functions of known driver genes and identify additional key genes in LUAD.<br><br>Conclusions<br><br>scGraph2Vec not only reconstructs tissue-specific gene networks but also obtains a low-dimensional representation of genes implying their biological functions.
**Corresponding Author:**	Peilin Jia, Ph.D.<br>Beijing Institute of Genomics Chinese Academy of Sciences<br>Beijing, CHINA
**Corresponding Author Secondary Information:**	
**Corresponding Author's Institution:**	Beijing Institute of Genomics Chinese Academy of Sciences
**Corresponding Author's Secondary Institution:**	
**First Author:**	Shiqi Lin
**First Author Secondary Information:**	
**Order of Authors:**	Shiqi Lin<br>Peilin Jia, Ph.D.
**Order of Authors Secondary Information:**	

Powered by Editorial Manager® and ProduXion Manager® from Aries Systems Corporation

Additional Information:	
**Question**	**Response**
Are you submitting this manuscript to a special series or article collection?	No
**Experimental design and statistics**<br><br>Full details of the experimental design and statistical methods used should be given in the Methods section, as detailed in our Minimum Standards Reporting Checklist. Information essential to interpreting the data presented should be made available in the figure legends.<br><br>Have you included all the information requested in your manuscript?	Yes
**Resources**<br><br>A description of all resources used, including antibodies, cell lines, animals and software tools, with enough information to allow them to be uniquely identified, should be included in the Methods section. Authors are strongly encouraged to cite Research Resource Identifiers (RRIDs) for antibodies, model organisms and tools, where possible.<br><br>Have you included the information requested as detailed in our Minimum Standards Reporting Checklist?	Yes
**Availability of data and materials**<br><br>All datasets and code on which the conclusions of the paper rely must be either included in your submission or deposited in publicly available repositories (where available and ethically appropriate), referencing such data using a unique identifier in the references and in the “Availability of Data and Materials” section of your manuscript.	Yes

Powered by Editorial Manager® and ProduXion Manager® from Aries Systems Corporation

Have you have met the above requirement as detailed in our Minimum Standards Reporting Checklist?	

Powered by Editorial Manager® and ProduXion Manager® from Aries Systems Corporation

Manuscript

Click here to access/download;Manuscript;scGraph2Vec.Giga.final.docx

scGraph2Vec: a deep generative model for gene embedding augmented by Graph Neural Network and single-cell omics data

Shiqi Lin[1,2], Peilin Jia[1,2,*]

[1]CAS Key Laboratory of Genomic and Precision Medicine, Beijing Institute of Genomics, Chinese Academy of Sciences and China National Center for Bioinformation, Beijing 100101, China

[2]University of Chinese Academy of Sciences, Beijing 100049, China

[*]Address correspondence to:

Peilin Jia, Ph.D.

Key Laboratory of Genomic and Precision Medicine, Beijing Institute of Genomics, Chinese Academy of Sciences and China National Center for Bioinformation

No. 1 Beichen West Road 1-104, Chaoyang District, Beijing, 100101, China

Phone: (086)-010-84097798, Email: pjia@big.ac.cn

ORCID

Shiqi Lin: 0000-0001-9518-2027, Peilin Jia: 0000-0003-4523-4153

Running title

scGraph2Vec: a deep generative model for gene embedding

ABSTRACT

Background

Exploring the cellular processes of genes from the aspects of biological networks is of great interest to understanding the properties of complex diseases and biological systems. Biological networks, such as gene regulatory networks and protein-protein interaction networks, can provide insights into the molecular basis of cellular processes and functions and often form different modules in different tissue and disease contexts.

Results

Here, we present scGraph2Vec, a deep-learning framework for generating gene embeddings that are highly informative by incorporating single-cell data patterns of neighbor genes and communities. By extending the variational graph autoencoder (VGAE) framework and incorporating gene interaction networks, scGraph2Vec is more expressive than existing methods in representing high-dimensional gene information and clustering functional modules. We demonstrate that the gene embeddings are biologically interpretable and could reveal functional gene modules representing general or tissue-specific cellular processes. When applied to the genome-wide association study data (e.g., COVID-19 and Alzheimer's Disease), the embeddings can infer disease-associated genes. Furthermore, by obtaining gene embeddings using lung adenocarcinoma (LUAD) and matched normal samples, we gain insights into the functions of known driver genes and identify additional key genes in LUAD.

Conclusions

scGraph2Vec not only reconstructs tissue-specific gene networks but also obtains a low-dimensional representation of genes implying their biological functions.

Keywords: gene embedding, gene regulatory network, single-cell RNA-seq, tissue specificity, complex disease

BACKGROUND

Our understanding of the molecular property and regulatory mechanism is highly incomplete, especially at the cell type and tissue resolution. The common and tissue-specific processes are often controlled by different gene regulatory programs, which alter the expression of genes in different biological conditions [1, 2]. Comparison of gene regulatory networks in different tissues shows that the edges of the network (e.g., the links between transcription factors to target genes) have higher tissue-specificity than the nodes of the network (e.g., genes) because the links among genes are heavily regulated by their functional roles and the tissue environment [1]. In the disease environment, the topology of molecular networks helps identify novel genes and pathways associated with diseases [3].

In recent years, huge amounts of omics data from different human tissues and organs have been accumulated [4-7] and many methods have been developed to decode the dynamic regulatory links among genes. Network embeddings hold substantial promise for analyzing gene regulatory programs under various conditions. For example, the Set2Gaussian [8] method identified the gene set embeddings based on the topology of the protein-protein interaction (PPI) network and used the resultant embeddings for tumor stratification and clinical prognosis. Methods such as SAUCIE [9], scVI [10], LDVAE [11], and scGNN [12] used deep neural networks to aggregate and represent cell type-specific gene regulatory signals, enabling highly accurate downstream single-cell transcriptome analysis. Additionally, scVAE [13], scETM [14], siVAE [15], and SIMBA [16] demonstrated the broad potential of learning cell and gene embeddings simultaneously for studying cell heterogeneity, identifying gene expression features,

and integrating omics data. Furthermore, scapGNN [17] inferred stable gene-cell association networks from sparse single-cell profile data.

Deep learning approaches have proved advantageous in many human genetics and genomics studies. Variational graph autoencoder (VGAE) [21] is a type of Graph Neural Network [22, 23] used for unsupervised learning in tasks like link prediction. VGAE generally consists of encoders and decoders. The encoder compresses the input matrix to generate a latent representation through a multilayer graph convolutional network (GCN) [22, 24]. The decoder performs reconstruction of the original graph from the low-dimensional vector by calculating the connections among the nodes. VGAE achieves dimensionality reduction of graph data by maximizing the similarity of the generated graph to the original graph and minimizing the Kullback-Leibler (KL) divergence between the model-derived approximation and the true posterior distribution.

In this work, we developed a VGAE-based approach, namely scGraph2Vec, to generate highly informative and low-dimensional gene embeddings. The method extends the VGAE framework [21, 25] and integrates single-cell datasets and gene-gene interaction networks. We demonstrated that the resultant gene embeddings recapitulate high-dimensional biological information derived from the structures of gene-gene interaction networks and gene expression patterns across cells. These gene embeddings help us to understand the functional modules of genes, elucidate the influence of regulatory genes on biological processes in specific tissue environments, and infer more disease-related genes to explain disease risk. In addition, compared with nine existing embedding tools, scGraph2Vec achieved promising performance in gene

embedding and cluster prediction on the benchmark scRNA-seq datasets. In summary, scGraph2Vec can be used on a wide range of gene networks and single-cell datasets for different biological problems.

METHODS

Data collection and preprocessing

We collected the scRNA-seq data from six healthy human tissues [26], which are brain [27], heart [28], kidney [29], liver [30], lung [31], and peripheral blood mononuclear cells (PBMC) [32] (Supplementary Table S1). We also obtained paired scATAC-seq and scRNA-seq data from four mid-gestation human cerebral cortex samples (GEO accession ID: GSE162170) [33]. The LUAD tumor tissues and their distal non-malignant lung tissues were downloaded from EBI ArrayExpress (accession ID: E-MTAB-6149 and E-MTAB-6653) [26]. For each dataset, we filtered for genes with a non-zero expression value in at least 3 cells and for cells with at least 200 expressed genes. The originally downloaded count data were transformed to log (counts per million + 1) values and scaled by all cells. The interaction network was downloaded from the BioGRID database (release v.4.4.210) [34], which contained 977,356 interactions among 19,752 genes. As a comparison, we also constructed an interaction network based on the PathwayCommons database (v13, http://www.pathwaycommons.org/archives/PC2/v13/PathwayCommons13.All.hgnc.txt.gz) [35], which contained 1,874,081 interactions among 19572 genes.

scGraph2Vec model design

scGraph2Vec takes two input matrices: the adjacency matrix A from the gene-gene interaction network and the node feature matrix X from the single-cell gene profile. We first refine A by including a node community matrix and generate an enhanced adjacency matrix A_n. scGraph2Vec directs A_n and X to go through a multi-layer GCN to generate the low-dimensional vectors Z and reconstructs the graph structure of the network through the decoder process.

Specifically, we first calculate the primary assignment of node communities by using the Louvain greedy algorithm [36] based on the interaction network. Suppose M represents the membership matrix, $M \in \{0,1\}^{n \times k}$, n is the total number of nodes and k is the total number of communities or sets. Each element in M indicates whether a node i (i = 1, …, n) belongs to set j (j = 1,…, k), i.e., $M_{ij} = 1$ if $i \in C_j$ and 0 otherwise. Subsequently, a node community matrix is calculated as below:

$$A_c = MM^T - I_n \tag{1}$$

where I_n is the identity matrix. Then we calculate a new adjacency matrix A_n by:

$$A_n = A + \lambda A_c \tag{2}$$

where the hyperparameter $\lambda > 0$ is introduced to balance the contribution of the original adjacency matrix A and the derived community matrix A_c. To alleviate the computational cost, we require that in each community C_k, each node $i \in C_k$ only connects to a pre-defined number (denoted by s) of nodes in C_k, instead of connecting to all nodes in C_k. The number of nodes, s, is thus a hyperparameter that can be used to ensure the sparsity of A_n.

The encoder includes multiple convolutional layers. We design two alternative structures:

one with three layers (256-64-16) and the other with two layers (64-16). The encoder process generates the latent embedding matrix *Z*:

$$Z = GCN(A_n, X) \tag{3}$$

The decoder process reconstructs the connectivity structure of the network using *Z*, which relies on the inner product decoder followed by the non-linear process using the Sigmoid activation function:

$$\hat{A} = \sigma(ZZ^T) \tag{4}$$

where $\sigma(x) = \frac{1}{1+e^{-x}}$.

The standard optimization strategy for reconstruction typically uses the evidence lower bound (ELBO) loss L_{VGAE} to assess the similarity between the initial and the reconstructed graph structure:

$$L_{VGAE} = \mathrm{E}_{q(Z|A,X)}[\log p(A|Z,X)] - KL(q(Z|A,X)\|p(Z)) \tag{5}$$

where $KL(q(Z|A,X)\|p(Z))$ is the Kullback-Leibler divergence between $q(\cdot)$ and $p(\cdot)$. More description of L_{VGAE} can refer to the original VGAE article [21]. In scGraph2Vec, we include a complemental loss inspired by modularity for community detection. Modularity is designed to measure the strength of community structure in networks by comparing the density of connections inside and outside communities [37, 38]. To capture the global community structure, we soften the calculation of specific communities in the traditional modular formula and add a global regularizer [25]:

$$L_M = \frac{\beta}{2m}\sum_{i,j=1}^{n}\left(A_{ij} - \frac{d_i d_j}{2m}\right)e^{-\gamma\|z_i - z_j\|_2^2} \tag{6}$$

Here, the L2 distance $\|z_i - z_j\|_2^2$ is the soft counterpart of modularity which replaces the original community indicator $\delta(\mathrm{i},\mathrm{j}) \in \{0,1\}$, where $\delta(\mathrm{i},\mathrm{j}) = 1$ if nodes *i* and *j* belong to the same community and 0 otherwise. The $d_i = \sum_{j=1}^{n} A_{i,j}$ (*n* is the total number of nodes) is the degree for the ith node and $2m$ is the sum of the degrees of all nodes. Therefore, the loss function does not involve the "exact" modularity and is independent of the community information associated with nodes. The hyperparameter $\beta > 0$ balances the relative importance of the global community structure and the pairwise node connectivity. Overall, the loss function used in scGraph2Vec is: $L = L_{VGAE} + L_M$. Our optimization goal is to maximize the graph similarity and modularity score to obtain gene embeddings.

Training details

scGraph2Vec was implemented using TensorFlow (v.1.15.0). The genes in the feature matrix were adjusted to be consistent with the nodes of the interaction network. We deleted the genes that were not annotated in the interaction network and used 0 to fill in the node features that lacked feature values. The positive edges (i.e., the element values of the matrix being 1) of the initial adjacency matrix were split by 90%, 5%, and 5% respectively and the same number of negative edges (i.e., the element values of the matrix being 0) were randomly selected to form the training set, validation set, and test set, respectively. In the subsequent model training, we selected a three-layer neural network (256-64-16) encoder. The Adam optimizer strategy and a

learning rate = 1×10^{-4} were adopted. A maximum of 600 iterations and early stopping with patience = 50 was used. After a hyperparameter sweep, we recommended using $\beta = 10, \lambda = 1, \gamma = 0.1, s = 10$ as the hyperparameter combination. The model training was repeated 10 times in each tissue.

Output organization

The 16-dimensional gene embedding output by scGraph2Vec was reduced to a two-dimensional vector for visualization purposes by using *t*-Distributed Stochastic Neighbor Embedding (t-SNE) [39]. Then the hierarchical clustering method was used to identify gene modules. The total number of modules was determined according to the silhouette coefficient. We restricted each gene module to contain more than 10 genes. In each replication, the output with the maximum silhouette coefficient was selected.

Benchmark with known embedding methods

We adopt a unified benchmark framework to compare scGraph2Vec with other gene embedding calculation methods, including scVI [10], LDVAE [11], siVAE [15], scVAE [13], scGNN [12], scapGNN [17], scETM [14], SAUCIE [9], and SIMBA [16]. We used each method to extract the embeddings of all genes in the Brain and PBMC datasets and reduced them to two dimensions for visualization through t-SNE. Hierarchical clustering was utilized to identify gene modules. The effectiveness of various methods in distinguishing gene modules is assessed using the silhouette coefficient. A higher silhouette coefficient indicates better separation of distinct

modules. Considering the sparsity of single-cell data, all models assumed that data followed a zero-inflated negative binomial distribution. For the models within the unfixed VAE framework (including scVAE, siVAE, scVI, and LDVAE), we configured their structures to match that of scGraph2Vec, including the same encoder-decoder layer numbers and dimensions (256-64-16). Optimization for all models involved scanning hyperparameters without recommended values, adjusting the learning rate (1×10^{-3}, 1×10^{-4}, 1×10^{-5}) and the number of epochs (50, 100, 200, 400, 600), while other parameters were set to their defaults. Specifically, for SAUCIE, we tested parameter combinations that influenced clustering results: lambda_c (0.1, 0.2, 0.3) and lambda_d (0.5, 0.7, 0.9), and directly used the gene embeddings and clusters provided by SAUCIE. We modified the 'ConNetGNN' function of scapGNN to output gene embeddings learned from the hidden layer. For scETM, we followed the recommendation of training at least 6,000 epochs, and testing models at 6,000, 9,000, and 12,000 epochs. scGNN extracted embeddings from all genes and tested both models obtained with or without the Left Truncated Mixture Gaussian (LTMG). The final selection of the optimal model parameters was based on the convergence of the loss function.

Estimating biological implications of gene modules

We explored the biological implications of modules based on topology, housekeeping genes, and expression patterns. Firstly, we examined whether gene modules could reflect highly clustered gene sets in gene-gene interaction networks. Specifically, for each module, we divided all connections in the BioGRID network into intra-cluster and out-of-cluster connections. For

each gene, we calculated two closeness centrality values, i.e., the internal closeness centrality based on the intra-cluster connections and the external closeness centrality based on the out-of-cluster connections. Then for each module, we averaged the closeness centrality of internal connections among its genes to determine internal closeness centrality and did the same for external connections to establish external closeness centrality. Finally, we compared the difference in internal closeness centrality and external closeness centrality for all modules using the Wilcoxon rank-sum test.

A total of 397 housekeeping genes [40] were downloaded from the MSigDB database (human gene set: HSIAO_HOUSEKEEPING_GENES) [41]. We analyzed the distribution of housekeeping genes among gene modules in different tissues.

To evaluate whether each gene module had a specific expression pattern, we used the first principal component of module gene expression in each cell type as the module score. The module score can represent the overall expression pattern of module genes.

Identification of cell-type specificity and tissue specificity

For cell-type specificity, we used the gene set enrichment analysis (GSEA) [42] to examine whether module genes were significantly enriched in up-regulated or down-regulated differentially expressed genes (DEGs) across cell types. Based on the scRNA-seq data, we conducted differential gene expression analyses for each cell type using the 'FindAllMarkers' function of the Seurat software [43]. We then ranked all genes by their average log2 (fold change), where fold change (FC) was generated by comparing the cell type in investigation to all other cell types.

The predefined gene sets were derived from gene modules identified by scGraph2Vec. We used the R package GSEABase (v.1.56.0) to conduct GSEA and enrichplot (v.1.14.2) for visualization.

For tissue specificity, we used the R package clusterProfiler [44] for Gene Ontology (GO) functional enrichment analysis. To define tissue-specific modules, we performed the hypergeometric test to examine the overlap between a module in the investigated tissue and all the other modules identified in all other tissues. Modules with a significant depleted overlap (hypergeometric test $P < 0.05$ and less than 5% overlapping genes with all other tissue modules) were considered specific to the tissue where the module was initially identified.

Identification of disease-candidate genes

We took COVID-19 and Alzheimer's Disease (AD) as examples to demonstrate the performance of scGraph2Vec on inferring disease-candidate genes (Fig. 1B). We used genome-wide association studies (GWAS) summary statistics to identify the initial candidate genes. We also collected bulk RNA-seq data from disease and healthy individuals for validation. For COVID-19, we downloaded GWAS summary statistics from Host Genetics Initiative (HGI, release 7, access date: April 8, 2022, file name: A2_ALL_eur_leave23andme), including 13,769 severe COVID-19 patients and 1,072,442 healthy individuals. We also downloaded leukocytes bulk RNA-seq data of 102 patients with COVID-19 and 26 healthy individuals [45]. For AD, we downloaded GWAS summary statistics from a study conducted using 71,880 clinically diagnosed AD or AD-by-proxy and 383,378 controls [46]. We also downloaded bulk RNA-seq data from postmortem brain tissues of 376 late-onset Alzheimer's disease patients and 173 normal samples

[47].

We first defined significant genes using the software MAGMA [48], by considering the study-wide significant level based on the Bonferroni correction. These significant genes and the other containing more than 6 genes within a default radius of 0.5 were considered as seed genes. We hypothesized that genes with similar functions were located closer to each other in the two-dimensional latent space and implemented the density-based clustering algorithm [49] to identify candidate genes based on initial seed genes. For each seed gene, we searched for those located within a radius of 0.5 of the seed gene as candidate genes. Seeds located within radius 0.5 were merged as a new seed module. For each disease, we conducted the analyses in the disease-relevant tissues. We excluded genes that had a step greater than 1 with the seed gene from the final candidate genes.

Identification of candidate tumor-driver genes

We took LUAD as an example to assess the capabilities of scGraph2Vec in identifying candidate driver genes (Fig. 1B). We downloaded scRNA-seq data from a non-small cell lung cancer (NSCLC) patient [26], including data from the tumor core sample and the distal normal tissue. The gene embeddings were generated using scGraph2Vec for tumor or normal, respectively. The seeds for our investigation were the 18 LUAD-driver genes reported in the original study [50]. Subsequently, we conducted a search for additional candidate genes within the two-dimensional latent space of tumor core and normal samples, utilizing the same algorithm and parameters employed for AD and COVID-19.

To investigate the correlation between candidate genes and LUAD, we conducted a comprehensive analysis utilizing bulk RNA-seq data from The Cancer Genome Atlas (TCGA). Using the R package TCGAbiolinks (version 2.31.2) [51], we obtained preprocessed gene expression and clinical data for 600 LUAD samples (541 LUAD and 59 normal controls). Transcript per million (TPM) normalized gene expression data was utilized for subsequent analyses, with log2 transformation applied to the (TPM + 1). DEGs were identified using the R package DESeq2 (version 1.34.0) [52] (|log2(FC)| > 0.5 and $P_{BH} < 0.05$). Subsequently, the univariate Cox proportional hazards regression analysis was applied to identify prognosis-related genes (Hazard Ratio (HR) ≠ 1, $p < 0.05$). The intersection of prognosis-related genes and DEGs yielded overlapping candidate genes for the survival analysis. Samples were categorized according to the median of log2 (TPM + 1). The Kaplan-Meier (KM) curves and the log-rank tests were implemented using the R package survival (version 3.5.1) [53].

RESULTS

Overview of scGraph2Vec

scGraph2Vec was built on a VGAE framework with extensions for the task of generating informative embeddings. It took a gene-gene interaction network and a gene-feature matrix as the input and generated low-dimension gene embeddings in the latent space (Fig. 1A). scGraph2Vec had three major improvements to the standard VGAE framework. First, instead of using the standard adjacency matrix A, we generated an enhanced adjacency matrix A_n by combining *a priori* community information and the standard adjacency matrix. We used the Louvain algorithm

[36] to construct a membership matrix of genes and used this matrix to provide prior community information. The enhanced adjacency matrix A_n thus informed the encoder with information on primary assignments for gene clusters. Second, link prediction and community detection are simultaneously implemented during the model optimization process to enhance the representation of gene communities in the embedding space. Lastly, a modularity-inspired method was implemented to optimize the loss function to reduce the impact of local pairwise connections on community structure [37, 38]. By iteratively maximizing the joint of graph likelihood and modularity scores, scGraph2Vec generated latent features representing various information of genes in a high-dimensional space.

The working network was downloaded from BioGRID [34], including 19,752 genes and 977,356 interactions. Notably, the interactions were either physical (98.3%) or genetic interactions (1.7%). Thus, it has no annotation about tissue-specificity or cell-type specificity and remains the same for all the following applications. The gene feature matrix was constructed using the scRNA-seq [54, 55] or the scATAC-seq [56, 57] data in the gene-by-cell format. We collected scRNA-seq data for 6 human tissues, which are brain [27], heart [28], kidney [29], liver [30], lung [31] and PBMC [32]. All scRNA-seq data were quality-controlled and processed following the same pipeline. On average, each tissue contained 66,395 cells (ranging from 2,638 cells in PBMC to 287,269 in heart) and 24,064 genes (ranging from 13,714 genes in PBMC to 33,694 in heart and kidney) (Supplementary Table S1). To maintain the same dimensionality of the inputs, the scRNA-seq gene expression matrix is trimmed or imputed to match the 19,752 genes available in the working network.

We carried out a hyperparameter sweep to examine the key hyperparameters of scGraph2Vec and the best hyperparameters were determined by using a combination of assessment parameters, including the silhouette coefficient, the number of clusters, and the running time. We first selected the hyperparameters $\beta = 10$, $\lambda = 1$, $\gamma = 0.1$, and $s = 10$ (pink line, Supplementary Fig. S1A). Based on these hyperparameters, we next determined the following hyperparameters for model training: learning rate = 1×10^{-4}, a three-layer GCN encoder with 256, 64, and 16 neurons, respectively, and epoch time = 600 (blue line, Supplementary Fig. S1B). For larger datasets, we recommend a fast model with a two-layer GCN encoder (64 and 16 neurons for each layer). The resulting latent feature is a 19,752 genes × 16 vectors matrix.

scGraph2Vec generates gene embeddings for gene module identification

We reduced the latent features to a two-dimensional visualization space using t-SNE [39] and identified gene modules by using the hierarchical clustering method. Due to the nature of neural network algorithms, such as random initialization, the same algorithm might generate slightly different results although each output was a faithful approximation of the input graph. Hence, we replicated the model training process 10 times for each dataset, establishing a pool for selecting the best gene modules (Supplementary Fig. S2A-C).

Next, we measured the stability of the resultant models and gene modules obtained from the 10 replications. We found that the predicted edges were highly consistent across 10 replications (69% of the BioGRID edges were identified in all 10 replicates). To compare the stability of gene clustering, we took the fifth replication as the reference because it had the

highest silhouette coefficient and then compared its resultant clusters with those from the other replications. We found that the majority of clusters could be replicated except cluster 8 and cluster 10, which were slightly mixed with other clusters (Supplementary Fig. S2C, D).

Key components of scGraph2Vec are important for gene module identification

We further investigated the importance of key components in scGraph2Vec by varying its structure through excluding the gene feature matrix, randomizing the reference network, and excluding or randomizing the primary community. In each case, we generated gene embeddings using the corresponding scGraph2Vec settings (the original design or those using alternative structures) and conducted hierarchical clustering based on their two-dimensional latent features to construct gene modules. Then, we compared the resultant gene modules to evaluate the models.

As a result, the standard output of scGraph2Vec has the best module partitioning (Fig. 2A). We found that when excluding the gene feature matrix, there were hardly any modules formed based on the resultant embeddings in the brain or any other tissues tested (Supplementary Fig. S3A, B and Fig. S4A, B). We also found that the reference network was critical to forming gene modules, which was demonstrated by either randomizing the BioGRID network [34] or using an alternative reference network such as the PathwayCommons (PC) network [35]. In the former case, when using a randomized BioGRID network, we did not find any formed module (Supplementary Fig. S3C and Fig. S4C). In the latter case, the PC network contained protein, genetics, and pathway interactions. However, when we used the PC network to generate gene embeddings, the resultant gene modules were quite difficult to identify (Supplementary Fig. S5),

likely because the complex interactions from PC provided confounding information to gene expression patterns.

Then, we evaluated the impact of the primary community on module identification. When no primary community or a disrupted primary community was used, the formation of clusters became obscure (Supplementary Fig. S3D, E and Fig. S4D, E). This suggested that the primary community of scGraph2Vec played important roles in enhancing the clustering signal, which was critical to generate clusters of genes with similar behaviors in the network.

We also compared the gene modules identified using latent features trained on scRNA-seq and scATAC-seq data from the same brain samples [33]. Notably, when using scATAC-seq data, we constructed the feature matrix based on chromatin accessibility and mapped genomic regions to genes [58]. Through t-SNE plot, most modules could be replicated across different omics (Supplementary Fig. S6). This indicated that scGraph2Vec could detect gene modules conserved in chromatin accessibility and gene expression through latent features.

Collectively, we demonstrated that the latent features extracted by scGraph2Vec could be used to effectively construct gene modules in different tissue contexts.

Benchmark of scGraph2Vec with existing methods

We benchmarked scGraph2Vec using the brain [27] and PBMC [32] datasets, along with 9 single-cell embedding methods: scVAE [13], LDVAE [11], scVI [10] and siVAE [15], scapGNN [17], scGNN [12], scETM [14], SAUCIE [9], and SIMBA [16]. We uniformly extracted latent features of all genes for each method based on the same gene expression matrix,

where the unfixed VAE variants all used the same size encoder-decoder (256-64-16) structure. Notably, all these methods were designed based on the gene expression matrix only.

Overall, the key components of scGraph2Vec make it more competitive than other embedding methods in identifying gene modules (Fig. 2B), whereas the other methods always showed ambiguous gene modules (Fig. 2C, D). Methods developed for cell embeddings, such as scVI, LDVAE, SAUCIE and scETM, generally lose effectiveness in generating gene embeddings. Among the methods equipped with gene embedding, only siVAE demonstrated comparable module division to scGraph2Vec on the PBMC dataset. On the contrary, the gene embeddings of scGraph2Vec had the highest silhouette coefficients, implying the best module partitioning (Fig. 2C, D).

scGraph2Vec generated biologically meaningful modules

We applied scGraph2Vec to generate gene embeddings for 6 representative human tissues and subsequently generated gene modules using hierarchical clustering (Supplementary Fig. S7 and Table S2). The number of gene modules in each tissue was determined by the maximum value of the silhouette coefficient. As a result, we obtained an average of 134 gene modules for each tissue, ranging from 108 to 149 (Supplementary Fig. S8). Each module contained tens to hundreds of genes, e.g., 68 to 270 genes per module for the brain (Supplementary Table S2). We next evaluated these modules for their topological characteristics, housekeeping genes, and cell-type specificity.

To test if the module genes were topologically correlated, we examined the closeness

centrality of module genes. Taking the brain tissue as an example, the internal closeness centrality was significantly higher than the external closeness centrality of the modules ($P < 2.22 \times 10^{-16}$, Fig. 3A). Thus, module genes were topologically related.

We next explored the biological implications of the modules identified in different tissues using housekeeping genes, as these genes were known to play critical functions in cells [40]. We found that housekeeping genes tended to cluster together and were enriched in a few gene modules (Fig. 3B and Supplementary Fig. S9). Such a pattern of distribution was consistently observed across all tissues but not in the modules generated using alternative scGraph2Vec structures.

To represent the expression of gene modules in different cell types, we calculated the module score for each cell using the first principal component of the expression value of module genes in each cell. We used t-SNE to compress the cells into a two-dimensional space and annotated the cells with cell types from the original study [27]. We found that different module scores showed similar trends across cell types, such as excitatory neurons always showing higher or lower expression than other cell types (Fig. 3C and Supplementary Fig. S10). This indicated that the latent embeddings extracted by scGraph2Vec were informative for gene expression patterns.

scGraph2Vec generated modules with cell-type and tissue specificity

Next, we tested if the module genes were enriched with cell-type-specific genes using GSEA. To this end, we defined cell-type specific genes as the DEGs for each cell type using the

original scRNA-seq data [27]. Taking the brain tissue as an example, we found a total of 37 modules significantly enriched with at least one cell type (Benjamini-Hochberg (BH) adjusted *P*, or $P_{BH} < 0.05$, Fig. 3D and Supplementary Table S3). For example, cluster 9 was enriched with up-regulated DEGs of the largest number of associated cell types, including excitatory neuronal (Ex) subtype 3c (Ex3c), Ex5a, Ex8, Ex3b, Ex4, Ex3e, inhibitory neuronal (In) subtype 1c (In1c), pericytes (Per), and cerebellar granule cells (Gran) (Fig. 3D-F and Supplementary Fig. S11). GO enrichment analyses [44] showed that cluster 9 was related to functions such as cytoplasmic translation ($P_{BH} = 1.02 \times 10^{-8}$), ribosome biogenesis ($P_{BH} = 8.75 \times 10^{-8}$), ribonucleoprotein complex biogenesis ($P_{BH} = 7.83 \times 10^{-5}$) and ribonucleoprotein processing ($P_{BH} = 1.68 \times 10^{-8}$) (Fig. 4A). Another example is cluster 59, which was enriched in multiple excitatory and inhibitory neurons and was particularly related to down-regulated DEGs in these cell types (Fig. 3D, E and Supplementary Fig. S11). Cluster 59 was mainly associated with functions of chromatin organization ($P_{BH} = 1.59 \times 10^{-39}$), histone modification ($P_{BH} = 3.44 \times 10^{-31}$), and histone acetylation ($P_{BH} = 6.57 \times 10^{-16}$) (Fig. 4B).

In the other five tissues, we identified 16 to 81 modules enriched in cell type DEGs of the corresponding tissue. In the lung tissue, 24 of 120 modules were significantly enriched in DEGs of 27 lung cell types, including airway smooth muscle, capillary aerocyte, alveolar fibroblast, and alveolar epithelial type 1 ($P_{BH} < 0.05$; Supplementary Fig. S12). In the heart tissue, 16 of 149 modules were associated with DEGs of 15 heart cell types, such as cytoplasmic cardiomyocyte I and II, atrial cardiomyocyte, ventricular cardiomyocyte I and II ($P_{BH} < 0.05$; Supplementary Fig. S13). In liver, 81 of 137 modules were significantly enriched in DEGs of 19 liver cell types,

including hepatocytes, central venous liver sinusoidal endothelial cells, and hepatic stellate cells (P_{BH} < 0.05; Supplementary Fig. S14). In addition, we identified a total of 143 modules in kidney, of which 72 were related to the DEGs of kidney cell types (P_{BH} < 0.05; Supplementary Fig. S15). In PBMC, 7 of 128 modules were significantly enriched in DEGs of 4 cell types, i.e., B cell, CD14+ monocytes, memory CD4+ T cell, and naïve CD4+ T cell (P_{BH} < 0.05; Supplementary Fig. S16).

We also investigated the functions of the modules (Supplementary Fig. S17). The cluster 49 of lung, which enriched in cell types of alveolar epithelial type 1, alveolar fibroblast, and airway smooth muscle, was associated with functions of chemokine-mediated signaling pathway ($P_{BH} = 1.85 \times 10^{-6}$) and negative regulation of endopeptidase activity ($P_{BH} = 2.08 \times 10^{-6}$). The cluster 80 of heart was enriched in atrial cardiomyocyte and was associated with muscle cell development ($P_{BH} = 1.68 \times 10^{-8}$) and myofibril assembly ($P_{BH} = 2.80 \times 10^{-6}$). Among the modules enriched in hepatocytes, we discovered many associations with diverse metabolic processes. For example, cluster 35 was related to sulfur compound metabolic process ($P_{BH} = 0.002$) and gluconeogenesis ($P_{BH} = 0.002$), cluster 39 was related to organic acid catabolic process ($P_{BH} = 1.31 \times 10^{-12}$) and carboxylic acid catabolic process ($P_{BH} = 8.30 \times 10^{-12}$), and cluster 40 was related to nucleotide metabolic process ($P_{BH} = 3.57 \times 10^{-7}$). Additionally, in the kidney, we found cluster 4 was associated with response to dietary excess ($P_{BH} = 0.007$) and fatty acid transport ($P_{BH} = 0.007$).

Next, we compared the cross-tissue differences in gene modules. We defined tissue-specific modules as those that had less than 5% overlapping genes with all modules identified in

the other 5 tissues. For the brain tissue, we identified 50/127 modules as brain-specific, among which the cluster 59, aforementioned as closely associated with the brain and enriched in genes of nBAF complex, was included (Fig. 4B, C). Then we searched for genes located near *SMARCE1*, a key gene of the nBAF complex, by density-based clustering method [49] in the latent feature space and included genes located within 0.5 radius of *SMARCE1* (Fig. 1B). We compared the neighbor genes found in the brain and non-brain (i.e. lung) and reconstructed the predicted gene subnetwork (Fig. 4D, E). In the brain tissue, 14 genes were found in the neighboring subnetwork of *SMARCE1* and these genes were enriched with a neuron fate commitment, medial motor column neuron differentiation, and somatic motor neuron differentiation, among others (Fig. 4D). In contrast, in the lung tissue, 20 genes were found adjacent to *SMARCE1* but they were enriched with general functions related to chromatin organization and histone H3-K9 (Fig. 4E).

Collectively, we demonstrated the high-dimensional biological information implied by gene embeddings in six representative human tissues and provided a general reference panel of tissue-related gene modules.

Applications of scGraph2Vec found more disease-associated genes

Large-scale GWAS have identified thousands of genetic associations with diseases. Disease-associated genes often interact with each other and jointly disturb multiple pathways or regulatory networks in disease tissues or cell types. Therefore, understanding how genes interact in a context is crucial to understanding the molecular mechanisms of diseases. We next explored the ability of scGraph2Vec to infer disease-associated genes by using the resultant gene

embeddings and demonstrated it in COVID-19 and Alzheimer's Disease.

Application in COVID-19. We downloaded GWAS summary statistics from HGI, which conducted the largest GWAS to date for COVID-19 susceptibility, severity, and outcomes [59]. Specifically, we selected COVID-19 severity as the phenotype for the following analyses. By using MAGMA [48], we calculated gene-based p-values and identified 60 significant genes for COVID-19 severity (Bonferroni-corrected threshold $P < 2.63 \times 10^{-6}$). To verify the associations, we defined DEGs by comparing the bulk RNA-seq data from 102 COVID-19-positive patients and 26 COVID-19-negative individuals [45] ($P_{BH} < 0.05$ and |log2(FC)>1|) following the original study [45]. Notably, for the 60 GWAS-implied genes, only six were DEGs (Fig. 5A). Next, we used the 60 genes as the seed genes and searched for their neighbor genes in the two-dimensional latent space generated using t-SNE based on the lung gene embeddings aforementioned. As a result, we obtained 356 candidate genes for COVID-19 severity. Interestingly, 28 of 356 candidate genes were validated to be DEGs in COVID-19 versus non-COVID-19. This proportion, though only marginally significant ($P = 0.067$, hypergeometric test; Fig. 5B), indicated that the gene embedding data indeed could be used to identify more disease-associated genes. The 28 genes showed distinct expression patterns in COVID-19 and non-COVID-19 samples (Fig. 5D). Importantly, many genes were identified but were missed by the original GWAS results, such as *MKI67*, *STIL*, *NUF2*, *OIP5*, *TNFRSF17*, and *CEACAM8*. On the contrary, we conducted the same analyses using the brain latent features (i.e., a disease irrelevant tissue) and found 681 candidate genes, among which 35 were significantly associated with COVID-19 but were not statistically significant ($P = 0.83$; Fig. 5C).

Functional enrichment analysis of the 356 genes showed that they were mainly enriched in GO terms related to cytokine receptor activity ($P_{BH} = 4.86 \times 10^{-6}$), C-C chemokine receptor activity ($P_{BH} = 4.86 \times 10^{-5}$), and peptidyl-lysine modification ($P_{BH} = 8.23 \times 10^{-5}$), among others (Fig. 5E left plot). We particularly examined the 8 neighbor genes of the gene *CEACAM8*. These genes were enriched in biological processes related to the glycosaminoglycan (GAG) catabolic process ($P_{BH} = 3.19 \times 10^{-4}$, Fig. 5E right plot). GAGs are receptors used by a large number of microbial pathogens to adhere to and invade cells [60]. Recent evidence had shown that the entry process of SARS-CoV-2 into host cells was mediated by the transmembrane spike (S) protein interacting with both cellular heparan sulfate GAG and angiotensin converting enzyme 2 (ACE2) [61, 62]. Thus, GAG derivatives have been promising candidates for SARS-CoV-2 antiviral therapy [63, 64].

Application in Alzheimer's Disease (AD). Using a large-scale GWAS for AD (71,880 cases and 383,378 controls) [46], we identified 65 AD-associated genes by MAGMA at the Bonferroni-corrected threshold $P < 3.77 \times 10^{-6}$. Fourteen of 65 genes were DEGs between the AD group and the control group using an independent bulk gene expression dataset ($P_{BH} < 0.05$ and |fold change| > 1.1; Fig. 6A) [65]. Using the 65 genes as seed genes and the gene embeddings generated for the brain tissue, we identified 362 candidate genes for AD. These newly identified AD-candidate genes were significantly enriched with DEGs (112/362, hypergeometric test $P = 0.011$, Fig. 6B). As a negative control, we found 317 neighbor genes near the 65 seed genes using the gene embeddings generated for an irrelevant tissue, i.e., the healthy lung tissue. However, only 78 of 317 genes were DEGs and this proportion was not statistically significant ($P = 0.68$,

hypergeometric test; Fig. 6C). We found a causal gene *SERPINA3* that had been verified to be associated with AD but had never been detected by GWAS [66]. These candidate genes identified by latent features can well distinguish AD patients from healthy controls (Fig. 6D). Functional enrichment analysis of these genes indicated interesting GO terms that might be important pathogenic causes for AD, such as the mitochondrial electron transport chain ($P_{BH} = 2.75 \times 10^{-8}$; Fig. 6E left plot) [67]. Furthermore, the newly identified AD-candidate genes can help to better explain the molecular mechanisms of potential targets. For example, we found that an AD risk gene, *APOC1*, and its 8 neighbor genes (such as *ADORA2A*, *CDH2*, and *SLC28A2*) were enriched in functions related to negative regulation of hydrolase activity ($P_{BH} = 0.01$), regulation of synaptic transmission ($P_{BH} = 0.01$), glutamatergic ($P_{BH} = 0.01$), negative regulation of phosphatidylcholine catabolic process ($P_{BH} = 0.01$) and so on (Fig. 6E right plot).

Applications of scGraph2Vec identified candidate driver genes in LUAD

We also demonstrated scGraph2Vec in identifying candidate driver-like genes in cancer. Using scRNA-seq data LUAD patients [26], we generated latent embeddings for tumor and normal lung tissues, respectively, and identified gene clusters in each condition (Supplementary Fig. S18A, B and Table S2). Notably, in the two-dimensional latent space, we observed a partial enrichment of housekeeping genes (Supplementary Fig. S18C, D). By comparing the overlapping genes in the clusters from either normal or tumor, we identified 41 clusters specifically enriched in tumor (Fig. 7A). These tumor-specific clusters were enriched in functions related to oxidative phosphorylation ($P_{BH} = 1.13 \times 10^{-87}$), double-strand break repair ($P_{BH} = 1.45 \times 10^{-7}$), cytokine-

mediated signaling pathway ($P_{BH} = 4.14 \times 10^{-6}$), cell-cell adhesion via plasma-membrane adhesion molecules ($P_{BH} = 0.005$), and stem cell population maintenance ($P_{BH} = 0.004$) (Fig. 7B). Then, we used the 18 LUAD-driver genes reported by TCGA as seeds (*ARID1A*, *BRAF*, *CDKN2A*, *EGFR*, *KEAP1*, *KRAS*, *MET*, *MGA*, *NF1*, *PIK3CA*, *RB1*, *RBM10*, *RIT10*, *SETD2*, *SMARCA4*, *STK11*, *TP53*, and *U2AF1*) [50], and identified 251 neighbor genes in the embedding space of tumor and 505 neighbor genes in normal. Notably, we observed that the enrichment of these neighbor genes aligned with the tissue context of the respective samples. For example, *TP53* neighboring genes in normal are enriched for functions like DNA conformation change ($P_{BH} = 0.005$), protein-DNA complex assembly ($P_{BH} = 0.005$) and epidermal cell division ($P_{BH} = 0.005$). However, the genes adjacent to *TP53* in tumor are enriched for functions related to tumor development, such as negative regulation of DNA replication ($P_{BH} = 0.02$), 7-methylguanosine cap hypermethylation ($P_{BH} = 0.02$), negative regulation of pentose-phosphate shunt ($P_{BH} = 0.02$), mitotic DNA damage checkpoint signaling ($P_{BH} = 0.03$) (Fig. 7C).

To validate the connection between neighbor genes and LUAD, we downloaded bulk RNA-seq data of LUAD samples from TCGA including expression data for 238 out of the 251 neighbor genes identified in the tumor. Among all 238 neighbor genes, 109 were also DEGs ($|\log2(FC)| > 0.5$ and $P_{BH} < 0.05$). Specifically, for *TP53*, 9 out of 10 neighbor genes were DEGs (Fig. 7D, Wilcoxon rank-sum test, $p < 0.05$). Subsequently, using the univariate Cox proportional hazards model, we discovered 44 prognosis-related genes (Hazard Ratio (HR) $\neq$ 1, $p < 0.05$), among which 27 genes were also DEGs (Fig. 7E). Then we conducted survival analysis using each of the 27 genes to stratify samples by the median of log2 (TPM + 1) as the cutoff value.

High expression of 13 genes and low expression of 5 genes were associated with poor prognosis in LUAD patients (Fig. 7F, G and Supplementary Fig. S19). Among these, several were recognized as LUAD-related genes, including *ABCE1* [68], *CCNE1* [69], *CHCHD2* [70], *ERG* [71], *HOXA1* [72], *KRAS* [73], *KRT17* [74], *LHX2* [75], *LTB* [76] and *SHMT2* [77], among others.

Taken together, our results reveal that the low-dimensional gene feature vectors generated by scGraph2Vec can efficiently represent the high-dimensional characteristics of genes. These gene embeddings can be used to investigate gene interaction patterns in specific tissues and discover more disease candidate genes, which can be subsequently used to infer the causes and potential targets for diseases.

DISCUSSION

We developed scGraph2Vec based on VGAE to represent tissue-specific gene expression characteristics by integrating high-dimensional heterogeneous information of gene interaction networks and single-cell gene matrices in specific tissues. scGraph2Vec effectively identified gene modules in specific tissue environments by learning the coordinated expression patterns of interacting genes in similar cells, providing new insights into the regulatory functions of genes in tissues. We applied this method to disease-related gene inference. Using GWAS-identified genes as seeds, we found more disease-associated genes and provided tissue-specific etiological explanations for these genes. Because gene latent features accurately measure and integrate molecular patterns at different levels, they provide more complete associations between genes

and phenotypes in tissues, effectively complementing population genetics as a tool in disease research.

The precise description of genes in complex multicellular organisms depends on the interaction between genes and gene products in response to the cellular environment. A large amount of public single-cell omics sequencing data has been accumulated, providing an unprecedented opportunity to identify the cellular processes of genes under different cell lineages and environmental changes. However, it is challenging to accurately infer the regulatory programs of genes in cell lineages from these data. One is that most of the data have heterogeneous structures and are generated from different experimental designs and technical platforms. In addition, experimental techniques for generating high-throughput tissue-specific gene interactions are currently largely unfeasible, especially for cell line samples that are not readily available. Therefore, there is an urgent need to develop comprehensive computational methods to integrate multi-level genome data and provide systematic explanations for tissue-specific regulatory networks. Our approach uses genetic and physical gene interactions as the reference network and can be extended to other forms of gene networks, such as transcription factor-target gene interactions, metabolic pathways, etc. This extensibility is especially important in an era when there is already a large amount of publicly available gene networks and single-cell omics data. Comprehensive gene latent feature profiling of human tissues can provide the necessary background panel for gene regulation and will contribute to genome-wide disease association studies represented by GWAS.

One limitation of scGraph2Vec is that it relies on the data quality of the gene feature

matrix that provides tissue-specific information. In this work, we used publicly available human tissue scRNA-seq data. The scRNA-seq data is noisy and incomplete, especially since it comes from specific sampling sites and different experimental designs. Graph embedding can reduce some technical noises, but cannot impute the unknown tissue-specific information. In the future, strong generative learning algorithms may help. Second, the boundaries of gene modules are difficult to determine. The regulation pattern of gene sets in a given tissue is ambiguous, although we assume that genetically and physically interacting gene sets are more likely to regulate each other. In the feature space extracted by scGraph2Vec, the physical distance between genes does not represent the distance of the regulatory relationship between genes, because it often depends on the gene interaction network used in model training. Therefore, to increase the interpretability of our method, we recommend the use of a single relationship gene interaction network.

AVAILABILITY OF SOURCE CODE AND REQUIREMENTS

Project name: scGraph2Vec

Project home page: https://github.com/LPH-BIG/scGraph2Vec

Operating system(s): Platform independent

Programming language: Python

Other requirements: Python 3.7 or higher, TensorFlow 1.15.0

License: MIT License

RRID: SCR_025322

DATA AVAILABILITY

All data supporting this study are publicly available from the references cited in the paper. The scRNA-seq and RNA data can be downloaded following the references in Additional file 2: Supplementary Table.1.

ADDITIONAL FILES

Supplementary Table.1: Statistics of the datasets used.

Supplementary Table.2: Gene modules identified by scGraph2Vec in 6 representative human tissues and tumor/normal tissues of LUAD.

Supplementary Table.3: The results of applying GSEA to identify potential enrichment of gene modules among brain tissue cell type-specific genes.

Supplementary Fig.1: The hyperparameter sweep result.

Supplementary Fig.2: The evaluation of model stability.

Supplementary Fig.3: Performance evaluation of the key components in scGraph2Vec by varying its structure using brain dataset.

Supplementary Fig.4: Performance evaluation of the key components in scGraph2Vec by varying its structure using PBMC dataset.

Supplementary Fig.5: Evaluate model performance using gene networks from Pathway Commons.

Supplementary Fig.6: Gene modules cross-validated using scRNA-seq data and scATAC-seq from the same brain samples.

Supplementary Fig.7: Gene modules in 6 human tissues.

Supplementary Fig.8: The optimal clustering was selected by silhouette coefficient in 10 replications.

Supplementary Fig.9: The distribution of housekeeping genes in latent features from 6 human tissues.

Supplementary Fig.10: Module score in cells from cluster 41 to cluster 60.

Supplementary Fig.11: GSEA analysis between the gene modules (cluster 9 and cluster 59) and cell types in the brain dataset.

Supplementary Fig.12: GSEA for lung modules.

Supplementary Fig.13: GSEA for heart modules.

Supplementary Fig.14: GSEA for liver modules.

Supplementary Fig.15: GSEA of kidney modules.

Supplementary Fig.16: GSEA of PBMC modules.

Supplementary Fig.17: GO enrichment analysis for module genes.

Supplementary Fig.18: Gene modules in tumor tissue (A) and normal tissue(B). Housekeeping genes in latent features from tumor tissue (C) and normal tissue (D).

Supplementary Fig.19: Kaplan–Meier survival analysis of 16 out of 18 overlap candidate genes.

ABBREVIATIONS

AD: Alzheimer's disease; DEG: differentially expressed gene; Ex: excitatory neuronal; GCN: graph convolutional network; Gran: cerebellar granule cells; GSEA: gene set enrichment

analysis; GO: Gene Ontology; GWAS: genome-wide association studies; HR: hazard ratio; In: inhibitory neuronal; LUAD: lung adenocarcinoma; NSCLC: non-small cell lung cancer; PBMC: peripheral blood mononuclear cells; PCA: principal component analysis; Per: pericytes; TCGA: The Cancer Genome Atlas; t-SNE: t-Distributed Stochastic Neighbor Embedding; TPM: transcript per million; VGAE: variational graph autoencoder.

FUNDING STATEMENT

This research was funded by the Strategic Priority Research Program of the Chinese Academy of Sciences [XDB38010400], and the National Natural Science Foundation of China [32270706].

ACKNOWLEDGMENTS

The authors would like to thank the members of the Laboratory for Precision Health for their valuable discussion.

AUTHOR CONTRIBUTIONS

P.J. conceived the project. S.L. developed the method and analyzed the results. P.J. supervised the study. S.L. and P.J. wrote the paper.

DECLARATION OF INTEREST

The authors declare no competing interests.

REFERENCES

1. Sonawane AR, Platig J, Fagny M, Chen CY, Paulson JN, Lopes-Ramos CM, et al. Understanding Tissue-Specific Gene Regulation. Cell Rep. 2017;21 4:1077-88. doi:10.1016/j.celrep.2017.10.001.
2. Pierson E, Koller D, Battle A, Mostafavi S, Ardlie KG, Getz G, et al. Sharing and Specificity of Co-expression Networks across 35 Human Tissues. PLoS Comput Biol. 2015;11 5:e1004220. doi:10.1371/journal.pcbi.1004220.
3. Huang JK, Carlin DE, Yu MK, Zhang W, Kreisberg JF, Tamayo P, et al. Systematic Evaluation of Molecular Networks for Discovery of Disease Genes. Cell Syst. 2018;6 4:484-95.e5. doi:10.1016/j.cels.2018.03.001.
4. Lonsdale J, Thomas J, Salvatore M, Phillips R, Lo E, Shad S, et al. The genotype-tissue expression (GTEx) project. Nat Genet. 2013;45 6:580-5.
5. Lizio M, Abugessaisa I, Noguchi S, Kondo A, Hasegawa A, Hon CC, et al. Update of the FANTOM web resource: expansion to provide additional transcriptome atlases. Nucleic Acids Res. 2019;47 D1:D752-d8. doi:10.1093/nar/gky1099.
6. The human body at cellular resolution: the NIH Human Biomolecular Atlas Program. Nature. 2019;574 7777:187-92. doi:10.1038/s41586-019-1629-x.
7. Regev A, Teichmann SA, Lander ES, Amit I, Benoist C, Birney E, et al. The Human Cell Atlas. Elife. 2017;6 doi:10.7554/eLife.27041.
8. Wang S, Flynn ER and Altman RB. Gaussian Embedding for Large-scale Gene Set Analysis. Nat Mach Intell. 2020;2 7:387-95. doi:10.1038/s42256-020-0193-2.
9. Amodio M, van Dijk D, Srinivasan K, Chen WS, Mohsen H, Moon KR, et al. Exploring single-cell data with deep multitasking neural networks. Nature methods. 2019;16 11:1139-45. doi:10.1038/s41592-019-0576-7.
10. Lopez R, Regier J, Cole MB, Jordan MI and Yosef N. Deep generative modeling for single-cell transcriptomics. Nature methods. 2018;15 12:1053-8. doi:10.1038/s41592-018-0229-2.
11. Svensson V, Gayoso A, Yosef N and Pachter L. Interpretable factor models of single-cell RNA-seq via variational autoencoders. Bioinformatics (Oxford, England). 2020;36 11:3418-21. doi:10.1093/bioinformatics/btaa169.
12. Wang J, Ma A, Chang Y, Gong J, Jiang Y, Qi R, et al. scGNN is a novel graph neural network framework for single-cell RNA-Seq analyses. Nature communications. 2021;12 1:1882. doi:10.1038/s41467-021-22197-x.
13. Grønbech CH, Vording MF, Timshel PN, Sønderby CK, Pers TH and Winther O. scVAE: variational auto-encoders for single-cell gene expression data. Bioinformatics (Oxford, England). 2020;36 16:4415-22. doi:10.1093/bioinformatics/btaa293.
14. Zhao Y, Cai H, Zhang Z, Tang J and Li Y. Learning interpretable cellular and gene signature embeddings from single-cell transcriptomic data. Nature communications. 2021;12 1:5261.

doi:10.1038/s41467-021-25534-2.
15. Choi Y, Li R and Quon G. siVAE: interpretable deep generative models for single-cell transcriptomes. Genome Biol. 2023;24 1:29. doi:10.1186/s13059-023-02850-y.
16. Chen H, Ryu J, Vinyard ME, Lerer A and Pinello L. SIMBA: single-cell embedding along with features. Nature methods. 2023; doi:10.1038/s41592-023-01899-8.
17. Han X, Wang B, Situ C, Qi Y, Zhu H, Li Y, et al. scapGNN: A graph neural network-based framework for active pathway and gene module inference from single-cell multi-omics data. PLoS biology. 2023;21 11:e3002369. doi:10.1371/journal.pbio.3002369.
18. Zhao W, Gu X, Chen S, Wu J and Zhou Z. MODIG: integrating multi-omics and multi-dimensional gene network for cancer driver gene identification based on graph attention network model. Bioinformatics (Oxford, England). 2022;38 21:4901-7. doi:10.1093/bioinformatics/btac622.
19. Yang X, Wang W, Ma JL, Qiu YL, Lu K, Cao DS, et al. BioNet: a large-scale and heterogeneous biological network model for interaction prediction with graph convolution. Briefings in bioinformatics. 2022;23 1 doi:10.1093/bib/bbab491.
20. Bernett J, Krupke D, Sadegh S, Baumbach J, Fekete SP, Kacprowski T, et al. Robust disease module mining via enumeration of diverse prize-collecting Steiner trees. Bioinformatics (Oxford, England). 2022;38 6:1600-6. doi:10.1093/bioinformatics/btab876.
21. Kipf TN and Welling MJapa. Variational graph auto-encoders. 2016.
22. Scarselli F, Gori M, Tsoi AC, Hagenbuchner M and Monfardini G. The graph neural network model. IEEE Trans Neural Netw. 2009;20 1:61-80. doi:10.1109/tnn.2008.2005605.
23. Micheli A. Neural network for graphs: a contextual constructive approach. IEEE Trans Neural Netw. 2009;20 3:498-511. doi:10.1109/tnn.2008.2010350.
24. Kipf TN and Welling MJapa. Semi-supervised classification with graph convolutional networks. 2016.
25. Salha-Galvan G, Lutzeyer JF, Dasoulas G, Hennequin R and Vazirgiannis M. Modularity-aware graph autoencoders for joint community detection and link prediction. Neural Netw. 2022;153:474-95. doi:10.1016/j.neunet.2022.06.021.
26. Lambrechts D, Wauters E, Boeckx B, Aibar S, Nittner D, Burton O, et al. Phenotype molding of stromal cells in the lung tumor microenvironment. Nat Med. 2018;24 8:1277-89. doi:10.1038/s41591-018-0096-5.
27. Lake BB, Chen S, Sos BC, Fan J, Kaeser GE, Yung YC, et al. Integrative single-cell analysis of transcriptional and epigenetic states in the human adult brain. Nat Biotechnol. 2018;36 1:70-80. doi:10.1038/nbt.4038.
28. Tucker NR, Chaffin M, Fleming SJ, Hall AW, Parsons VA, Bedi KC, Jr., et al. Transcriptional and Cellular Diversity of the Human Heart. Circulation. 2020;142 5:466-82. doi:10.1161/circulationaha.119.045401.
29. Stewart BJ, Ferdinand JR, Young MD, Mitchell TJ, Loudon KW, Riding AM, et al. Spatiotemporal immune zonation of the human kidney. Science. 2019;365 6460:1461-6. doi:10.1126/science.aat5031.
30. MacParland SA, Liu JC, Ma XZ, Innes BT, Bartczak AM, Gage BK, et al. Single cell RNA

sequencing of human liver reveals distinct intrahepatic macrophage populations. Nature communications. 2018;9 1:4383. doi:10.1038/s41467-018-06318-7.
31. Travaglini KJ, Nabhan AN, Penland L, Sinha R, Gillich A, Sit RV, et al. A molecular cell atlas of the human lung from single-cell RNA sequencing. Nature. 2020;587 7835:619-25. doi:10.1038/s41586-020-2922-4.
32. Zheng GX, Terry JM, Belgrader P, Ryvkin P, Bent ZW, Wilson R, et al. Massively parallel digital transcriptional profiling of single cells. Nature communications. 2017;8:14049. doi:10.1038/ncomms14049.
33. Trevino AE, Müller F, Andersen J, Sundaram L, Kathiria A, Shcherbina A, et al. Chromatin and gene-regulatory dynamics of the developing human cerebral cortex at single-cell resolution. Cell. 2021;184 19:5053-69.e23. doi:10.1016/j.cell.2021.07.039.
34. Oughtred R, Rust J, Chang C, Breitkreutz BJ, Stark C, Willems A, et al. The BioGRID database: A comprehensive biomedical resource of curated protein, genetic, and chemical interactions. Protein Sci. 2021;30 1:187-200. doi:10.1002/pro.3978.
35. Rodchenkov I, Babur O, Luna A, Aksoy BA, Wong JV, Fong D, et al. Pathway Commons 2019 Update: integration, analysis and exploration of pathway data. Nucleic Acids Res. 2020;48 D1:D489-d97. doi:10.1093/nar/gkz946.
36. Blondel VD, Guillaume J-L, Lambiotte R, Lefebvre EJJosmt and experiment. Fast unfolding of communities in large networks. 2008;2008 10:P10008.
37. Lobov I and Ivanov SJapa. Unsupervised community detection with modularity-based attention model. 2019.
38. Wang X, Cui P, Wang J, Pei J, Zhu W and Yang S. Community preserving network embedding. In: *Proceedings of the AAAI conference on artificial intelligence* 2017.
39. Van der Maaten L and Hinton GJJomlr. Visualizing data using t-SNE. 2008;9 11.
40. Hsiao LL, Dangond F, Yoshida T, Hong R, Jensen RV, Misra J, et al. A compendium of gene expression in normal human tissues. Physiol Genomics. 2001;7 2:97-104. doi:10.1152/physiolgenomics.00040.2001.
41. Liberzon A, Birger C, Thorvaldsdóttir H, Ghandi M, Mesirov JP and Tamayo P. The Molecular Signatures Database (MSigDB) hallmark gene set collection. Cell Syst. 2015;1 6:417-25. doi:10.1016/j.cels.2015.12.004.
42. Subramanian A, Tamayo P, Mootha VK, Mukherjee S, Ebert BL, Gillette MA, et al. Gene set enrichment analysis: a knowledge-based approach for interpreting genome-wide expression profiles. Proc Natl Acad Sci U S A. 2005;102 43:15545-50. doi:10.1073/pnas.0506580102.
43. Hao Y, Hao S, Andersen-Nissen E, Mauck WM, 3rd, Zheng S, Butler A, et al. Integrated analysis of multimodal single-cell data. Cell. 2021;184 13:3573-87.e29. doi:10.1016/j.cell.2021.04.048.
44. Wu T, Hu E, Xu S, Chen M, Guo P, Dai Z, et al. clusterProfiler 4.0: A universal enrichment tool for interpreting omics data. Innovation (Camb). 2021;2 3:100141. doi:10.1016/j.xinn.2021.100141.
45. Overmyer KA, Shishkova E, Miller IJ, Balnis J, Bernstein MN, Peters-Clarke TM, et al.

Large-Scale Multi-omic Analysis of COVID-19 Severity. Cell Syst. 2021;12 1:23-40.e7. doi:10.1016/j.cels.2020.10.003.

46. Jansen IE, Savage JE, Watanabe K, Bryois J, Williams DM, Steinberg S, et al. Genome-wide meta-analysis identifies new loci and functional pathways influencing Alzheimer's disease risk. Nat Genet. 2019;51 3:404-13. doi:10.1038/s41588-018-0311-9.

47. Zhang B, Gaiteri C, Bodea LG, Wang Z, McElwee J, Podtelezhnikov AA, et al. Integrated systems approach identifies genetic nodes and networks in late-onset Alzheimer's disease. Cell. 2013;153 3:707-20. doi:10.1016/j.cell.2013.03.030.

48. de Leeuw CA, Mooij JM, Heskes T and Posthuma D. MAGMA: generalized gene-set analysis of GWAS data. PLoS Comput Biol. 2015;11 4:e1004219. doi:10.1371/journal.pcbi.1004219.

49. Ester M, Kriegel H-P, Sander J and Xu X. Density-based spatial clustering of applications with noise. In: *Int Conf knowledge discovery and data mining* 1996.

50. Comprehensive molecular profiling of lung adenocarcinoma. Nature. 2014;511 7511:543-50. doi:10.1038/nature13385.

51. Colaprico A, Silva TC, Olsen C, Garofano L, Cava C, Garolini D, et al. TCGAbiolinks: an R/Bioconductor package for integrative analysis of TCGA data. Nucleic Acids Res. 2016;44 8:e71. doi:10.1093/nar/gkv1507.

52. Love MI, Huber W and Anders S. Moderated estimation of fold change and dispersion for RNA-seq data with DESeq2. Genome Biol. 2014;15 12:550. doi:10.1186/s13059-014-0550-8.

53. Lin H and Zelterman D. Modeling survival data: extending the Cox model. Taylor & Francis, 2002.

54. Fan HC, Fu GK and Fodor SP. Expression profiling. Combinatorial labeling of single cells for gene expression cytometry. Science. 2015;347 6222:1258367. doi:10.1126/science.1258367.

55. Klein AM, Mazutis L, Akartuna I, Tallapragada N, Veres A, Li V, et al. Droplet barcoding for single-cell transcriptomics applied to embryonic stem cells. Cell. 2015;161 5:1187-201. doi:10.1016/j.cell.2015.04.044.

56. Buenrostro JD, Wu B, Litzenburger UM, Ruff D, Gonzales ML, Snyder MP, et al. Single-cell chromatin accessibility reveals principles of regulatory variation. Nature. 2015;523 7561:486-90. doi:10.1038/nature14590.

57. Satpathy AT, Granja JM, Yost KE, Qi Y, Meschi F, McDermott GP, et al. Massively parallel single-cell chromatin landscapes of human immune cell development and intratumoral T cell exhaustion. Nat Biotechnol. 2019;37 8:925-36. doi:10.1038/s41587-019-0206-z.

58. Baek S and Lee I. Single-cell ATAC sequencing analysis: From data preprocessing to hypothesis generation. Comput Struct Biotechnol J. 2020;18:1429-39. doi:10.1016/j.csbj.2020.06.012.

59. The COVID-19 Host Genetics Initiative, a global initiative to elucidate the role of host genetic factors in susceptibility and severity of the SARS-CoV-2 virus pandemic. Eur J Hum Genet. 2020;28 6:715-8. doi:10.1038/s41431-020-0636-6.

60. Aquino RS and Park PW. Glycosaminoglycans and infection. Front Biosci (Landmark Ed). 2016;21 6:1260-77. doi:10.2741/4455.
61. Clausen TM, Sandoval DR, Spliid CB, Pihl J, Perrett HR, Painter CD, et al. SARS-CoV-2 Infection Depends on Cellular Heparan Sulfate and ACE2. Cell. 2020;183 4:1043-57.e15. doi:10.1016/j.cell.2020.09.033.
62. Kim SY, Jin W, Sood A, Montgomery DW, Grant OC, Fuster MM, et al. Characterization of heparin and severe acute respiratory syndrome-related coronavirus 2 (SARS-CoV-2) spike glycoprotein binding interactions. Antiviral Res. 2020;181:104873. doi:10.1016/j.antiviral.2020.104873.
63. Möller S, Theiß J, Deinert TIL, Golat K, Heinze J, Niemeyer D, et al. High-Sulfated Glycosaminoglycans Prevent Coronavirus Replication. Viruses. 2022;14 2 doi:10.3390/v14020413.
64. Kwon PS, Oh H, Kwon SJ, Jin W, Zhang F, Fraser K, et al. Sulfated polysaccharides effectively inhibit SARS-CoV-2 in vitro. Cell Discov. 2020;6 1:50. doi:10.1038/s41421-020-00192-8.
65. Williams JB, Cao Q and Yan Z. Transcriptomic analysis of human brains with Alzheimer's disease reveals the altered expression of synaptic genes linked to cognitive deficits. Brain Commun. 2021;3 3:fcab123. doi:10.1093/braincomms/fcab123.
66. Zattoni M, Mearelli M, Vanni S, Colini Baldeschi A, Tran TH, Ferracin C, et al. Serpin Signatures in Prion and Alzheimer's Diseases. Mol Neurobiol. 2022;59 6:3778-99. doi:10.1007/s12035-022-02817-3.
67. Parker WD, Jr., Parks J, Filley CM and Kleinschmidt-DeMasters BK. Electron transport chain defects in Alzheimer's disease brain. Neurology. 1994;44 6:1090-6. doi:10.1212/wnl.44.6.1090.
68. Tian Y, Tian X, Han X, Chen Y, Song CY, Jiang WJ, et al. ABCE1 plays an essential role in lung cancer progression and metastasis. Tumour Biol. 2016;37 6:8375-82. doi:10.1007/s13277-015-4713-3.
69. Sivakumar S, Moore JA, Montesion M, Sharaf R, Lin DI, Colón CI, et al. Integrative Analysis of a Large Real-World Cohort of Small Cell Lung Cancer Identifies Distinct Genetic Subtypes and Insights into Histologic Transformation. Cancer Discov. 2023;13 7:1572-91. doi:10.1158/2159-8290.Cd-22-0620.
70. Wang H, Wang X, Xu L, Lin Y and Zhang J. CCT6A and CHCHD2 Are Coamplified with EGFR and Associated with the Unfavorable Clinical Outcomes of Lung Adenocarcinoma. Dis Markers. 2022;2022:1560199. doi:10.1155/2022/1560199.
71. Catelain C, Pailler E, Oulhen M, Faugeroux V, Pommier AL and Farace F. Detection of Gene Rearrangements in Circulating Tumor Cells: Examples of ALK-, ROS1-, RET-Rearrangements in Non-Small-Cell Lung Cancer and ERG-Rearrangements in Prostate Cancer. Adv Exp Med Biol. 2017;994:169-79. doi:10.1007/978-3-319-55947-6_9.
72. Wei Y, Vellanki RN, Coyaud É, Ignatchenko V, Li L, Krieger JR, et al. CHCHD2 Is Coamplified with EGFR in NSCLC and Regulates Mitochondrial Function and Cell Migration. Mol Cancer Res. 2015;13 7:1119-29. doi:10.1158/1541-7786.Mcr-14-0165-t.

73. Riely GJ, Marks J and Pao WJPotATS. KRAS mutations in non–small cell lung cancer. 2009;6 2:201-5.
74. Wang Z, Yang M-Q, Lei L, Fei L-R, Zheng Y-W, Huang W-J, et al. Overexpression of KRT17 promotes proliferation and invasion of non-small cell lung cancer and indicates poor prognosis. 2019:7485-97.
75. Shi X, Zhan L, Xiao C, Lei Z, Yang H, Wang L, et al. miR-1238 inhibits cell proliferation by targeting LHX2 in non-small cell lung cancer. 2015;6 22:19043.
76. Carpagnano GE, Palladino GP, Lacedonia D, Koutelou A, Orlando S and Foschino-Barbaro MPJBc. Neutrophilic airways inflammation in lung cancer: the role of exhaled LTB-4 and IL-8. 2011;11 1:1-9.
77. Luo L, Zheng Y, Lin Z, Li X, Li X, Li M, et al. Identification of SHMT2 as a Potential Prognostic Biomarker and Correlating with Immune Infiltrates in Lung Adenocarcinoma. J Immunol Res. 2021;2021:6647122. doi:10.1155/2021/6647122.

FIGURE LEGENDS

Figure 1: Schematic description of scGraph2Vec. (**A)** Feature extraction. The input of scGraph2Vec is an adjacency matrix from the gene interaction network and a feature matrix from single-cell data. The membership matrix derived from the primary community is calculated by the adjacency matrix using the Louvain algorithm [36] and is added to the adjacency matrix. The model adopts the modified VGAE framework. The encoder is composed of a 3-layer GCN network, and the decoder considers both the reconstruction of the original dimension and the detection of the node community. The latent features were extracted as model output and reduced to two-dimensional by t-SNE. Gene modules were obtained using a hierarchical clustering algorithm. (**B)** The inference of disease-associated genes. Seed genes can be defined using GWAS data or known driver genes. Candidate disease-associated genes can be identified by density-based clustering [49] using latent features extracted from disease-related tissues.

Figure 2: Performance evaluation through comparing the differences of latent features under different conditions. (A) Comparison of silhouette coefficients among standard outputs of scGraph2Vec and other variants using brain and PBMC datasets. The X-axis is the number of clusters. The Y-axis is the silhouette coefficients. **(B)** Comparison of silhouette coefficients among scGraph2Vec and eight tools using brain and PBMC datasets. The method scapGNN was excluded as it failed to extract clear gene modules. The X-axis is the number of clusters. The Y-axis is the silhouette coefficients. **(C, D)** Comparison of gene embedding visualizations on the same brain and PBMC datasets shows that genes are more distinctly separated between clusters

when embedded using scGraph2Vec. Except for SAUCIE, which uses the original two-dimensional features and clustering results, all other methods cluster genes through hierarchical clustering method and use t-SNE for visualization.

Figure 3: Biological implications of latent features from brain tissue. (A) Comparison of inner and outer closeness centralities for all gene modules using the Wilcoxon rank-sum test. Each dot represents a gene module. **(B)** The distribution of housekeeping genes in brain latent features. **(C)** The left plot is the t-SNE plot of brain scRNA-seq data. The color indicated cell types labeled from the original article [27]. The right plot shows the module score of each cell for cluster 59. **(D)** The scatter plot shows the GSEA results. The X-axis represents gene modules that had at least one enriched cell type ($P_{BH} < 0.05$). The Y-axis is the -log10(P_{BH}) for the enrichment test of each module with cell-type DEGs. The color indicated cell types, including excitatory (Ex) and inhibitory (In) neuronal subtypes, cerebellar granule (Gran) cells, Purkinje (Purk) neurons, and non-neuronal cells, such as pericytes (Per), astrocytes (Ast) and oligodendrocytes precursor cells (OPCs). **(E)** The heatmap of normalized enrichment score (NES) from GSEA results. **(F)** The GSEA enrichment results for cluster 9 and two cell types (i.e., Ast and In1c).

Figure 4: Tissue-specific modules and functional analysis. (A) GO enrichment for cluster 9. **(B)** GO enrichment for cluster 59. **(C)** The heatmap plot shows the brain-specific gene modules. We performed hypergeometric tests for overlapping genes in each module versus each other tissue module. The significant modules were defined as $P < 0.05$ and less than 5% overlapping

genes with all other tissue modules. **(D)** The predicted subnetwork (left) and GO enrichment (right) of *SMARCE1* adjacent genes from latent feature space of brain tissue. **(E)** The predicted subnetwork (left) and GO enrichment (right) of *SMARCE1* adjacent genes from latent feature space of lung tissue.

Figure 5: Inference of COVID-19-associated genes. (A) Volcano plot displaying GWAS-reported genes. The X-axis represents the log2(FC) of genes in COVID-19 and non-COVID-19 groups. The Y-axis represents the associated gene from MAGMA analysis (gene-based $P < 2.63 \times 10^{-6}$ by Bonferroni correction). The dashed lines represent the threshold of significant associated genes ($P < 2.63 \times 10^{-6}$) and differential expression gene threshold ($|\log2(FC)| > 1$). **(B, C)** Differential expression analysis of COVID-19-associated genes inferred from lung latent features (B) and brain latent features (C). The dashed lines represent the threshold of significant DEGs ($P_{BH} < 0.05$, $|\log2(FC)| > 1$). **(D)** The expression profiles for COVID-19-associated candidate genes were inferred from lung tissue. The samples in the columns were grouped by COVID-19 positive samples and COVID-19 negative samples, and the genes on the rows were clustered according to Ward's method. **(E)** GO enrichment of all candidate genes (left) and *CEACAM8* neighbor genes (right) from lung tissues.

Figure 6: Inference of AD-associated genes. (A) Volcano plot displaying GWAS-reported genes. The X-axis represents the log2 (FC) of genes in AD and non-AD groups. The Y-axis represents the associated gene significant level after Bonferroni correction ($P < 3.77 \times 10^{-6}$). The

dashed lines represent the threshold of significant associated genes ($P < 3.77 \times 10^{-6}$) and differential expression gene threshold (|fold change| > 1.1). **(B, C)** Differential expression analysis AD-associated genes inferred from brain latent features (B) and lung latent features (C). The dashed lines represent the threshold of significant DEGs ($P_{BH} < 0.05$, |fold change| > 1.1). **(D)** AD-associated gene expression profiles inferred from brain tissue. The samples in the columns were grouped by AD positive samples and AD negative samples, and the genes on the rows were clustered according to Ward's method. **(E)** GO analysis of all neighbor genes (left) and *APOE* neighbor genes (right) from brain tissues.

Figure 7: Discovery of additional tumor-driver genes. (A) The heatmap plot shows the tumor-specific gene modules. We performed hypergeometric tests for overlap genes in each tumor module vs. each normal module. The significant modules were defined as $P < 0.05$ and less than 5% overlapping genes with all other normal modules. **(B)** The most significant GO terms enriched for tumor-specific module, containing only biological processes. **(C)** Compare the top 10 GO terms of biological process enriched in *TP53* neighbor genes in tumor and normal tissues, and sort them according to P_{BH}. **(D)** Boxplot shows the expression differences of *TP53* neighbor genes in TCGA LUAD samples. Use the Wilcoxon test to compare the log2 (TPM + 1) values of the same gene in tumor samples and normal samples. *, $P < 0.05$; **, $P < 0.01$; ***, $P < 0.001$; ****, $P < 0.0001$. **(E)** The Venn diagram shows the overlap of the four gene sets of up-regulated DEGs, down-regulated DEGs, HR > 1 and HR < 1. **(F, G)** Kaplan-Meier curve based on the survival time (days) of TCGA LUAD samples and the expression levels of *ABCE1* and *LTB*

genes. High expression and low expression were distinguished according to the median log2 (TPM + 1) value.

Figure1 Click here to access/download;Figure;Figure1.pdf

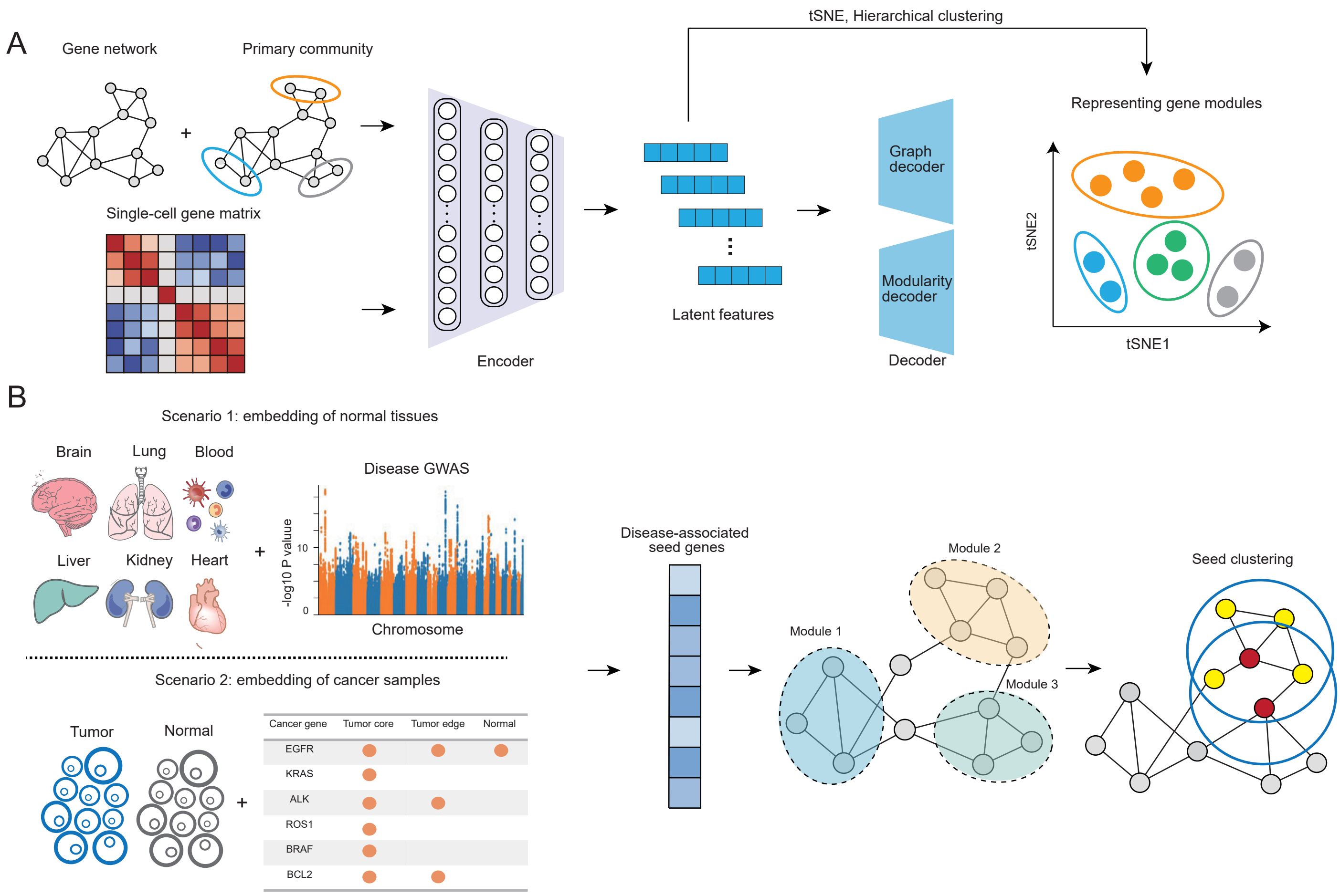

Figure2

Click here to access/download;Figure;Figure2.pdf

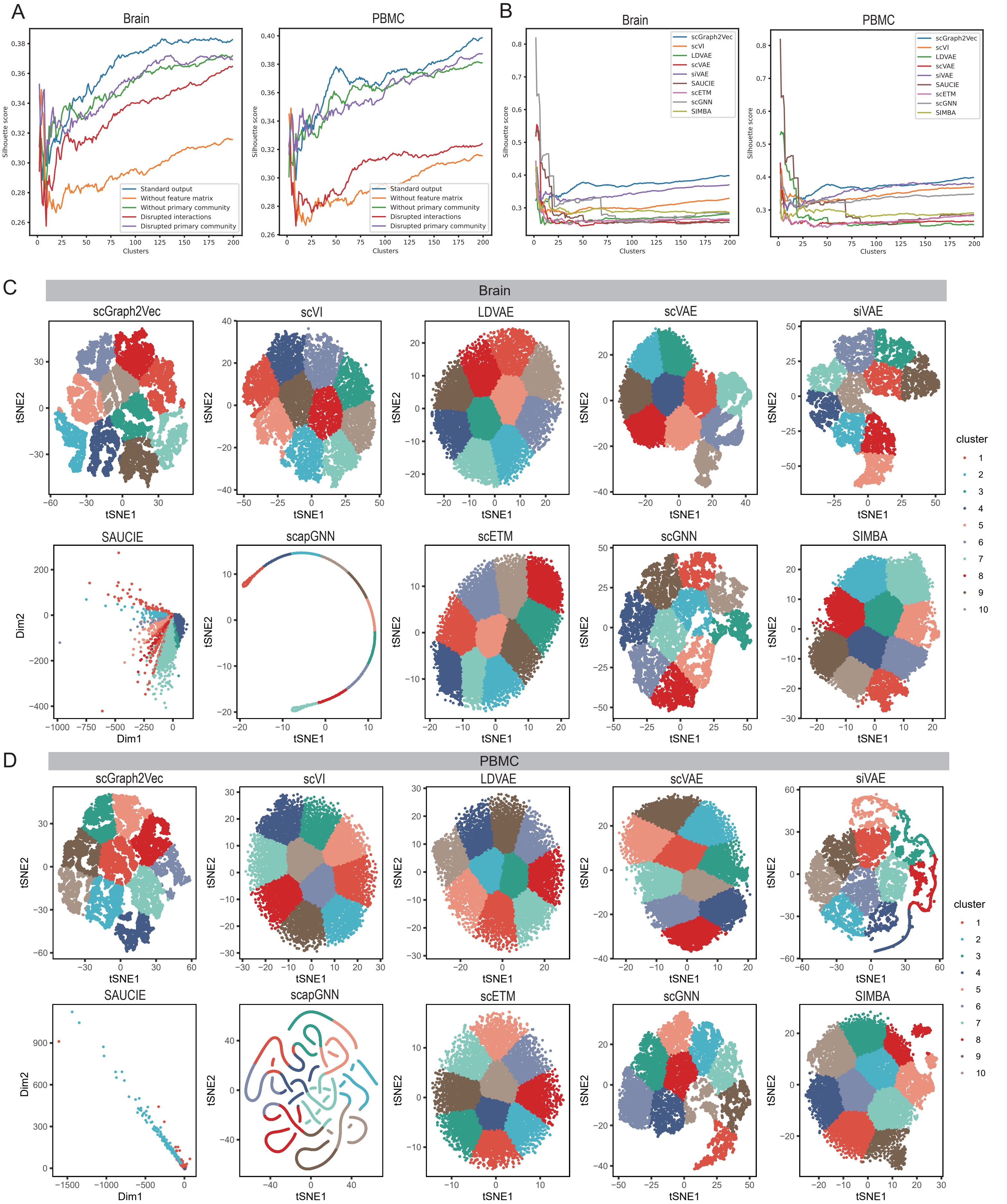

Figure3

Click here to access/download;Figure;Figure3.pdf

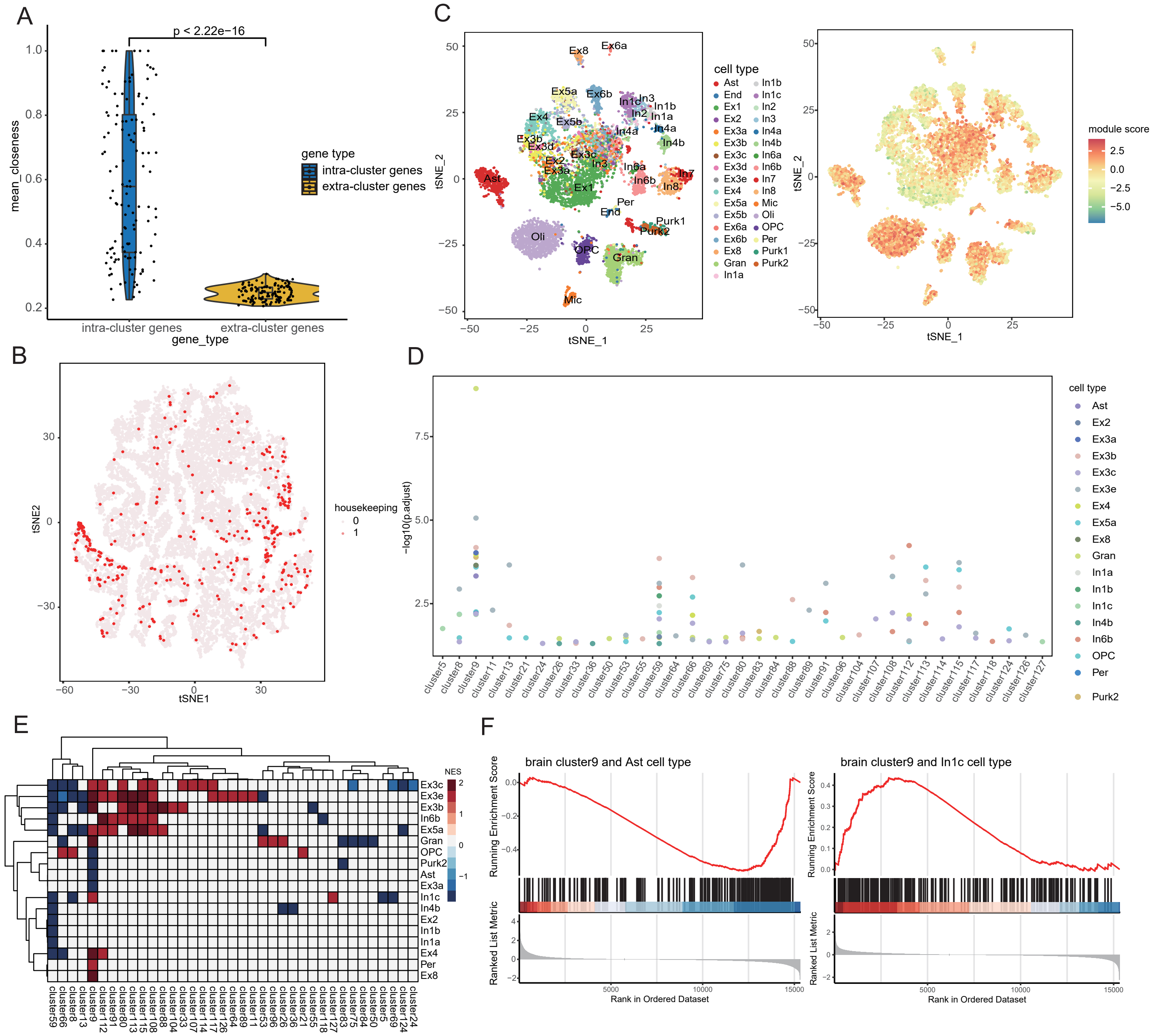

Figure4

Click here to access/download;Figure;Figure4.pdf

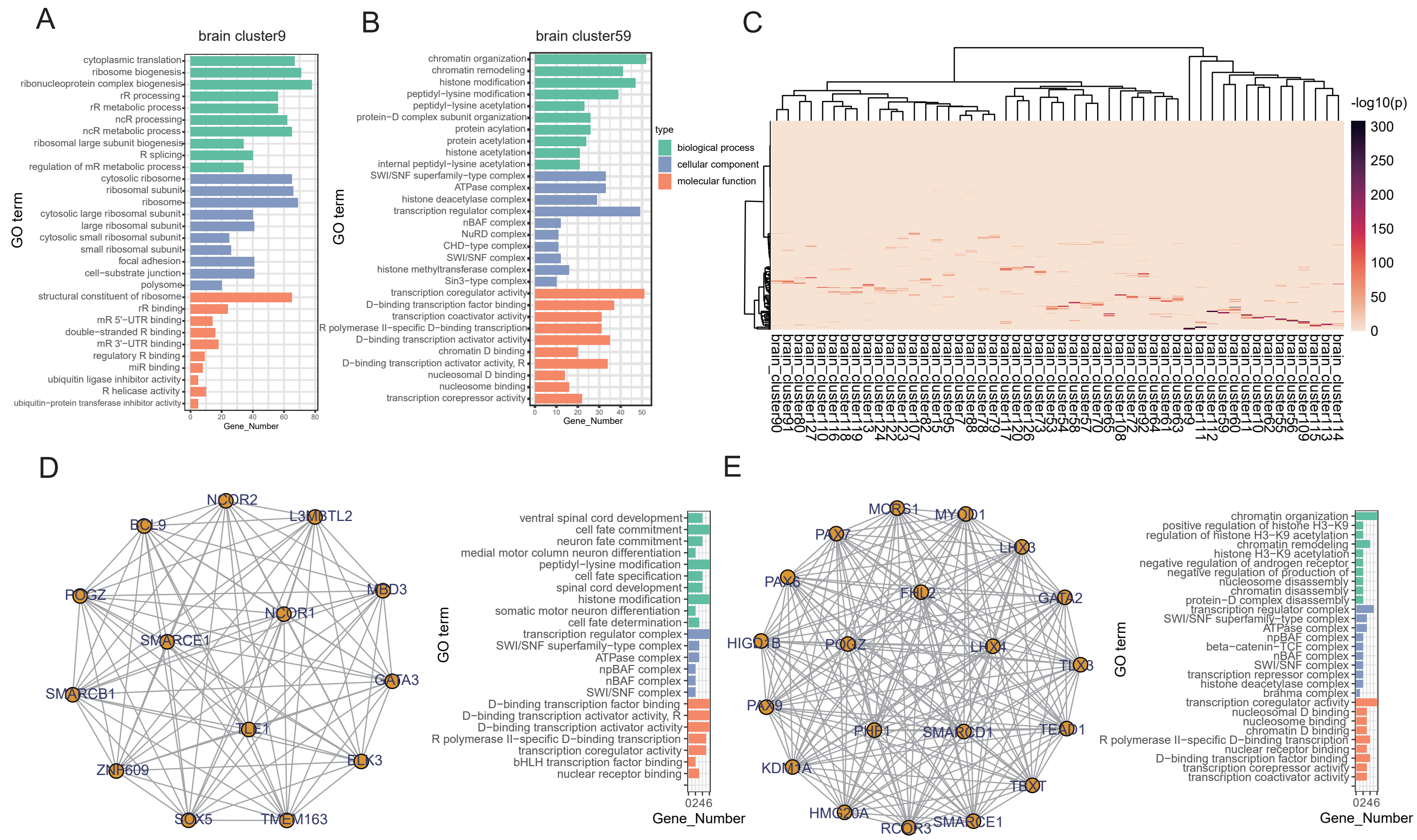

Figure5

Click here to access/download;Figure;Figure5.pdf

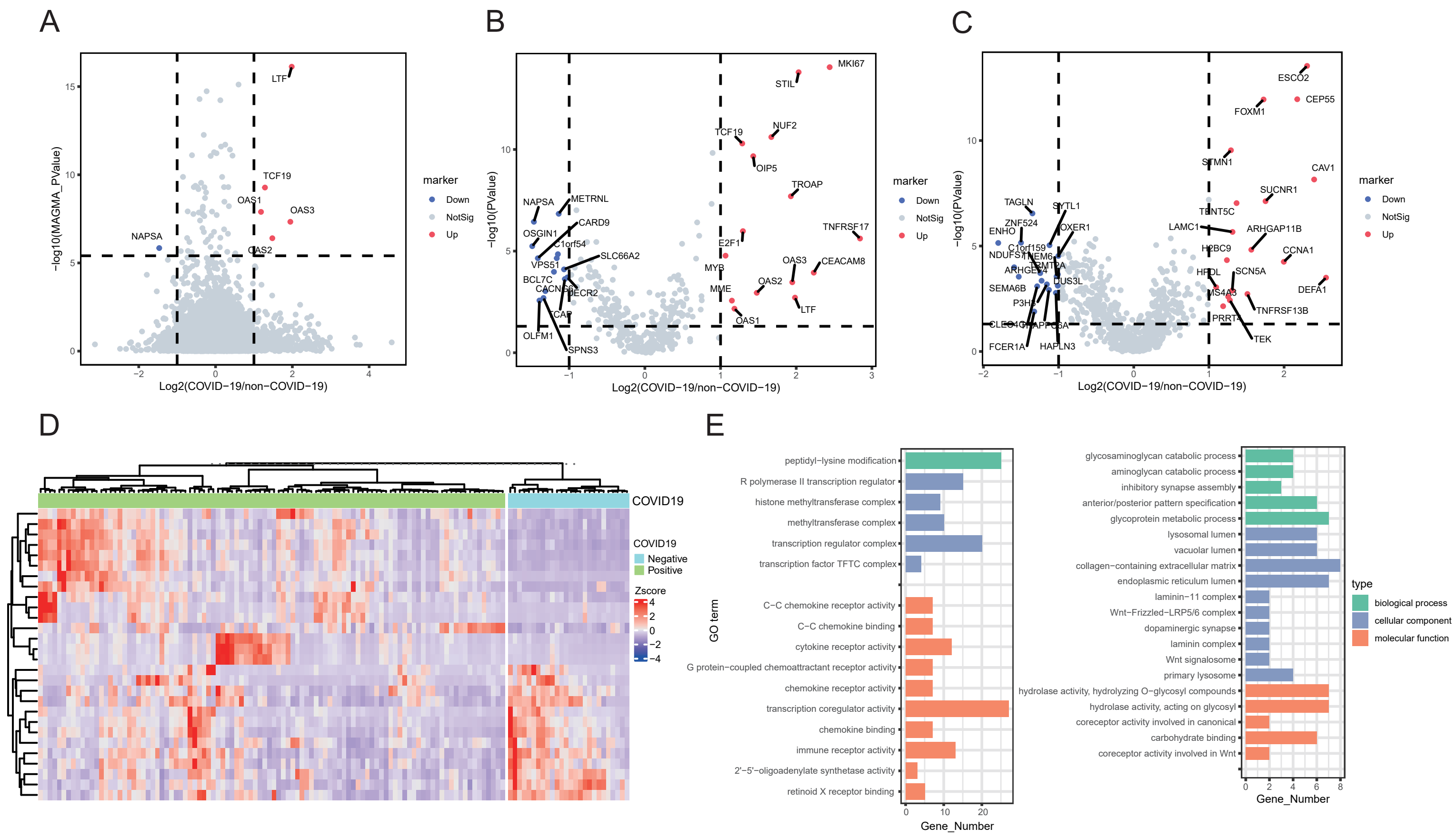

Figure6

Click here to access/download;Figure;Figure6.pdf

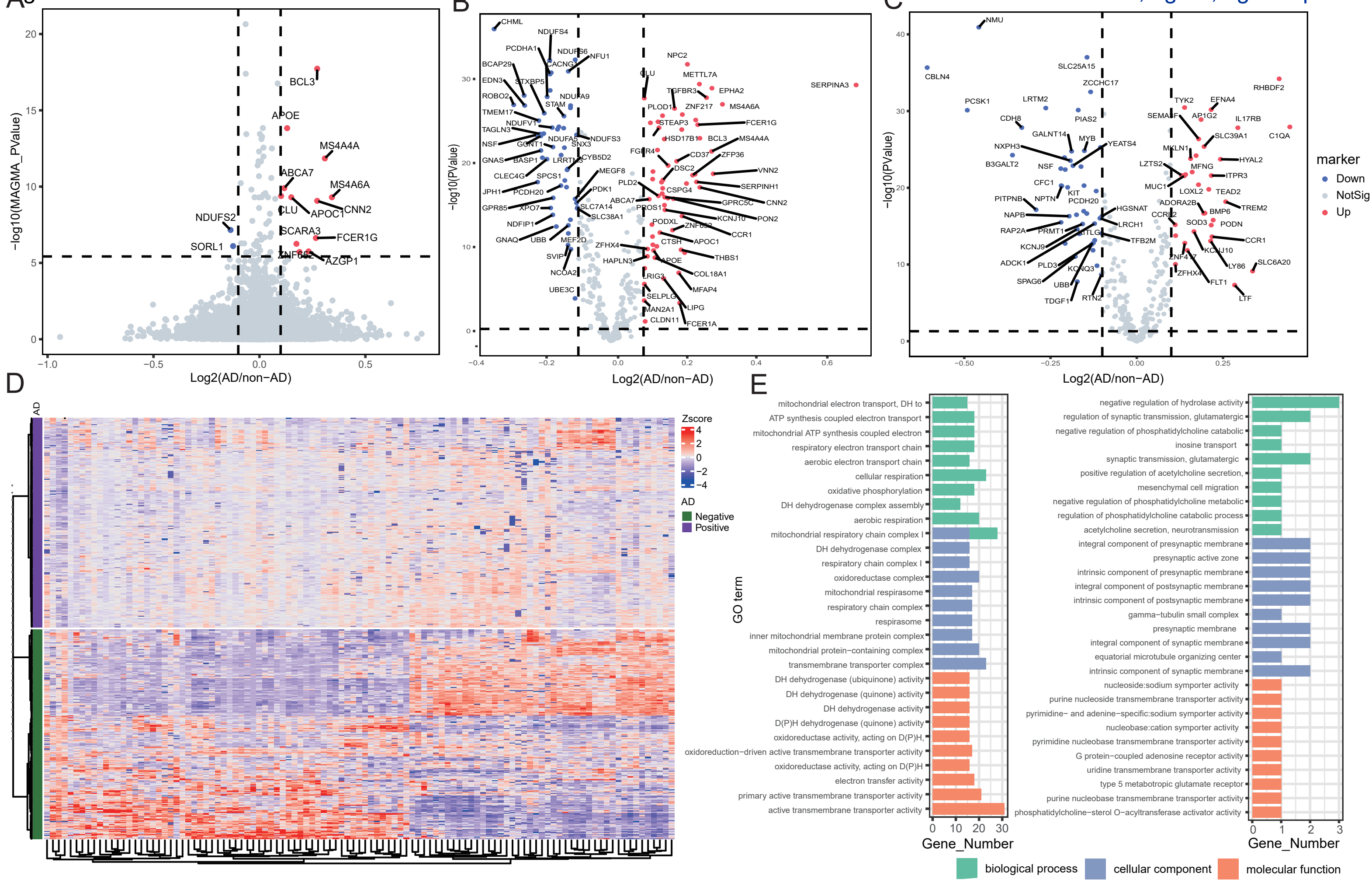

Figure7

Click here to access/download;Figure;Figure7.pdf

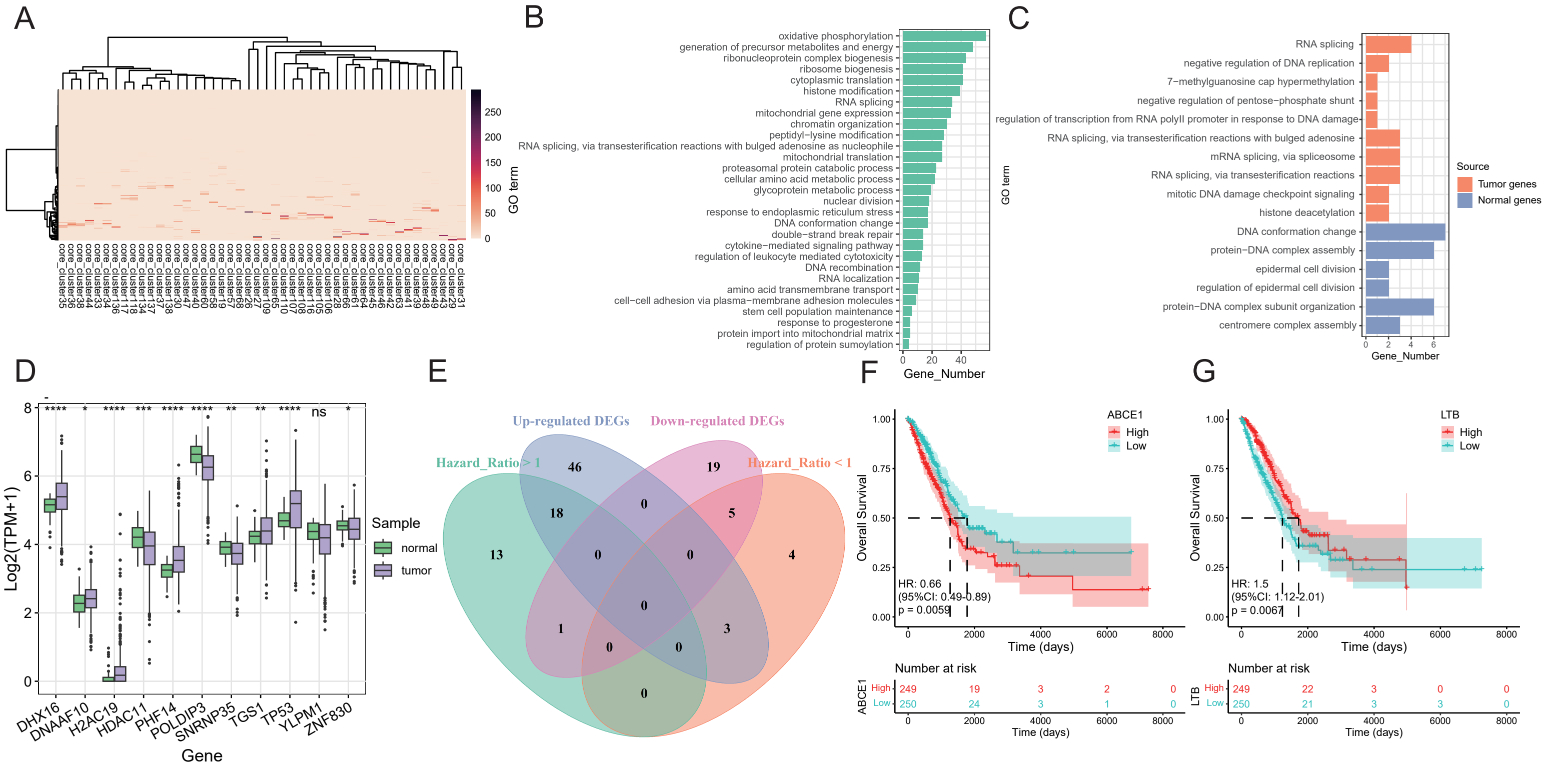

Additional file 1

Click here to access/download
Supplementary Material
Supporting Information.docx

Additional file 2

Click here to access/download
Supplementary Material
Supplementary_Table.xlsx
