# Supplementary material for: scGraph2Vec: a deep generative model for gene embedding augmented by graph neural network and single-cell omics data
Source: Gigascience. 2024 Dec 20;13:giae108. doi: 10.1093/gigascience/giae108 (PMC11659981; doi:10.1093/gigascience/giae108)
Supplement: giae108_GIGA-D-24-00200_Revision_1 [file giae108_giga-d-24-00200_revision_1.pdf]

GigaScience

scGraph2Vec: a deep generative model for gene embedding augmented by Graph Neural Network and single-cell omics data

--Manuscript Draft--

**Manuscript Number:**	GIGA-D-24-00200R1
**Full Title:**	scGraph2Vec: a deep generative model for gene embedding augmented by Graph Neural Network and single-cell omics data
**Article Type:**	Technical Note
**Funding Information:**	the Strategic Priority Research Program of the Chinese Academy of Sciences (XDB38010400) — Prof. Peilin Jia<br>the National Natural Science Foundation of China (32270706) — Prof. Peilin Jia<br>the Shanghai Municipal Science and Technology Major Project (2018SHZDZX01) — Ms. Shiqi Lin
**Abstract:**	Background<br><br>Exploring the cellular processes of genes from the aspects of biological networks is of great interest to understanding the properties of complex diseases and biological systems. Biological networks, such as protein-protein interaction networks and gene regulatory networks, provide insights into the molecular basis of cellular processes and often form functional clusters in different tissue and disease contexts.<br><br>Results<br><br>We present scGraph2Vec, a deep-learning framework for generating informative gene embeddings. scGraph2Vec extends the variational graph autoencoder framework and integrates single-cell datasets and gene-gene interaction networks. We demonstrate that the gene embeddings are biologically interpretable and enable the identification of gene clusters representing functional or tissue-specific cellular processes. By comparing similar tools, we showed that scGraph2Vec clearly distinguished different gene clusters and aggregated more biologically functional genes. scGraph2Vec can be widely applied in diverse biological contexts. We illustrated that the embeddings generated by scGraph2Vec can infer disease-associated genes from genome-wide association study data (e.g., COVID-19 and Alzheimer’s Disease), identify additional driver genes in lung adenocarcinoma, and identify regulatory genes responsible for maintaining or transitioning melanoma cell states.<br><br>Conclusions<br><br>scGraph2Vec not only reconstructs tissue-specific gene networks but also obtains a latent representation of genes implying their biological functions.
**Corresponding Author:**	Peilin Jia, Ph.D.<br>Beijing Institute of Genomics Chinese Academy of Sciences<br>Beijing, CHINA
**Corresponding Author Secondary Information:**	
**Corresponding Author's Institution:**	Beijing Institute of Genomics Chinese Academy of Sciences
**Corresponding Author's Secondary Institution:**	
**First Author:**	Shiqi Lin
**First Author Secondary Information:**	
**Order of Authors:**	Shiqi Lin

Powered by Editorial Manager® and ProduXion Manager® from Aries Systems Corporation

	Peilin Jia, Ph.D.
**Order of Authors Secondary Information:**	
**Response to Reviewers:**	Response to Referee #1:<br>The main contribution of this study lies in the proposal of a new model, scGraph2Vec, which leverages the powerful representational capabilities of Graph Neural Networks (GNNs) to capture the intricate interaction between genes, while incorporating omics information at the single-cell level. This approach facilitates a deeper understanding for researchers of gene regulation, cell type differentiation, and the molecular mechanisms underlying diseases. However, the content of this manuscript requires further enhancement, particularly in elucidating the novel biological insights or advantages offered by the gene embeddings generated by this model compared to those gene modules derived from other state-of-the-art algorithms.<br><br>Response: We thank the reviewer for the positive evaluation and suggestions of our manuscript. Following your suggestion, we compared the gene embeddings by scGraph2Vec with those derived by other state-of-the-art algorithms. In total, we compared 13 gene embedding methods published in recent years and included two additional representative gene clustering methods, SCENIC and LIGER. Additionally, we incorporated a comparison of three types of gene networks to highlight the unique advantage of scGraph2Vec in integrating gene interaction networks with single-cell omics data. We assessed the ability of each method to generate gene embeddings and gene clusters. To evaluate the biological advantages of each method, we used the MsigDB hallmark gene set as a ground truth.<br>The results showed that scGraph2Vec effectively combined diverse gene interaction networks with single-cell omics to generate highly informative gene embeddings. These embeddings capture both network-specific interaction features and biological context-specific expression patterns, allowing for clearer separation of gene clusters and more precise identification of co-functional gene programs.<br><br>In addition to the shortcomings in novelty and biological significance within this manuscript, here are some revision suggestions:<br>#1. In Fig. 2, the authors show that the gene embeddings acquired through the scGraph2Vec model achieve higher silhouette scores than those resulting from alternative algorithms. Nonetheless, they fail to expound upon the biological relevance of these gene embeddings, nor do they illustrate that the gene embeddings procured via scGraph2Vec encompass greater or more precise biological insights relative to the gene modules yielded by competing algorithms.<br><br>Response: We thank the reviewer for this valuable suggestion. During revision, we compared scGraph2Vec with 13 gene embedding methods and two representative gene clustering methods, SCENIC and LIGER. We evaluated the gene clusters using the silhouette coefficient and Davies-Bouldin Index and assessed the similarity of gene clusters using the Jaccard index. To ensure a fair evaluation of biological relevance, we introduced the MsigDB hallmark gene sets as the ground truth to measure the functions of gene clusters. The results showed that scGraph2Vec outperformed nearly all other methods in separating gene clusters, except for siVAE, which performed better on the PBMC dataset (Fig. 2B-C and Supplementary Fig. 9). We observed significant differences in the gene clusters generated by these methods (Fig. 2D). However, when compared to the MsigDB hallmark gene sets, scGraph2Vec clustered the highest number of functionally similar genes among all competing algorithms (Fig. 2E).<br>Additionally, we explored the biological relevance of gene embeddings from several perspectives, including network topology, clustering of annotated gene sets, cell type specificity, and tissue specificity.<br>(1) Network topology. The internal closeness centrality of genes in each cluster was significantly higher than their external closeness centrality (Fig. 3A).<br>(2) The clustering of annotated genes. Genes with similar functions, e.g., the housekeeping genes and the hallmark genes, tend to cluster together (Fig. 3B, C and Supplementary Fig. 13).<br>(3) Cell type specificity. When we applied scGraph2Vec to data from six representative human tissues, gene embeddings helped us identify clusters with cell type-specific expression (Fig. 4A, B and Supplementary Fig. S15). For instance, cluster 59 was enriched in multiple excitatory and inhibitory neurons and was particularly associated

Powered by Editorial Manager® and ProduXion Manager® from Aries Systems Corporation

with down-regulated differentially expressed genes (DEGs) in these cell types (Fig. 4A and Supplementary Fig. S15).
(4) Tissue specificity. Embeddings also captured tissue-specific functional clusters. For example, our comparison of SMARCE1's neighboring genes in the brain and lung revealed distinct functions: in brain tissue, 14 neighboring genes were enriched in neuronal fate commitment and motor neuron differentiation (Fig. 4F), while in lung tissue, 20 neighboring genes were enriched in chromatin organization and histone H3-K9-related functions (Fig. 4G).
(5) Four applications to identify critical genes in different biological contexts. The four application cases, i.e., COVID-19, Alzheimer’s disease, lung adenocarcinoma, and melanoma, illustrated that scGraph2Vec can be widely used in various scenarios and identify candidate genes critical to the biological conditions, be it disease-associated genes, cancer driver genes, or regulatory genes for cell states.
In summary, scGraph2Vec integrates various gene networks and single-cell omics data from diverse biological backgrounds to generate highly informative gene embeddings. These embeddings capture both the topological characteristics of biological networks and biologically significant gene clusters, uncovering context-specific gene regulatory programs.

#2: In Fig. 2B, the authors indicated that "housekeeping genes tended to cluster together and were enriched in a few gene modules". Does this suggest that these gene modules are related to cell maintenance functions or states?

Response: We thank the reviewer for pointing this out. We extracted gene clusters containing more than 20 housekeeping genes in the six normal human tissues and conducted functional analysis (See Table 1). Interestingly, in all tissues, the clusters with the largest number of housekeeping genes were all enriched with cytoplasmic translation and ribosome biogenesis, indicating that these clusters were involved in the basic functions of cell maintenance.

#3: The authors used module score to represent the overall expression pattern of module genes, is there mathematical proof for this? Is there biological evidence to support this claim? Is there a ground truth available to validate its reliability?

Response: We thank the reviewer for these valuable suggestions. In the first submission, we used the module eigengene to calculate the module score. This procedure followed the Weighted correlation network analysis (WGCNA) algorithm and had been well-validated in the original WGCNA study as well as in many studies citing WGCNA [1]. However, during revision, we found that the “AddModuleScore” function in the Seurat software was more frequently used in scRNA-seq data analyses, especially to summarize the expression patterns of gene sets in different cell types [2]. Thus, in the revised manuscript, we updated our analysis strategy and used the “AddModuleScore” to calculate module scores.

#4: In Fig. 4, the authors performed a GO-term analysis of the gene modules obtained from the scGraph2Vec model, and utilized the functions of these modules to investigate the functionalities of various cell types. What are the advantages of this approach compared to directly using differentially expressed genes (DEGs), or gene modules obtained from other algorithms?

Response: We thank the reviewer for this valuable suggestion. scGraph2Vec aims to identify gene clusters with coordinated expression patterns across cells. It does not use predefined cell type information during training. The relative distances among genes in the two-dimensional embedding space reflect their biological relationships, capturing not only cell type-specific expression patterns but also the topological characteristics of the gene interaction network. In contrast, directly using DEGs focuses on comparisons between cell types but does not consider the coordinated gene expression across multiple cells. In case studies such as COVID-19, Alzheimer’s disease (AD), lung adenocarcinoma (LUAD), and melanoma, the density-based search algorithm allowed us to identify genes associated with candidate disease genes within the embedding space. This highlights the unique strengths of gene embeddings in uncovering additional disease-associated genes and revealing regulatory genes specific to different biological contexts.

Powered by Editorial Manager® and ProduXion Manager® from Aries Systems Corporation

#5: In studies on COVID-19 and Alzheimer's disease, the authors utilized disease-related variants identified through GWAS analysis, combined with information from gene modules, to screen for genes associated with these diseases. How did the authors determine that these variants in the genes would lead to changes in the RNA expression levels of these genes? Was it because these variant sites were located in promoter or enhancer regions of these genes? Is there any experimental evidence for this?

Response: We thank the reviewer for pointing this out. We used the MAGMA software to calculate gene-based p-values and defined seed genes by considering the study-wide significant level based on the Bonferroni correction. MAGMA maps SNPs to genes based on genomic locations, assigning an SNP to a gene if the SNP's location falls inside the gene region. In addition, SNPs near a gene but outside its transcription region can be mapped to that gene using a pre-defined window. We used the default settings by MAGMA, i.e., "--annotate window=5,1.5", where SNPs located 5kb upstream and 1.5kb downstream were also mapped to a gene. Then, MAGMA implemented a multiple linear principal components regression model and an F-test to compute the gene p-values. The MAGMA pipeline has been widely used in various GWAS analysis applications.

#6: Some paragraphs and sentences in this manuscript have rather stiff language expression. The authors can use English proofreading service to prevent grammatical and semantic errors in expression.

Response: Thank you for your valuable feedback. During revision, we have carefully revised the text to improve clarity and readability. Additionally, we have considered professional English proofreading services to further enhance the manuscript and ensure that it meets the highest linguistic standards. We appreciate your suggestion and will make every effort to improve the overall quality of the writing.

Response to Referee #2:
The authors have proposed a computational method called 'scGraph2Vec' that takes a single-cell gene expression profile and a gene interaction network as input, and produces low-dimensional gene embedding as output. The links to the used datasets are either provided in the Supplementary Table S1 or mentioned in the main text. The source code has been made publicly available at https://github.com/LPH-BIG/scGraph2Vec. I highly appreciate the effort the authors have made to improve the reproducibility of their work. It would be great if the authors could address the following comments to strengthen the publication.

Response: We thank the reviewer for the positive comments on our manuscript. Each of these comments has been considered in the revised manuscript and is discussed below.

#1: The background section needs to be significantly improved. It has been written that scGraph2Vec "extends the VGAE framework". However, VGAE is a data-agnostic framework; scGraph2Vec rather utilises the VGAE framework for analysing gene expression and gene interaction network data. Therefore, the authors need to incorporate a critical review of the recent method that do the same. I am listing a few references I could find for that purpose:
https://academic.oup.com/nar/article/51/13/6578/7184155
https://isip.piconepress.com/conferences/ieee_spmb/2022/papers/p01_07.pdf
http://dr.iiserpune.ac.in:8080/xmlui/handle/123456789/7885
https://arxiv.org/abs/1802.04407
There are more references that I could not list here. I request the authors to enhance the background section by reviewing such methods.

Response: Thank you for your insightful comments. In the revised manuscript, we have reinforced a critical review of recent VGAE-based embedding methods in the background section.

#2: In addition to the critically reviewing other VGAE-based gene embedding methods, I request the authors to include them in the comparisons based on the benchmark datasets.

Powered by Editorial Manager® and ProduXion Manager® from Aries Systems Corporation

Response: We thank the reviewer for this valuable suggestion. Following your suggestion, during the revision process, we compared scGraph2Vec with four VGAE-based embedding methods and nine single-cell embedding methods.
Notably, we were unable to run the two methods cited in the first and second references by the due date of this revision. The first method, GenKI, was designed to capture shifting patterns in gene regulation caused by knockout (KO) perturbations. In our attempt, GenKI took days to construct the gene networks. Even after we reduced the genes to 1,865 highly variable genes in PBMC and 5,182 in the brain, we still could not obtain results using 60 CPU cores after a month. For the second reference, we were unable to test the method as its source code was unavailable.
Thus, we focused on the other VGAE-based embedding methods, including the highly cited SIG-VAE and DGVAE. SIG-VAE extends VGAE's flexibility by using a hierarchical variational framework and a Bernoulli-Poisson decoder, allowing for better modeling of complex graph dependencies and implicit posteriors. DGVAE, on the other hand, introduces graph cluster memberships as latent factors, demonstrating strong performance in graph generation and clustering tasks. In total, we benchmarked 13 VGAE-based embedding methods (ARGA, ARGVA, SIG-VAE, DGVAE, scVI, LDVAE, siVAE, scVAE, scGNN, scapGNN, scETM, SAUCIE and SIMBA) against scGraph2Vec.
We systematically evaluated the ability of different methods to divide clusters, the similarity of gene clusters among methods, and the overlap of each cluster with the MsigDB hallmark gene sets. The results showed that scGraph2Vec could divide different gene clusters more clearly than other methods, except siVAE on the PBMC dataset (Fig. 2B-C). We found that the gene clusters identified by different methods were quite different (Fig. D). However, when compared with the MsigDB hallmark gene sets, the clusters by scGraph2Vec tended to have higher proportions of the hallmark genes, i.e., genes with common functions, than other competing algorithms (Fig. 2E). These results indicated that the clusters by scGraph2Vec tended to be more functionally correlated.

#3: The authors have utilized the gene embedding outputs of scGraph2Vec for clustering genes. Therefore, I request the authors to compare the resultant gene clusters with that of other single-cell gene expression based gene clustering methods such as SCENIC (https://www.nature.com/articles/nmeth.4463) and LIGER (https://www.cell.com/cell/pdf/S0092-8674(19)30504-5.pdf).

Response: We thank the reviewer for bringing this point up. SCENIC and LIGER generate gene clusters but do not generate gene embeddings. Thus, we applied SCENIC and LIGER using the same brain and lung datasets as we used in scGraph2Vec and generated gene clusters. Then we compared the gene clusters identified by SCENIC and LIGER in the gene embedding space identified by scGraph2Vec. Our analysis revealed that SCENIC and LIGER gene clusters were not closely distributed in our embedding space and did not overlap significantly with our scGraph2Vec clusters (Supplementary Fig. 10). By assessing the similarity of the gene clusters (by SCENIC, LIGER, and scGraph2Vec, respectively) with the MsigDB hallmark gene set, we found that the gene clusters by scGraph2Vec showed a higher proportion of the hallmark genes (Fig. 2E). Thus, we consider that scGraph2Vec can generate gene clusters more functionally correlated.

#4: The authors have used the silhouette index to compare the gene clusters produced by different methods. I would request the authors to incorporate additional evaluation metrics such as the Davies-Bouldin index (DBI) and Jaccard index.

Response: We thank the reviewer for raising this important point. During revision, we have included the Davies-Bouldin Index and the Jaccard Index to compare the gene clusters (Fig. 2).

Response to Referee #3:
The manuscript introduces a novel deep-learning framework designed to generate informative gene embeddings. This framework, scGraph2Vec, extends the variational graph autoencoder (VGAE) model and integrates single-cell data patterns along with gene interaction networks. The model's effectiveness is demonstrated through its ability to produce biologically interpretable gene embeddings that reveal functional

modules of genes, which can be applied to identify disease-associated genes and understand tissue-specific cellular processes.

Response: We thank the reviewer for the positive comments on our manuscript.

However, the authors still have the following problems to solve:
#1. The manuscript compares scGraph2Vec with several existing gene embedding methods. It would be beneficial to include more recent methods, especially those with novel algorithmic approaches to network analysis.

Response: We thank the reviewer for this valuable suggestion. During the revision process, we systematically reviewed existing gene embedding methods published in recent years, especially those with novel algorithmic approaches to network analysis. In total, we selected 13 embedding methods (ARGA, ARGVA, SIG-VAE, DGVAE, scVI, LDVAE, siVAE, scVAE, scGNN, scapGNN, scETM, SAUCIE, and SIMBA) and 2 gene clustering methods (SCENC and LIGER) for comparison with scGraph2Vec.
All methods generated gene clusters. We employed three measurements to assess the gene clusters, i.e., the silhouette coefficient, the Davies-Bouldin Index, and the overrepresentation status of each cluster with the hallmark gene sets. The silhouette coefficient and the Davies-Bouldin Index measure how the clusters generated by each method can be clearly distinguished. As shown in Fig.2B-C in the revised manuscript, the clusters by scGraph2Vec outperformed nearly all other methods in the brain and PBMC tissues. The overrepresentation status of the hallmark genes measures the functional convergence of each cluster. As shown in Fig. 2E in the revised manuscript, scGraph2Vec generated gene clusters that shared the most with the hallmark genes, indicating that the clusters by scGraph2Vec were functionally convergent (Fig. 2E).

#2. The authors have used several datasets to validate the performance of scGraph2Vec. To further demonstrate the model's generalizability and robustness, testing on a broader range of datasets is recommended, especially those from different biological contexts or species.

Response: We thank the reviewer for this valuable suggestion. During the revision process, we included an additional application case by using melanoma cell lines with different states. In total, we have four applications representing three unique contexts (See Table 2). We hope these applications are satisfactory to answer your question.

#3. While the gene embeddings generated by scGraph2Vec are said to be biologically interpretable, the authors should further explore how these embeddings correspond to known biological processes and disease mechanisms. For the identified disease-associated genes, a more profound functional annotation and pathway analysis are suggested to uncover their specific roles in disease progression.

Response: We thank the reviewer for this valuable suggestion. To explore the biological relevance of gene embeddings, we conducted the following analyses.
(1) Network topology. The internal closeness centrality of genes in each cluster was significantly higher than their external closeness centrality (Fig. 3A).
(2) The clustering of annotated genes. Genes with similar functions, e.g., the housekeeping genes and the hallmark genes, tend to cluster together (Fig. 3B, C and Supplementary Fig. 13).
(3) Cell type specificity. When we applied scGraph2Vec to data from six representative human tissues, gene embeddings helped us identify clusters with cell type-specific expression (Fig. 4A, B and Supplementary Fig. S15). For instance, cluster 59 was enriched in multiple excitatory and inhibitory neurons and was particularly associated with down-regulated differentially expressed genes (DEGs) in these cell types (Fig. 4A and Supplementary Fig. S15).
(4) Tissue specificity. Embeddings also captured tissue-specific functional clusters. For example, our comparison of SMARCE1's neighboring genes in the brain and lung revealed distinct functions: in brain tissue, 14 neighboring genes were enriched in neuronal fate commitment and motor neuron differentiation (Fig. 4F), while in lung tissue, 20 neighboring genes were enriched in chromatin organization and histone H3-K9-related functions (Fig. 4G).
(5) Four applications to identify critical genes in different biological contexts. The four application cases, i.e., COVID-19, Alzheimer’s disease, lung adenocarcinoma, and

Powered by Editorial Manager® and ProduXion Manager® from Aries Systems Corporation

melanoma, illustrated that scGraph2Vec can be widely used in various scenarios and identify candidate genes critical to the biological conditions, be it disease-associated genes, cancer driver genes, or regulatory genes for cell states.
In summary, scGraph2Vec integrates various gene networks and single-cell omics data from diverse biological backgrounds to generate highly informative gene embeddings. These embeddings capture both the topological characteristics of biological networks and biologically significant gene clusters, uncovering context-specific gene regulatory programs.

For the identified disease-related genes, we conducted in-depth functional annotation and pathway analysis. Based on pathway databases such as KEGG, WikiPathways, and Reactome, we applied the R packages clusterProfiler (v.4.6.2) and ReactomePA (v.1.42.0) to perform pathway enrichment analysis. Pathway annotation was done with ClueGO (v.2.5.10). Protein-protein interaction analysis was carried out using STRING. In COVID-19, pathway enrichment analysis revealed significant enrichment in Glycosaminoglycan degradation (PBH = 3.08 × 10-4), type I interferon induction and signaling during SARS-CoV-2 infection (PBH = 6.46 × 10-4), Interleukin-10 signaling (PBH = 3.03 × 10-3), and ABO blood group biosynthesis (PBH = 3.29 × 10-3) (Fig. 5F). STRING analysis further demonstrated protein-protein interactions among genes involved in these pathways (Fig. 5G). In AD, our pathway annotation of 362 neighboring genes further revealed regulatory pathways involved in AD, mainly complex I biogenesis (PBH = 9.00 × 10-10), cholesterol transport (PBH = 1.07 × 10-4), regulation of cholesterol transport (PBH = 9.48 × 10-4), regulation of amyloid-beta clearance (PBH = 9.83 × 10-5) and oxidative phosphorylation (PBH = 3.67 × 10-9) (Fig. 6F, G).

#4. The manuscript mentions the optimization of model parameters through hyperparameter sweeping. The authors should provide more details on this process, including the specific ranges and tuning strategies used.

Response: We apologize for not providing the details of the hyperparameter sweeping process. In the revised manuscript, we added a detailed description of the hyperparameter selection and optimization process in the Methods section. Specifically, we considered standard VGAE parameters, i.e., learning rate, training iterations, encoding layers, and their dimensions, and new parameters that were specific to scGraph2Vec, i.e., λ, s, β, and γ. We tested λ (0.001, 0.1, 1), β (0.5, 5, 10), γ (0.1, 1), and s (2, 10). The optimal hyperparameters were determined by combining evaluation metrics, prioritizing a higher silhouette coefficient, a greater number of clusters, and a shorter running time. As a result, the optimal settings were λ=1,β=10,γ=0.1,s=10. Subsequently, we tested a two-layer encoder with dimensions 64-16, 128-16, 256-16, 128-32, and 256-32, as well as a three-layer encoder with dimensions 256-64-16 and 512-64-16. Finally, we recommended using a three-layer neural network (256-64-16) encoder. The Adam optimizer strategy and a learning rate = 110-4 were adopted. A maximum of 600 iterations and early stopping with patience = 50 was used. The model training was repeated 10 times in each tissue.

#5. For large-scale datasets, how efficient is scGraph2Vec in terms of computational resources? The authors are encouraged to assess and report the model's runtime and resource consumption, especially when dealing with large single-cell datasets.

Response: We apologize for not providing details about running processes. In the revised manuscript, we have added information about the computational resources. On page 20, in the last paragraph, we added:

“We used 64 logical CPUs and 512 GB RAM for all experiments. The execution time of scGraph2Vec depends on the size of the single-cell dataset and the interaction network. It ranges from 6.5 hours on the smallest dataset (PBMC: 2,638 cells and 13,714 genes) to 37.3 hours on the largest dataset (heart: 287,269 cells and 33,694 genes). For the same brain dataset, the TRRUST network (the smallest in size: 2,862 genes and 8,427 interactions) takes 0.28 hours, while the BioGRID network (the largest in size: 19,752 genes and 977,356 interactions) takes 7.6 hours.”

#6. The performance of scGraph2Vec on different types of gene networks, such as metabolic or signaling networks, should be evaluated to test its universality. Discuss

Powered by Editorial Manager® and ProduXion Manager® from Aries Systems Corporation

	whether the model can be easily adapted to handle different types of omics data, such as proteomics or metabolomics.<br><br>Response: We thank the reviewer for this valuable suggestion. During the revision process, we applied scGraph2Vec to three widely used gene networks: the BioGRID network representing physical interactions, the Reactome network representing biological pathways, and the TRRUST network representing transcriptional regulatory relationships. We tested these on single-cell datasets from the brain and PBMC. The results demonstrated that scGraph2Vec can distinguish gene clusters regardless of the biological network type (Supplementary Fig. S5). Additionally, we assessed how different network features influenced gene clustering in the two-dimensional embedding space. By comparing gene clusters composed of hub genes with varying centrality levels and their neighboring genes, we found that scGraph2Vec effectively captures small, locally connected clusters, making it suitable for a range of biological networks (Supplementary Fig. S6-S7). In summary, scGraph2Vec can be broadly applied across different network types, including various omics data that provide clear gene-gene interaction relationships, such as proteomes and metabolomes.<br><br>#7. The visualizations provided are crucial for understanding the model's performance. The authors are advised to include additional visualizations to help readers more intuitively grasp the biological characteristics and differences of gene modules.<br><br>Response: We appreciate the reviewer's valuable suggestion regarding the visualizations. To address this, we included additional visualizations in the revised manuscript that highlight the biological characteristics and differences of the identified gene clusters. Specifically, we added Figure 2, and Supplementary Figures 9 and 10 to compare the cluster partitioning capabilities, gene cluster similarities, and overlap with known functional gene sets between scGraph2Vec and other competing algorithms. Supplementary Figures 6 and 7 were also included to assess how different characteristics of biological networks impact cluster partitioning. Additionally, we performed further functional annotation and pathway analysis for the identified disease-related genes (Fig. 5F, G and 6F, G). Furthermore, we introduced a case study applying scGraph2Vec to melanoma cell lines in different cell states, demonstrating its potential in uncovering cell state-related regulatory genes (Fig. 8).<br><br>#8. The discussion section should delve deeper into the advantages and limitations of scGraph2Vec compared to existing methods and its potential impact in practical applications.<br><br>Response: We appreciate the reviewer's insightful suggestion. In the revised manuscript, we expanded the discussion section to provide a more in-depth comparison of the advantages and limitations of scGraph2Vec relative to existing methods and its potential impact in practical applications.<br><br>References<br>1.Langfelder P and Horvath S. WGCNA: an R package for weighted correlation network analysis. BMC Bioinformatics. 2008;9:559. doi:10.1186/1471-2105-9-559.<br>2.Tirosh I, Izar B, Prakadan SM, Wadsworth MH, 2nd, Treacy D, Trombetta JJ, et al. Dissecting the multicellular ecosystem of metastatic melanoma by single-cell RNA-seq. Science (New York, NY). 2016;352 6282:189-96. doi:10.1126/science.aad0501.
**Additional Information:**	
**Question**	**Response**
Are you submitting this manuscript to a special series or article collection?	No
**Experimental design and statistics**	Yes

Powered by Editorial Manager® and ProduXion Manager® from Aries Systems Corporation

Full details of the experimental design and statistical methods used should be given in the Methods section, as detailed in our Minimum Standards Reporting Checklist. Information essential to interpreting the data presented should be made available in the figure legends.<br><br>Have you included all the information requested in your manuscript?	
**Resources**<br><br>A description of all resources used, including antibodies, cell lines, animals and software tools, with enough information to allow them to be uniquely identified, should be included in the Methods section. Authors are strongly encouraged to cite Research Resource Identifiers (RRIDs) for antibodies, model organisms and tools, where possible.<br><br>Have you included the information requested as detailed in our Minimum Standards Reporting Checklist?	Yes
**Availability of data and materials**<br><br>All datasets and code on which the conclusions of the paper rely must be either included in your submission or deposited in publicly available repositories (where available and ethically appropriate), referencing such data using a unique identifier in the references and in the "Availability of Data and Materials" section of your manuscript.<br><br>Have you have met the above requirement as detailed in our Minimum Standards Reporting Checklist?	Yes

Powered by Editorial Manager® and ProduXion Manager® from Aries Systems Corporation

Manuscript

Click here to access/download;Manuscript;scGraph2Vec.GigaScience.R1.fina

Response to the comments of the editor and reviewers

Manuscript ID: GIGA-D-24-00200

Title: scGraph2Vec: a deep generative model for gene embedding augmented by Graph Neural Network and single-cell omics data

We appreciate very much the valuable comments of the editor and reviewers. Each of these comments has been considered in the revised manuscript or is discussed below. In response to the reviewers' comments, we have made substantial changes to our manuscript, including addressing specific issues raised and providing additional clarification where needed. Briefly, we conducted the following analyses during revision:

1. Substantially expand the comparison with other methods. In total, we benchmarked 15 methods, including 6 newly added during revision per the request by the reviewers. The results showed that scGraph2Vec produced gene embeddings that distinguished different gene clusters more clearly compared to other embedding algorithms. Moreover, the clusters by scGraph2Vec contained a higher proportion of genes with known functions.

2. We included two more reference networks to validate scGraph2Vec. In total, we evaluated scGraph2Vec using three common biological networks, each representing distinct network properties: protein-protein interaction (PPI) network, transcriptional regulatory network, and metabolic pathway network. The results indicated that scGraph2Vec is highly effective at capturing small, locally connected clusters, making it particularly suited to scale-free networks characterized by sparse connections, a feature common to most biological interaction networks.

3. For the gene embeddings, clusters, and candidate genes identified by scGraph2Vec, we included more functional annotations to demonstrate their biological relevance, including network and pathway analysis, benchmark with the hallmark genes and housekeeping genes, and comparison with cell-type and tissue-specific genes.

4. We added more application cases to demonstrate scGraph2Vec. Specifically, we inferred the regulatory genes involved in melanoma cell state transitions.

The following is an outline of our point-to-point responses to editor and reviewers' criticisms.

Response to the editor:

Comment: Overall, the reviewers felt that the content of this manuscript requires further enhancement, particularly in elucidating the novel biological insights or advantages offered by the gene embeddings generated by this model compared to those gene modules derived from other state-of-the-art algorithms. In addition, more benchmarking is required against recent methods, especially those with novel algorithmic approaches to network analysis.

In addition, please register any new software application in the bio.tools and SciCrunch.org databases to receive RRID (Research Resource Identification Initiative ID) and biotoolsID identifiers, and include these in your manuscript. Computational workflows should be registered in workflowhub.eu and the DOIs cited in the relevant places in the manuscript. These will facilitate tracking, reproducibility and re-use of your tool.

We thank the editor for the valuable comments. We have registered scGraph2Vec in both bio.tools and SciCrunch.org databases, and include the RRID (SCR_025322) and biotoolsID (scGraph2Vec) identifiers in our manuscript.

Response to Referee #1:

The main contribution of this study lies in the proposal of a new model, scGraph2Vec, which leverages the powerful representational capabilities of Graph Neural Networks (GNNs) to capture the intricate interaction between genes, while incorporating omics information at the single-cell level. This approach facilitates a deeper understanding for researchers of gene regulation, cell type differentiation, and the molecular mechanisms underlying diseases. However, the content of this manuscript requires further enhancement, particularly in elucidating the novel biological insights or advantages offered by the gene embeddings generated by this model compared to those gene modules derived from other state-of-the-art algorithms.

Response: We thank the reviewer for the positive evaluation and suggestions of our manuscript. Following your suggestion, we compared the gene embeddings by scGraph2Vec with those derived by other state-of-the-art algorithms. In total, we compared 13 gene embedding methods published in recent years and included two additional representative gene clustering methods, SCENIC and LIGER. Additionally, we incorporated a comparison of three types of gene networks to highlight the unique advantage of scGraph2Vec in integrating gene interaction networks with single-cell omics data. We assessed the ability of each method to generate gene embeddings and gene clusters. To evaluate the biological advantages of each method, we used the MsigDB hallmark gene set as a ground truth.

The results showed that scGraph2Vec effectively combined diverse gene interaction networks with single-cell omics to generate highly informative gene embeddings. These embeddings capture both network-specific interaction features and biological context-specific expression patterns, allowing for clearer separation of gene clusters and more precise identification of co-functional gene programs.

In addition to the shortcomings in novelty and biological significance within this manuscript, here are some revision suggestions:

#1. In Fig. 2, the authors show that the gene embeddings acquired through the scGraph2Vec model achieve higher silhouette scores than those resulting from alternative algorithms. Nonetheless, they fail to expound upon the biological relevance of these gene embeddings, nor do they illustrate that the gene embeddings procured via scGraph2Vec encompass greater or more precise biological insights relative to the gene modules yielded by competing algorithms.

Response: We thank the reviewer for this valuable suggestion. During revision, we compared scGraph2Vec with 13 gene embedding methods and two representative gene clustering methods, SCENIC and LIGER. We evaluated the gene clusters using the silhouette coefficient and Davies-

Bouldin Index and assessed the similarity of gene clusters using the Jaccard index. To ensure a fair evaluation of biological relevance, we introduced the MsigDB hallmark gene sets as the ground truth to measure the functions of gene clusters. The results showed that scGraph2Vec outperformed nearly all other methods in separating gene clusters, except for siVAE, which performed better on the PBMC dataset (Fig. 2B-C and Supplementary Fig. 9). We observed significant differences in the gene clusters generated by these methods (Fig. 2D). However, when compared to the MsigDB hallmark gene sets, scGraph2Vec clustered the highest number of functionally similar genes among all competing algorithms (Fig. 2E).

Additionally, we explored the biological relevance of gene embeddings from several perspectives, including network topology, clustering of annotated gene sets, cell type specificity, and tissue specificity.

(1) Network topology. The internal closeness centrality of genes in each cluster was significantly higher than their external closeness centrality (Fig. 3A).

(2) The clustering of annotated genes. Genes with similar functions, e.g., the housekeeping genes and the hallmark genes, tend to cluster together (Fig. 3B, C and Supplementary Fig. 13).

(3) Cell type specificity. When we applied scGraph2Vec to data from six representative human tissues, gene embeddings helped us identify clusters with cell type-specific expression (Fig. 4A, B and Supplementary Fig. S15). For instance, cluster 59 was enriched in multiple excitatory and inhibitory neurons and was particularly associated with down-regulated differentially expressed genes (DEGs) in these cell types (Fig. 4A and Supplementary Fig. S15).

(4) Tissue specificity. Embeddings also captured tissue-specific functional clusters. For example, our comparison of *SMARCE1*'s neighboring genes in the brain and lung revealed distinct functions: in brain tissue, 14 neighboring genes were enriched in neuronal fate commitment and motor neuron differentiation (Fig. 4F), while in lung tissue, 20 neighboring genes were enriched in chromatin organization and histone H3-K9-related functions (Fig. 4G).

(5) Four applications to identify critical genes in different biological contexts. The four application cases, i.e., COVID-19, Alzheimer’s disease, lung adenocarcinoma, and melanoma, illustrated that scGraph2Vec can be widely used in various scenarios and identify candidate genes critical to the biological conditions, be it disease-associated genes, cancer driver genes, or regulatory genes for cell states.

In summary, scGraph2Vec integrates various gene networks and single-cell omics data from diverse biological backgrounds to generate highly informative gene embeddings. These embeddings capture both the topological characteristics of biological networks and biologically significant gene clusters, uncovering context-specific gene regulatory programs.

#2: In Fig. 2B, the authors indicated that "housekeeping genes tended to cluster together and were enriched in a few gene modules". Does this suggest that these gene modules are related to cell maintenance functions or states?

Response: We thank the reviewer for pointing this out. We extracted gene clusters containing more

than 20 housekeeping genes in the six normal human tissues and conducted functional analysis (Table 1 below). Interestingly, in all tissues, the clusters with the largest number of housekeeping genes were all enriched with cytoplasmic translation and ribosome biogenesis, indicating that these clusters were involved in the basic functions of cell maintenance.

Table 1. The GO enrichment results for top gene clusters containing more than 20 housekeeping genes.

**Tissue**	**Cluster ID**	**#HK genes***	**Enriched functions#**
**Brain**	9	59	cytoplasmic translation ($P_{BH} = 1.02 \times 10^{-8}$)<br>ribosome biogenesis ($P_{BH} = 8.75 \times 10^{-8}$)
**Lung**	5	57	cytoplasmic translation ($P_{BH} = 1.20 \times 10^{-97}$)<br>ribosome biogenesis ($P_{BH} = 1.82 \times 10^{-55}$)
**Lung**	111	30	proteasome-mediated ubiquitin-dependent protein catabolic process ($P_{BH} = 2.28 \times 10^{-10}$)<br>positive regulation of establishment of protein localization to telomere ($P_{BH} = 8.56 \times 10^{-8}$)
**Lung**	85	27	cellular respiration ($P_{BH} = 3.59 \times 10^{-26}$)<br>ribose phosphate metabolic process ($P_{BH} = 4.47 \times 10^{-26}$)
**PBMC**	4	57	cytoplasmic translation ($P_{BH} = 1.03 \times 10^{-99}$)<br>ribosome biogenesis ($P_{BH} = 3.31 \times 10^{-63}$)
**PBMC**	101	27	proteasome-mediated ubiquitin-dependent protein catabolic process ($P_{BH} = 2.98 \times 10^{-18}$)<br>positive regulation of establishment of protein localization to telomere ($P_{BH} = 5.31 \times 10^{-9}$)
**PBMC**	49	21	ribose phosphate metabolic process ($P_{BH} = 6.11 \times 10^{-10}$)<br>purine nucleotide metabolic process ($P_{BH} = 6.11 \times 10^{-10}$)
**Liver**	51	49	cytoplasmic translation ($P_{BH} = 1.11 \times 10^{-99}$)<br>ribosome biogenesis ($P_{BH} = 2.99 \times 10^{-30}$)
**Liver**	55	21	cellular respiration ($P_{BH} = 1.16 \times 10^{-107}$)<br>energy derivation by oxidation of organic compounds ($P_{BH} = 2.43 \times 10^{-95}$)
**Heart**	100	52	cytoplasmic translation ($P_{BH} = 2.86 \times 10^{-84}$)<br>ribosome biogenesis ($P_{BH} = 2.77 \times 10^{-62}$)
**Heart**	114	26	proteasome-mediated ubiquitin-dependent protein catabolic process ($P_{BH} = 1.27 \times 10^{-18}$)<br>regulation of protein catabolic process ($P_{BH} = 1.95 \times 10^{-4}$)
**Heart**	94	21	cellular respiration ($P_{BH} = 1.79 \times 10^{-114}$)<br>oxidative phosphorylation ($P_{BH} = 1.79 \times 10^{-101}$)

**Kidney**	104	44	cytoplasmic translation ($P_{BH} = 5.37 \times 10^{-64}$)<br>ribosome biogenesis ($P_{BH} = 6.31 \times 10^{-63}$)
**Kidney**	133	25	integrator complex ($P_{BH} = 8.59 \times 10^{-4}$)<br>transcription factor TFIIH core complex ($P_{BH} = 1.04 \times 10^{-3}$)

* HK genes: housekeeping genes.

# To save space, only the two best enriched GO terms are listed for each cluster. P_{BH}: Benjamini-Hochberg (BH) adjusted *P*.

#3: The authors used module score to represent the overall expression pattern of module genes, is there mathematical proof for this? Is there biological evidence to support this claim? Is there a ground truth available to validate its reliability?

Response: We thank the reviewer for these valuable suggestions. In the first submission, we used the module eigengene to calculate the module score. This procedure followed the Weighted correlation network analysis (WGCNA) algorithm and had been well-validated in the original WGCNA study as well as in many studies citing WGCNA [1]. However, during revision, we found that the "AddModuleScore" function in the Seurat software was more frequently used in scRNA-seq data analyses, especially to summarize the expression patterns of gene sets in different cell types [2]. Thus, in the revised manuscript, we updated our analysis strategy and used the "AddModuleScore" to calculate module scores.

#4: In Fig. 4, the authors performed a GO-term analysis of the gene modules obtained from the scGraph2Vec model, and utilized the functions of these modules to investigate the functionalities of various cell types. What are the advantages of this approach compared to directly using differentially expressed genes (DEGs), or gene modules obtained from other algorithms?

Response: We thank the reviewer for this valuable suggestion. scGraph2Vec aims to identify gene clusters with coordinated expression patterns across cells. It does not use predefined cell type information during training. The relative distances among genes in the two-dimensional embedding space reflect their biological relationships, capturing not only cell type-specific expression patterns but also the topological characteristics of the gene interaction network. In contrast, directly using DEGs focuses on comparisons between cell types but does not consider the coordinated gene expression across multiple cells. In case studies such as COVID-19, Alzheimer's disease (AD), lung adenocarcinoma (LUAD), and melanoma, the density-based search algorithm allowed us to identify genes associated with candidate disease genes within the embedding space. This highlights the unique strengths of gene embeddings in uncovering additional disease-associated genes and revealing regulatory genes specific to different biological contexts.

#5: In studies on COVID-19 and Alzheimer's disease, the authors utilized disease-related variants identified through GWAS analysis, combined with information from gene modules, to screen for genes associated with these diseases. How did the authors determine that these variants in the

genes would lead to changes in the RNA expression levels of these genes? Was it because these variant sites were located in promoter or enhancer regions of these genes? Is there any experimental evidence for this?

Response: We thank the reviewer for pointing this out. We used the MAGMA software to calculate gene-based p-values and defined seed genes by considering the study-wide significant level based on the Bonferroni correction. MAGMA maps SNPs to genes based on genomic locations, assigning an SNP to a gene if the SNP's location falls inside the gene region. In addition, SNPs near a gene but outside its transcription region can be mapped to that gene using a pre-defined window. We used the default settings by MAGMA, i.e., "--annotate window=5,1.5", where SNPs located 5kb upstream and 1.5kb downstream were also mapped to a gene. Then, MAGMA implemented a multiple linear principal components regression model and an F-test to compute the gene p-values. The MAGMA pipeline has been widely used in various GWAS analysis applications.

#6: Some paragraphs and sentences in this manuscript have rather stiff language expression. The authors can use English proofreading service to prevent grammatical and semantic errors in expression.

Response: Thank you for your valuable feedback. During revision, we have carefully revised the text to improve clarity and readability. Additionally, we have considered professional English proofreading services to further enhance the manuscript and ensure that it meets the highest linguistic standards. We appreciate your suggestion and will make every effort to improve the overall quality of the writing.

Response to Referee #2:

The authors have proposed a computational method called 'scGraph2Vec' that takes a single-cell gene expression profile and a gene interaction network as input, and produces low-dimensional gene embedding as output. The links to the used datasets are either provided in the Supplementary Table S1 or mentioned in the main text. The source code has been made publicly available at https://github.com/LPH-BIG/scGraph2Vec. I highly appreciate the effort the authors have made to improve the reproducibility of their work. It would be great if the authors could address the following comments to strengthen the publication.

Response: We thank the reviewer for the positive comments on our manuscript. Each of these comments has been considered in the revised manuscript and is discussed below.

#1: The background section needs to be significantly improved. It has been written that scGraph2Vec "extends the VGAE framework". However, VGAE is a data-agnostic framework; scGraph2Vec rather utilises the VGAE framework for analysing gene expression and gene interaction network data. Therefore, the authors need to incorporate a critical review of the recent method that do the same. I am listing a few references I could find for that purpose:

https://academic.oup.com/nar/article/51/13/6578/7184155
https://isip.piconepress.com/conferences/ieee_spmb/2022/papers/p01_07.pdf
http://dr.iiserpune.ac.in:8080/xmlui/handle/123456789/7885
https://arxiv.org/abs/1802.04407
There are more references that I could not list here. I request the authors to enhance the background section by reviewing such methods.

Response: Thank you for your insightful comments. In the revised manuscript, we have reinforced a critical review of recent VGAE-based embedding methods in the background section.

#2: In addition to the critically reviewing other VGAE-based gene embedding methods, I request the authors to include them in the comparisons based on the benchmark datasets.

Response: We thank the reviewer for this valuable suggestion. Following your suggestion, during the revision process, we compared scGraph2Vec with four VGAE-based embedding methods and nine single-cell embedding methods.

Notably, we were unable to run the two methods cited in the first and second references by the due date of this revision. The first method, GenKI, was designed to capture shifting patterns in gene regulation caused by knockout (KO) perturbations. In our attempt, GenKI took days to construct the gene networks. Even after we reduced the genes to 1,865 highly variable genes in PBMC and 5,182 in the brain, we still could not obtain results using 60 CPU cores after a month. For the second reference, we were unable to test the method as its source code was unavailable.

Thus, we focused on the other VGAE-based embedding methods, including the highly cited SIG-VAE and DGVAE. SIG-VAE extends VGAE's flexibility by using a hierarchical variational framework and a Bernoulli-Poisson decoder, allowing for better modeling of complex graph dependencies and implicit posteriors. DGVAE, on the other hand, introduces graph cluster memberships as latent factors, demonstrating strong performance in graph generation and clustering tasks. In total, we benchmarked 13 VGAE-based embedding methods (ARGA, ARGVA, SIG-VAE, DGVAE, scVI, LDVAE, siVAE, scVAE, scGNN, scapGNN, scETM, SAUCIE and SIMBA) against scGraph2Vec.

We systematically evaluated the ability of different methods to divide clusters, the similarity of gene clusters among methods, and the overlap of each cluster with the MsigDB hallmark gene sets. The results showed that scGraph2Vec could divide different gene clusters more clearly than other methods, except siVAE on the PBMC dataset (Fig. 2B-C). We found that the gene clusters identified by different methods were quite different (Fig. D). However, when compared with the MsigDB hallmark gene sets, the clusters by scGraph2Vec tended to have higher proportions of the hallmark genes, i.e., genes with common functions, than other competing algorithms (Fig. 2E). These results indicated that the clusters by scGraph2Vec tended to be more functionally correlated.

#3: The authors have utilized the gene embedding outputs of scGraph2Vec for clustering genes.

Therefore, I request the authors to compare the resultant gene clusters with that of other single-cell gene expression based gene clustering methods such as SCENIC (https://www.nature.com/articles/nmeth.4463) and LIGER (https://www.cell.com/cell/pdf/S0092-8674(19)30504-5.pdf).

Response: We thank the reviewer for bringing this point up. SCENIC and LIGER generate gene clusters but do not generate gene embeddings. Thus, we applied SCENIC and LIGER using the same brain and lung datasets as we used in scGraph2Vec and generated gene clusters. Then we compared the gene clusters identified by SCENIC and LIGER in the gene embedding space identified by scGraph2Vec. Our analysis revealed that SCENIC and LIGER gene clusters were not closely distributed in our embedding space and did not overlap significantly with our scGraph2Vec clusters (Supplementary Fig. 10). By assessing the similarity of the gene clusters (by SCENIC, LIGER, and scGraph2Vec, respectively) with the MsigDB hallmark gene set, we found that the gene clusters by scGraph2Vec showed a higher proportion of the hallmark genes (Fig. 2E). Thus, we consider that scGraph2Vec can generate gene clusters more functionally correlated.

#4: The authors have used the silhouette index to compare the gene clusters produced by different methods. I would request the authors to incorporate additional evaluation metrics such as the Davies-Bouldin index (DBI) and Jaccard index.

Response: We thank the reviewer for raising this important point. During revision, we have included the Davies-Bouldin Index and the Jaccard Index to compare the gene clusters (Fig. 2).

Response to Referee #3:

The manuscript introduces a novel deep-learning framework designed to generate informative gene embeddings. This framework, scGraph2Vec, extends the variational graph autoencoder (VGAE) model and integrates single-cell data patterns along with gene interaction networks. The model's effectiveness is demonstrated through its ability to produce biologically interpretable gene embeddings that reveal functional modules of genes, which can be applied to identify disease-associated genes and understand tissue-specific cellular processes.

Response: We thank the reviewer for the positive comments on our manuscript.

However, the authors still have the following problems to solve:

#1. The manuscript compares scGraph2Vec with several existing gene embedding methods. It would be beneficial to include more recent methods, especially those with novel algorithmic approaches to network analysis.

Response: We thank the reviewer for this valuable suggestion. During the revision process, we systematically reviewed existing gene embedding methods published in recent years, especially those with novel algorithmic approaches to network analysis. In total, we selected 13 embedding

methods (ARGA, ARGVA, SIG-VAE, DGVAE, scVI, LDVAE, siVAE, scVAE, scGNN, scapGNN, scETM, SAUCIE, and SIMBA) and 2 gene clustering methods (SCENC and LIGER) for comparison with scGraph2Vec.

All methods generated gene clusters. We employed three measurements to assess the gene clusters, i.e., the silhouette coefficient, the Davies-Bouldin Index, and the overrepresentation status of each cluster with the hallmark gene sets. The silhouette coefficient and the Davies-Bouldin Index measure how the clusters generated by each method can be clearly distinguished. As shown in Fig.2B-C in the revised manuscript, the clusters by scGraph2Vec outperformed nearly all other methods in the brain and PBMC tissues. The overrepresentation status of the hallmark genes measures the functional convergence of each cluster. As shown in Fig. 2E in the revised manuscript, scGraph2Vec generated gene clusters that shared the most with the hallmark genes, indicating that the clusters by scGraph2Vec were functionally convergent (Fig. 2E).

#2. The authors have used several datasets to validate the performance of scGraph2Vec. To further demonstrate the model's generalizability and robustness, testing on a broader range of datasets is recommended, especially those from different biological contexts or species.

Response: We thank the reviewer for this valuable suggestion. During the revision process, we included an additional application case by using melanoma cell lines with different states. In total, we have four applications representing three unique contexts (Table 2 below). We hope these applications are satisfactory to answer your question.

Table 2. Application cases of scGraph2Vec.

**Case**	**Seed gene**	**Embedding dataset**	**Validation dataset**	**Validation method**
COVID-19	GWAS	Normal lung	Bulk RNA-seq from disease and healthy individuals	DEG
AD	GWAS	Normal brain	Bulk RNA-seq from disease and healthy individuals	DEG
LUAD	Known driver genes	Normal and tumor samples	Bulk RNA-seq from disease and healthy individuals (TCGA)	DEG and survival analysis
Melanoma	*SOX10*	Cell lines in 3 states	scRNA-seq and bulk RNA-seq from normal and *SOX10*-KD samples	DEG and AUCell scores

#3. While the gene embeddings generated by scGraph2Vec are said to be biologically interpretable, the authors should further explore how these embeddings correspond to known biological processes and disease mechanisms. For the identified disease-associated genes, a more profound

functional annotation and pathway analysis are suggested to uncover their specific roles in disease progression.

Response: We thank the reviewer for this valuable suggestion. To explore the biological relevance of gene embeddings, we conducted the following analyses.

(1) Network topology. The internal closeness centrality of genes in each cluster was significantly higher than their external closeness centrality (Fig. 3A).

(2) The clustering of annotated genes. Genes with similar functions, e.g., the housekeeping genes and the hallmark genes, tend to cluster together (Fig. 3B, C and Supplementary Fig. 13).

(3) Cell type specificity. When we applied scGraph2Vec to data from six representative human tissues, gene embeddings helped us identify clusters with cell type-specific expression (Fig. 4A, B and Supplementary Fig. S15). For instance, cluster 59 was enriched in multiple excitatory and inhibitory neurons and was particularly associated with down-regulated differentially expressed genes (DEGs) in these cell types (Fig. 4A and Supplementary Fig. S15).

(4) Tissue specificity. Embeddings also captured tissue-specific functional clusters. For example, our comparison of *SMARCE1*'s neighboring genes in the brain and lung revealed distinct functions: in brain tissue, 14 neighboring genes were enriched in neuronal fate commitment and motor neuron differentiation (Fig. 4F), while in lung tissue, 20 neighboring genes were enriched in chromatin organization and histone H3-K9-related functions (Fig. 4G).

(5) Four applications to identify critical genes in different biological contexts. The four application cases, i.e., COVID-19, Alzheimer's disease, lung adenocarcinoma, and melanoma, illustrated that scGraph2Vec can be widely used in various scenarios and identify candidate genes critical to the biological conditions, be it disease-associated genes, cancer driver genes, or regulatory genes for cell states.

In summary, scGraph2Vec integrates various gene networks and single-cell omics data from diverse biological backgrounds to generate highly informative gene embeddings. These embeddings capture both the topological characteristics of biological networks and biologically significant gene clusters, uncovering context-specific gene regulatory programs.

For the identified disease-related genes, we conducted in-depth functional annotation and pathway analysis. Based on pathway databases such as KEGG, WikiPathways, and Reactome, we applied the R packages clusterProfiler (v.4.6.2) and ReactomePA (v.1.42.0) to perform pathway enrichment analysis. Pathway annotation was done with ClueGO (v.2.5.10). Protein-protein interaction analysis was carried out using STRING. In COVID-19, pathway enrichment analysis revealed significant enrichment in Glycosaminoglycan degradation ($P_{\mathrm{BH}} = 3.08 \times 10^{-4}$), type I interferon induction and signaling during SARS-CoV-2 infection ($P_{\mathrm{BH}} = 6.46 \times 10^{-4}$), Interleukin-10 signaling ($P_{\mathrm{BH}} = 3.03 \times 10^{-3}$), and ABO blood group biosynthesis ($P_{\mathrm{BH}} = 3.29 \times 10^{-3}$) (Fig. 5F). STRING analysis further demonstrated protein-protein interactions among genes involved in these pathways (Fig. 5G). In AD, our pathway annotation of 362 neighboring genes further revealed regulatory pathways involved in AD, mainly complex I biogenesis ($P_{\mathrm{BH}} = 9.00 \times 10^{-10}$), cholesterol

transport ($P_{BH} = 1.07 \times 10^{-4}$), regulation of cholesterol transport ($P_{BH} = 9.48 \times 10^{-4}$), regulation of amyloid-beta clearance ($P_{BH} = 9.83 \times 10^{-5}$) and oxidative phosphorylation ($P_{BH} = 3.67 \times 10^{-9}$) (Fig. 6F, G).

#4. The manuscript mentions the optimization of model parameters through hyperparameter sweeping. The authors should provide more details on this process, including the specific ranges and tuning strategies used.

Response: We apologize for not providing the details of the hyperparameter sweeping process. In the revised manuscript, we added a detailed description of the hyperparameter selection and optimization process in the Methods section. Specifically, we considered standard VGAE parameters, i.e., learning rate, training iterations, encoding layers, and their dimensions, and new parameters that were specific to scGraph2Vec, i.e., λ, s, β, and γ. We tested λ (0.001, 0.1, 1), β (0.5, 5, 10), γ (0.1, 1), and s (2, 10). The optimal hyperparameters were determined by combining evaluation metrics, prioritizing a higher silhouette coefficient, a greater number of clusters, and a shorter running time. As a result, the optimal settings were $\lambda = 1, \beta = 10, \gamma = 0.1, s = 10$. Subsequently, we tested a two-layer encoder with dimensions 64-16, 128-16, 256-16, 128-32, and 256-32, as well as a three-layer encoder with dimensions 256-64-16 and 512-64-16. Finally, we recommended using a three-layer neural network (256-64-16) encoder. The Adam optimizer strategy and a learning rate = 1×10^{-4} were adopted. A maximum of 600 iterations and early stopping with patience = 50 was used. The model training was repeated 10 times in each tissue.

#5. For large-scale datasets, how efficient is scGraph2Vec in terms of computational resources? The authors are encouraged to assess and report the model's runtime and resource consumption, especially when dealing with large single-cell datasets.

Response: We apologize for not providing details about running processes. In the revised manuscript, we have added information about the computational resources. On page 20, in the last paragraph, we added:

"We used 64 logical CPUs and 512 GB RAM for all experiments. The execution time of scGraph2Vec depends on the size of the single-cell dataset and the interaction network. It ranges from 6.5 hours on the smallest dataset (PBMC: 2,638 cells and 13,714 genes) to 37.3 hours on the largest dataset (heart: 287,269 cells and 33,694 genes). For the same brain dataset, the TRRUST network (the smallest in size: 2,862 genes and 8,427 interactions) takes 0.28 hours, while the BioGRID network (the largest in size: 19,752 genes and 977,356 interactions) takes 7.6 hours."

#6. The performance of scGraph2Vec on different types of gene networks, such as metabolic or signaling networks, should be evaluated to test its universality. Discuss whether the model can be easily adapted to handle different types of omics data, such as proteomics or metabolomics.

Response: We thank the reviewer for this valuable suggestion. During the revision process, we applied scGraph2Vec to three widely used gene networks: the BioGRID network representing physical interactions, the Reactome network representing biological pathways, and the TRRUST network representing transcriptional regulatory relationships. We tested these on single-cell datasets from the brain and PBMC. The results demonstrated that scGraph2Vec can distinguish gene clusters regardless of the biological network type (Supplementary Fig. S5). Additionally, we assessed how different network features influenced gene clustering in the two-dimensional embedding space. By comparing gene clusters composed of hub genes with varying centrality levels and their neighboring genes, we found that scGraph2Vec effectively captures small, locally connected clusters, making it suitable for a range of biological networks (Supplementary Fig. S6-S7). In summary, scGraph2Vec can be broadly applied across different network types, including various omics data that provide clear gene-gene interaction relationships, such as proteomes and metabolomes.

#7. The visualizations provided are crucial for understanding the model's performance. The authors are advised to include additional visualizations to help readers more intuitively grasp the biological characteristics and differences of gene modules.

Response: We appreciate the reviewer's valuable suggestion regarding the visualizations. To address this, we included additional visualizations in the revised manuscript that highlight the biological characteristics and differences of the identified gene clusters. Specifically, we added Figure 2, and Supplementary Figures 9 and 10 to compare the cluster partitioning capabilities, gene cluster similarities, and overlap with known functional gene sets between scGraph2Vec and other competing algorithms. Supplementary Figures 6 and 7 were also included to assess how different characteristics of biological networks impact cluster partitioning. Additionally, we performed further functional annotation and pathway analysis for the identified disease-related genes (Fig. 5F, G and 6F, G). Furthermore, we introduced a case study applying scGraph2Vec to melanoma cell lines in different cell states, demonstrating its potential in uncovering cell state-related regulatory genes (Fig. 8).

#8. The discussion section should delve deeper into the advantages and limitations of scGraph2Vec compared to existing methods and its potential impact in practical applications.

Response: We appreciate the reviewer's insightful suggestion. In the revised manuscript, we expanded the discussion section to provide a more in-depth comparison of the advantages and limitations of scGraph2Vec relative to existing methods and its potential impact in practical applications.

References

1. Langfelder P and Horvath S. WGCNA: an R package for weighted correlation network analysis. BMC Bioinformatics. 2008;9:559. doi:10.1186/1471-2105-9-559.

2. Tirosh I, Izar B, Prakadan SM, Wadsworth MH, 2nd, Treacy D, Trombetta JJ, et al. Dissecting the multicellular ecosystem of metastatic melanoma by single-cell RNA-seq. Science (New York, NY). 2016;352 6282:189-96. doi:10.1126/science.aad0501.

scGraph2Vec: a deep generative model for gene embedding augmented by Graph Neural Network and single-cell omics data

Shiqi Lin[1,2,3], Peilin Jia[1,2,3,*]

[1]National Genomics Data Center, China National Center for Bioinformation, Beijing, 100101, China

[2]Beijing Institute of Genomics, Chinese Academy of Sciences, Beijing, 100101, China

[3]University of Chinese Academy of Sciences, Beijing, 100049, China

[*]Address correspondence to:

Peilin Jia, Ph.D.

Email: pjia@big.ac.cn

ORCID

Shiqi Lin: 0000-0001-9518-2027

Peilin Jia: 0000-0003-4523-4153

Running title

scGraph2Vec: a deep generative model for gene embedding

ABSTRACT

Background

Exploring the cellular processes of genes from the aspects of biological networks is of great interest to understanding the properties of complex diseases and biological systems. Biological networks, such as protein-protein interaction networks and gene regulatory networks, provide insights into the molecular basis of cellular processes and often form functional clusters in different tissue and disease contexts.

Results

We present scGraph2Vec, a deep-learning framework for generating informative gene embeddings. scGraph2Vec extends the variational graph autoencoder framework and integrates single-cell datasets and gene-gene interaction networks. We demonstrate that the gene embeddings are biologically interpretable and enable the identification of gene clusters representing functional or tissue-specific cellular processes. By comparing similar tools, we showed that scGraph2Vec clearly distinguished different gene clusters and aggregated more biologically functional genes. scGraph2Vec can be widely applied in diverse biological contexts. We illustrated that the embeddings generated by scGraph2Vec can infer disease-associated genes from genome-wide association study data (e.g., COVID-19 and Alzheimer's Disease), identify additional driver genes in lung adenocarcinoma, and identify regulatory genes responsible for maintaining or transitioning melanoma cell states.

Conclusions

scGraph2Vec not only reconstructs tissue-specific gene networks but also obtains a latent representation of genes implying their biological functions.

Keywords: gene embedding, gene regulatory network, single-cell RNA-seq, tissue specificity, complex disease

BACKGROUND

Our understanding of the molecular property and regulatory mechanism is highly incomplete, especially at the cell type and tissue resolution. The common and tissue-specific processes are often controlled by different gene regulatory programs, which alter the expression of genes in different biological conditions [3, 4]. Comparison of gene regulatory networks in different tissues shows that the edges of the network (e.g., the links between transcription factors to target genes) have higher tissue-specificity than the nodes of the network (e.g., genes) because the links among genes are heavily regulated by their functional roles and the tissue contexts [3]. In the disease environment, the topology of molecular networks helps identify novel genes and pathways associated with diseases [5].

In recent years, huge amounts of omics data from different human tissues and organs have been accumulated [6-9]. Many methods have been developed to decode the dynamic regulatory links among genes [10]. Network embeddings hold substantial promise for analyzing gene regulatory programs under various conditions. For example, the Set2Gaussian [11] method identified the gene set embeddings based on the topology of the protein-protein interaction (PPI) network and used the resultant embeddings for tumor stratification and clinical prognosis. Methods such as SAUCIE [12], scVI [13], LDVAE [14], scGNN [15], and scVAE [16] used deep neural networks to aggregate and represent cell type-specific gene regulatory signals from single-cell transcriptome data, enabling highly accurate downstream analysis. Additionally, scETM [17], siVAE [18], and SIMBA [19] demonstrated the broad potential of learning cell and gene embeddings simultaneously for studying cell heterogeneity, identifying gene expression features,

and integrating omics data. Furthermore, scapGNN [20] inferred stable gene-cell association networks from sparse single-cell profile data.

Deep learning approaches have proved advantageous in many human genetics and genomics studies [10]. Variational graph autoencoder (VGAE) [21] is a type of Graph Neural Network (GNN) designed to efficiently learn latent representations of graph-structured data, enabling tasks like link prediction and node clustering by leveraging both graph topology and node features [22, 23]. Given the strong ability of VGAE to generate graph embeddings, several methods have been developed based on this framework, including ARGA [24], ARGVA [24], SIG-VAE [25], and DGVAE [26], among others. ARGA and ARGVA [24] incorporate an adversarial regularized embedding framework to improve the efficiency in handling real-world graph data. SIG-VAE [25] enhances VGAE's flexibility using a hierarchical variational framework, enabling it to capture graph dependency structure and produce more interpretable latent representations. DGVAE [26] introduces graph cluster memberships as latent factors and a new GNN variant, leading to better performance in graph generation and clustering tasks. These methods demonstrated the adaptability and interpretability of VGAE for diverse real-world graph modeling challenges.

In this work, we developed an extended VGAE approach [21, 27], namely scGraph2Vec, that integrates single-cell datasets with gene-gene interaction networks to generate highly informative gene embeddings. We demonstrated that the resultant gene embeddings recapitulate high-dimensional biological information derived from the structures of gene-gene interaction networks and gene expression patterns across cells. Compared to 13 existing embedding tools,

scGraph2Vec demonstrated promising performance in gene embedding and cluster prediction on benchmark scRNA-seq datasets. These gene embeddings help us to understand the functional clusters of genes, elucidate the influence of regulatory genes on biological processes in specific tissue environments, and infer more disease-related genes to explain disease risk. In summary, scGraph2Vec can be used on a wide range of gene networks and single-cell datasets for different biological problems.

METHODS

Data collection and preprocessing

We collected the scRNA-seq data from six healthy human tissues, which are brain [28], heart [29], kidney [30], liver [31], lung [32], and peripheral blood mononuclear cells (PBMC) [33] (Supplementary Table S1). We also obtained paired scATAC-seq and scRNA-seq data from four mid-gestation human cerebral cortex samples (GEO accession ID: GSE162170) [34]. The lung adenocarcinoma (LUAD) tumor tissues and their distal non-malignant lung tissues were downloaded from EBI ArrayExpress (accession ID: E-MTAB-6149 and E-MTAB-6653) [35]. The scRNA-seq and bulk RNA-seq datasets from human melanoma cell lines with and without perturbations (*SOX10* knockdown) were downloaded from Scope (Scope session: Wouters_Human_Melanoma) and GEO (accession ID: GSE134432) [36]. For each dataset, we filtered for genes with a non-zero expression value in at least 3 cells and for cells with at least 200 expressed genes. The originally downloaded count data were transformed to log (counts per million + 1) values and scaled by all cells.

The interaction network was downloaded from the BioGRID database (release v.4.4.210) [37], which contained 977,356 interactions among 19,752 genes. We also extracted the Reactome [38] dataset from PathwayCommons (v14, https://download.baderlab.org/PathwayCommons/PC2/v14/pathways.txt.gz) [39] to construct interaction networks that characterize biological pathways such as signaling or metabolism, which contained 326,439 interactions among 10,800 genes. The transcriptional regulatory network from the TRRUST database (v2, https://www.grnpedia.org/trrust/data/trrust_rawdata.human.tsv) [40] contained 8427 interactions among 2862 genes. To explore the impact of different network features on embedding, we divided the nodes of each network into 20 groups evenly based on degree and took the top 100 genes of each group as hub genes. The hub gene and its neighbor genes were formed into a gene cluster, and then we calculated the Hopkins statistic of each cluster in the two-dimensional embeddings to measure the aggregation of these gene clusters.

scGraph2Vec model design

scGraph2Vec takes two input matrices: the adjacency matrix A from the gene-gene interaction network and the node feature matrix X from the single-cell gene profile. We first refine A by including a node community matrix and generate an enhanced adjacency matrix A_n. scGraph2Vec directs A_n and X to go through a multi-layer GCN to generate the low-dimensional vectors Z and reconstructs the graph structure of the network through the decoder process.

Specifically, we first calculate the primary assignments of node communities by using the

Louvain greedy algorithm [41] based on the interaction network. Let $M \in \{0,1\}^{n\times k}$ represent the membership matrix, where *n* is the total number of nodes and *k* is the total number of communities. Each element in *M* indicates whether a node *i* (*i* = 1, …, n) belongs to a community C_j (*j* = 1, …, k), i.e., $M_{ij} = 1$ if $i \in C_j$ and 0 otherwise. Subsequently, the node community matrix A_c is calculated as below:

$$A_c = MM^T - I_n \tag{1}$$

where I_n is the identity matrix. Then we calculate a new adjacency matrix A_n by:

$$A_n = A + \lambda A_c \tag{2}$$

where the hyperparameter λ > 0 is introduced to balance the contribution of the original adjacency matrix *A* and the derived community matrix A_c. To alleviate the computational cost, we require that in each community C_j, each node $i \in C_j$ only connects to a pre-defined number (denoted by *s*) of nodes in C_j, instead of connecting to all nodes in C_j.Thus, *s* is a hyperparameter that can tune the sparsity of A_n.

The encoder includes multiple convolutional layers. We design two alternative structures: one with three layers (256-64-16) and the other with two layers (64-16). The encoder generates the embedding matrix *Z*:

$$Z = GCN(A_n, X) \tag{3}$$

The decoder reconstructs the connectivity structure of the network using *Z*, which relies on the inner product decoder followed by the non-linear process using the Sigmoid activation function:

$$\hat{A} = \sigma(ZZ^T) \tag{4}$$

where $\sigma(x) = \frac{1}{1+e^{-x}}$.

The optimization strategy for reconstruction typically uses the evidence lower bound (ELBO) loss L_{VGAE} to assess the similarity between the initial and the reconstructed graph structure:

$$L_{VGAE} = \mathrm{E}_{q(Z|A,X)}[\log p(A|Z,X)] - KL(q(Z|A,X)\|p(Z)) \tag{5}$$

where $KL(q(Z|A,X)\|p(Z))$ is the Kullback-Leibler divergence between $q(\cdot)$ and $p(\cdot)$. More description of L_{VGAE} can refer to the original VGAE article [21]. In scGraph2Vec, we include a complemental loss inspired by modularity for community detection. Modularity is designed to measure the strength of community structure in networks by comparing the density of connections inside and outside communities [42, 43]. To capture the global community structure, we soften the calculation of specific communities in the traditional modular formula and add a global regularizer [27]:

$$L_M = \frac{\beta}{2m} \sum_{i,j=1}^{n} \left(A_{ij} - \frac{d_i d_j}{2m} \right) e^{-\gamma \|z_i - z_j\|_2^2} \tag{6}$$

Here, the L2 distance $\|z_i - z_j\|_2^2$ is the soft counterpart of modularity which replaces the original community indicator $\delta(\mathrm{i},\mathrm{j}) \in \{0,1\}$, where $\delta(\mathrm{i},\mathrm{j}) = 1$ if nodes *i* and *j* belong to the same community and 0 otherwise. The $d_i = \sum_{j=1}^{n} A_{i,j}$ (*n* is the total number of nodes) is the degree for the ith node and 2*m* is the sum of the degrees of all nodes. Therefore, the loss function does not involve the "exact" modularity and is independent of the community information associated with nodes. The hyperparameter $\beta > 0$ balances the relative importance of the global

community structure and the pairwise node connectivity. Overall, the loss function used in scGraph2Vec is: $L = L_{VGAE} + L_M$. Our optimization goal is to maximize the graph similarity and modularity score to obtain gene embeddings.

Training details

scGraph2Vec was implemented using TensorFlow (v.1.15.0). The genes in the feature matrix were matched with those in the interaction network, where genes not annotated in the interaction network were removed and genes with missing feature values were filled with 0. The positive edges (i.e., the element values of the matrix being 1) of the initial adjacency matrix were split by 90%, 5%, and 5% and the same number of negative edges (i.e., the element values of the matrix being 0) were randomly selected to form the training set, validation set, and test set, respectively. For hyperparameter selection in scGraph2Vec, we considered standard VGAE parameters, including learning rate, training iterations, encoding layers and their dimensions, plus new parameters that were specifically designed for scGraph2Vec, i.e., λ, s, β, and γ. The optimal hyperparameters were determined by combining evaluation metrics, prioritizing a higher silhouette coefficient, a greater number of clusters, and a shorter running time. We first tested λ (0.001, 0.1, 1), β (0.5, 5, 10), γ (0.1, 1), and s (2, 10). The optimal settings were $\lambda = 1, \beta = 10, \gamma = 0.1, s = 10$. Subsequently, we tested a two-layer encoder with five possible dimensions (i.e., 64-16, 128-16, 256-16, 128-32, and 256-32), as well as a three-layer encoder with two possible dimensions (256-64-16 and 512-64-16). We tested 500, 600, and 1000 epochs, using the Adam optimizer and a learning rate of 1×10^{-4}. The model training was repeated 10 times in each

tissue.

Gene cluster identification

The 16-dimensional gene embeddings output by scGraph2Vec were reduced to a two-dimensional representation for visualization by using *t*-Distributed Stochastic Neighbor Embedding (t-SNE) [44]. Then the hierarchical clustering method was used to identify gene clusters. The total number of clusters was determined according to the silhouette coefficient. We restricted each gene cluster to contain more than 10 genes. In each replication, the output with the maximum silhouette coefficient was selected.

Benchmark with known embedding methods

We adopt a unified benchmark framework to compare scGraph2Vec with other embedding calculation methods, including ARGA [24], ARGVA [24], SIG-VAE [25], DGVAE [26], scVI [13], LDVAE [14], siVAE [18], scVAE [16], scGNN [15], scapGNN [20], scETM [17], SAUCIE [12], and SIMBA [19]. We used each method to extract the embeddings of all genes from the brain and PBMC datasets and reduced them to two dimensions for visualization through t-SNE. Hierarchical clustering was utilized to identify gene clusters. Considering the sparsity of single-cell data, all models assumed that data followed a zero-inflated negative binomial distribution. For the models within the unfixed VAE framework (including ARGA, ARGVA, SIG-VAE, DGVAE, scVAE, siVAE, scVI, and LDVAE), we configured their structures to match that of scGraph2Vec, including the same encoder-decoder layer numbers and dimensions (256-64-16).

Optimization for all models involved scanning hyperparameters without recommended values, adjusting the learning rate (1×10^{-3}, 1×10^{-4}, 1×10^{-5}) and the number of epochs (50, 100, 200, 400, 600), while other parameters were set to their defaults. Specifically, for SAUCIE, we tested parameter combinations that influenced clustering results: lambda_c (0.1, 0.2, 0.3) and lambda_d (0.5, 0.7, 0.9), and directly used the gene embeddings and clusters provided by SAUCIE. We modified the 'ConNetGNN' function of scapGNN to output gene embeddings learned from the hidden layer. For scETM, we followed the recommendation of training at least 6,000 epochs, and testing models at 6,000, 9,000, and 12,000 epochs. scGNN extracted embeddings from all genes and tested both models obtained with or without the Left Truncated Mixture Gaussian (LTMG). The final selection of the optimal model parameters was based on the convergence of the loss function.

The effectiveness of different methods in distinguishing gene clusters was evaluated using the silhouette coefficient and Davies-Bouldin index (DBI). A higher silhouette coefficient or lower DBI indicates better separation of distinct clusters. Furthermore, to estimate the biological meaning of gene clusters, we collected 50 hallmark gene sets from the MSigDB database [45, 46] as the ground truth. These gene sets represent well-defined biological states or processes. The similarity of two gene sets is measured by the Jaccard Index.

Benchmark with SCENIC and LIGER

We implemented the Python workflow pySCENIC (v.0.12.1) [47] to analyze gene clusters co-expressed with transcription factors and their potential target genes. The workflow

first builds a gene co-expression network from scRNA-seq data, then uses transcription factor binding motifs to identify clusters with regulators and remove unsupported genes. We used motif data from the cisTarget Human database v9 and ran pySCENIC with default settings. Additionally, we implemented the LIGER software (v.2.0.1) [48]. LIGER utilized integrative non-negative matrix factorization (iNMF) to identify factors that are either shared between datasets or specific to each. These factors were then used to cluster cells jointly and identify marker genes for all clusters. We applied both methods to the brain and PBMC datasets.

Estimating biological implications of gene clusters

We explored the biological implications of clusters based on topology, annotated gene sets, and expression patterns. Firstly, we examined whether gene clusters could reflect highly clustered gene sets in gene-gene interaction networks. Specifically, for each cluster, we divided all connections in the BioGRID network into intra-cluster and out-of-cluster connections. For each gene, we calculated two closeness centrality values, i.e., the internal closeness centrality based on the intra-cluster connections and the external closeness centrality based on the out-of-cluster connections. Then, we averaged these centrality values across all genes in each cluster to determine the overall internal and external closeness centrality. Wilcoxon rank-sum test was employed to compare the internal and external closeness centrality for each cluster.

For gene set enrichment analysis, we downloaded 50 hallmark gene sets [49] and curated a set of 397 housekeeping genes [50] (set name: HSIAO_HOUSEKEEPING_GENES) from the MSigDB database. Fisher's exact test was employed to identify clusters enriched for the

annotated gene sets.

For gene expression patterns, we used the “AddModuleScore” function from the Seurat software (v.4.3.0.1) [2] to calculate a score for each gene cluster.

Identification of cell-type specificity and tissue specificity

For cell-type specificity, we used the gene set enrichment analysis (GSEA) [45] to examine whether cluster genes were significantly enriched in differentially expressed genes (DEGs) across cell types. We identified DEGs for each cell type using the ‘FindAllMarkers’ function of the Seurat software [51] and ranked all genes by their average log2 (fold change). The predefined gene sets were derived from gene clusters identified by scGraph2Vec. We used the R package GSEABase (v.1.56.0) for GSEA and enrichplot (v.1.14.2) for visualization.

We then applied Fisher's exact test to identify tissue-specific clusters. Specifically, clusters significantly overlapped with other clusters from all other tissues were identified first (Bonferroni corrected $P < 0.05$). Clusters with significant overlap in less than 5% of the total clusters were considered tissue-specific.

Identification of disease-candidate genes

We developed a framework to identify novel candidate genes using the gene embeddings (Fig. 1B) [52]. The framework can be applied in various conditions. Here we demonstrated it to identify disease-candidate genes from genome-wide association studies (GWAS) data, cancer-driver genes from tumor tissue scRNA-seq data, and regulatory genes from melanoma scRNA-

seq data. In each case, scGraph2Vec was first applied to the corresponding single-cell omics data to generate gene embeddings. Next, a set of ground-truth genes was used as seed genes. Each seed gene can form a cluster if it contains more than 6 genes within a default radius of 0.5 in the embedding space. The cluster is expanded if any of its component genes has neighbor genes located within a radius of 0.5 until no further genes can be added. We excluded genes that are not direct interactors of the seed genes. Genes in the cluster are thus considered candidates for further analyses.

We demonstrated the framework to infer disease-candidate genes from GWAS summary statistics using COVID-19 and Alzheimer's Disease (AD) as examples. For COVID-19, we downloaded GWAS data from Host Genetics Initiative (HGI, release 7, access date: April 8, 2022, file name: A2_ALL_eur_leave23andme) [53], including 13,769 severe COVID-19 patients and 1,072,442 healthy individuals. For AD, we downloaded GWAS data from a study conducted using 71,880 clinically diagnosed AD or AD-by-proxy and 383,378 controls [54]. In both cases, we used the genome-wide significant genes identified by MAGMA [55] (Bonferroni correction) as seed genes. Gene embeddings were generated using the scRNA-seq data in the disease-relevant tissues, e.g., lung for COVID-19 and brain for AD. We collected bulk RNA-seq data from the disease and healthy individuals for validation [56, 57].

The framework can also be used to identify candidate driver genes (Fig. 1B). To this end, we downloaded scRNA-seq data from a non-small cell lung cancer (NSCLC) patient [35], including data from the tumor core sample and the distal normal tissue. The gene embeddings were generated using scGraph2Vec for tumor or normal, respectively. A total of 18 LUAD-driver

genes were collected from previous studies [58] and used as seed genes. To validate the candidate genes identified from our framework, we utilized the bulk RNA-seq data from The Cancer Genome Atlas (TCGA) including 541 LUAD and 59 normal controls [59]. Transcript per million (TPM) normalized gene expression data was utilized to identify DEGs using the R package DESeq2 (version 1.34.0) [60] ($|\log2(FC)| > 0.5$ and Benjamini-Hochberg (BH) adjusted P, or $P_{BH} < 0.05$). The univariate Cox proportional hazards regression analysis was applied to identify prognosis-related genes ($P < 0.05$). The Kaplan-Meier curves and the log-rank tests were implemented using the R package survival (v.3.5.1) [61].

We also demonstrated the framework to identify regulatory genes using the melanoma scRNA-seq dataset. This dataset includes nine patient-derived cultures and the A375 cell line, categorized into the stable melanocytic (MM001, MM011, MM031, A375), intermediate (MM057, MM074, MM087) and mesenchymal-like (MM029, MM047, MM099) states [36]. We applied scGraph2Vec to generate gene embeddings and gene clusters for each state. For the resultant clusters, we investigated their state-specificity following the same approach as for tissue-specific clusters and calculated AUCell scores using the AUCell R package (v.1.25.2) [62]. Furthermore, we collected gene signatures representing 14 crucial functional states of cancer cells, including stemness, invasion, metastasis, proliferation, epithelial-mesenchymal transition (EMT), angiogenesis, apoptosis, cell cycle, differentiation, DNA damage, DNA repair, hypoxia, inflammation and quiescence [63]. Then we applied Fisher's exact test to identify gene clusters significantly enriched in these 14 functional states. We identified candidate genes located near *SOX10* in the gene embeddings of both the melanoma and intermediate cell states, with a

clustering radius of 0.8 and a minimum of six genes per cluster. To validate the *SOX10* neighboring genes, we downloaded the scRNA-seq data from the same study for *SOX10* knockdown (KD) experiments for 3 intermediate samples (MM057, MM074, and MM087), each generated at multiple time points (24, 48, and 72 hours post-*SOX10* knockdown, plus a control with *SOX10*). Additionally, bulk RNA-seq data were collected from melanoma and intermediate cell lines following *SOX10* negative control and 72 hours post-knockdown. We compared AUCell scores [62] of *SOX10* neighboring genes before and after *SOX10* knockdown using scRNA-seq data (t-test, $P < 0.05$) and performed differential expression analysis on bulk RNA-seq data from normal and *SOX10*-KD samples using DESeq2 ($|\log2(FC)| > 1$ and $P < 0.05$) [60].

We performed pathway enrichment analysis using the R packages clusterProfiler (v.4.6.2) [64] and ReactomePA (v.1.42.0) [65], with KEGG [66], WikiPathways [67], and Reactome [68] as reference databases. ClueGO (v.2.5.10) [69] was used for pathway annotation and STRING [70] to analyze protein-protein interactions.

RESULTS

Overview of scGraph2Vec

scGraph2Vec was built on a VGAE framework with extensions for the task of generating informative embeddings. It took a gene-gene interaction network and a gene-feature matrix as the input and generated low-dimension gene embeddings as the output (Fig. 1A). scGraph2Vec had three major improvements to the standard VGAE framework. First, instead of using the standard adjacency matrix A, we generated an enhanced adjacency matrix A_n by combining *a priori*

primary community information and the standard adjacency matrix. We used the Louvain algorithm [41] to construct a membership matrix of genes and used this matrix to provide prior community information. The enhanced adjacency matrix A_n thus informed the encoder with information on primary assignments for gene clusters. Second, link prediction and community detection are simultaneously implemented during the model optimization process to enhance the representation of gene communities in the embedding space. Lastly, a modularity-inspired method was implemented to optimize the loss function to reduce the impact of local pairwise connections on community structure [42, 43]. By iteratively maximizing the joint of graph likelihood and modularity scores, scGraph2Vec generated latent features representing various information of genes in a high-dimensional space.

The working network was downloaded from BioGRID [37], including 19,752 genes and 977,356 interactions. Notably, the interactions were either physical (98.3%) or genetic (1.7%). Thus, it has no annotation about tissue-specificity or cell-type specificity and remains the same for all applications. The gene feature matrix was constructed using the scRNA-seq [71, 72] or the scATAC-seq [73, 74] data in the gene-by-cell format. We collected scRNA-seq data for 6 human tissues, which are brain [28], heart [29], kidney [30], liver [31], lung [32] and PBMC [33]. All scRNA-seq data were quality-controlled and processed following the same pipeline. On average, each tissue contained 66,395 cells (ranging from 2,638 cells in PBMC to 287,269 in the heart) and 24,064 genes (ranging from 13,714 genes in PBMC to 33,694 in the heart and kidney) (Supplementary Table S1). To ensure the same dimensionality of the inputs, the scRNA-seq gene expression matrix is trimmed or imputed to match the 19,752 genes available in the working

network.

We carried out a hyperparameter sweep to examine the key hyperparameters of scGraph2Vec. The best hyperparameters were determined by using a combination of assessment parameters, including the silhouette coefficient, the number of clusters, and the running time. We first selected the hyperparameters $\beta = 10$, $\lambda = 1$, $\gamma = 0.1$, and $s = 10$ (pink line, Supplementary Fig. S1A). Based on these hyperparameters, we next determined the following hyperparameters for model training: learning rate = 1×10^{-4}, a three-layer GCN encoder with 256, 64, and 16 neurons, respectively, and epoch time = 600 (blue line, Supplementary Fig. S1B). For larger datasets, we recommend a fast model with a two-layer GCN encoder (64 and 16 neurons for each layer). The resulting latent feature is a 19,752 genes × 16 vectors matrix.

scGraph2Vec generates gene embeddings for gene cluster identification

We reduced the latent features to a two-dimensional visualization space using t-SNE [44] and identified gene clusters by using the hierarchical clustering method. Due to the nature of neural network algorithms, such as random initialization, the same algorithm might generate slightly different results although each output was a faithful approximation of the input graph. Hence, we replicated the model training process 10 times for each dataset, establishing a pool for selecting the best gene clusters (Supplementary Fig. S2A-C).

Next, we measured the stability of the resultant models and gene clusters obtained from the 10 replications. We found that the predicted edges were highly consistent across 10 replications (69% of the BioGRID edges were identified in all 10 replicates). To assess the

stability of gene clustering, we took the fifth replication as the reference because it had the highest silhouette coefficient and then compared its resultant clusters with those from the other replications. We found that the majority of clusters could be replicated except cluster 8 and cluster 10, which were slightly mixed with other clusters (Supplementary Fig. S2C, D).

Key components of scGraph2Vec are important for gene cluster identification

We further investigated the importance of key components in scGraph2Vec by varying its structure, e.g., excluding the gene feature matrix, randomizing the reference network, and excluding or randomizing the primary community. In each case, we generated gene embeddings using the corresponding scGraph2Vec settings (the original design or those using alternative structures) and conducted hierarchical clustering based on their two-dimensional latent features to construct gene clusters. Then, we compared the resultant gene clusters to evaluate the models.

The standard output of scGraph2Vec generated the most distinguishable clusters (Fig. 2A). Particularly, when excluding the gene feature matrix, there were hardly any clusters formed based on the resultant embeddings in the brain or any other tissues tested (Supplementary Fig. S3A, B and Fig. S4A, B). As for the impact of the primary community, the formation of clusters became obscure either by excluding the primary community or by disrupting the primary community (Supplementary Fig. S3D, E and Fig. S4D, E), indicating that the primary community of scGraph2Vec played important roles in generating gene clusters with similar behaviors in the network.

We also found that the reference network was critical to identifying gene clusters. We

demonstrated this by randomizing the BioGRID network [37] or using alternative types of reference networks such as the Reactome [68] network that characterizes biological pathways or the TRRUST [40] network that represents transcription regulatory relationships. In the former case, when using a randomized BioGRID network, we did not find any cluster (Supplementary Fig. S3C and Fig. S4C). In the latter case, gene clusters can be distinguished regardless of the type of biological network used (Supplementary Fig. S5). The three types of networks are typical scale-free networks with different network characteristics (Supplementary Fig. S6). We evaluated the effect of degree centrality on the clustering of genes in the two-dimensional embedding space. By comparing gene clusters composed of hub genes with varying degree centrality and their neighbor genes, we found that scGraph2Vec is good at capturing small clusters with local connections, which is suitable for different types of biological networks (Supplementary Fig. S7). In contrast, the BioGRID network has a more uniform clustering effect. Thus, we used BioGRID as the main working network in the subsequent analysis.

Then, we compared the gene clusters identified using embedding trained on scRNA-seq and scATAC-seq data from the same brain samples [34]. Notably, when using scATAC-seq data, we constructed the feature matrix based on chromatin accessibility and mapped genomic regions to genes [75]. Through the t-SNE plot, most clusters could be replicated across different omics (Supplementary Fig. S8). This indicated that scGraph2Vec could detect gene clusters conserved in gene expression and chromatin accessibility through embeddings.

We used 64 logical CPUs and 512 GB RAM for all experiments. The execution time of scGraph2Vec depends on the size of the single-cell dataset and the interaction network. It ranges

from 6.5 hours on the smallest dataset (PBMC: 2,638 cells and 13,714 genes) to 37.3 hours on the largest dataset (heart: 287,269 cells and 33,694 genes). For the same brain dataset, the TRRUST network (the smallest in size: 2,862 genes and 8,427 interactions) takes 0.28 hours, while the BioGRID network (the largest in size: 19,752 genes and 977,356 interactions) takes 7.6 hours.

Benchmark of scGraph2Vec with existing methods

We benchmarked scGraph2Vec using the brain [28] and PBMC [33] datasets, along with 13 embedding methods: ARGA [24], ARGVA [24], SIG-VAE [25], DGVAE [26], scVAE [16], LDVAE [14], scVI [13], siVAE [18], scapGNN [20], scGNN [15], scETM [17], SAUCIE [12], and SIMBA [19]. We uniformly extracted latent features of all genes for each method based on the same gene expression matrix, where the unfixed VAE variants all used the same size encoder-decoder (256-64-16) structure. Notably, ARGA, ARGVA, SIG-VAE, and DGVAE are not designed specifically for biological data, and other methods were designed based on the gene expression matrix only (Supplementary Table S2).

Overall, scGraph2Vec showed more competitive results than other embedding methods in identifying gene clusters (Fig. 2B, C) (Supplementary Fig. 9). Methods developed for cell embeddings, such as scVI, LDVAE, SAUCIE and scETM, generally lose effectiveness in generating gene embeddings. Among the methods equipped with gene embedding, only siVAE demonstrated comparable cluster division to scGraph2Vec on the PBMC dataset (Fig. 2B, C). We were surprised to find very low similarity between clusters identified by different methods (Fig. 2D). Consistent conclusions were found when we compared two commonly used gene clustering

methods (SCENIC [47] and LIGER [48]) with scGraph2Vec (Supplementary Fig.10). Then we used the MSigDB hallmark gene set [46] as the ground truth gene set to evaluate the biological meaning of different clusters. scGraph2Vec accurately aggregates more functional genes than all competing algorithms (Fig. 2E).

scGraph2Vec generated biologically meaningful clusters

We applied scGraph2Vec to generate gene embeddings for 6 representative human tissues and subsequently generated gene clusters (Supplementary Fig. S11 and Table S3). As a result, we obtained an average of 134 gene clusters for each tissue, ranging from 108 to 149 (Supplementary Fig. S12). Each cluster contained tens to hundreds of genes, e.g., 68 to 270 genes per cluster for the brain (Supplementary Table S3). We evaluated these clusters for their topological characteristics, annotated gene sets, and expression patterns.

To test if the cluster genes were topologically correlated, we examined the closeness centrality of cluster genes. Taking the brain tissue as an example, the internal closeness centrality was significantly higher than the external closeness centrality of the clusters ($P < 2.22 \times 10^{-16}$, Fig. 3A). Thus, cluster genes were topologically related.

We next explored the biological implications of the clusters identified in different tissues using housekeeping genes and hallmark gene sets, as these genes were known to play critical functions in cells [49, 50]. We found that housekeeping genes tended to cluster together (Fig. 3B and Supplementary Fig. S13). As for the 50 hallmark gene sets, 26 were enriched in 40 gene clusters (Fig. 3C). The brain cluster 9 contained the highest number of housekeeping genes, with

59 genes, and enriched in hallmark gene sets related to MYC targets V1 ($P = 6.83 \times 10^{-29}$), MYC targets V2 ($P = 1.42 \times 10^{-7}$) and G2M checkpoint ($P = 3.97 \times 10^{-5}$).

To represent gene cluster expression across cell types, we computed the module score for each cluster at the single-cell level and visualized it using t-SNE, generated from the cell-by-gene expression matrix, with cell types annotated according to the original article [28] (Fig. 3D). We observed highly variable expression patterns of clusters among different cell types (the top 20 highly variable clusters, Supplementary Fig. S14). Among them, brain cluster 9 had high module scores in most neurons and cerebellar granule cells but had low module scores in other cell types (Fig. 3D).

scGraph2Vec generated clusters with cell-type and tissue specificity

Next, we tested if the cluster genes were enriched with cell-type-specific genes using GSEA [45]. To this end, we defined cell-type specific genes as the DEGs for each cell type using the original scRNA-seq data [28]. Taking the brain tissue as an example, we found a total of 37 clusters significantly enriched with at least one cell type ($P_{BH} < 0.05$, Fig. 4A and Supplementary Table S4). For example, cluster 9 was enriched with up-regulated DEGs of the largest number of associated cell types, including excitatory neuronal (Ex) subtype 3c (Ex3c), Ex5a, Ex8, Ex3b, Ex4, Ex3e, inhibitory neuronal (In) subtype 1c (In1c), pericytes (Per), and cerebellar granule cells (Gran) (Fig. 4A, B and Supplementary Fig. S15). GO enrichment analyses [64] showed that cluster 9 was related to functions such as cytoplasmic translation ($P_{BH} = 1.02 \times 10^{-8}$), ribosome biogenesis ($P_{BH} = 8.75 \times 10^{-8}$), ribonucleoprotein complex biogenesis ($P_{BH} = 7.83 \times 10^{-5}$) and

ribonucleoprotein processing ($P_{BH} = 1.68 \times 10^{-8}$) (Fig. 4C). Another example is cluster 59, which was enriched in multiple excitatory and inhibitory neurons and was particularly related to down-regulated DEGs in these cell types (Fig. 4A and Supplementary Fig. S15). Cluster 59 was mainly associated with functions of chromatin organization ($P_{BH} = 1.59 \times 10^{-39}$), histone modification ($P_{BH} = 3.44 \times 10^{-31}$), and histone acetylation ($P_{BH} = 6.57 \times 10^{-16}$) (Fig. 4D).

In the other five tissues, we identified 16 to 81 clusters enriched in cell type DEGs of the corresponding tissue. In the lung tissue, 24 of 120 clusters were significantly enriched in DEGs of 27 lung cell types, including airway smooth muscle, capillary aerocyte, alveolar fibroblast, and alveolar epithelial type 1 ($P_{BH} < 0.05$; Supplementary Fig. S16). In the heart tissue, 16 of 149 clusters were associated with DEGs of 15 heart cell types, such as cytoplasmic cardiomyocyte I and II, atrial cardiomyocyte, ventricular cardiomyocyte I and II ($P_{BH} < 0.05$; Supplementary Fig. S17). In liver, 81 of 137 clusters were significantly enriched in DEGs of 19 liver cell types, including hepatocytes, central venous liver sinusoidal endothelial cells, and hepatic stellate cells ($P_{BH} < 0.05$; Supplementary Fig. S18). In addition, we identified a total of 143 clusters in kidney, of which 72 were related to the DEGs of kidney cell types ($P_{BH} < 0.05$; Supplementary Fig. S19). In PBMC, 7 of 128 clusters were significantly enriched in DEGs of 4 cell types, i.e., B cell, CD14+ monocytes, memory CD4+ T cell, and naïve CD4+ T cell ($P_{BH} < 0.05$; Supplementary Fig. S20).

We also investigated the functions of the clusters and observed consistency in function and related cell types (Supplementary Fig. S21). The lung cluster 49 was enriched in cell types of alveolar epithelial type 1, alveolar fibroblast, and airway smooth muscle. It was associated with

functions of the chemokine-mediated signaling pathway ($P_{BH} = 1.85 \times 10^{-6}$) and negative regulation of endopeptidase activity ($P_{BH} = 2.08 \times 10^{-6}$). The heart cluster 80 was enriched in atrial cardiomyocyte and was associated with muscle cell development ($P_{BH} = 1.68 \times 10^{-8}$) and myofibril assembly ($P_{BH} = 2.80 \times 10^{-6}$). In the liver, several hepatocyte-enriched clusters were associated with distinct metabolic processes: cluster 35 with sulfur compound metabolic process ($P_{BH} = 0.002$) and gluconeogenesis ($P_{BH} = 0.002$), cluster 39 with organic acid catabolic process ($P_{BH} = 1.31 \times 10^{-12}$) and carboxylic acid catabolic process ($P_{BH} = 8.30 \times 10^{-12}$), and cluster 40 with nucleotide metabolic process ($P_{BH} = 3.57 \times 10^{-7}$). The kidney cluster 4 was associated with response to dietary excess ($P_{BH} = 0.007$) and fatty acid transport ($P_{BH} = 0.007$).

Next, we compared the cross-tissue differences in gene clusters. We defined tissue-specific clusters as those with no more than 5% significantly overlapping clusters, where overlap clusters were identified by shared genes with clusters from other tissues using Fisher's exact test. For the brain tissue, we identified 50/127 clusters as brain-specific, among which cluster 59, aforementioned as closely associated with the brain and enriched in genes of nBAF complex, was included (Fig. 4D, E). Then we searched for genes located near *SMARCE1*, a key gene of the nBAF complex in the embedding space using the framework to identify candidate genes. We compared the neighbor genes found in the brain and non-brain (i.e. lung) and reconstructed the predicted gene subnetwork (Fig. 4F, G). In the brain tissue, 14 genes were found in the neighboring subnetwork of *SMARCE1* and these genes were enriched with a neuron fate commitment, medial motor column neuron differentiation, and somatic motor neuron differentiation, among others (Fig. 4F). In contrast, in the lung tissue, 20 genes were found

adjacent to *SMARCE1* but they were enriched with general functions related to chromatin organization and histone H3-K9 (Fig. 4G).

Collectively, we demonstrated the high-dimensional biological information implied by gene embeddings in six representative human tissues and provided a general reference panel of tissue-related gene clusters.

scGraph2Vec found candidate disease-associated genes

Large-scale GWAS have identified thousands of genetic associations with diseases. Disease-associated genes often interact with each other and jointly disturb multiple pathways or regulatory networks in disease tissues or cell types. Therefore, understanding how genes interact in a context is crucial to understanding the molecular mechanisms of diseases. We next explored the ability of scGraph2Vec to infer disease-associated genes by using the resultant gene embeddings and demonstrated it in COVID-19 and AD.

Application in COVID-19. Using the HGI GWAS summary statistics for COVID-19 severity [76], we calculated gene-based p-values by using MAGMA [55] and identified 60 significant genes for COVID-19 severity (Bonferroni-corrected threshold $P < 2.63 \times 10^{-6}$). Next, we used the 60 genes as the seeds and searched for their neighbor genes in the two-dimensional embedding space of the lung tissue. As a result, we obtained 356 neighbor genes for COVID-19 severity. To validate these genes, we identified DEGs by comparing the bulk RNA-seq data from 102 COVID-19-positive patients and 26 COVID-19-negative individuals [56] ($P_{BH} < 0.05$ and |log2(FC)>1|) following the original study [56]. Notably, for the 60 GWAS-implied genes, only

six were DEGs (Fig. 5A). In contrast, 28 of 356 neighbor genes were validated to be DEGs. This proportion, though only marginally significant ($P = 0.067$, hypergeometric test; Fig. 5B), indicated that the gene embeddings indeed could be used to identify more disease-associated genes. This was further proved when we conducted the analyses using a disease-irrelevant tissue, i.e., brain, where 35 of the 681 identified neighbor genes were DEGs but were not statistically significant ($P = 0.83$; Fig. 5C). The 28 genes showed distinct expression patterns in COVID-19 and non-COVID-19 samples (Fig. 5D). Importantly, many genes were identified but were missed by the original GWAS results, such as *MKI67*, *STIL*, *NUF2*, *OIP5*, *TNFRSF17*, and *CEACAM8*. The 356 neighbor genes mainly related to cytokine receptor activity ($P_{BH} = 4.86 \times 10^{-6}$), C-C chemokine receptor activity ($P_{BH} = 4.86 \times 10^{-5}$), and peptidyl-lysine modification ($P_{BH} = 8.23 \times 10^{-5}$), among other functions (Fig. 5E left plot). Pathway enrichment analysis also revealed significant enrichment in Glycosaminoglycan degradation ($P_{BH} = 3.08 \times 10^{-4}$), type I interferon induction and signaling during SARS-CoV-2 infection ($P_{BH} = 6.46 \times 10^{-4}$), Interleukin-10 signaling ($P_{BH} = 3.03 \times 10^{-3}$), and ABO blood group biosynthesis ($P_{BH} = 3.29 \times 10^{-3}$) (Fig. 5F). STRING analysis further demonstrated protein-protein interactions among genes involved in these pathways (Fig. 5G). We particularly examined the 8 neighbor genes of the gene *CEACAM8*. These genes were enriched in biological processes such as the glycosaminoglycan (GAG) catabolic process ($P_{BH} = 3.19 \times 10^{-4}$, Fig. 5E right plot). GAGs serve as receptors for numerous microbial pathogens to adhere to and invade cells [77]. Recent evidence has shown that the entry process of SARS-CoV-2 into host cells was mediated by the transmembrane spike (S) protein interacting with both cellular heparan sulfate GAG and angiotensin converting enzyme 2 (ACE2)

[78, 79]. Thus, GAG derivatives have been promising candidates for SARS-CoV-2 antiviral therapy [80, 81].

Application in Alzheimer's Disease. Using a large-scale GWAS for AD (71,880 cases and 383,378 controls) [54], we identified 65 AD-associated genes by MAGMA at the Bonferroni-corrected threshold $P < 3.77 \times 10^{-6}$. Fourteen of 65 genes were DEGs ($P_{BH} < 0.05$ and |fold change| > 1.1) between the AD group and the control group using an independent bulk RNA-seq data [57] (postmortem brain tissues of 376 late-onset AD patients and 173 normal samples, Fig. 6A). Using the 65 genes as seed genes and the gene embeddings for the normal brain tissue, we identified 362 neighbor genes for AD. These newly identified AD-candidate genes were significantly enriched with DEGs (112/362, hypergeometric test $P = 0.011$, Fig. 6B). As a negative control, we found 317 neighbor genes near the 65 seed genes using the gene embeddings generated for an irrelevant tissue, i.e., the healthy lung tissue. However, only 78 of 317 genes were DEGs and this proportion was not statistically significant ($P = 0.68$, hypergeometric test; Fig. 6C). These neighbor genes identified by latent features can well distinguish AD patients from healthy controls (Fig. 6D). Functional enrichment analysis of these genes indicated interesting GO terms that might be important pathogenic causes for AD, such as the mitochondrial electron transport chain ($P_{BH} = 2.75 \times 10^{-8}$; Fig. 6E left plot) [82]. Pathway annotation of 362 genes revealed regulatory pathways involved in AD, mainly complex I biogenesis ($P_{BH} = 9.00 \times 10^{-10}$), cholesterol transport ($P_{BH} = 1.07 \times 10^{-4}$), regulation of cholesterol transport ($P_{BH} = 9.48 \times 10^{-4}$), regulation of amyloid-beta clearance ($P_{BH} = 9.83 \times 10^{-5}$) and oxidative phosphorylation ($P_{BH} = 3.67 \times 10^{-9}$) (Fig. 6F, G). Furthermore, the newly identified AD-candidate genes can help to better

explain the molecular mechanisms of potential targets. For example, we found a causal gene *SERPINA3* that had been verified to be associated with AD but had never been detected by GWAS [83]. Another AD risk gene, *APOC1*, and its 8 neighbor genes (such as *ADORA2A*, *CDH2*, and *SLC28A2*) were enriched in functions related to negative regulation of hydrolase activity (P_{BH} = 0.01), regulation of synaptic transmission (P_{BH} = 0.01), glutamatergic (P_{BH} = 0.01), negative regulation of phosphatidylcholine catabolic process (P_{BH} = 0.01) and so on (Fig. 6E right plot).

scGraph2Vec identified candidate driver genes in LUAD

We also demonstrated the effectiveness of scGraph2Vec in identifying candidate driver-like genes in cancer. Using scRNA-seq data from LUAD patients [35], we generated embeddings for tumor and normal lung tissues, respectively, and identified gene clusters in each condition (Supplementary Fig. S22A, B and Table S3). Notably, in the two-dimensional embedding space, we observed a partial enrichment of housekeeping genes (Supplementary Fig. S22C, D). By comparing the overlapping genes in the clusters from either normal or tumor, we identified 41 clusters specifically enriched in tumor (Fig. 7A). These tumor-specific clusters were enriched in functions related to oxidative phosphorylation ($P_{BH} = 1.13 \times 10^{-87}$), double-strand break repair ($P_{BH} = 1.45 \times 10^{-7}$), cytokine-mediated signaling pathway ($P_{BH} = 4.14 \times 10^{-6}$), cell-cell adhesion via plasma-membrane adhesion molecules (P_{BH} = 0.005), and stem cell population maintenance (P_{BH} = 0.004) (Fig. 7B). Then, we used the 18 LUAD-driver genes reported by TCGA as seeds (*ARID1A*, *BRAF*, *CDKN2A*, *EGFR*, *KEAP1*, *KRAS*, *MET*, *MGA*, *NF1*, *PIK3CA*, *RB1*, *RBM10*,

RIT10, *SETD2*, *SMARCA4*, *STK11*, *TP53*, and *U2AF1*) [58], and identified 251 neighbor genes in the embeddings of tumor and 505 neighbor genes in normal. Notably, we observed that the enrichment of these neighbor genes aligned with the tissue context of the respective samples. For example, *TP53* neighbor genes in normal are enriched for functions like DNA conformation change (P_{BH} = 0.005), protein-DNA complex assembly (P_{BH} = 0.005), and epidermal cell division (P_{BH} = 0.005). However, *TP53* neighbor genes in tumor are enriched for functions related to tumor development, such as negative regulation of DNA replication (P_{BH} = 0.02), 7-methylguanosine cap hypermethylation (P_{BH} = 0.02), negative regulation of pentose-phosphate shunt (P_{BH} = 0.02), and mitotic DNA damage checkpoint signaling (P_{BH} = 0.03) (Fig. 7C).

To validate the neighbor genes, we downloaded bulk RNA-seq data of LUAD samples from TCGA including expression data for 238 of the 251 neighbor genes identified in the tumor. Among all 238 neighbor genes, 109 were also DEGs ($|\log2(FC)| > 0.5$ and $P_{BH} < 0.05$). Specifically, for *TP53*, 9 out of 10 neighbor genes were DEGs (Fig. 7D, Wilcoxon rank-sum test, $P < 0.05$). Subsequently, using the univariate Cox proportional hazards model, we discovered 44 prognosis-related genes ($P < 0.05$), among which 27 genes were also DEGs (Fig. 7E). Then we conducted survival analysis using each of the 27 genes to stratify samples by the median of log2 (TPM + 1) as the cutoff value. High expression of 13 genes and low expression of 5 genes were associated with poor prognosis in LUAD patients (Fig. 7F, G and Supplementary Fig. S23). Several were recognized as LUAD-related genes, including *ABCE1* [84], *CCNE1* [85], *CHCHD2* [86], *ERG* [87], *HOXA1* [88], *KRAS* [89], *KRT17* [90], *LHX2* [91], *LTB* [92] and *SHMT2* [93], among others.

scGraph2Vec revealed regulatory genes underlie cell state transitions in melanoma

scGraph2Vec can generate gene embeddings for different cell states and reveal gene regulatory programs underlying cell state stabilization and transitions. We demonstrated it using melanoma scRNA-seq data including cells of three melanocyte cell states. Based on the gene embeddings, we identified 149 clusters for the melanocytic state (62 significantly specific to melanocytic), 149 for the intermediate state (59 specifics), and 133 for the mesenchymal state (47 specifics) (Supplementary Table S3). Furthermore, using AUCell [62] to quantitatively assess the cluster activities, we found that 120/149 melanocytic clusters, 122/149 intermediate clusters, and 37 mesenchymal clusters showed significant differences across three states ($P < 0.05$ for ANOVA and t-test; high activity). Then we identified gene clusters that reflect distinct cellular functional states based on the gene signatures of 14 crucial cancer cell states [63]. In total, we discovered 15 gene clusters that were significantly enriched with these functional signatures (Fisher's exact test, $P < 0.05/14$). These clusters also exhibited notable differences in AUCell activity, with the majority (13 out of 15) being cell state-specific (Fig. 8A). We observed that gene clusters in the melanocytic state were primarily associated with the DNA repair, cell cycle and differentiation, while those in the mesenchymal state were enriched for DNA damage, quiescence, inflammation, metastasis, hypoxia, EMT and so on (Fig. 8B). The intermediate state, acting as a transitional phase, displayed features of both (Fig. 8B). Among three cell states, melanocytic cluster 4, intermediate cluster 30, and mesenchymal cluster 37 showed the most significantly increase in AUCell activity (Fig. 8C), each representing a distinct functional state: melanocytic cluster 4 was

enriched in DNA repair, intermediate cluster 30 in hypoxia responses, and mesenchymal cluster 37 in EMT and hypoxia responses. Consistent with previous studies, melanocytic cells were characterized by a high proliferation rate; mesenchymal cells exhibited high invasive capacity; and intermediate cells presented with mixed characteristics that shared with the other two states [94-96]. And EMT is a process where melanocytes lose their epithelial characteristics and acquire a mesenchymal phenotype, which enhances melanoma cell motility, invasiveness, and metastatic potential [97, 98].

In addition to identifying state-specific gene clusters, scGraph2Vec can also help identify critical genes during cell state transitions. Previous studies have reported that the transcription factor *SOX10* drives the dynamic transition of melanoma cells from a melanocytic state to a mesenchymal state [36, 99]. *SOX10* was found to be highly expressed in seven cell cultures at the melanocytic and intermediate states (MM001, MM011, MM031, MM057, MM074, MM087, and A375) (Fig. 8D). Using the embeddings generated by scGraph2Vec, we identified 169 neighbor genes of *SOX10* in the melanocytic state and 182 in the intermediate state, respectively. Using the bulk RNA-seq data generated at 72 hours after *SOX10* knockdown, we identified 555 DEGs ($|\log2(FC)| > 1$ and $P < 0.05$) for the melanocytic state. Five of these DEGs were included in the *SOX10* neighbor genes, which were *E2F1*, *JUN*, *SMAD3*, *ETS1* and *CBX2* (Fig. 8E, left panel). Similarly, among the 498 DEGs for the intermediate state, six were included in our neighbor genes, including those involved in cell differentiation and cell cycle processes, such as *SOX9*, *CCNA2*, *TBL1X*, and *NUMA1*, as well as immune signaling-related genes like *RBPJ* (Fig. 8E, right panel). STRING analysis also revealed close protein-protein interactions among these genes

(Supplementary Fig. 24). We further validated the 182 neighbor genes for the intermediate state where scRNA-seq data were available. As shown in Fig. 8F, *SOX10*-neighbor genes showed significantly increased activity 72 hours after *SOX10* knockdown in the intermediate-state cells compared to the control cells (T-test, $P < 0.05$). This was observed in all three samples with the intermediate state (Fig. 8F). Most of these neighboring genes were highly expressed in the mesenchymal state (Fig. 8G), with *SOX9*, *JUN*, and *SMAD3* showing significantly high expression (log2(FC) > 1 and $P_{BH} < 0.05$). In summary, scGraph2Vec can infer neighboring genes of interest in different cell states based on gene embeddings, providing insights into cell state-specific regulatory relationships.

DISCUSSION

We developed scGraph2Vec based on GNN to represent tissue-specific gene high-dimensional characteristics by integrating heterogeneous information of gene interaction networks and single-cell gene matrices. On benchmark datasets, we showed that scGraph2Vec outperformed competing algorithms by generating gene embeddings that can delineate gene clusters and cluster functionally meaningful genes. Using scRNA-seq data from six types of human normal tissues and three different types of gene networks, we demonstrated that gene embeddings can capture the topological features of various biological networks, cluster sets of co-functioning genes, and reveal tissue- or cell-type-specific gene clusters. Additionally, we highlighted application cases in COVID-19, AD, LUAD, and melanoma, showing that scGraph2Vec is versatile in analyzing samples from normal human tissues, case-control tumor

samples, and cell lines in different states. By searching for neighboring genes in the embedding space, we identified additional disease-associated genes and revealed gene regulatory programs across diverse biological contexts. Since gene embeddings accurately measure and integrate molecular patterns at different levels, they provide more complete associations between genes and phenotypes in tissues, effectively complementing strategies for studying disease-associated genes.

The precise description of genes in complex multicellular organisms depends on the interaction between genes and gene products in response to the cellular environment. The rapidly accumulated single-cell omics data have provided an unprecedented opportunity to identify the cellular processes of genes under different cell lineages and environmental changes. However, it is challenging to accurately infer the regulatory programs of genes in cell lineages from these data. One is that most of the data have heterogeneous structures and are generated from different experimental designs and technical platforms. In addition, experimental techniques for generating high-throughput tissue-specific gene interactions are currently unfeasible, especially for cell line samples that are not readily available. With few exceptions, existing networks often lack fine-grained regulation, resulting in disease modules that are either overly fragmented or too extensive, making observable disease modules less discernible [100, 101]. Therefore, there is an urgent need to develop comprehensive computational methods to integrate multi-level genome data and provide systematic explanations for tissue-specific regulatory networks. Our approach integrated gene interaction network with single-cell gene profiles, allowing for extension to various types of gene networks and single-cell data across different biological contexts. This scalability is especially

important in an era when there is already a large amount of publicly available gene networks and single-cell omics data. The gene embeddings generated from scGraph2Vec can provide the necessary background panel for gene regulation, identify co-functional gene modules, and contribute to studying pathogenic mechanisms of disease-associated genes.

One limitation of scGraph2Vec is that it relies on the data quality of the gene feature matrix that provides tissue-specific information. In this work, we used publicly available scRNA-seq data from human normal tissues, tumor tissues, and cell lines. The scRNA-seq data is noisy and incomplete, especially since it comes from specific sampling sites and different experimental designs. Graph embedding can reduce some technical noises, but cannot impute the unknown tissue-specific information. Second, the boundaries of gene clusters are difficult to determine. The regulation pattern of gene sets in a given tissue is ambiguous. The physical distance between genes in the embedding space extracted by scGraph2Vec does not represent the actual distance of the regulatory relationship between genes, because it often depends on the gene interaction network and gene feature matrix used in the model training. Furthermore, scGraph2Vec is better at capturing small clusters with local connections, making it more suitable for scale-free networks with numerous sparse connections, a characteristic of most biological interaction networks. To increase the adaptability and interpretability of our method, we recommend the use of multiple single-relationship gene interaction networks for the analyses. Finally, scGraph2Vec is a unified and highly scalable framework for integrative analysis of various networks and single-cell datasets from a given tissue. We therefore expect this work to be of interest, especially since tissue-specific gene interaction networks are lacking and needed for various types of downstream

analysis.

AVAILABILITY OF SOURCE CODE AND REQUIREMENTS

Project name: scGraph2Vec

Project home page: https://github.com/LPH-BIG/scGraph2Vec

Operating system: Platform independent

Programming language: Python

Other requirements: Python 3.7 or higher, TensorFlow 1.15.0

License: MIT License

RRID: SCR_025322

biotoolsID: scGraph2Vec

DATA AVAILABILITY

The scRNA-seq data used in this article are all publicly available. The liver tissue data and paired scRNA-seq and scATAC-seq data can be accessed from GEO, with the access numbers: GSE115469 and GSE162170, respectively. Other scRNA-seq datasets for this article are available via the following databases: brain dataset from the human cell landscape [102]; heart dataset from the Single Cell Portal [103] with accession code: SCP498; kidney dataset from the Kidney Cell Atlas, specifically the mature kidney dataset [30]; lung dataset from the Human Lung Cell Atlas [32] with Synapse ID: syn21041850; PBMC dataset from the 10X Genomics 3k PBMC supporting dataset; LUAD dataset from EMBL-EBI database with accession code: E-MTAB-6149 and E-MTAB-6653.

Furthermore, the RNA-seq data from the brain and plasma are available as supplementary data of their respective article [56, 57]. The RNA-seq data for LUAD is from the TCGA database with project ID TCGA-LUAD [58]. The melanoma datasets were downloaded from the Scope (Scope session: Wouters_Human_Melanoma) and GEO (accession ID: GSE134432). The data and code for transparent and reproducible results are available in Zenodo [104] and GitHub [105]. DOME-ML annotations are available via the DOME wizard [106].

ADDITIONAL FILES

Supplementary Table 1: Statistics of the datasets used.

Supplementary Table 2: Summary of methods used for comparison.

Supplementary Table 3: Gene clusters identified by scGraph2Vec in 6 representative human tissues, tumor/normal tissues of LUAD and 3 melanocyte cell states.

Supplementary Table 4: The results of applying GSEA to identify potential enrichment of gene clusters among brain cell type-specific genes.

Supplementary Figure 1: The hyperparameter sweep result.

Supplementary Figure 2: The evaluation of model stability.

Supplementary Figure 3: Performance evaluation of the key components in scGraph2Vec by varying its structure using brain dataset.

Supplementary Figure 4: Performance evaluation of the key components in scGraph2Vec by varying its structure using PBMC dataset.

Supplementary Figure 5: Clustering results based on the embeddings obtained by using the

Reactome network (the left panel) and the TRRUST network (the right panel), respectively.

Supplementary Figure 6: Characteristics of three biological networks.

Supplementary Figure 7: Evaluate the clustering of genes grouped by varying degree centrality.

Supplementary Figure 8: Gene clusters cross-validated using scRNA-seq and scATAC-seq data from the same brain samples.

Supplementary Figure 9: Comparison of gene embedding among methods on the same brain and PBMC datasets.

Supplementary Figure 10: Clustering measurement of SCENIC and LIGER gene clusters in scGraph2Vec two-dimensional embedding space.

Supplementary Figure 11: Gene clusters in 6 human tissues.

Supplementary Figure 12: The optimal clustering was selected by silhouette coefficient in 10 replications.

Supplementary Figure 13: The distribution of housekeeping genes in latent features from 6 human tissues.

Supplementary Figure 14: Scaled module scores in cells for the top 20 highly variable clusters.

Supplementary Figure 15: GSEA analysis between the gene clusters (cluster 9 and cluster 59) and cell types in the brain dataset.

Supplementary Figure 16: GSEA for lung clusters.

Supplementary Figure 17: GSEA for heart clusters.

Supplementary Figure 18: GSEA for liver clusters.

Supplementary Figure 19: GSEA of kidney clusters.

Supplementary Figure 20: GSEA of PBMC clusters.

Supplementary Figure 21: GO enrichment analysis for cluster genes.

Supplementary Figure 22: Gene clusters in tumor tissue (A) and normal tissue(B). Housekeeping genes in latent features from tumor tissue (C) and normal tissue (D).

Supplementary Figure 23: Kaplan–Meier survival analysis of 16 out of 18 overlap candidate genes.

Supplementary Figure 24: STRING analysis for the 12 validated *SOX10* neighbor genes.

ABBREVIATIONS

AD: Alzheimer's disease; DEG: differentially expressed gene; DBI: Davies-Bouldin index; Ex: excitatory neuronal; EMT: epithelial-mesenchymal transition; GAG: glycosaminoglycan; GCN: graph convolutional network; GNN: graph neural network; Gran: cerebellar granule cells; GSEA: gene set enrichment analysis; GO: Gene Ontology; GWAS: genome-wide association studies; HR: hazard ratio; In: inhibitory neuronal; iNMF: integrative non-negative matrix factorization; KD: knockdown; LTMG: Left Truncated Mixture Gaussian; LUAD: lung adenocarcinoma; NSCLC: non-small cell lung cancer; PBMC: peripheral blood mononuclear cells; Per: pericytes; PPI: protein-protein interaction; TCGA: The Cancer Genome Atlas; t-SNE: t-Distributed Stochastic Neighbor Embedding; TPM: transcript per million; VGAE: variational graph autoencoder.

FUNDING STATEMENT

This research was funded by the Strategic Priority Research Program of the Chinese Academy of Sciences [XDB38010400], the National Natural Science Foundation of China [32270706] and the Shanghai Municipal Science and Technology Major Project (2018SHZDZX01).

ACKNOWLEDGMENTS

The authors would like to thank Yinying Wang and the members of the Laboratory for Precision Health for their valuable discussion.

AUTHOR CONTRIBUTIONS

P.J. conceived the project. S.L. and P.J. developed the method and analyzed the results. P.J. supervised the study. S.L. and P.J. wrote the paper.

DECLARATION OF INTEREST

The authors declare no competing interests.

REFERENCES

1. Langfelder P and Horvath S. WGCNA: an R package for weighted correlation network analysis. BMC Bioinformatics. 2008;9:559. doi:10.1186/1471-2105-9-559.
2. Tirosh I, Izar B, Prakadan SM, Wadsworth MH, 2nd, Treacy D, Trombetta JJ, et al. Dissecting the multicellular ecosystem of metastatic melanoma by single-cell RNA-seq. Science (New York, NY). 2016;352 6282:189-96. doi:10.1126/science.aad0501.
3. Sonawane AR, Platig J, Fagny M, Chen CY, Paulson JN, Lopes-Ramos CM, et al. Understanding Tissue-Specific Gene Regulation. Cell Rep. 2017;21 4:1077-88. doi:10.1016/j.celrep.2017.10.001.
4. Pierson E, Koller D, Battle A, Mostafavi S, Ardlie KG, Getz G, et al. Sharing and

Specificity of Co-expression Networks across 35 Human Tissues. PLoS Comput Biol. 2015;11 5:e1004220. doi:10.1371/journal.pcbi.1004220.
5. Huang JK, Carlin DE, Yu MK, Zhang W, Kreisberg JF, Tamayo P, et al. Systematic Evaluation of Molecular Networks for Discovery of Disease Genes. Cell systems. 2018;6 4:484-95.e5. doi:10.1016/j.cels.2018.03.001.
6. Lonsdale J, Thomas J, Salvatore M, Phillips R, Lo E, Shad S, et al. The genotype-tissue expression (GTEx) project. Nat Genet. 2013;45 6:580-5.
7. Lizio M, Abugessaisa I, Noguchi S, Kondo A, Hasegawa A, Hon CC, et al. Update of the FANTOM web resource: expansion to provide additional transcriptome atlases. Nucleic acids research. 2019;47 D1:D752-d8. doi:10.1093/nar/gky1099.
8. The human body at cellular resolution: the NIH Human Biomolecular Atlas Program. Nature. 2019;574 7777:187-92. doi:10.1038/s41586-019-1629-x.
9. Regev A, Teichmann SA, Lander ES, Amit I, Benoist C, Birney E, et al. The Human Cell Atlas. Elife. 2017;6 doi:10.7554/eLife.27041.
10. Wong AK, Sealfon RSG, Theesfeld CL and Troyanskaya OG. Decoding disease: from genomes to networks to phenotypes. Nature reviews Genetics. 2021;22 12:774-90. doi:10.1038/s41576-021-00389-x.
11. Wang S, Flynn ER and Altman RB. Gaussian Embedding for Large-scale Gene Set Analysis. Nat Mach Intell. 2020;2 7:387-95. doi:10.1038/s42256-020-0193-2.
12. Amodio M, van Dijk D, Srinivasan K, Chen WS, Mohsen H, Moon KR, et al. Exploring single-cell data with deep multitasking neural networks. Nature methods. 2019;16 11:1139-45. doi:10.1038/s41592-019-0576-7.
13. Lopez R, Regier J, Cole MB, Jordan MI and Yosef N. Deep generative modeling for single-cell transcriptomics. Nature methods. 2018;15 12:1053-8. doi:10.1038/s41592-018-0229-2.
14. Svensson V, Gayoso A, Yosef N and Pachter L. Interpretable factor models of single-cell RNA-seq via variational autoencoders. Bioinformatics (Oxford, England). 2020;36 11:3418-21. doi:10.1093/bioinformatics/btaa169.
15. Wang J, Ma A, Chang Y, Gong J, Jiang Y, Qi R, et al. scGNN is a novel graph neural network framework for single-cell RNA-Seq analyses. Nature communications. 2021;12 1:1882. doi:10.1038/s41467-021-22197-x.
16. Grønbech CH, Vording MF, Timshel PN, Sønderby CK, Pers TH and Winther O. scVAE: variational auto-encoders for single-cell gene expression data. Bioinformatics (Oxford, England). 2020;36 16:4415-22. doi:10.1093/bioinformatics/btaa293.
17. Zhao Y, Cai H, Zhang Z, Tang J and Li Y. Learning interpretable cellular and gene signature embeddings from single-cell transcriptomic data. Nature communications. 2021;12 1:5261. doi:10.1038/s41467-021-25534-2.
18. Choi Y, Li R and Quon G. siVAE: interpretable deep generative models for single-cell transcriptomes. Genome Biol. 2023;24 1:29. doi:10.1186/s13059-023-02850-y.
19. Chen H, Ryu J, Vinyard ME, Lerer A and Pinello L. SIMBA: single-cell embedding along with features. Nature methods. 2023; doi:10.1038/s41592-023-01899-8.

20. Han X, Wang B, Situ C, Qi Y, Zhu H, Li Y, et al. scapGNN: A graph neural network-based framework for active pathway and gene module inference from single-cell multi-omics data. PLoS biology. 2023;21 11:e3002369. doi:10.1371/journal.pbio.3002369.
21. Kipf TN and Welling MJapa. Variational graph auto-encoders. 2016.
22. Scarselli F, Gori M, Tsoi AC, Hagenbuchner M and Monfardini G. The graph neural network model. IEEE Trans Neural Netw. 2009;20 1:61-80. doi:10.1109/tnn.2008.2005605.
23. Micheli A. Neural network for graphs: a contextual constructive approach. IEEE Trans Neural Netw. 2009;20 3:498-511. doi:10.1109/tnn.2008.2010350.
24. Pan S, Hu R, Long G, Jiang J, Yao L and Zhang CJapa. Adversarially regularized graph autoencoder for graph embedding. 2018.
25. Hasanzadeh A, Hajiramezanali E, Narayanan K, Duffield N, Zhou M and Qian XJAinips. Semi-implicit graph variational auto-encoders. 2019;32.
26. Li J, Yu J, Li J, Zhang H, Zhao K, Rong Y, et al. Dirichlet graph variational autoencoder. 2020;33:5274-83.
27. Salha-Galvan G, Lutzeyer JF, Dasoulas G, Hennequin R and Vazirgiannis M. Modularity-aware graph autoencoders for joint community detection and link prediction. Neural Netw. 2022;153:474-95. doi:10.1016/j.neunet.2022.06.021.
28. Lake BB, Chen S, Sos BC, Fan J, Kaeser GE, Yung YC, et al. Integrative single-cell analysis of transcriptional and epigenetic states in the human adult brain. Nat Biotechnol. 2018;36 1:70-80. doi:10.1038/nbt.4038.
29. Tucker NR, Chaffin M, Fleming SJ, Hall AW, Parsons VA, Bedi KC, Jr., et al. Transcriptional and Cellular Diversity of the Human Heart. Circulation. 2020;142 5:466-82. doi:10.1161/circulationaha.119.045401.
30. Stewart BJ, Ferdinand JR, Young MD, Mitchell TJ, Loudon KW, Riding AM, et al. Spatiotemporal immune zonation of the human kidney. Science (New York, NY). 2019;365 6460:1461-6. doi:10.1126/science.aat5031.
31. MacParland SA, Liu JC, Ma XZ, Innes BT, Bartczak AM, Gage BK, et al. Single cell RNA sequencing of human liver reveals distinct intrahepatic macrophage populations. Nature communications. 2018;9 1:4383. doi:10.1038/s41467-018-06318-7.
32. Travaglini KJ, Nabhan AN, Penland L, Sinha R, Gillich A, Sit RV, et al. A molecular cell atlas of the human lung from single-cell RNA sequencing. Nature. 2020;587 7835:619-25. doi:10.1038/s41586-020-2922-4.
33. Zheng GX, Terry JM, Belgrader P, Ryvkin P, Bent ZW, Wilson R, et al. Massively parallel digital transcriptional profiling of single cells. Nature communications. 2017;8:14049. doi:10.1038/ncomms14049.
34. Trevino AE, Müller F, Andersen J, Sundaram L, Kathiria A, Shcherbina A, et al. Chromatin and gene-regulatory dynamics of the developing human cerebral cortex at single-cell resolution. Cell. 2021;184 19:5053-69.e23. doi:10.1016/j.cell.2021.07.039.
35. Lambrechts D, Wauters E, Boeckx B, Aibar S, Nittner D, Burton O, et al. Phenotype molding of stromal cells in the lung tumor microenvironment. Nat Med. 2018;24 8:1277-89. doi:10.1038/s41591-018-0096-5.

36. Wouters J, Kalender-Atak Z, Minnoye L, Spanier KI, De Waegeneer M, Bravo González-Blas C, et al. Robust gene expression programs underlie recurrent cell states and phenotype switching in melanoma. Nature cell biology. 2020;22 8:986-98. doi:10.1038/s41556-020-0547-3.
37. Oughtred R, Rust J, Chang C, Breitkreutz BJ, Stark C, Willems A, et al. The BioGRID database: A comprehensive biomedical resource of curated protein, genetic, and chemical interactions. Protein Sci. 2021;30 1:187-200. doi:10.1002/pro.3978.
38. Jassal B, Matthews L, Viteri G, Gong C, Lorente P, Fabregat A, et al. The reactome pathway knowledgebase. Nucleic acids research. 2020;48 D1:D498-d503. doi:10.1093/nar/gkz1031.
39. Rodchenkov I, Babur O, Luna A, Aksoy BA, Wong JV, Fong D, et al. Pathway Commons 2019 Update: integration, analysis and exploration of pathway data. Nucleic acids research. 2020;48 D1:D489-d97. doi:10.1093/nar/gkz946.
40. Han H, Cho JW, Lee S, Yun A, Kim H, Bae D, et al. TRRUST v2: an expanded reference database of human and mouse transcriptional regulatory interactions. Nucleic acids research. 2018;46 D1:D380-d6. doi:10.1093/nar/gkx1013.
41. Blondel VD, Guillaume J-L, Lambiotte R, Lefebvre EJJosmt and experiment. Fast unfolding of communities in large networks. 2008;2008 10:P10008.
42. Lobov I and Ivanov SJapa. Unsupervised community detection with modularity-based attention model. 2019.
43. Wang X, Cui P, Wang J, Pei J, Zhu W and Yang S. Community preserving network embedding. In: *Proceedings of the AAAI conference on artificial intelligence* 2017.
44. Van der Maaten L and Hinton GJJomlr. Visualizing data using t-SNE. 2008;9 11.
45. Subramanian A, Tamayo P, Mootha VK, Mukherjee S, Ebert BL, Gillette MA, et al. Gene set enrichment analysis: a knowledge-based approach for interpreting genome-wide expression profiles. Proc Natl Acad Sci U S A. 2005;102 43:15545-50. doi:10.1073/pnas.0506580102.
46. Liberzon A, Subramanian A, Pinchback R, Thorvaldsdóttir H, Tamayo P and Mesirov JP. Molecular signatures database (MSigDB) 3.0. Bioinformatics (Oxford, England). 2011;27 12:1739-40. doi:10.1093/bioinformatics/btr260.
47. Aibar S, González-Blas CB, Moerman T, Huynh-Thu VA, Imrichova H, Hulselmans G, et al. SCENIC: single-cell regulatory network inference and clustering. Nature methods. 2017;14 11:1083-6. doi:10.1038/nmeth.4463.
48. Welch JD, Kozareva V, Ferreira A, Vanderburg C, Martin C and Macosko EZ. Single-Cell Multi-omic Integration Compares and Contrasts Features of Brain Cell Identity. Cell. 2019;177 7:1873-87.e17. doi:10.1016/j.cell.2019.05.006.
49. Liberzon A, Birger C, Thorvaldsdóttir H, Ghandi M, Mesirov JP and Tamayo P. The Molecular Signatures Database (MSigDB) hallmark gene set collection. Cell systems. 2015;1 6:417-25. doi:10.1016/j.cels.2015.12.004.
50. Hsiao LL, Dangond F, Yoshida T, Hong R, Jensen RV, Misra J, et al. A compendium of gene expression in normal human tissues. Physiol Genomics. 2001;7 2:97-104. doi:10.1152/physiolgenomics.00040.2001.

51. Hao Y, Hao S, Andersen-Nissen E, Mauck WM, 3rd, Zheng S, Butler A, et al. Integrated analysis of multimodal single-cell data. Cell. 2021;184 13:3573-87.e29. doi:10.1016/j.cell.2021.04.048.
52. Ester M, Kriegel H-P, Sander J and Xu X. Density-based spatial clustering of applications with noise. In: *Int Conf knowledge discovery and data mining* 1996.
53. The COVID-19 Host Genetics Initiative, a global initiative to elucidate the role of host genetic factors in susceptibility and severity of the SARS-CoV-2 virus pandemic. 1476-5438 (Electronic).
54. Jansen IE, Savage JE, Watanabe K, Bryois J, Williams DM, Steinberg S, et al. Genome-wide meta-analysis identifies new loci and functional pathways influencing Alzheimer's disease risk. Nat Genet. 2019;51 3:404-13. doi:10.1038/s41588-018-0311-9.
55. de Leeuw CA, Mooij JM, Heskes T and Posthuma D. MAGMA: generalized gene-set analysis of GWAS data. PLoS Comput Biol. 2015;11 4:e1004219. doi:10.1371/journal.pcbi.1004219.
56. Overmyer KA, Shishkova E, Miller IJ, Balnis J, Bernstein MN, Peters-Clarke TM, et al. Large-Scale Multi-omic Analysis of COVID-19 Severity. Cell systems. 2021;12 1:23-40.e7. doi:10.1016/j.cels.2020.10.003.
57. Williams JB, Cao Q and Yan Z. Transcriptomic analysis of human brains with Alzheimer's disease reveals the altered expression of synaptic genes linked to cognitive deficits. Brain Commun. 2021;3 3:fcab123. doi:10.1093/braincomms/fcab123.
58. Comprehensive molecular profiling of lung adenocarcinoma. Nature. 2014;511 7511:543-50. doi:10.1038/nature13385.
59. Albertina B, Watson M, Holback C, Jarosz R, Kirk S, Lee Y, et al. The Cancer Genome Atlas Lung Adenocarcinoma Collection (TCGA-LUAD)(Version 4)[Data Set]. 2016.
60. Love MI, Huber W and Anders S. Moderated estimation of fold change and dispersion for RNA-seq data with DESeq2. Genome Biol. 2014;15 12:550. doi:10.1186/s13059-014-0550-8.
61. Lin H and Zelterman D. Modeling survival data: extending the Cox model. Taylor & Francis, 2002.
62. Van de Sande B, Flerin C, Davie K, De Waegeneer M, Hulselmans G, Aibar S, et al. A scalable SCENIC workflow for single-cell gene regulatory network analysis. Nature protocols. 2020;15 7:2247-76. doi:10.1038/s41596-020-0336-2.
63. Yuan H, Yan M, Zhang G, Liu W, Deng C, Liao G, et al. CancerSEA: a cancer single-cell state atlas. Nucleic acids research. 2019;47 D1:D900-d8. doi:10.1093/nar/gky939.
64. Wu T, Hu E, Xu S, Chen M, Guo P, Dai Z, et al. clusterProfiler 4.0: A universal enrichment tool for interpreting omics data. Innovation (Camb). 2021;2 3:100141. doi:10.1016/j.xinn.2021.100141.
65. Yu G and He QY. ReactomePA: an R/Bioconductor package for reactome pathway analysis and visualization. Molecular bioSystems. 2016;12 2:477-9. doi:10.1039/c5mb00663e.
66. Kanehisa M and Goto S. KEGG: kyoto encyclopedia of genes and genomes. Nucleic acids research. 2000;28 1:27-30. doi:10.1093/nar/28.1.27.

67. Agrawal A, Balcı H, Hanspers K, Coort SL, Martens M, Slenter DN, et al. WikiPathways 2024: next generation pathway database. Nucleic acids research. 2024;52 D1:D679-d89. doi:10.1093/nar/gkad960.
68. Milacic M, Beavers D, Conley P, Gong C, Gillespie M, Griss J, et al. The Reactome Pathway Knowledgebase 2024. Nucleic acids research. 2024;52 D1:D672-d8. doi:10.1093/nar/gkad1025.
69. Bindea G, Mlecnik B, Hackl H, Charoentong P, Tosolini M, Kirilovsky A, et al. ClueGO: a Cytoscape plug-in to decipher functionally grouped gene ontology and pathway annotation networks. Bioinformatics (Oxford, England). 2009;25 8:1091-3. doi:10.1093/bioinformatics/btp101.
70. Szklarczyk D, Kirsch R, Koutrouli M, Nastou K, Mehryary F, Hachilif R, et al. The STRING database in 2023: protein-protein association networks and functional enrichment analyses for any sequenced genome of interest. Nucleic acids research. 2023;51 D1:D638-d46. doi:10.1093/nar/gkac1000.
71. Fan HC, Fu GK and Fodor SP. Expression profiling. Combinatorial labeling of single cells for gene expression cytometry. Science (New York, NY). 2015;347 6222:1258367. doi:10.1126/science.1258367.
72. Klein AM, Mazutis L, Akartuna I, Tallapragada N, Veres A, Li V, et al. Droplet barcoding for single-cell transcriptomics applied to embryonic stem cells. Cell. 2015;161 5:1187-201. doi:10.1016/j.cell.2015.04.044.
73. Buenrostro JD, Wu B, Litzenburger UM, Ruff D, Gonzales ML, Snyder MP, et al. Single-cell chromatin accessibility reveals principles of regulatory variation. Nature. 2015;523 7561:486-90. doi:10.1038/nature14590.
74. Satpathy AT, Granja JM, Yost KE, Qi Y, Meschi F, McDermott GP, et al. Massively parallel single-cell chromatin landscapes of human immune cell development and intratumoral T cell exhaustion. Nat Biotechnol. 2019;37 8:925-36. doi:10.1038/s41587-019-0206-z.
75. Baek S and Lee I. Single-cell ATAC sequencing analysis: From data preprocessing to hypothesis generation. Comput Struct Biotechnol J. 2020;18:1429-39. doi:10.1016/j.csbj.2020.06.012.
76. The COVID-19 Host Genetics Initiative, a global initiative to elucidate the role of host genetic factors in susceptibility and severity of the SARS-CoV-2 virus pandemic. Eur J Hum Genet. 2020;28 6:715-8. doi:10.1038/s41431-020-0636-6.
77. Aquino RS and Park PW. Glycosaminoglycans and infection. Front Biosci (Landmark Ed). 2016;21 6:1260-77. doi:10.2741/4455.
78. Clausen TM, Sandoval DR, Spliid CB, Pihl J, Perrett HR, Painter CD, et al. SARS-CoV-2 Infection Depends on Cellular Heparan Sulfate and ACE2. Cell. 2020;183 4:1043-57.e15. doi:10.1016/j.cell.2020.09.033.
79. Kim SY, Jin W, Sood A, Montgomery DW, Grant OC, Fuster MM, et al. Characterization of heparin and severe acute respiratory syndrome-related coronavirus 2 (SARS-CoV-2) spike glycoprotein binding interactions. Antiviral Res. 2020;181:104873. doi:10.1016/j.antiviral.2020.104873.

80. Möller S, Theiß J, Deinert TIL, Golat K, Heinze J, Niemeyer D, et al. High-Sulfated Glycosaminoglycans Prevent Coronavirus Replication. Viruses. 2022;14 2 doi:10.3390/v14020413.
81. Kwon PS, Oh H, Kwon SJ, Jin W, Zhang F, Fraser K, et al. Sulfated polysaccharides effectively inhibit SARS-CoV-2 in vitro. Cell Discov. 2020;6 1:50. doi:10.1038/s41421-020-00192-8.
82. Parker WD, Jr., Parks J, Filley CM and Kleinschmidt-DeMasters BK. Electron transport chain defects in Alzheimer's disease brain. Neurology. 1994;44 6:1090-6. doi:10.1212/wnl.44.6.1090.
83. Zattoni M, Mearelli M, Vanni S, Colini Baldeschi A, Tran TH, Ferracin C, et al. Serpin Signatures in Prion and Alzheimer's Diseases. Mol Neurobiol. 2022;59 6:3778-99. doi:10.1007/s12035-022-02817-3.
84. Tian Y, Tian X, Han X, Chen Y, Song CY, Jiang WJ, et al. ABCE1 plays an essential role in lung cancer progression and metastasis. Tumour Biol. 2016;37 6:8375-82. doi:10.1007/s13277-015-4713-3.
85. Sivakumar S, Moore JA, Montesion M, Sharaf R, Lin DI, Colón CI, et al. Integrative Analysis of a Large Real-World Cohort of Small Cell Lung Cancer Identifies Distinct Genetic Subtypes and Insights into Histologic Transformation. Cancer Discov. 2023;13 7:1572-91. doi:10.1158/2159-8290.Cd-22-0620.
86. Wang H, Wang X, Xu L, Lin Y and Zhang J. CCT6A and CHCHD2 Are Coamplified with EGFR and Associated with the Unfavorable Clinical Outcomes of Lung Adenocarcinoma. Dis Markers. 2022;2022:1560199. doi:10.1155/2022/1560199.
87. Catelain C, Pailler E, Oulhen M, Faugeroux V, Pommier AL and Farace F. Detection of Gene Rearrangements in Circulating Tumor Cells: Examples of ALK-, ROS1-, RET-Rearrangements in Non-Small-Cell Lung Cancer and ERG-Rearrangements in Prostate Cancer. Adv Exp Med Biol. 2017;994:169-79. doi:10.1007/978-3-319-55947-6_9.
88. Wei Y, Vellanki RN, Coyaud É, Ignatchenko V, Li L, Krieger JR, et al. CHCHD2 Is Coamplified with EGFR in NSCLC and Regulates Mitochondrial Function and Cell Migration. Mol Cancer Res. 2015;13 7:1119-29. doi:10.1158/1541-7786.Mcr-14-0165-t.
89. Riely GJ, Marks J and Pao WJPotATS. KRAS mutations in non–small cell lung cancer. 2009;6 2:201-5.
90. Wang Z, Yang M-Q, Lei L, Fei L-R, Zheng Y-W, Huang W-J, et al. Overexpression of KRT17 promotes proliferation and invasion of non-small cell lung cancer and indicates poor prognosis. 2019:7485-97.
91. Shi X, Zhan L, Xiao C, Lei Z, Yang H, Wang L, et al. miR-1238 inhibits cell proliferation by targeting LHX2 in non-small cell lung cancer. 2015;6 22:19043.
92. Carpagnano GE, Palladino GP, Lacedonia D, Koutelou A, Orlando S and Foschino-Barbaro MPJBc. Neutrophilic airways inflammation in lung cancer: the role of exhaled LTB-4 and IL-8. 2011;11 1:1-9.
93. Luo L, Zheng Y, Lin Z, Li X, Li X, Li M, et al. Identification of SHMT2 as a Potential Prognostic Biomarker and Correlating with Immune Infiltrates in Lung Adenocarcinoma.

J Immunol Res. 2021;2021:6647122. doi:10.1155/2021/6647122.
94. Hoek KS, Schlegel NC, Brafford P, Sucker A, Ugurel S, Kumar R, et al. Metastatic potential of melanomas defined by specific gene expression profiles with no BRAF signature. Pigment cell research. 2006;19 4:290-302. doi:10.1111/j.1600-0749.2006.00322.x.
95. Hoek KS and Goding CR. Cancer stem cells versus phenotype-switching in melanoma. Pigment cell & melanoma research. 2010;23 6:746-59. doi:10.1111/j.1755-148X.2010.00757.x.
96. Aiello-Couzo NM and Kang Y. A bridge between melanoma cell states. Nature cell biology. 2020;22 8:913-4. doi:10.1038/s41556-020-0556-2.
97. Centeno PP, Pavet V and Marais R. The journey from melanocytes to melanoma. Nature reviews Cancer. 2023;23 6:372-90. doi:10.1038/s41568-023-00565-7.
98. Pearlman RL, Montes de Oca MK, Pal HC and Afaq F. Potential therapeutic targets of epithelial-mesenchymal transition in melanoma. Cancer letters. 2017;391:125-40. doi:10.1016/j.canlet.2017.01.029.
99. Capparelli C, Purwin TJ, Glasheen M, Caksa S, Tiago M, Wilski N, et al. Targeting SOX10-deficient cells to reduce the dormant-invasive phenotype state in melanoma. Nature communications. 2022;13 1:1381. doi:10.1038/s41467-022-28801-y.
100. Jia P and Zhao Z. Network.assisted analysis to prioritize GWAS results: principles, methods and perspectives. Human genetics. 2014;133 2:125-38. doi:10.1007/s00439-013-1377-1.
101. Menche J, Sharma A, Kitsak M, Ghiassian SD, Vidal M, Loscalzo J, et al. Uncovering disease-disease relationships through the incomplete interactome. Science (New York, NY). 2015;347 6224:1257601. doi:10.1126/science.1257601.
102. Han X, Zhou Z, Fei L, Sun H, Wang R, Chen Y, et al. Construction of a human cell landscape at single-cell level. Nature. 2020;581 7808:303-9. doi:10.1038/s41586-020-2157-4.
103. Tarhan L, Bistline J, Chang J, Galloway B, Hanna E and Weitz E. Single Cell Portal: an interactive home for single-cell genomics data. bioRxiv : the preprint server for biology. 2023; doi:10.1101/2023.07.13.548886.
104. Lin S and Jia P: scGraph2Vec: a deep generative model for gene embedding augmented by Graph Neural Network and single-cell omics data [Data set]. Zenodo. 2024. https://doi.org/10.5281/zenodo.12092871 (2024). Accessed Jun 18 2024.
105. Lin S and Jia P: GitHub repository. https://github.com/LPH-BIG/scGraph2Vec (2024).
106. DOME annotation: scGraph2Vec. https://dome.dsw.elixir-europe.org/wizard/projects/bfa1f32e-b569-4fe3-8cb5-1387e80bb051. Accessed Jun 18 2024.

FIGURE LEGENDS

Figure 1: Schematic description of scGraph2Vec. **(A)** Feature extraction. The input of scGraph2Vec is an adjacency matrix from the gene interaction network and a feature matrix from single-cell data. The membership matrix derived from the primary community is calculated by the adjacency matrix using the Louvain algorithm and is added to the adjacency matrix. The model adopts the modified VGAE framework. The encoder is composed of a 3-layer GCN network, and the decoder considers both the reconstruction of the original dimension and the detection of the node community. The latent features were extracted as model output and reduced to two dimensions by t-SNE. Gene clusters were obtained using a hierarchical clustering algorithm. (**B)** The inference of disease-associated genes. Seed genes can be defined using GWAS data or known driver genes. Candidate disease-associated genes can be identified using latent features extracted from disease-related tissues.

Figure 2: Performance comparison between different methods. **(A)** Comparison of silhouette coefficients among standard outputs of scGraph2Vec and other variants using brain and PBMC datasets. The X-axis is the number of clusters. The Y-axis is the silhouette coefficients. **(B, C)** Comparison of silhouette coefficients among scGraph2Vec and 11 tools using brain (B) and PBMC (C) datasets. The method scapGNN and SAUCIE were excluded as it failed to extract clear gene clusters. The X-axis is the number of clusters. The Y-axis is the silhouette coefficients and DBI. **(D)** Comparison of gene clustering similarity across methods using the Jaccard Index. **(E)** Comparison of the similarity between gene clusters from different methods and the MSigDB

hallmark gene sets.

Figure 3: Biological implications of latent features from brain tissue. (A) Comparison of internal and external closeness centralities for all gene clusters using the Wilcoxon rank-sum test. Each dot represents a gene cluster. **(B)** The distribution of housekeeping genes in brain embeddings. **(C)** The gene clusters significantly enriched in hallmark gene sets (Fisher's exact test, $P < 0.05/50$). **(D)** The left plot is the t-SNE plot of brain scRNA-seq data. The color indicates cell types labeled from the original article. The right plot shows the module score of each cell for brain cluster 9.

Figure 4: Tissue-specific clusters and functional analysis. (A) The heatmap of normalized enrichment score (NES) from GSEA results. Only significant enriched clusters and cell types are highlighted in color ($P_{BH} < 0.05$). **(B)** The GSEA enrichment results for cluster 9 and two cell types (i.e., Ast and In1c). **(C)** GO enrichment for cluster 9. **(D)** GO enrichment for cluster 59. **(E)** The heatmap plot shows the brain-specific gene clusters. **(F)** The predicted subnetwork (left) and GO enrichment (right) of *SMARCE1* neighbor genes from embeddings of brain tissue. **(G)** The predicted subnetwork (left) and GO enrichment (right) of *SMARCE1* neighbor genes from embeddings of lung tissue.

Figure 5: Inference of COVID-19-associated genes. (A) Volcano plot for GWAS-reported genes. The X-axis represents the log2(FC) of genes in COVID-19 and non-COVID-19 groups.

The Y-axis shows the gene-based p-value from the MAGMA analysis (Bonferroni correction). The dashed lines represent the thresholds of significantly associated genes ($P < 2.63 \times 10^{-6}$) and differential expression genes (|log2(FC)| > 1). (**B, C)** Differential expression analysis of COVID-19-associated genes inferred from lung embeddings (B) and brain embeddings (C). The dashed lines represent the threshold of significant DEGs ($P_{BH} < 0.05$, |log2(FC)| > 1). **(D)** The expression profiles for COVID-19-associated candidate genes inferred from lung tissue. The samples in the columns were grouped by COVID-19 positive samples and COVID-19 negative samples, and the genes on the rows were clustered according to Ward's method. **(E)** GO enrichment of all candidate genes (left) and *CEACAM8* neighbor genes (right) from lung tissue. **(F)** Pathway enrichment of all candidate genes based on the KEGG, WikiPathways and Reactome databases. **(G)** STRING analysis for the candidate genes involved in the enriched pathways.

Figure 6: Inference of AD-associated genes. (A) Volcano plot for GWAS-reported genes. The X-axis represents the log2 (FC) of genes in AD and non-AD groups. The Y-axis shows the gene-based p-value by MAGMA (after Bonferroni correction). The dashed lines represent the threshold of significant associated genes ($P < 3.77 \times 10^{-6}$) and differential expression gene threshold (|fold change| > 1.1). (**B, C)** Differential expression analysis AD-associated genes inferred from brain embeddings (B) and lung embeddings (C). The dashed lines represent the threshold of significant DEGs ($P_{BH} < 0.05$, |fold change| > 1.1). **(D)** AD-associated gene expression profiles inferred from brain tissue. The samples in the columns were grouped by AD positive samples and AD negative samples, and the genes on the rows were clustered according to Ward's method. **(E)** GO analysis

of all neighbor genes (left) and *APOE* neighbor genes (right) from brain tissue. **(F)** Pathway enrichment of all candidate genes based on the KEGG, WikiPathways and Reactome databases. **(G)** Pathway annotation for all candidate genes using the ClueGO software.

Figure 7: Discovery of additional tumor-driver genes. (A) The heatmap plot of the tumor-specific gene clusters. **(B)** The most significant GO terms enriched for tumor-specific clusters. Only biological processes were shown. **(C)** The top 10 GO terms (biological process) enriched in *TP53* neighbor genes (sorted by P_{BH}). **(D)** Boxplots showing the expression differences of *TP53* neighbor genes in TCGA LUAD samples. The P values were from the Wilcoxon test using log2 (TPM + 1). *, $P < 0.05$; **, $P < 0.01$; ***, $P < 0.001$; ****, $P < 0.0001$. **(E)** Venn diagram showing the overlap of the four gene sets: up-regulated DEGs, down-regulated DEGs, genes with hazard ratio (HR) > 1, and genes with HR < 1. **(F, G)** Kaplan-Meier curve based on the survival time (days) of TCGA LUAD samples and the expression levels of *ABCE1* and *LTB* genes. Samples were grouped based on the median of log2 (TPM + 1).

Figure 8: Discovery of regulatory genes for cell state transitions. (A) Venn diagram of the overlapping genes among three types of clusters: clusters specific to cell states, clusters with significant differences in AUCell scores between states, and clusters with enriched functional signatures. **(B)** The enrichment results for 14 functional state signatures and gene clusters from 3 cell states (Fisher's exact test, $P < 0.05/14$). **(C)** Cellular activity of gene clusters in three states measured by AUCell scores. One-way ANOVA was used to test across multiple cell states and a

two-sample t-test was used for comparisons between cell states. *, $P < 0.05$; **, $P < 0.01$; ***, $P < 0.001$; ****, $P < 0.0001$. **(D)** Expression distribution of *SOX10* in different cell lines. **(E)** Differential expression analysis of *SOX10* neighbor genes inferred from melanocytic embeddings (left) and intermediate embeddings (right). The dashed lines represent the threshold of significant DEGs ($P < 0.05$, $|\log2(FC)| > 1$). **(F)** Comparison of cell activity (AUCell) changes between normal and *SOX10*-KD samples. The two-sample t-test was used to compare the activity between normal and *SOX10*-KD samples. *, $P < 0.05$; **, $P < 0.01$; ***, $P < 0.001$; ****, $P < 0.0001$. **(G)** Stacked violin plot showing expression of validated *SOX10* neighboring genes in scRNA-seq data.

Figure1.R1

Click here to access/download;Figure;Figure1.R1.pdf

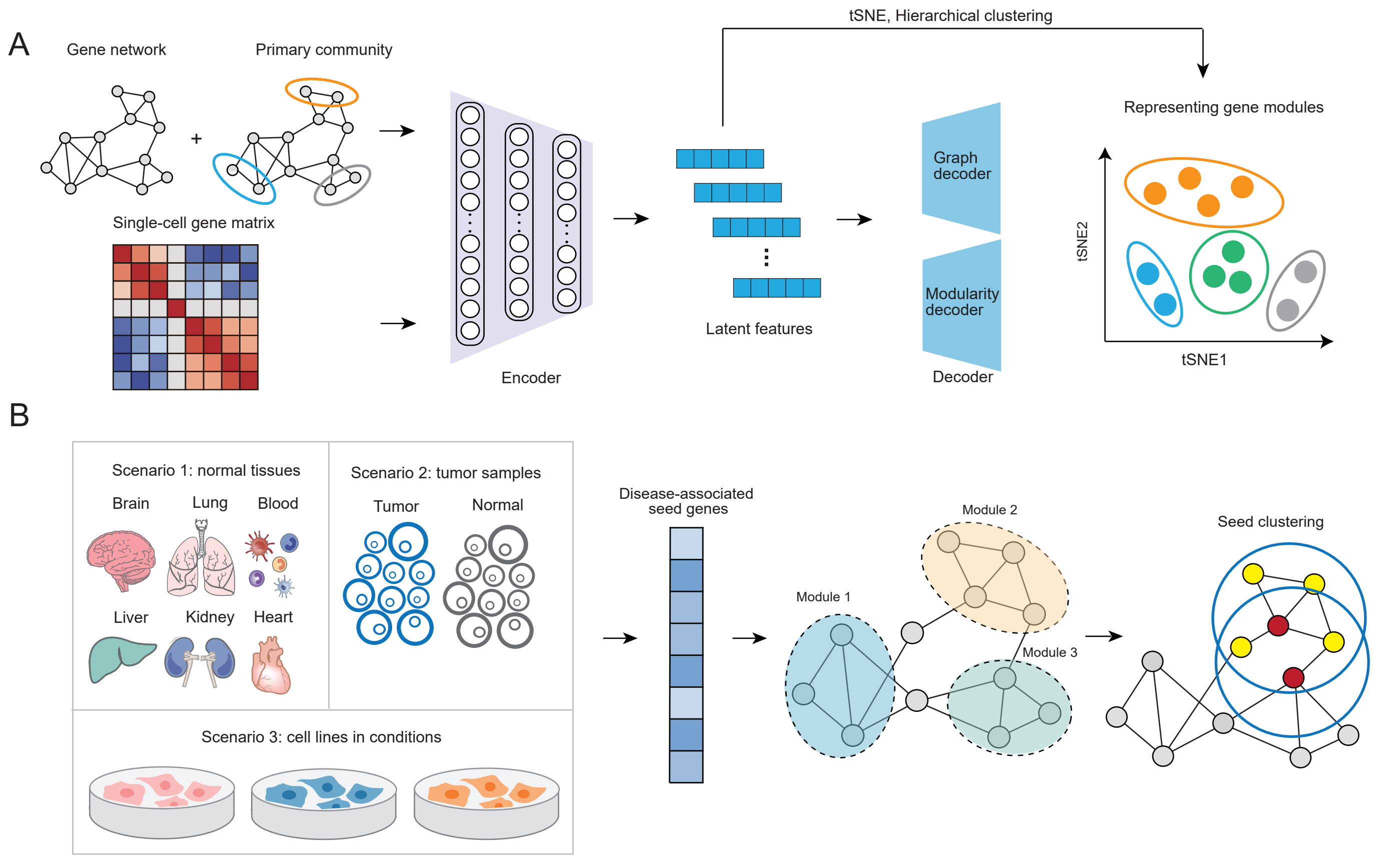

Figure2.R1

Click here to access/download;Figure;Figure2.R1.pdf

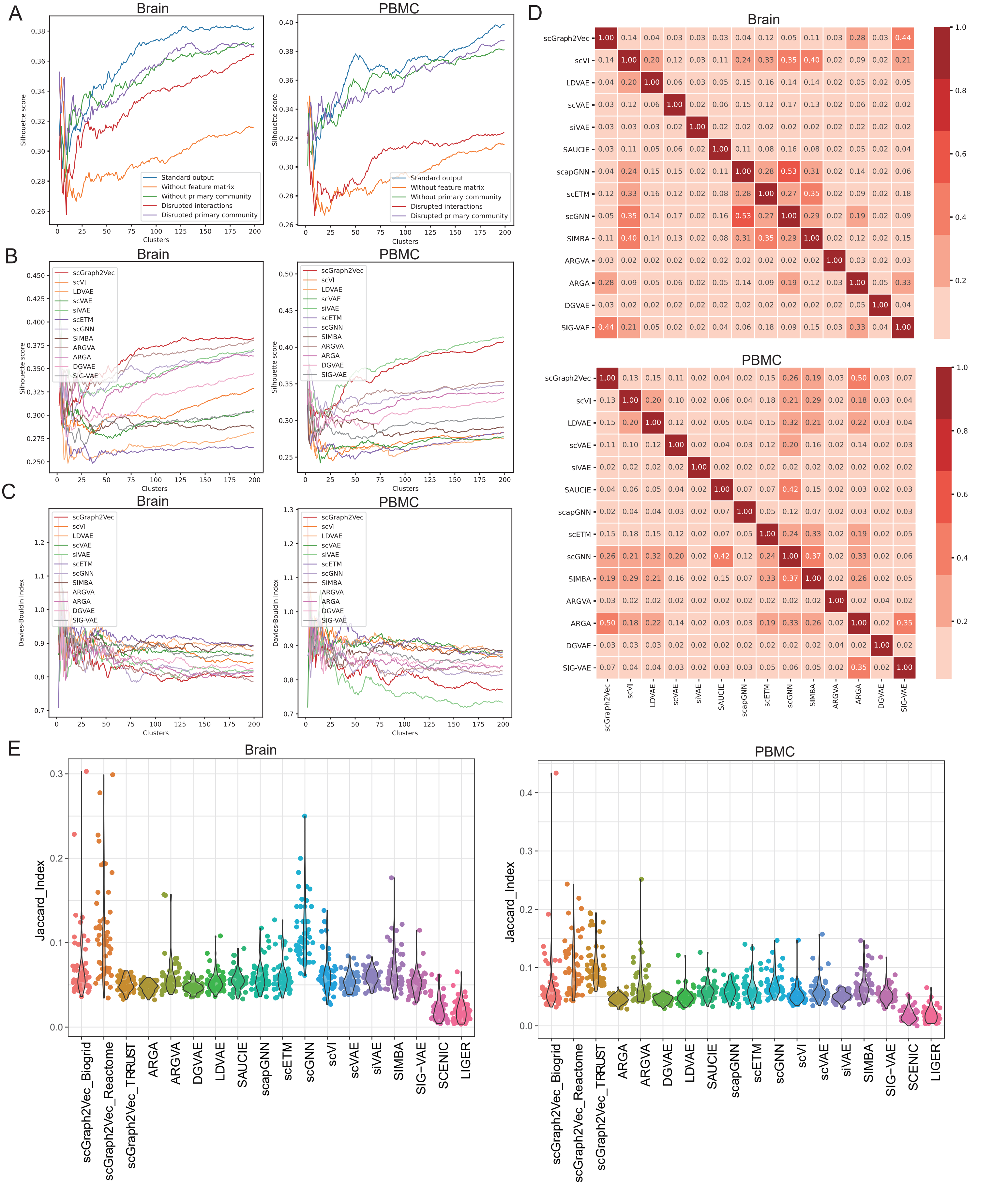

Figure3.R3

Click here to access/download;Figure;Figure3.R1.pdf

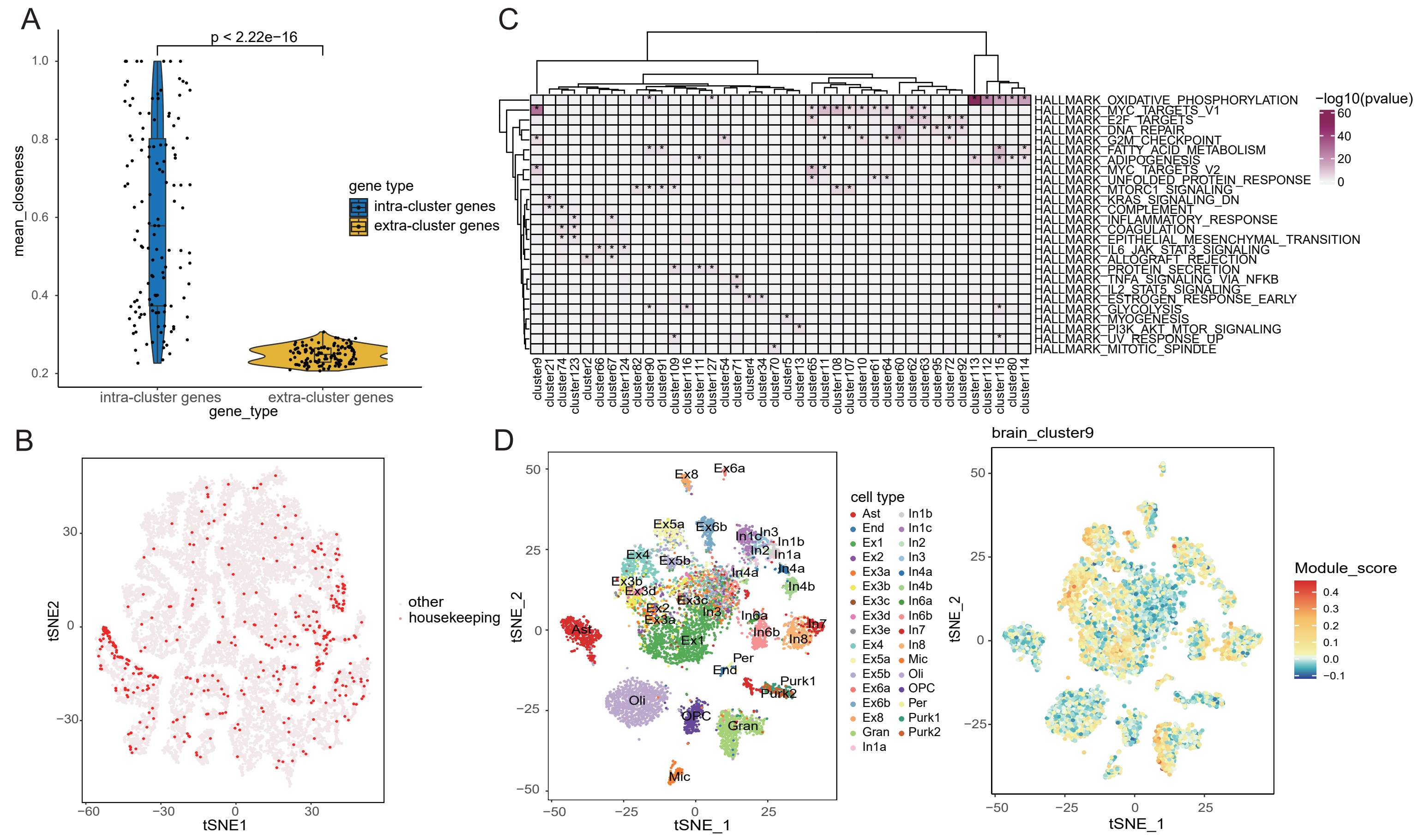

Figure4.R1

Click here to access/download;Figure;Figure4.R1.pdf

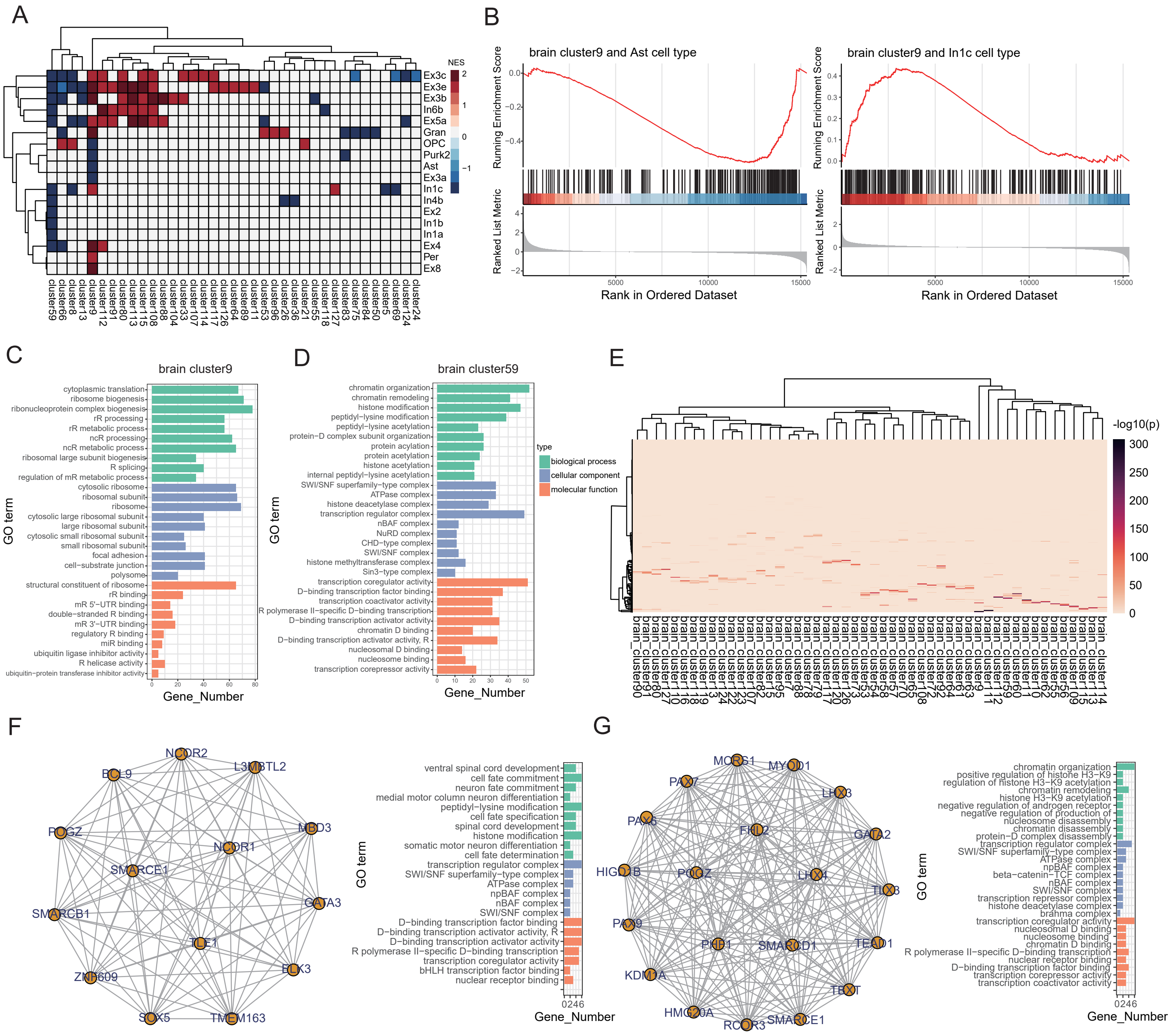

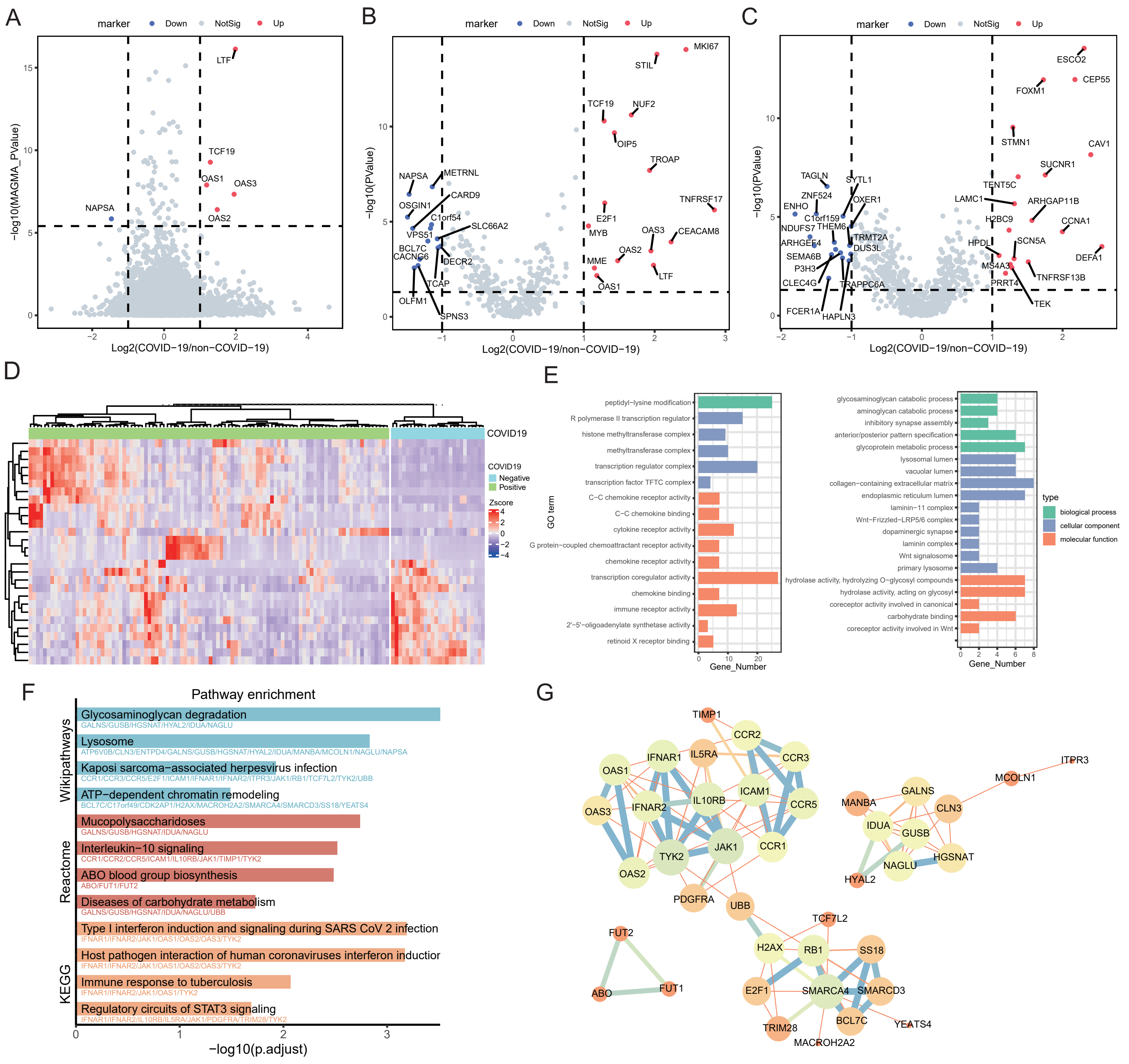
A
B
C
marker
Down
NotSig
Up
-log10(MAGMA_PValue)
-log10(PValue)
Log2(COVID−19/non−COVID−19)
LTF
TCF19
OAS1
OAS3
OAS2
NAPSA
MKI67
STIL
NUF2
OIP5
TROAP
TNFRSF17
E2F1
MYB
CEACAM8
MME
OAS1
METRNL
CARD9
OSGIN1
C1orf54
SLC66A2
VPS51
BCL7C
CACNG6
DECR2
TCAP
OLFM1
SPNS3
ESCO2
CEP55
FOXM1
STMN1
CAV1
SUCNR1
TENT5C
LAMC1
ARHGAP11B
H2BC9
CCNA1
HPDL
SCN5A
DEFA1
MS4A3
TNFRSF13B
PRRT4
TEK
TAGLN
SYTL1
ZNF524
OXER1
ENHO
C1orf159
NDUFS7
THEM6
TRMT2A
ARHGEF4
DUS3L
SEMA6B
P3H3
CLEC4G
TRAPPC6A
FCER1A
HAPLN3
D
COVID19
Negative
Positive
Zscore
E
GO term
Gene_Number
type
biological process
cellular component
molecular function
peptidyl−lysine modification
R polymerase II transcription regulator
histone methyltransferase complex
methyltransferase complex
transcription regulator complex
transcription factor TFTC complex
C−C chemokine receptor activity
C−C chemokine binding
cytokine receptor activity
G protein−coupled chemoattractant receptor activity
chemokine receptor activity
transcription coregulator activity
chemokine binding
immune receptor activity
2'−5'−oligoadenylate synthetase activity
retinoid X receptor binding
glycosaminoglycan catabolic process
aminoglycan catabolic process
inhibitory synapse assembly
anterior/posterior pattern specification
glycoprotein metabolic process
lysosomal lumen
vacuolar lumen
collagen−containing extracellular matrix
endoplasmic reticulum lumen
laminin−11 complex
Wnt−Frizzled−LRP5/6 complex
dopaminergic synapse
laminin complex
Wnt signalosome
primary lysosome
hydrolase activity, hydrolyzing O−glycosyl compounds
hydrolase activity, acting on glycosyl
coreceptor activity involved in canonical
carbohydrate binding
coreceptor activity involved in Wnt
F
Pathway enrichment
Wikipathways
Reactome
KEGG
Glycosaminoglycan degradation
Lysosome
Kaposi sarcoma−associated herpesvirus infection
ATP−dependent chromatin remodeling
Mucopolysaccharidoses
Interleukin−10 signaling
ABO blood group biosynthesis
Diseases of carbohydrate metabolism
Type I interferon induction and signaling during SARS CoV 2 infection
Host pathogen interaction of human coronaviruses interferon inductior
Immune response to tuberculosis
Regulatory circuits of STAT3 signaling
−log10(p.adjust)
G
TIMP1
CCR2
IL5RA
CCR3
IFNAR1
OAS1
ICAM1
IL10RB
CCR5
IFNAR2
OAS3
JAK1
TYK2
CCR1
OAS2
PDGFRA
UBB
ITPR3
MCOLN1
GALNS
MANBA
CLN3
IDUA
GUSB
NAGLU
HGSNAT
HYAL2
FUT2
ABO
FUT1
TCF7L2
H2AX
RB1
SS18
E2F1
SMARCA4
SMARCD3
TRIM28
BCL7C
YEATS4
MACROH2A2

Figure6.R1 Click here to access/download;Figure;Figure6.R1.pdf

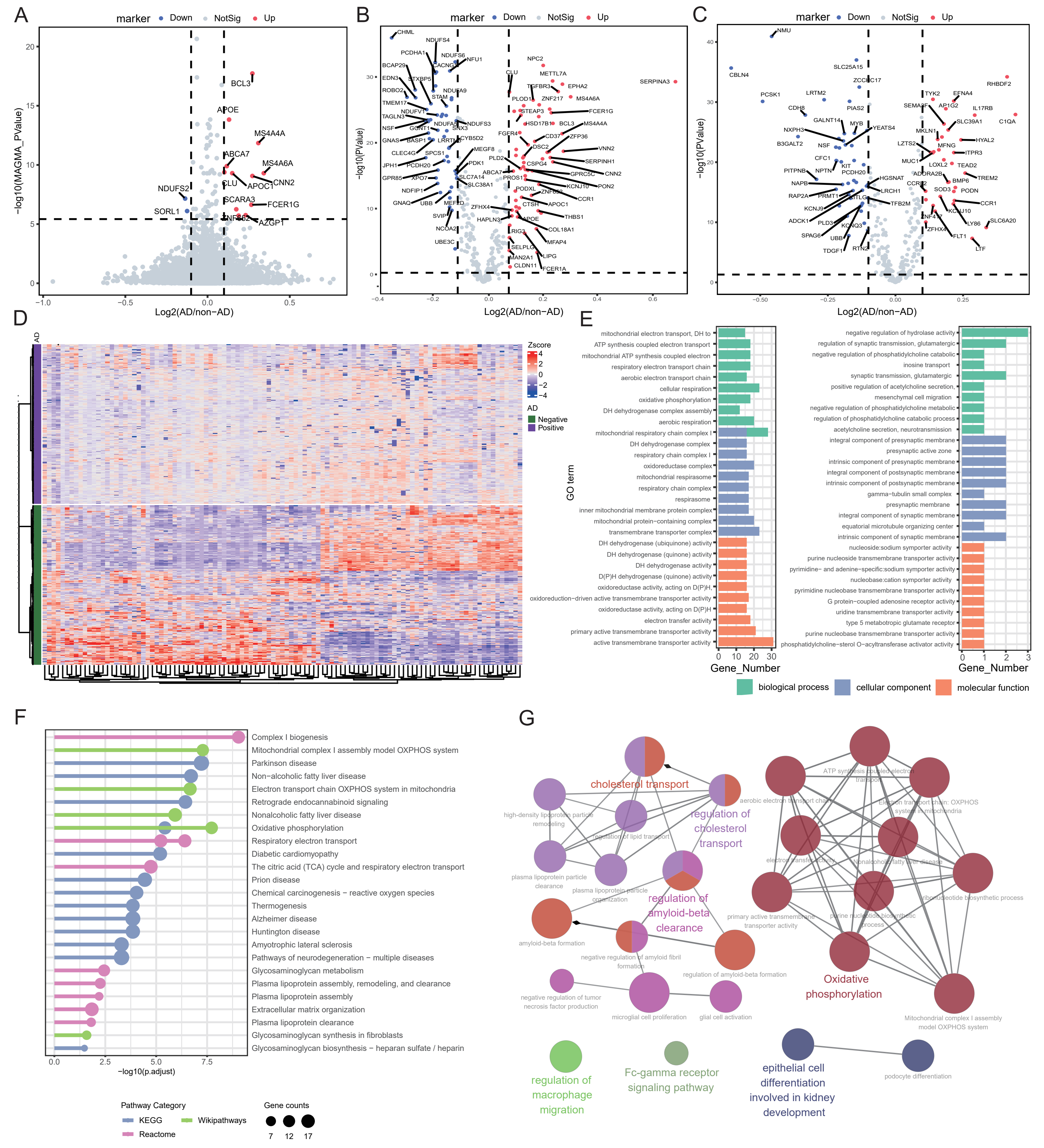

Figure7.R1

Click here to access/download;Figure;Figure7.R1.pdf

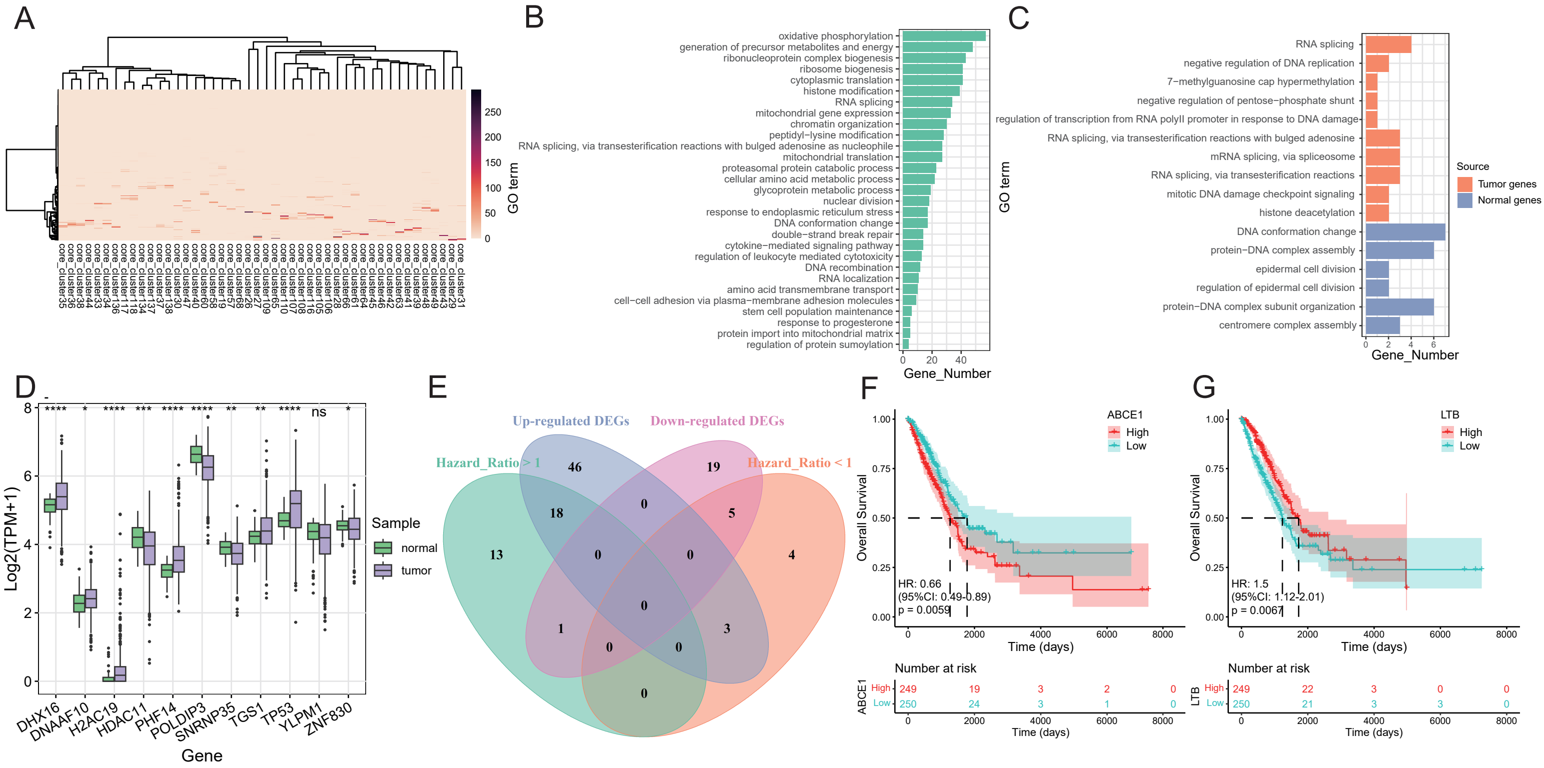

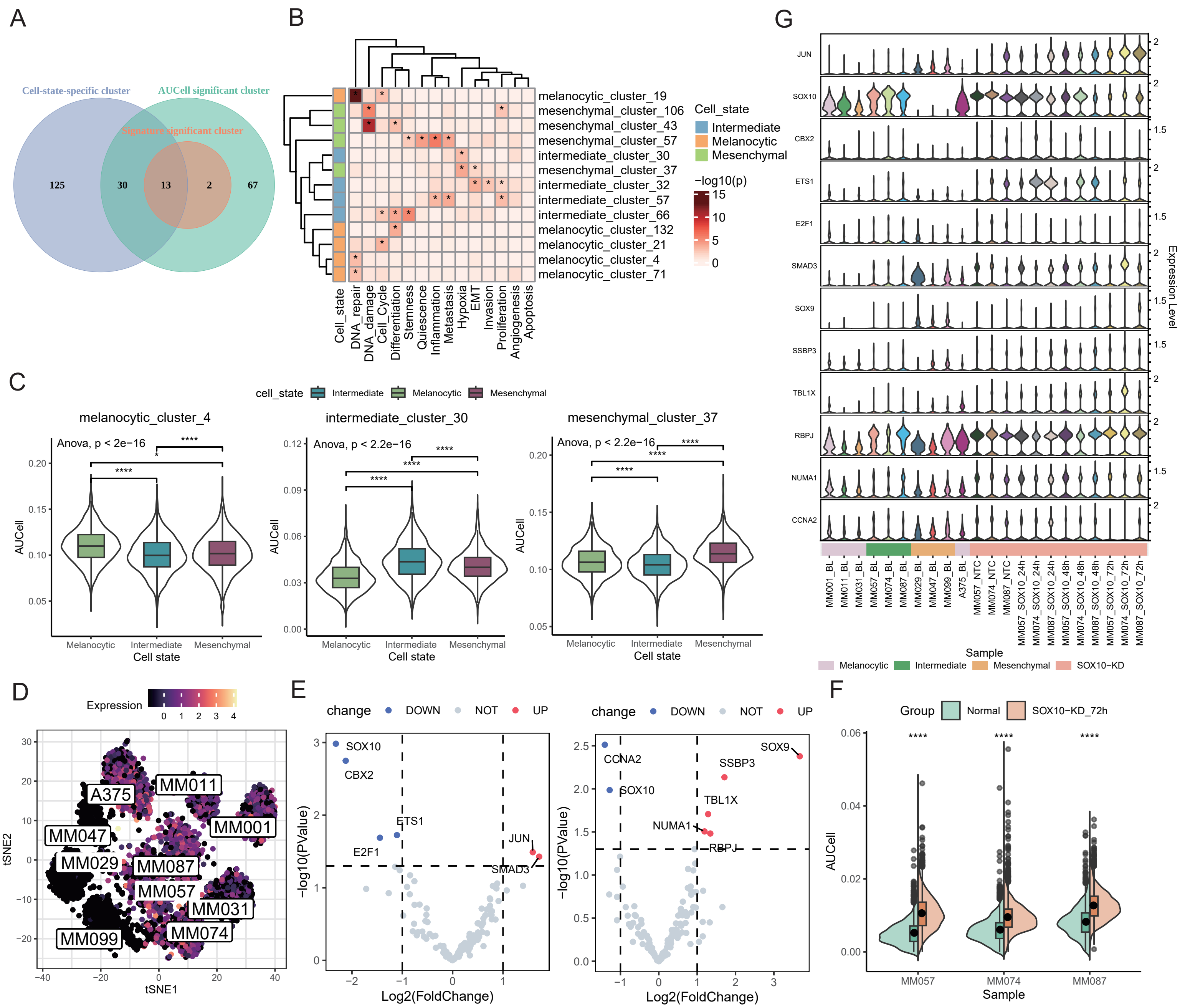
A
Cell-state-specific cluster
AUCell significant cluster
Signature significant cluster
125
30
13
2
67
B
Cell_state
Intermediate
Melanocytic
Mesenchymal
−log10(p)
15
10
5
0
melanocytic_cluster_19
mesenchymal_cluster_106
mesenchymal_cluster_43
mesenchymal_cluster_57
intermediate_cluster_30
mesenchymal_cluster_37
intermediate_cluster_32
intermediate_cluster_57
intermediate_cluster_66
melanocytic_cluster_132
melanocytic_cluster_21
melanocytic_cluster_4
melanocytic_cluster_71
DNA_repair
DNA_damage
Cell_Cycle
Differentiation
Stemness
Quiescence
Inflammation
Metastasis
Hypoxia
EMT
Invasion
Proliferation
Angiogenesis
Apoptosis
C
cell_state Intermediate Melanocytic Mesenchymal
melanocytic_cluster_4
Anova, p < 2e−16
intermediate_cluster_30
Anova, p < 2.2e−16
mesenchymal_cluster_37
Anova, p < 2.2e−16
AUCell
Cell state
Melanocytic
Intermediate
Mesenchymal
D
Expression
A375
MM011
MM001
MM047
MM029
MM087
MM057
MM031
MM099
MM074
tSNE1
tSNE2
E
change DOWN NOT UP
SOX10
CBX2
ETS1
E2F1
JUN
SMAD3
CCNA2
SOX9
SSBP3
TBL1X
NUMA1
RBPJ
−log10(PValue)
Log2(FoldChange)
F
Group Normal SOX10−KD_72h
AUCell
MM057
MM074
MM087
Sample
G
JUN
SOX10
CBX2
ETS1
E2F1
SMAD3
SOX9
SSBP3
TBL1X
RBPJ
NUMA1
CCNA2
Expression Level
MM001_BL
MM011_BL
MM031_BL
MM057_BL
MM074_BL
MM087_BL
MM029_BL
MM047_BL
MM099_BL
A375_BL
MM057_NTC
MM074_NTC
MM087_NTC
MM057_SOX10_24h
MM074_SOX10_24h
MM087_SOX10_24h
MM057_SOX10_48h
MM074_SOX10_48h
MM087_SOX10_48h
MM057_SOX10_72h
MM074_SOX10_72h
MM087_SOX10_72h
Sample
Melanocytic Intermediate Mesenchymal SOX10−KD

Click here to access/download
Supplementary Material
Supporting Information.R1.docx

Additional file2.R1

Click here to access/download
Supplementary Material
Supplementary Table.R1.xlsx

Manuscript

Click here to access/download
Supplementary Material
scGraph2Vec.response.final.docx

Cover letter
Click here to access/download;Personal Cover;scGraph2Vec.R1.coverletter.docx

国家基因组科学数据中心
National Genomics Data Center

Peilin Jia, PhD
Professor
Beijing Institute of Genomics Chinese Academy of Sciences
China National Center for Bioinformation

October 18, 2024

Dr. Nicole Nogoy
Editor, GigaScience

Dear Dr. Nogoy,

Here, we submit a revised manuscript (GIGA-D-24-00200) entitled “scGraph2Vec: a deep generative model for gene embedding augmented by Graph Neural Network and single-cell omics data” for your consideration in GigaScience.

We thank the valuable comments from the editor and the reviewers. We have fully addressed all the reviewers’ comments and provided a point-to-point response in the response letter. Briefly, we conducted the following analyses during revision:

1. Substantially expand the comparison with other methods. In total, we benchmarked 15 methods, including 6 newly added during revision per the request by the reviewers. The results showed that scGraph2Vec produced gene embeddings that distinguished different gene clusters more clearly compared to other embedding algorithms. Moreover, the clusters by scGraph2Vec contained a higher proportion of genes with known functions.

2. We included two more reference networks to validate scGraph2Vec. In total, we evaluated scGraph2Vec using three common biological networks, each representing distinct network properties: protein-protein interaction (PPI) network, transcriptional regulatory network, and metabolic pathway network. The results indicated that scGraph2Vec is highly effective at capturing small, locally connected clusters, making it particularly suited to scale-free networks characterized by sparse connections, a feature common to most biological networks.

3. For the gene embeddings, clusters, and candidate genes identified by scGraph2Vec, we included more functional annotations to demonstrate their biological relevance, including network and pathway analysis, benchmark with the hallmark genes and housekeeping genes, and comparison with cell-type and tissue-specific genes.

4. We added more application cases to demonstrate scGraph2Vec. Specifically, we inferred the regulatory genes involved in melanoma cell state transitions.

We hope the revised manuscript are satisfactory to the editor and the reviewers. Thank you in advance for your kind consideration. If you have any questions, please feel free to let us know.

Yours sincerely,
Peilin Jia, Ph.D.
Professor
National Genomics Data Center
Beijing Institute of Genomics (China National Center for Bioinformation)
Chinese Academy of Sciences, Beijing 100101, China
Email: pjia@big.ac.cn
