# Supplementary material for: scGraph2Vec: a deep generative model for gene embedding augmented by graph neural network and single-cell omics data
Source: Gigascience. 2024 Dec 20;13:giae108. doi: 10.1093/gigascience/giae108 (PMC11659981; doi:10.1093/gigascience/giae108)
Supplement: giae108_Supplemental_Files [file giae108_supplemental_files.zip › Supporting Information.R2.docx]

**Supplementary material**

**scGraph2Vec: a deep generative model for gene embedding augmented by Graph Neural Network and single-cell omics data**

Shiqi Lin^1,2,3^, Peilin Jia^1,2,3*^

^1^National Genomics Data Center, China National Center for Bioinformation, Beijing, 100101, China

^2^Beijing Institute of Genomics, Chinese Academy of Sciences, Beijing, 100101, China

^3^University of Chinese Academy of Sciences, Beijing, 100049, China

*Address correspondence to:

Peilin Jia, Ph.D.

Email: pjia@big.ac.cn

**Supplementary Table 1. Statistics of the datasets used.**

**Supplementary Table S2. Summary of methods used for comparison.** DNN, deep neural networks; structure; VAE, variational autoencoder; ETM, embedded topic model; GNN, graph neural network.

**Supplementary Table 3.** **Gene clusters identified by scGraph2Vec in 6 representative human tissues, tumor/normal tissues of LUAD and 3 melanocyte cell states.** The first column lists the gene symbols, and the subsequent columns show the cluster obtained through hierarchical clustering of gene embeddings extracted by scGraph2Vec.

**Supplementary Table 4.** **The results of applying GSEA to identify potential enrichment of gene clusters among brain cell type-specific genes.** The set size is the number of cluster genes after conditional screening. The normalized enrichment score (NES) considered the number of gene cluster, and the value of NES represents the degree of enrichment of cluster genes in the overall DEGs of a specific cell type. The p-value, p.adjust, and q-value represent the significance of the enrichment results. The rank means the position of the corresponding gene is in the sorted DEGs when the enrichment score is maximum. The core enrichment is the enriched core genes in cluster.


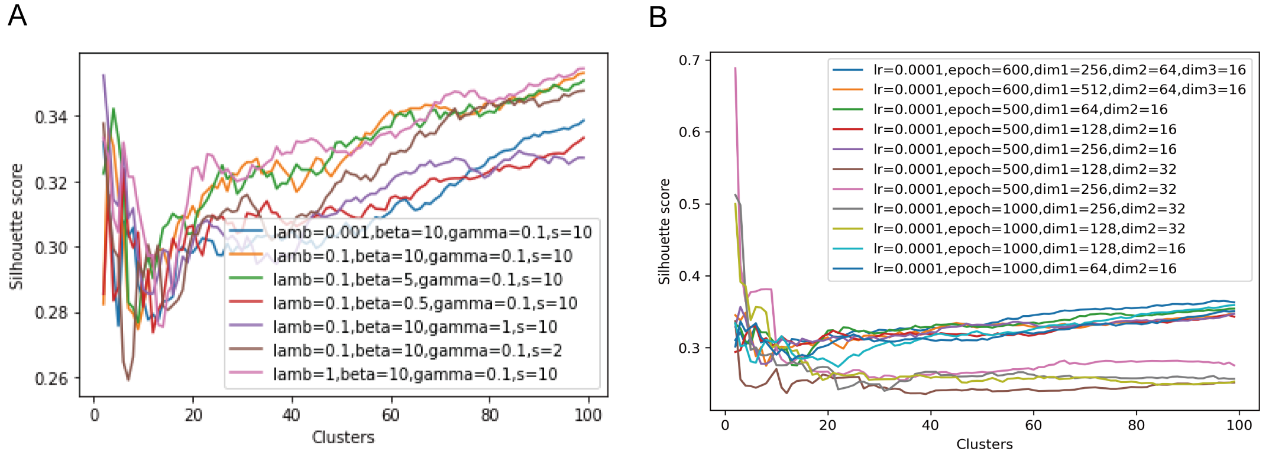


**Supplementary Figure 1. The hyperparameter sweep result**. Hyperparameter sweeping for model-specific parameters (A) and general parameters (B). The X-axis represents the number of gene clusters detected and the Y-axis represents the Silhouette coefficient with different numbers of clusters.


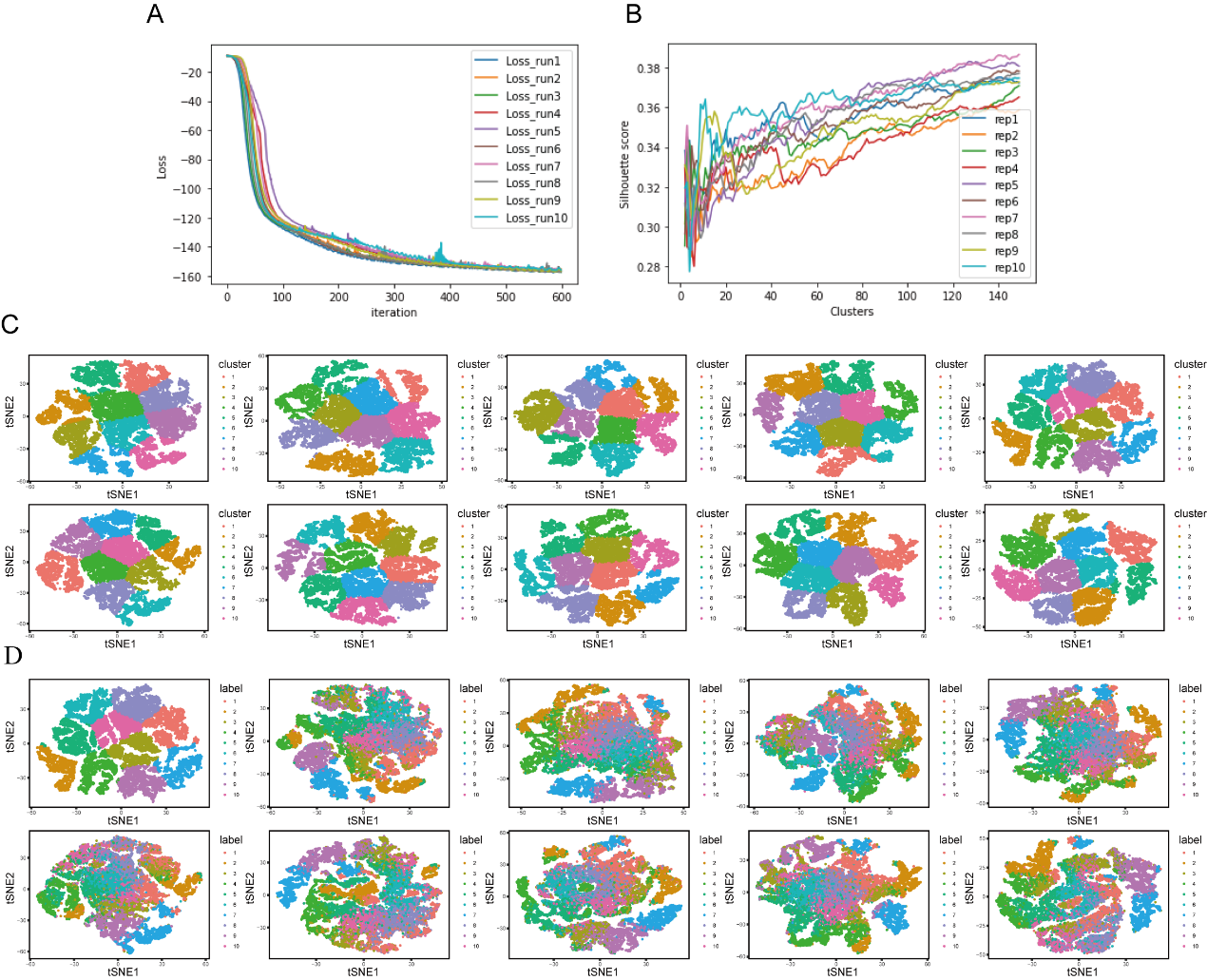


**Supplementary Figure 2. The evaluation of model stability in multiple ways.** The brain dataset was trained 10 times using the same parameters. **A.** The distribution of the loss values. **B.** The distribution of the silhouette coefficients. **C.** The cluster partitions in 10 replications. **D.** Using the clusters identified from “rep 5” to label genes from other replications.


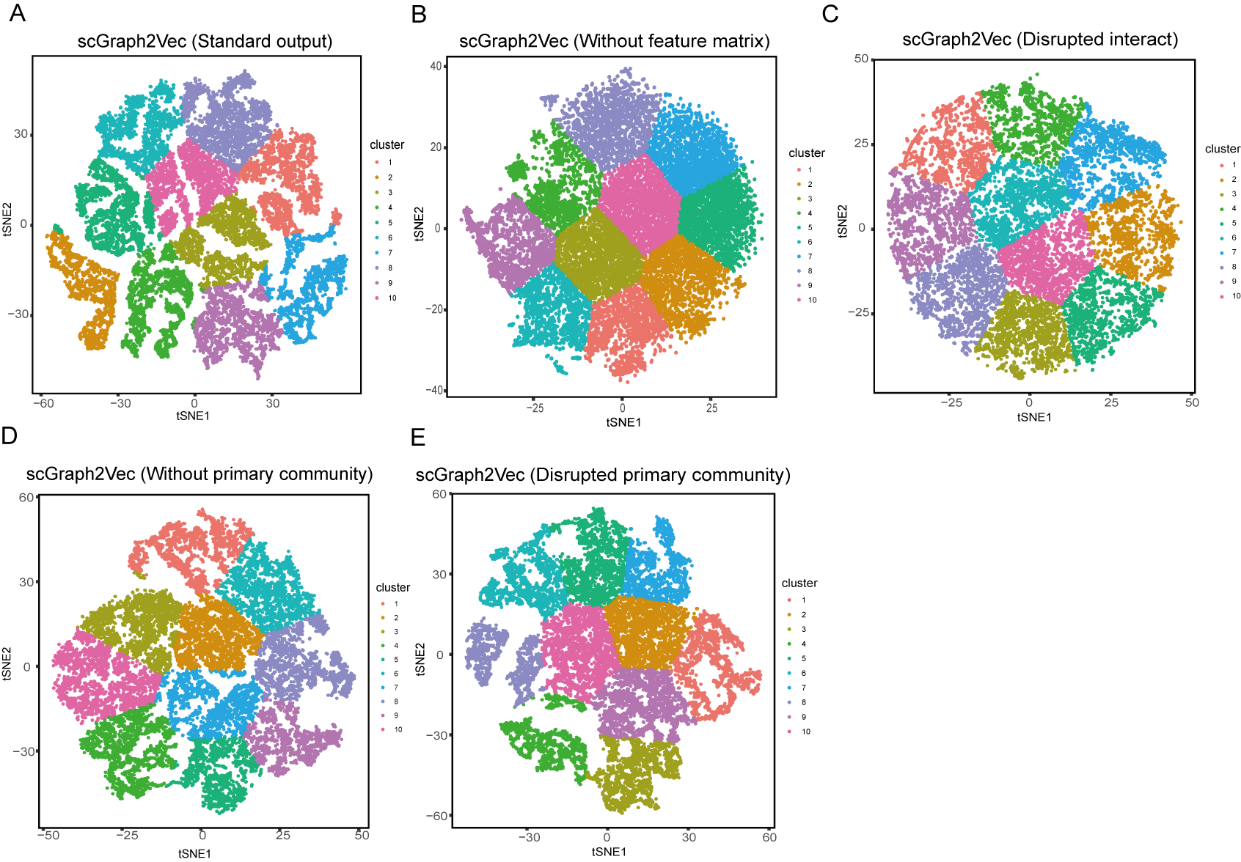


**Supplementary Figure 3. Performance evaluation of the key components in scGraph2Vec by varying its structure using brain dataset.**


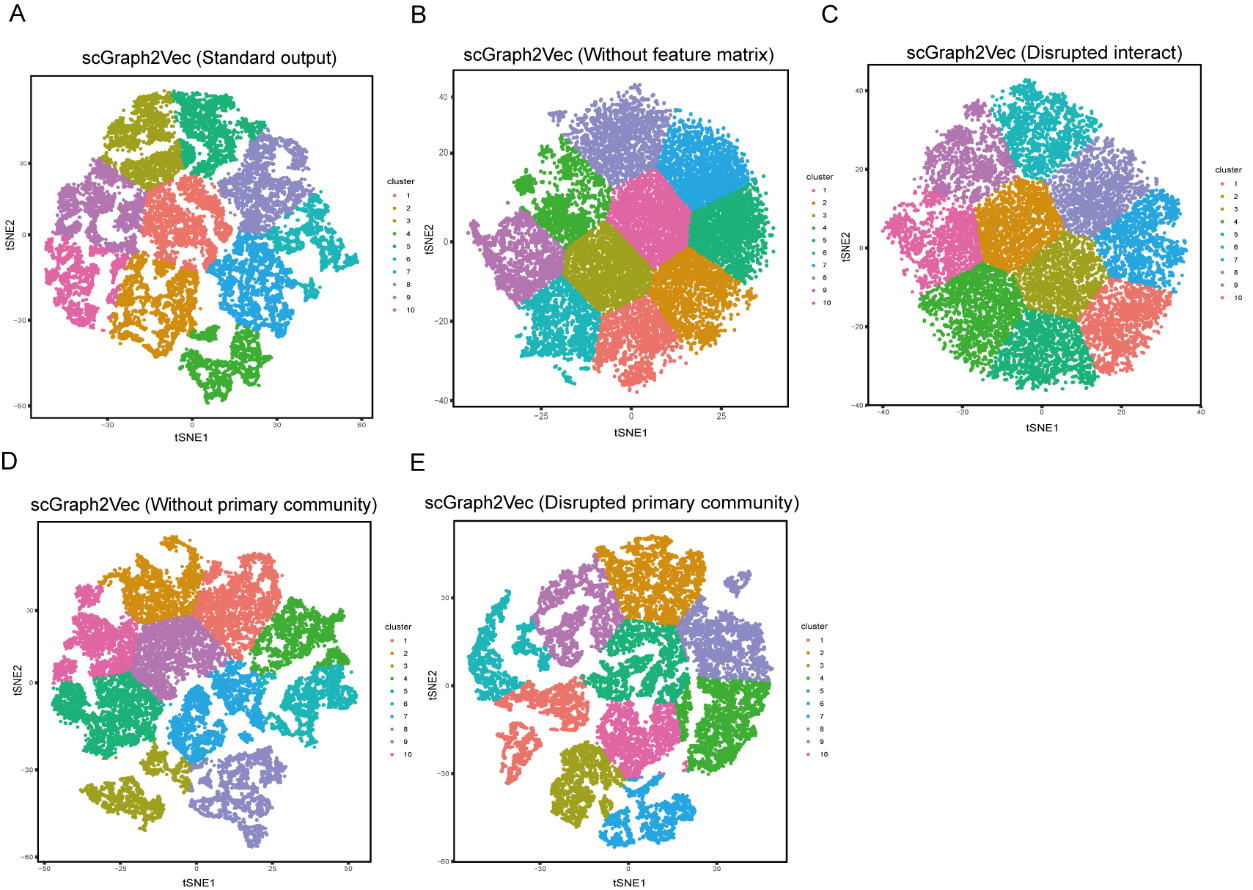


**Supplementary Figure 4. Performance evaluation of the key components in scGraph2Vec by varying its structure using PBMC dataset.**

**
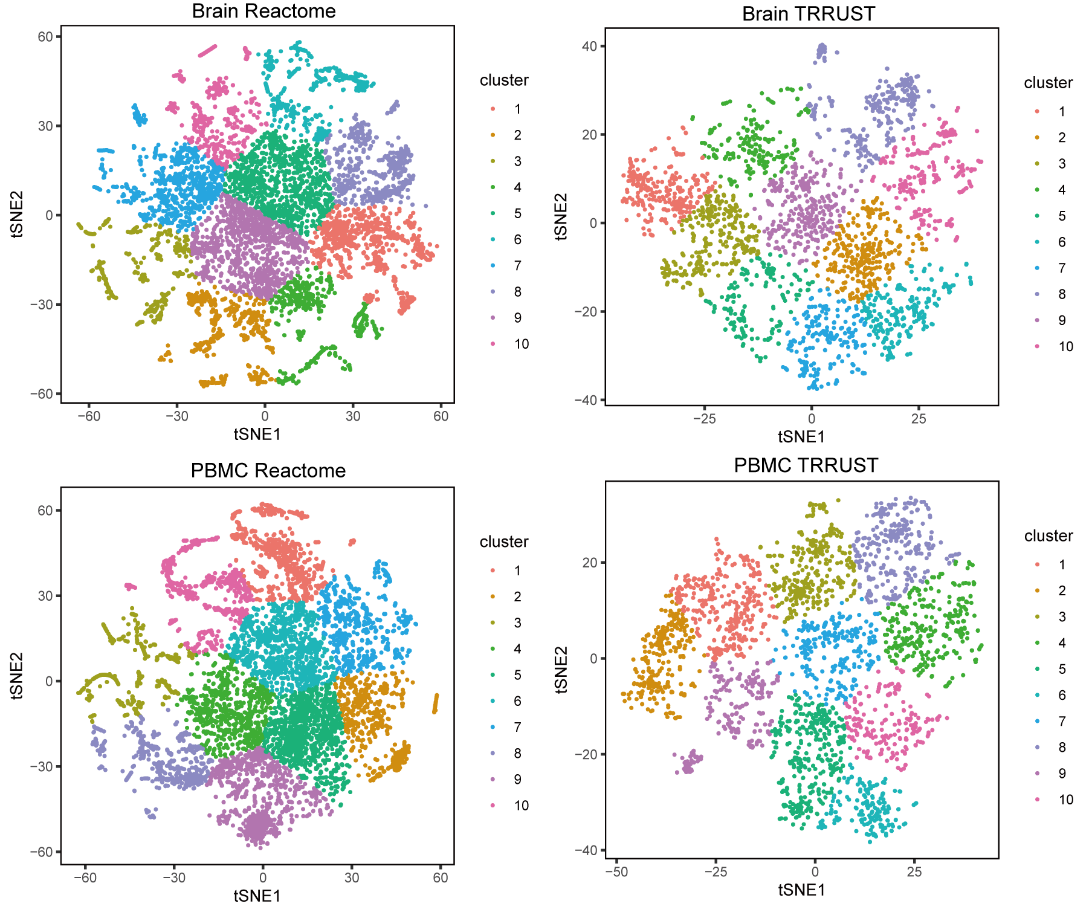
**

**Supplementary Figure 5. Clustering results based on the embeddings obtained by using the Reactome network (the left panel) and the TRRUST network (the right panel), respectively.**


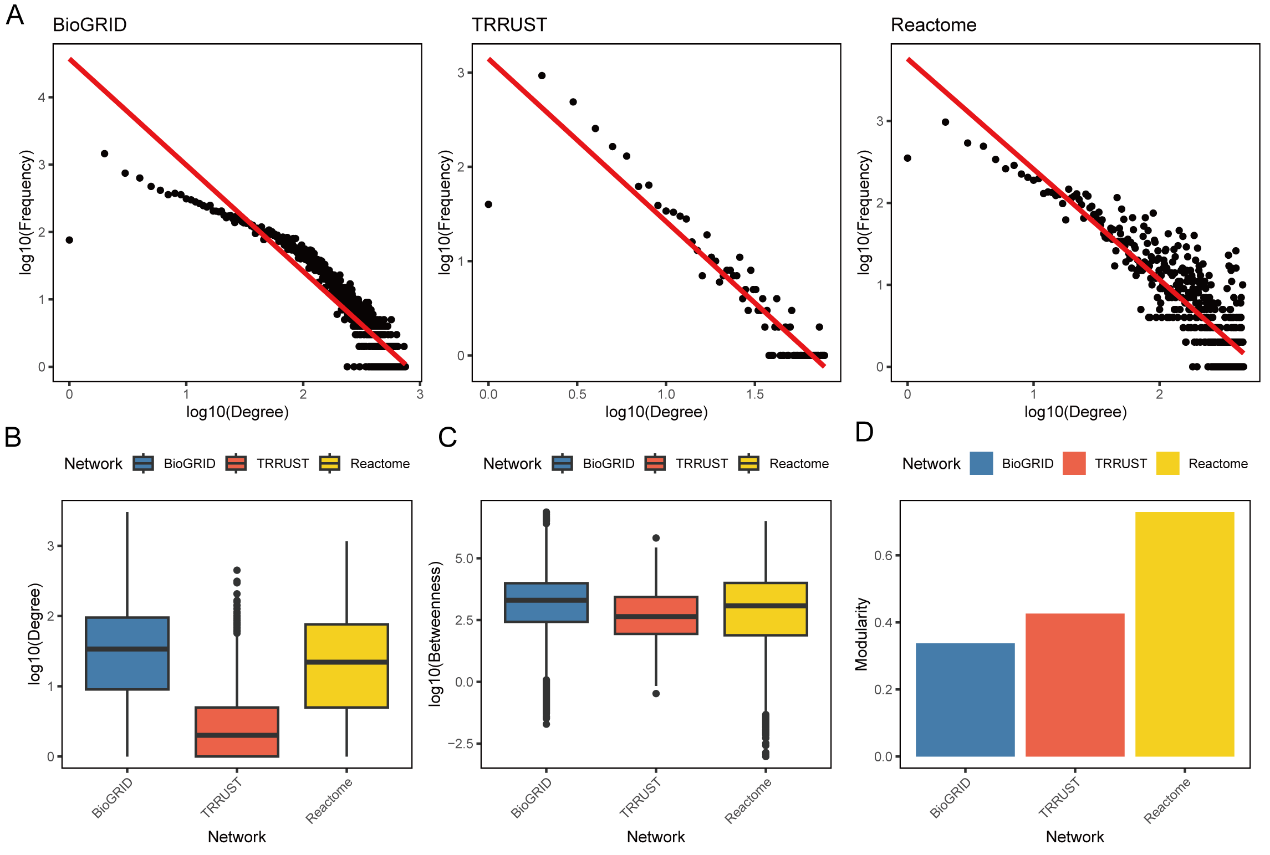


**Supplementary Figure 6.** **Characteristics of three biological networks.** We measured the network characteristics using the degree, betweenness, eigenvector centrality and modularity. **(A)** The degree of node is the number of its adjacent edges. We took the log10 of the frequency of degree and degree and fitted it with linear regression (the top three plot panel). **(B)** The betweenness of node is defined by the number of geodesics (shortest paths) going through a node or an edge. **(C)** The eigenvector centrality corresponds to the values of the first eigenvector of the graph adjacency matrix. In general, nodes with high eigenvector centralities are those which are connected to many other nodes which are, in turn, connected to many others (and so on). **(D)** We used Louvain algorithm to find community structure, and calculated the modularity of a network to measures how good the division is, or how separated are the different node types from each other.


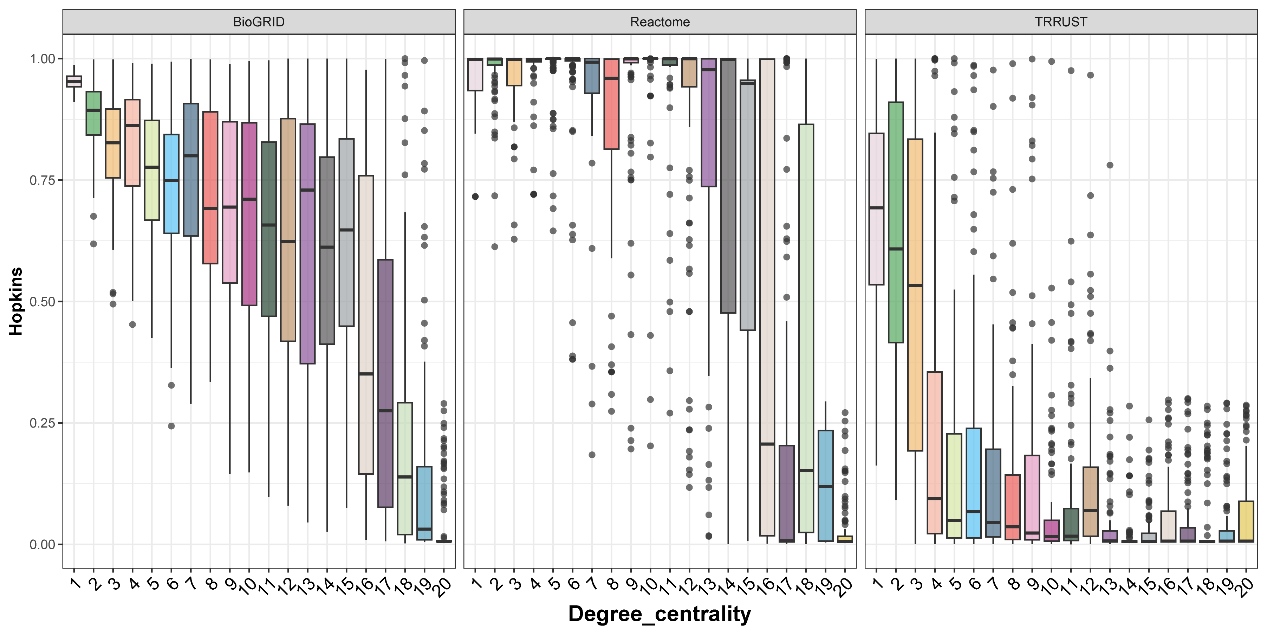


**Supplementary Figure 7. Evaluate the clustering of genes grouped by varying degree centrality.** The X axis represents 20 groups, each of which is 100 gene clusters consisting of 100 hub genes and their neighboring genes. The larger the value, the lower the degree centrality. The Y axis is the Hopkins statistic calculated for each cluster, which is used to evaluate the clustering of data in the space. Values closer to 0 indicate a tendency to cluster, while values near 1 suggest the data are more dispersed.


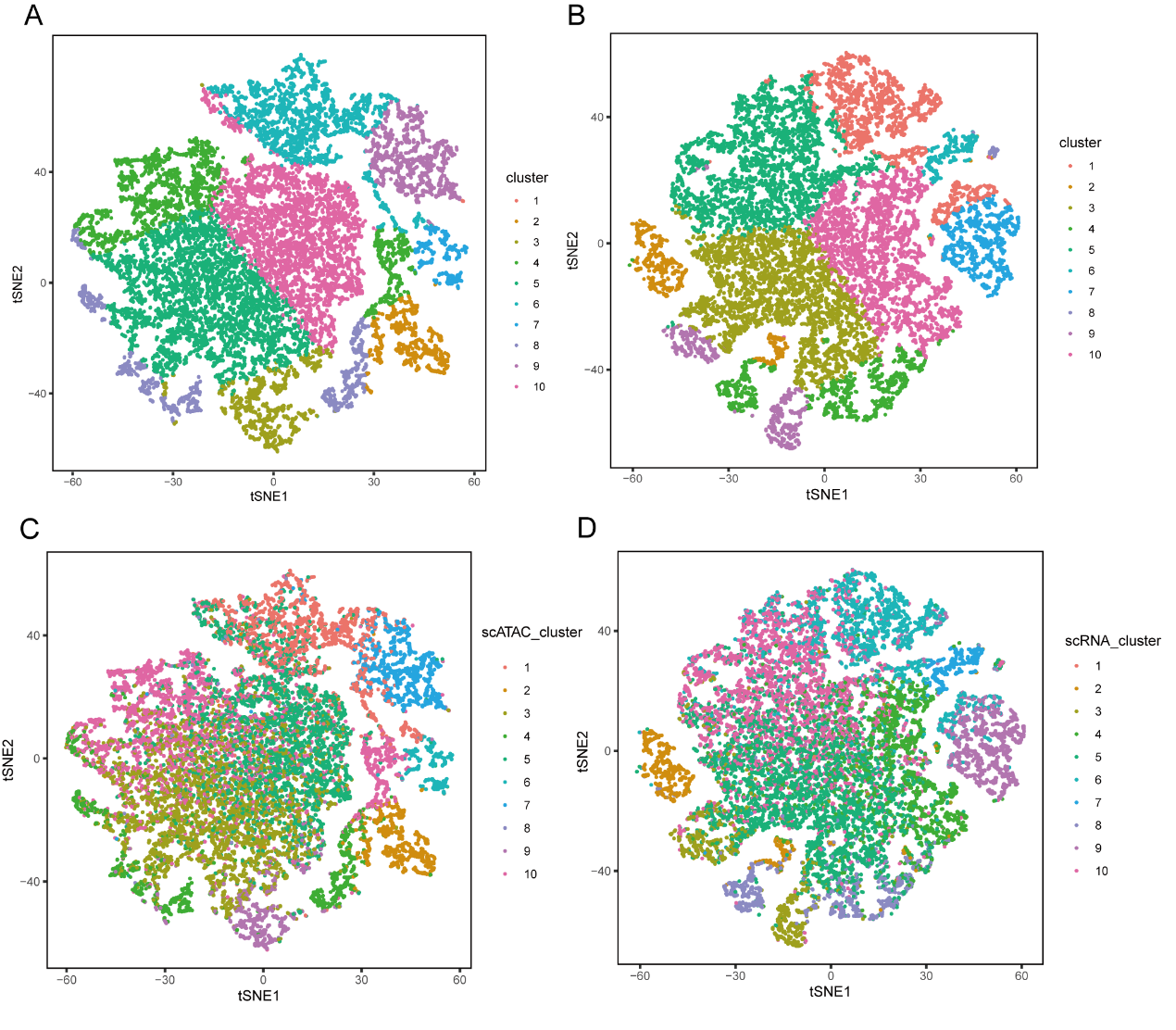


**Supplementary Figure 8. Gene clusters cross-validated using scRNA-seq and scATAC-seq data from the same brain samples.** **A.** The major gene clusters calculated by k-means clustering were labeled in the 2-dimensional space of scRNA-seq. **B.** The major gene clusters calculated by k-means clustering were labeled in the 2-dimensional space of scATAC-seq. **C.** scATAC-seq gene clusters were annotated in the 2-dimensional space of scRNA-seq. **D.** scRNA-seq gene clusters were annotated in the 2-dimensional space of scATAC-seq.


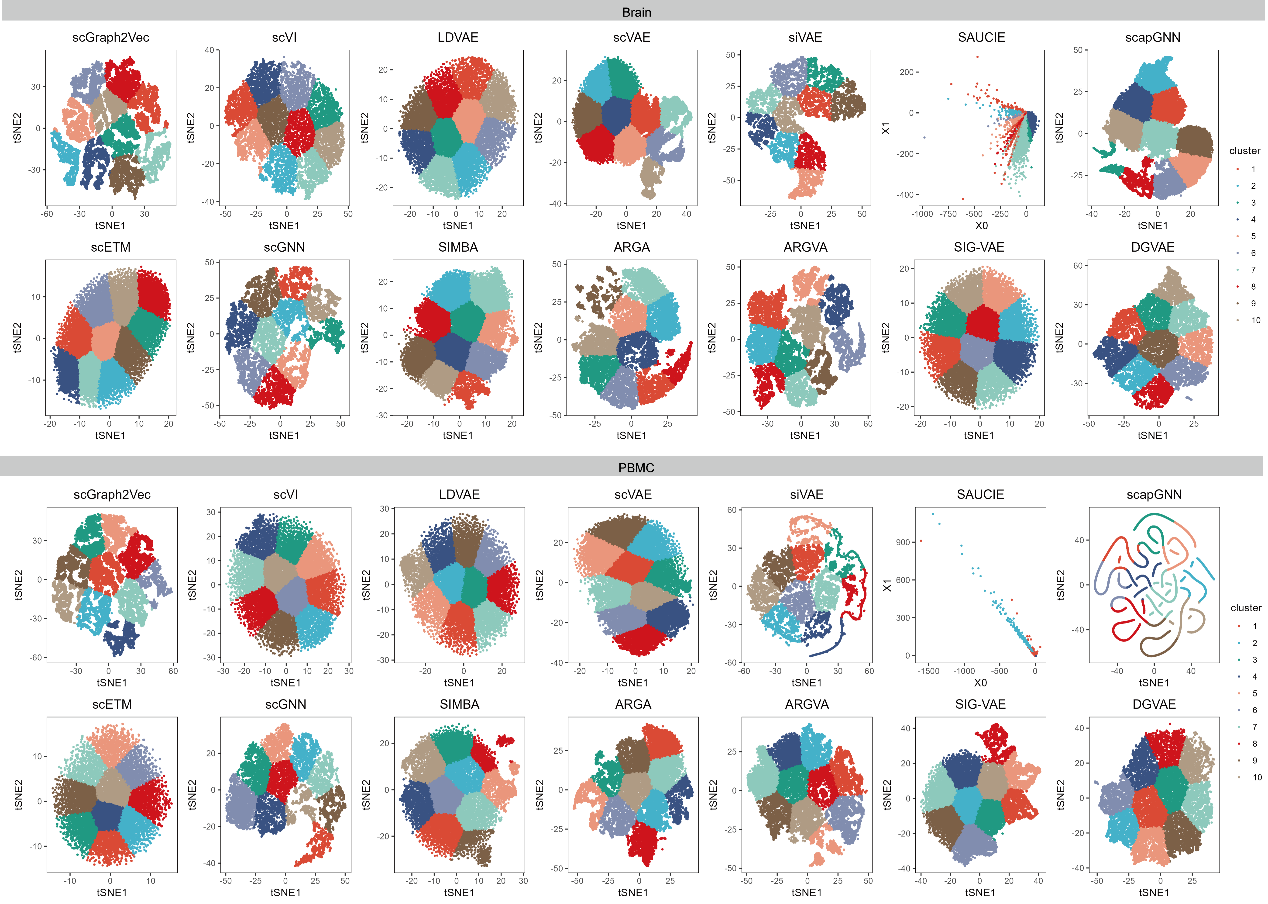


**Supplementary Figure 9.** **Comparison of gene embedding among methods on the same brain and PBMC datasets**. Except for SAUCIE, which uses the original two-dimensional features and clustering results, all other methods cluster genes through hierarchical clustering method and use t-SNE for visualization.


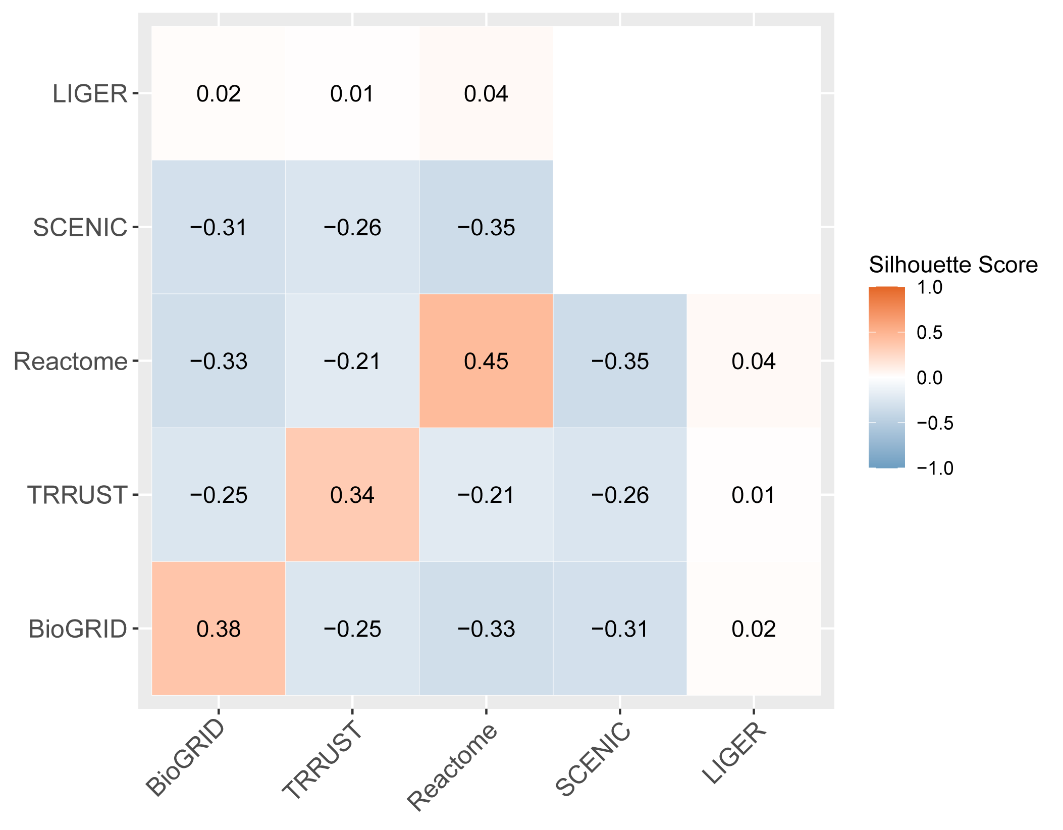


**Supplementary Figure 10.** **Clustering measurement of SCENIC and LIGER gene clusters in scGraph2Vec two-dimensional embedding space.** scGraph2Vec embeddings were generated from BioGRID network, TRRUST network and Reactome network.


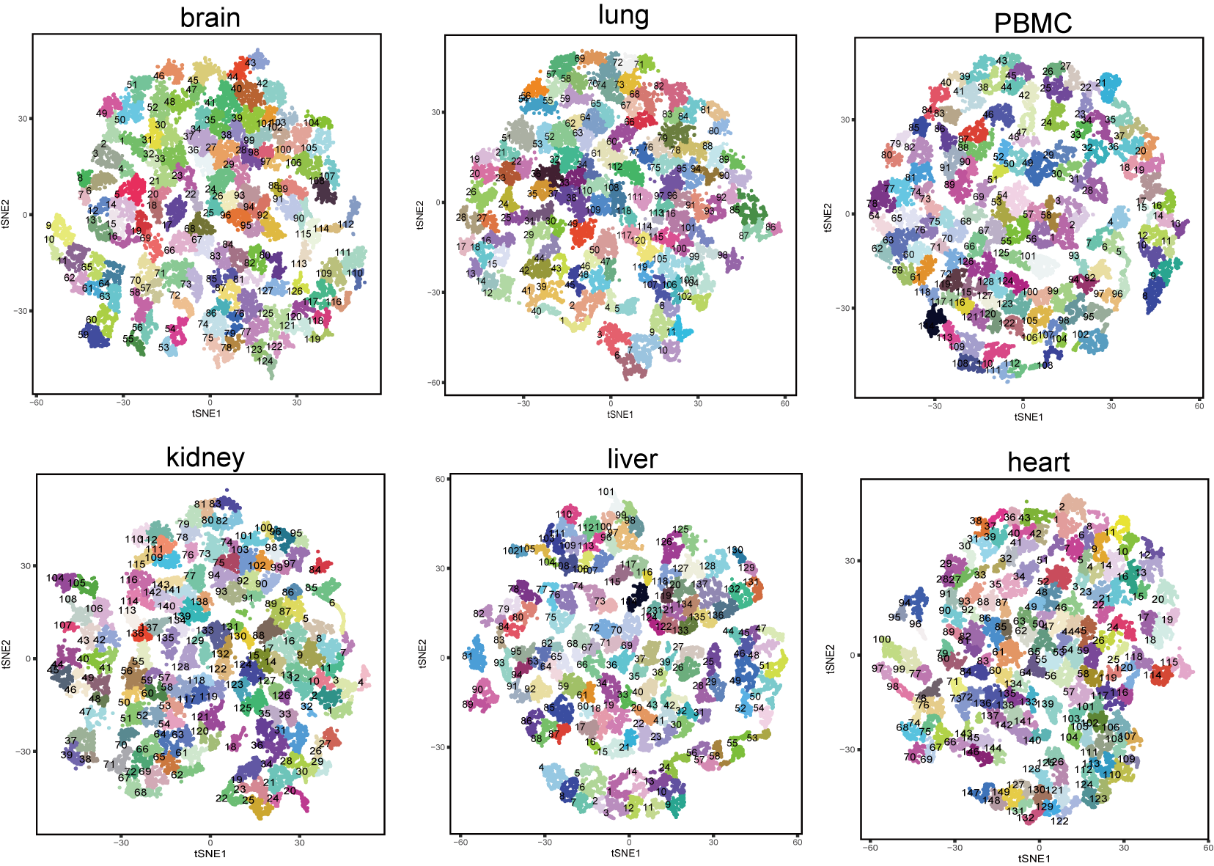


**Supplementary Figure 11. Gene clusters in 6 human tissues.**

**
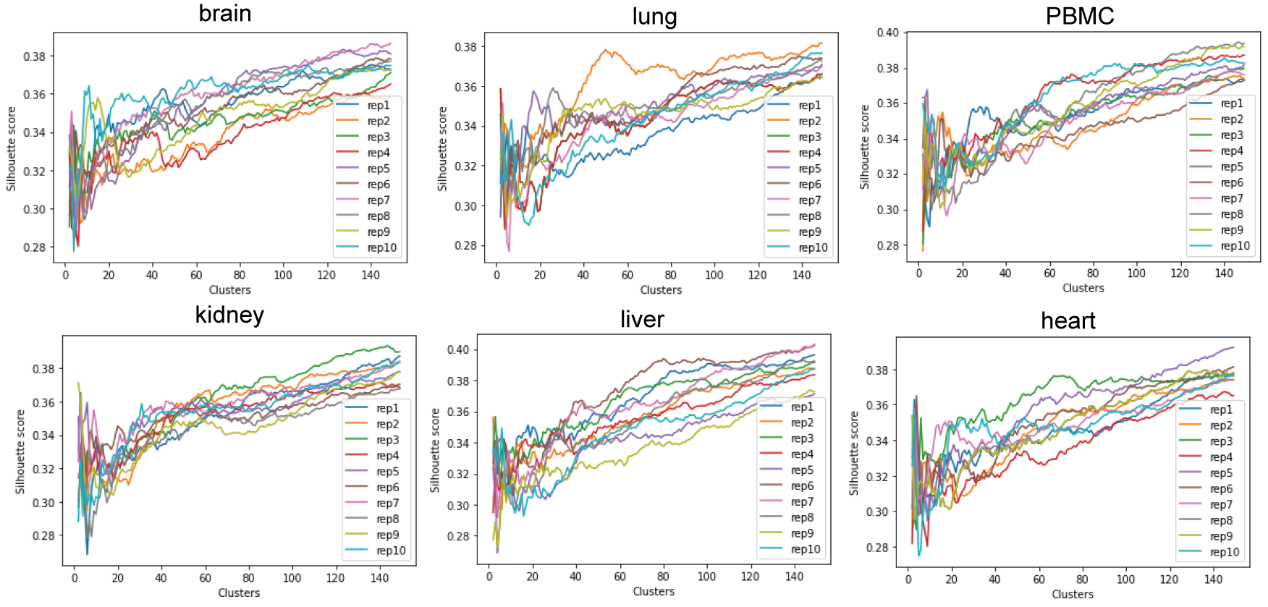
**

**Supplementary Figure 12. The optimal clustering was selected by silhouette coefficient in 10 replications.**

**
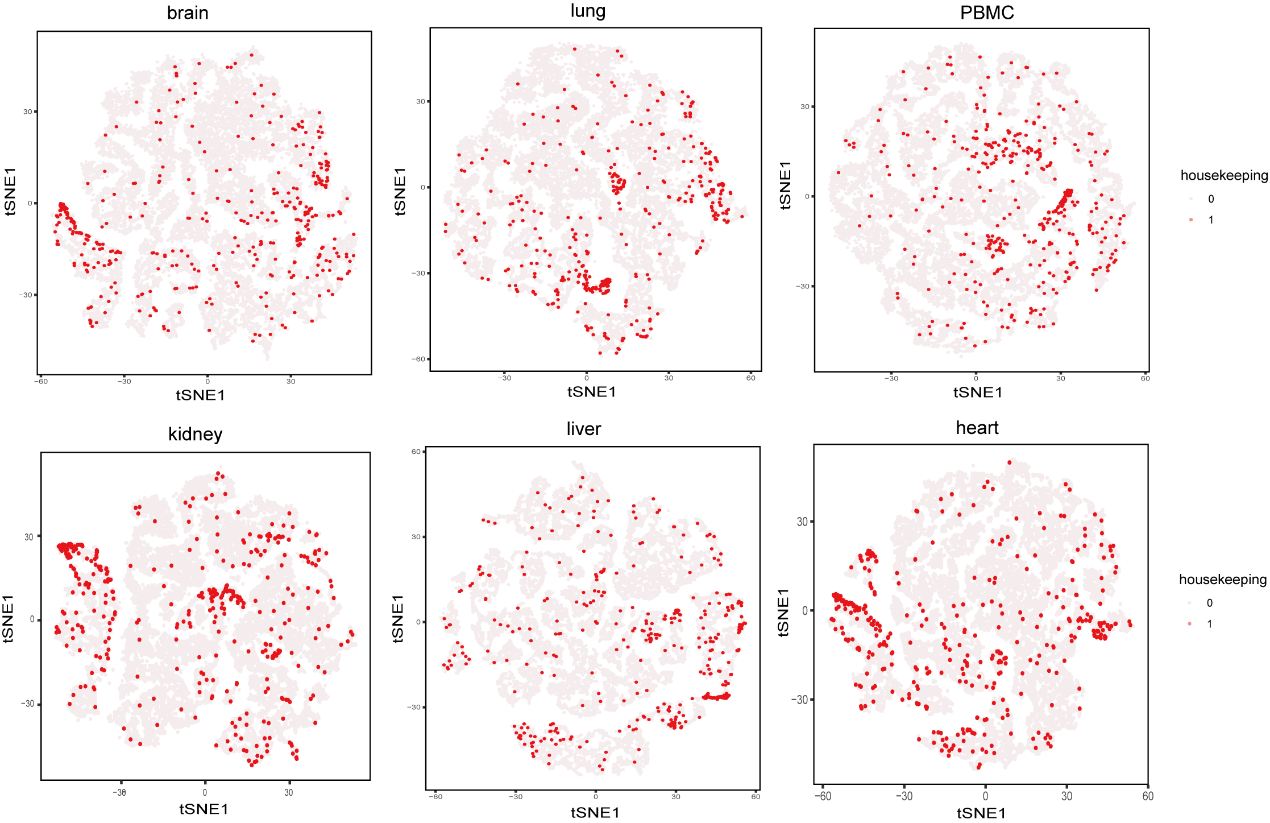
**

**Supplementary Figure 13. The distribution of housekeeping genes in latent features from 6 human tissues.**

**
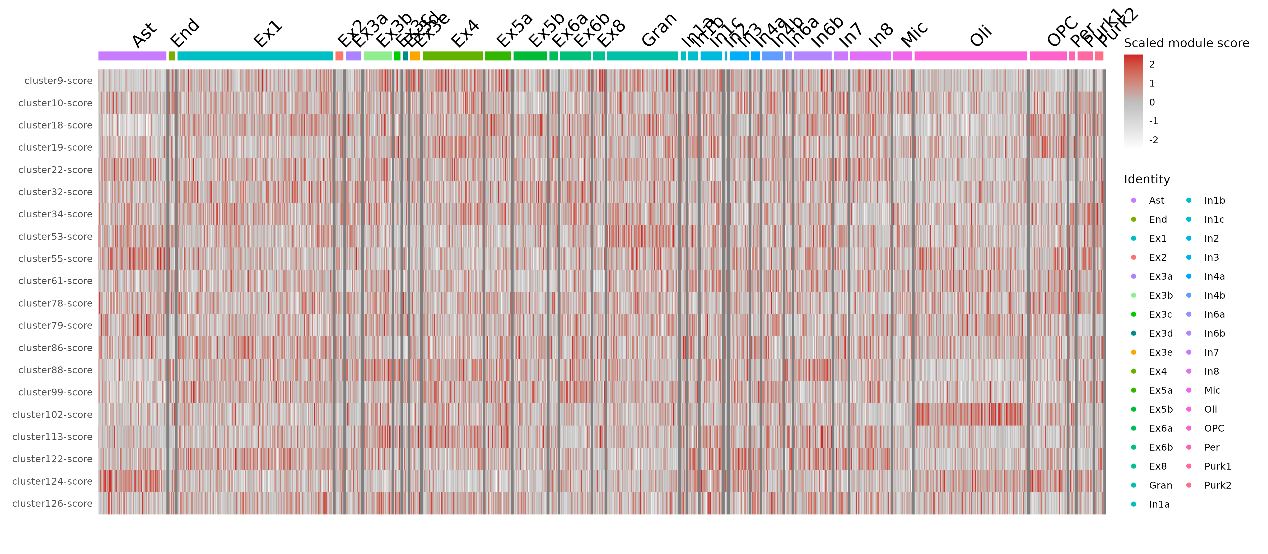
**

**Supplementary Figure 14.** **Scaled module scores in cells for the top 20 highly variable clusters.**

**
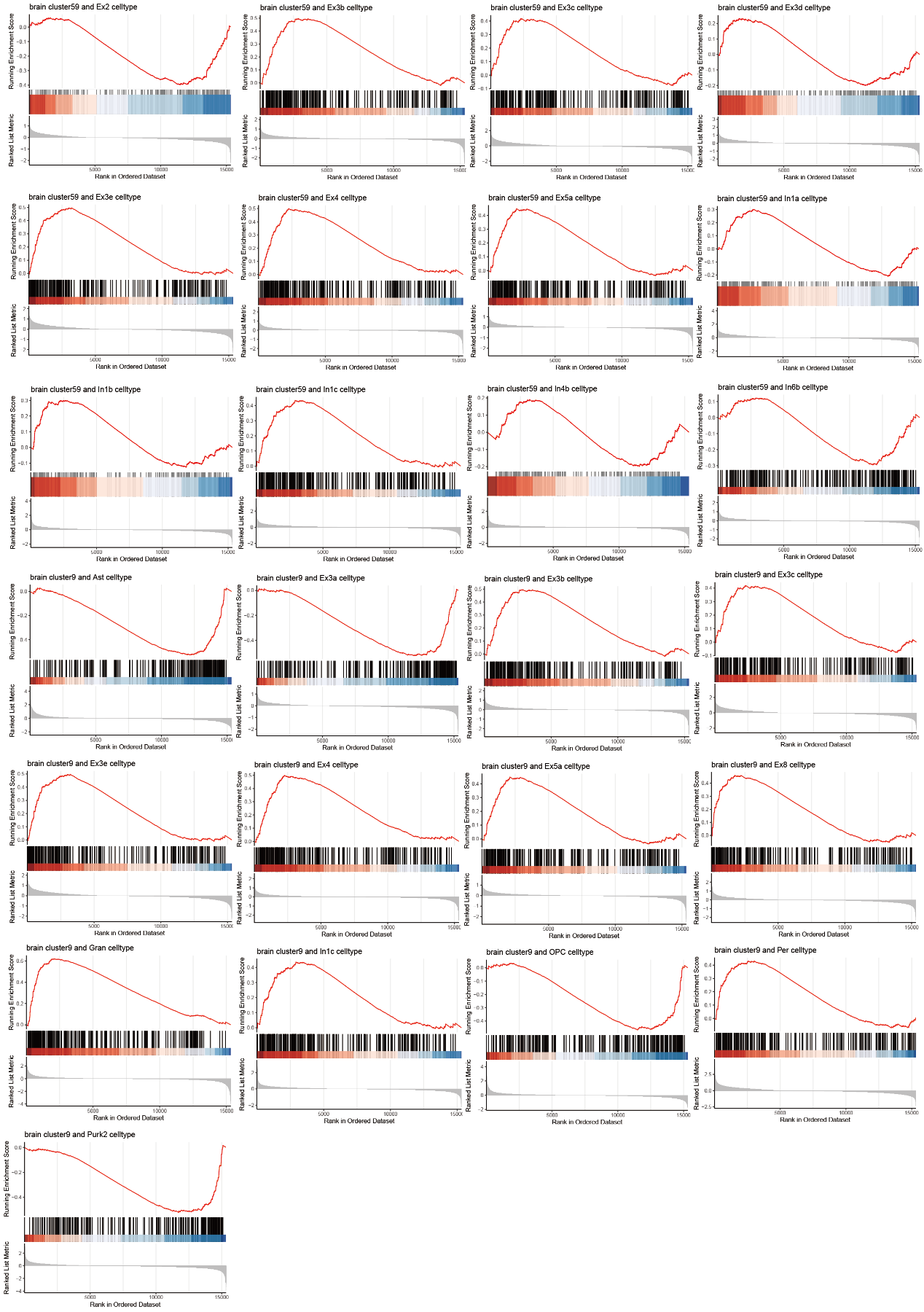
**

**Supplementary Figure 15. GSEA analysis between the gene clusters (cluster 9 and cluster 59) and cell types in the brain dataset.**


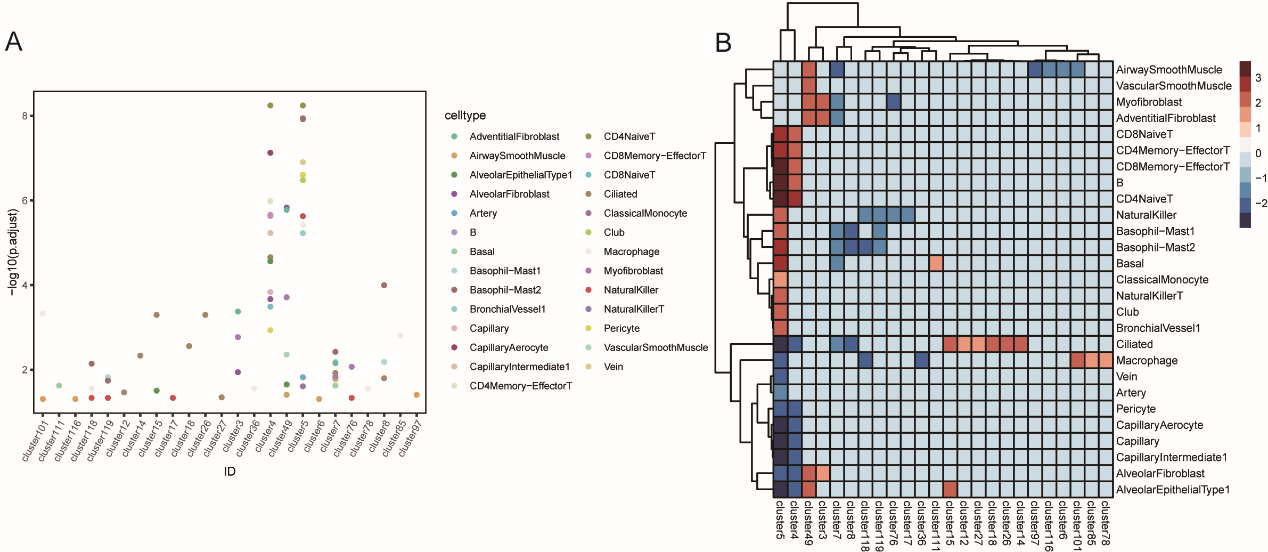


**Supplementary Figure 16. GSEA for lung clusters.** **A.** A scatter plot showing results with BH-adjusted p-value < 0.05. The X-axis shows gene clusters enriched in at least one cell type DEGs. The Y-axis shows -log10(BH-adjusted p-value). The color indicates different cell types. **B.** A heatmap to show NES in GSEA results.

**
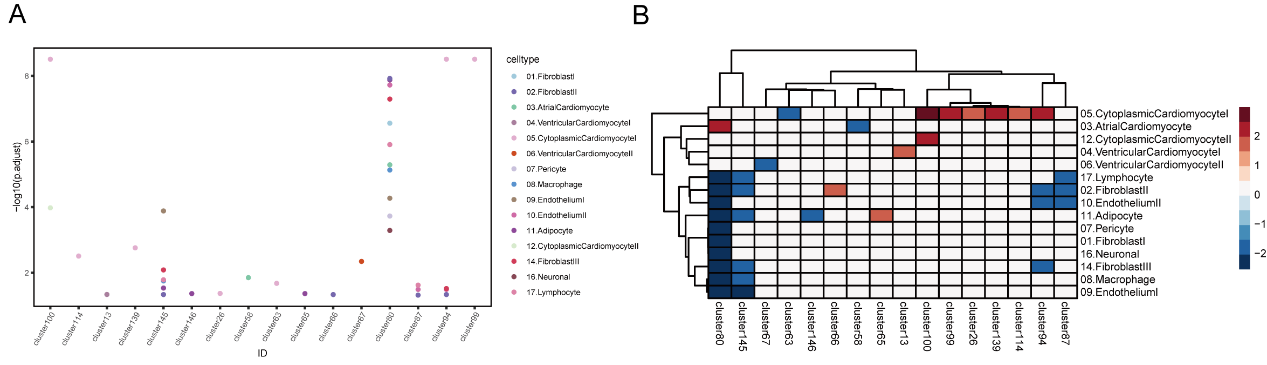
**

**Supplementary Figure 17. GSEA for heart clusters. A.** A scatter plot showing results with BH-adjusted p-value < 0.05. The X-axis shows gene clusters enriched in at least one cell type DEGs. The Y-axis shows -log10(BH-adjusted p-value). The color indicates different cell types. **B.** A heatmap to show NES in GSEA results.

**
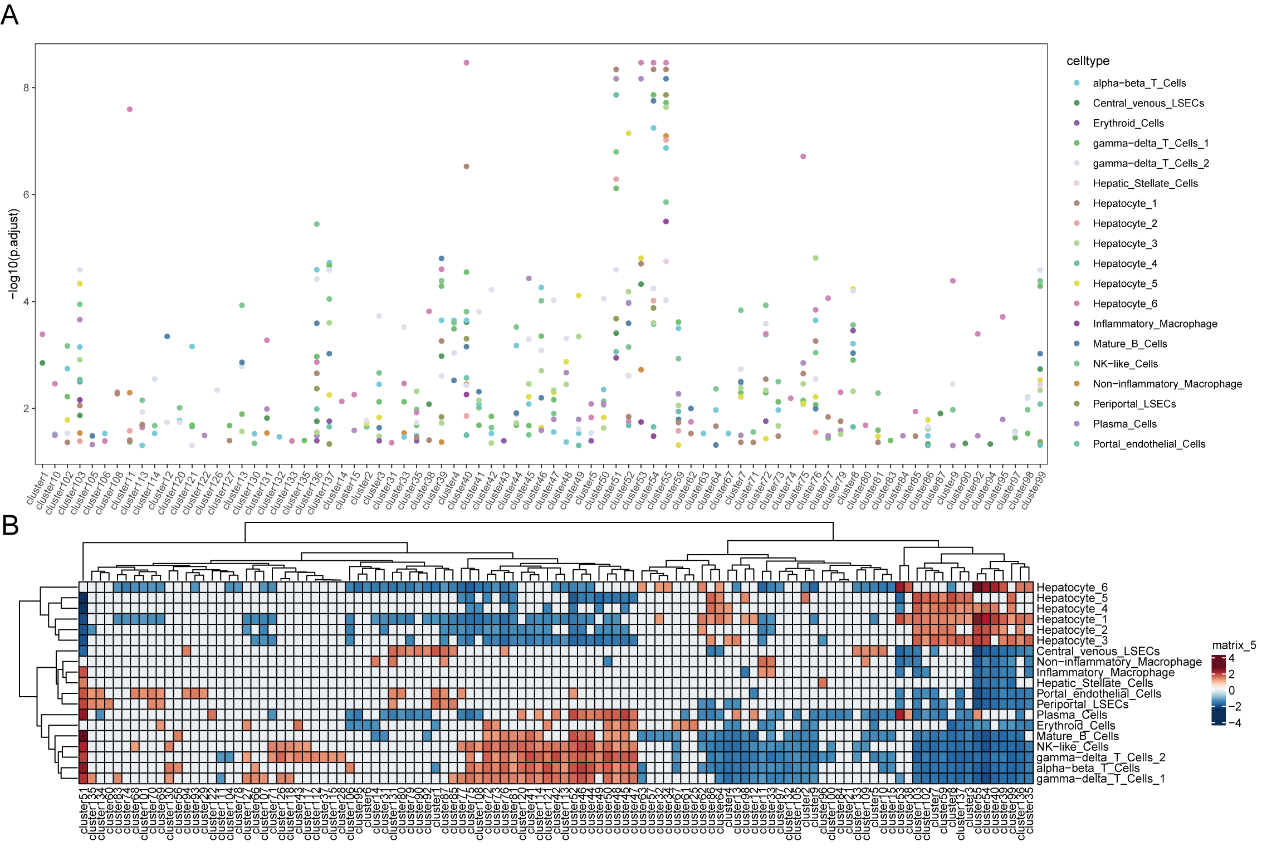
**

**Supplementary Figure 18. GSEA for liver clusters. A.** A scatter plot showing results with BH-adjusted p-value < 0.05. The X-axis shows gene clusters enriched in at least one cell type DEGs. The Y-axis shows -log10(BH-adjusted p-value). The color indicates different cell types. **B.** A heatmap to show NES in GSEA results.


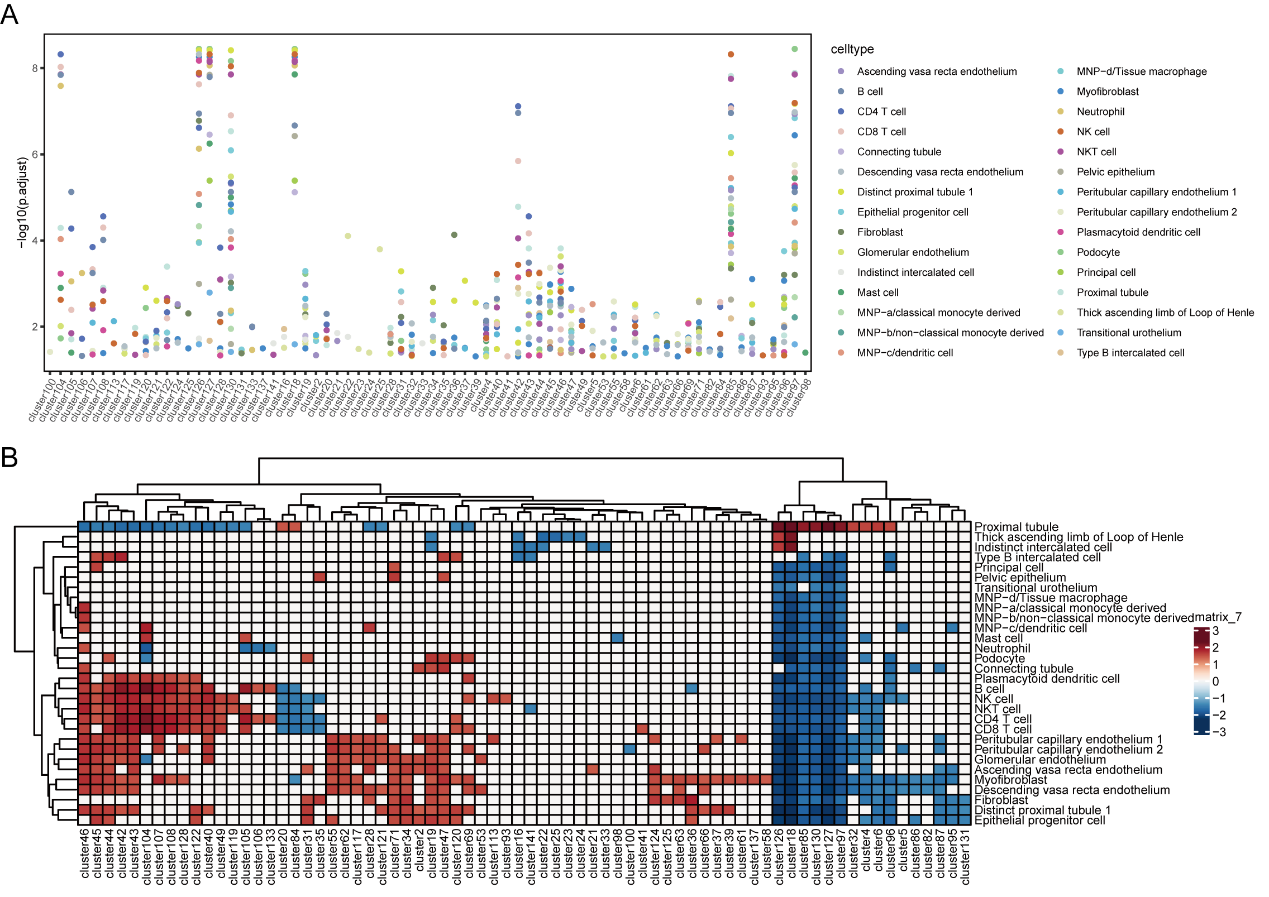


**Supplementary Figure 19. GSEA for kidney clusters. A.** A scatter plot showing results with BH-adjusted p-value < 0.05. The X-axis shows gene clusters enriched in at least one cell type DEGs. The Y-axis shows -log10(BH-adjusted p-value). The color indicates different cell types. **B.** A heatmap to show NES in GSEA results.

**
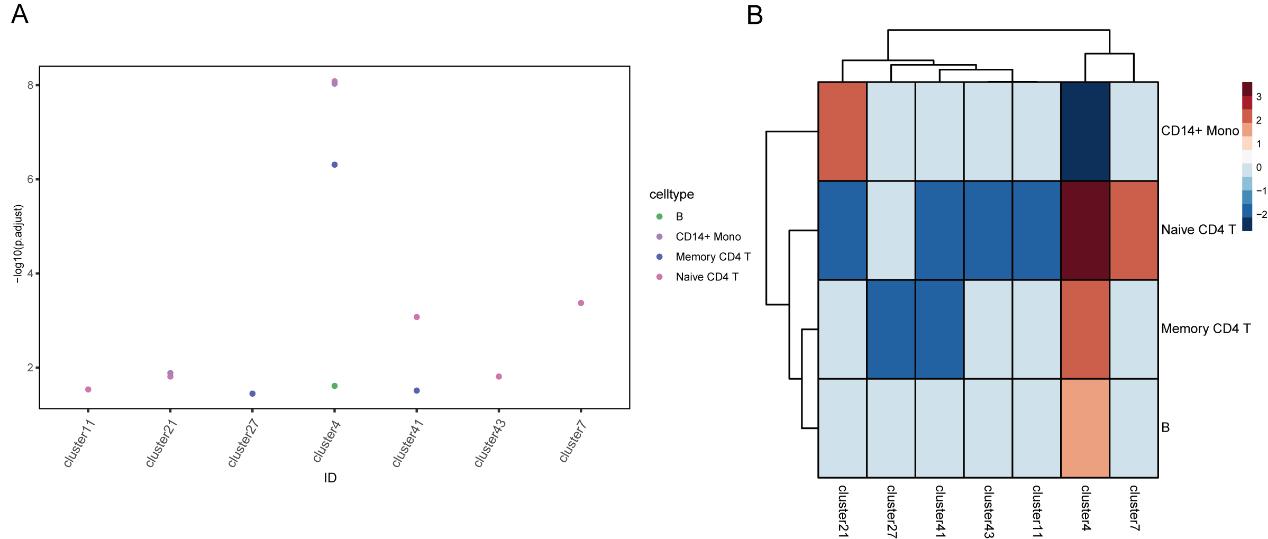
**

**Supplementary Figure 20. GSEA for PBMC clusters. A.** A scatter plot showing results with BH-adjusted p-value < 0.05. The X-axis shows gene clusters enriched in at least one cell type DEGs. The Y-axis shows -log10(BH-adjusted p-value). The color indicates different cell types. **B.** A heatmap to show NES in GSEA results.

**
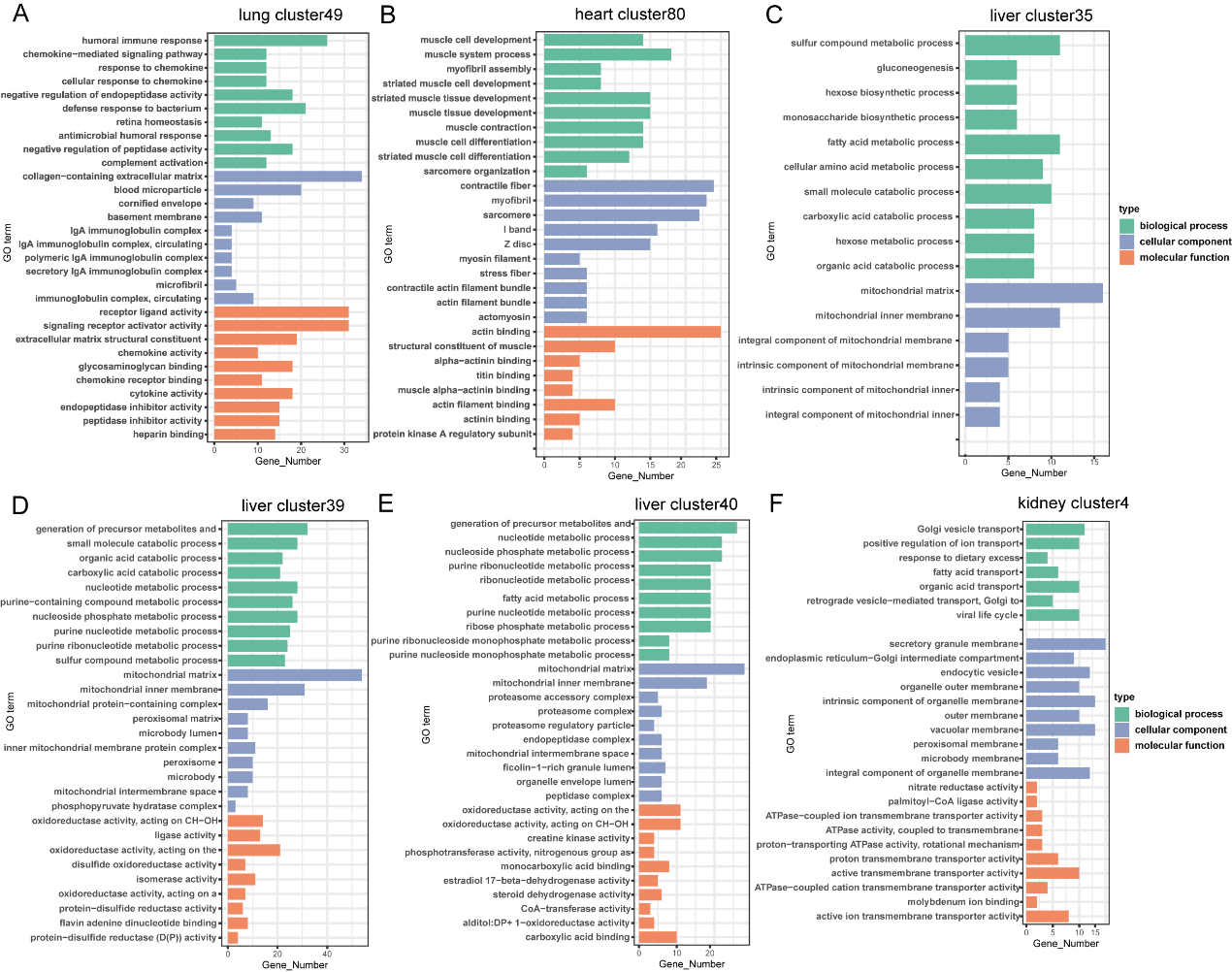
**

**Supplementary Figure 21. GO enrichment analysis for cluster genes.**

**
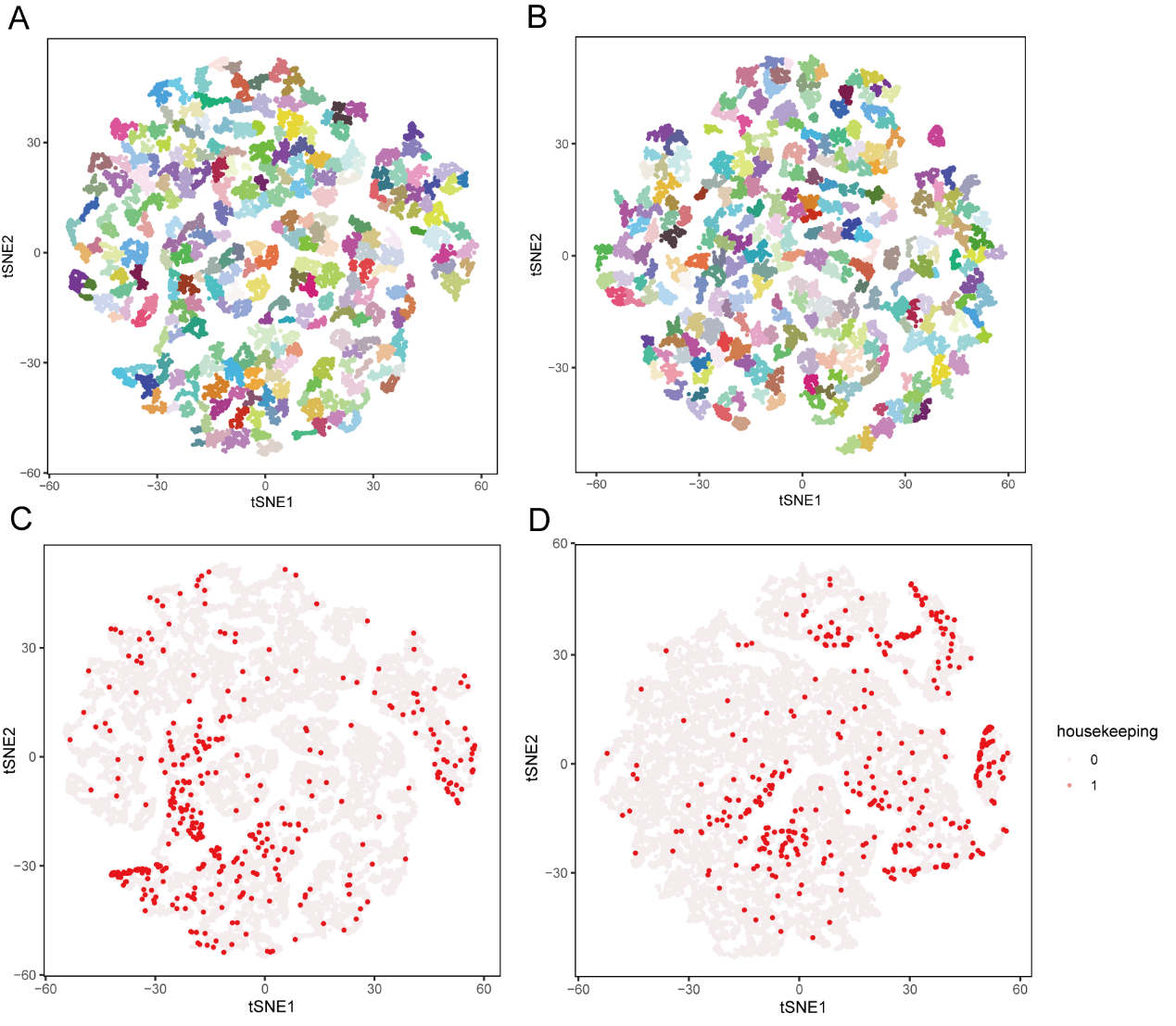
**

**Supplementary Figure 22.** Gene clusters in the tumor tissue (A) and the normal tissue (B). Housekeeping genes in latent features from the tumor tissue (C) and the normal tissue (D).


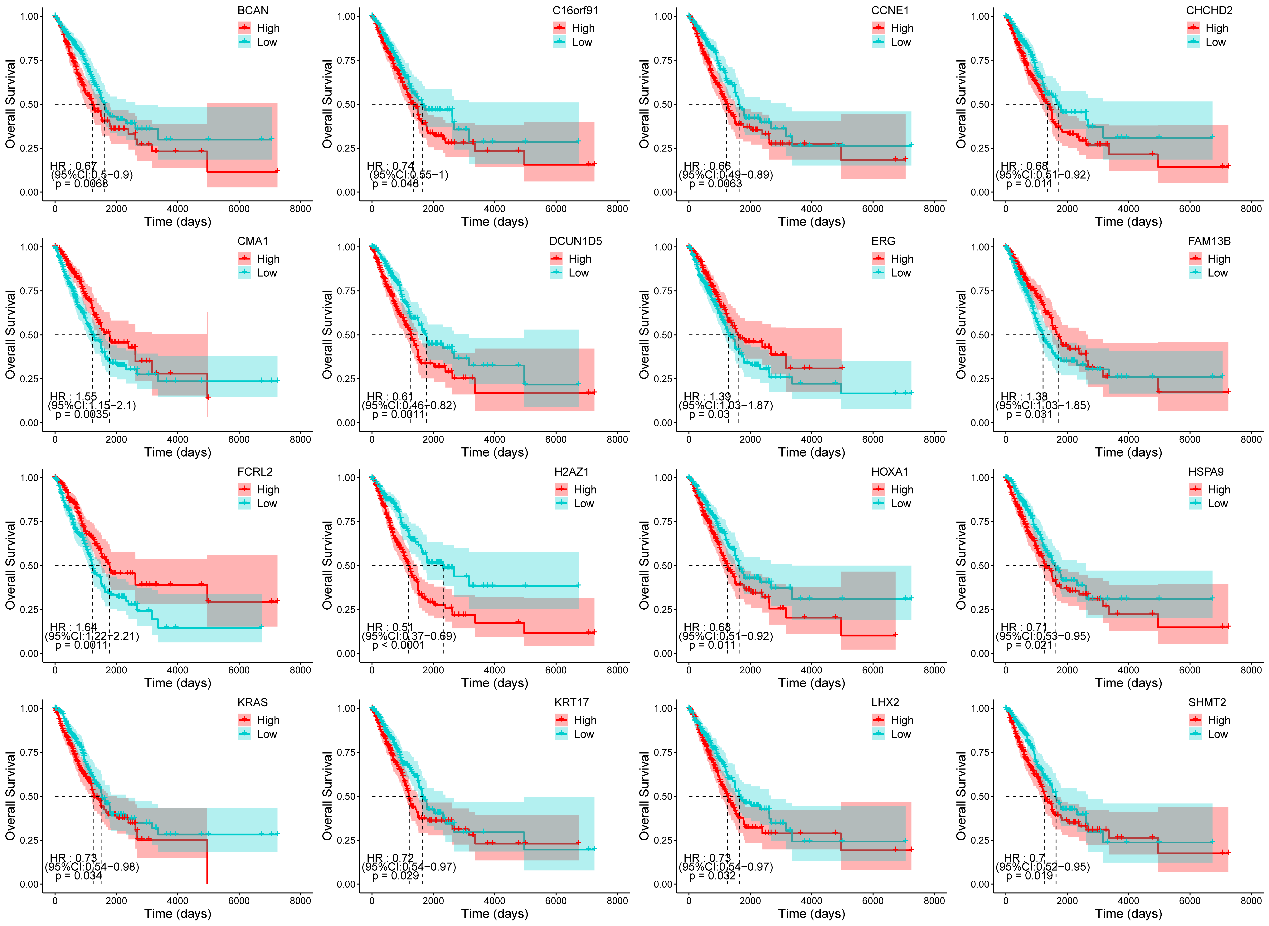


**Supplementary Figure 23. Kaplan–Meier survival analysis of 16 out of 18 overlap candidate genes.**

**
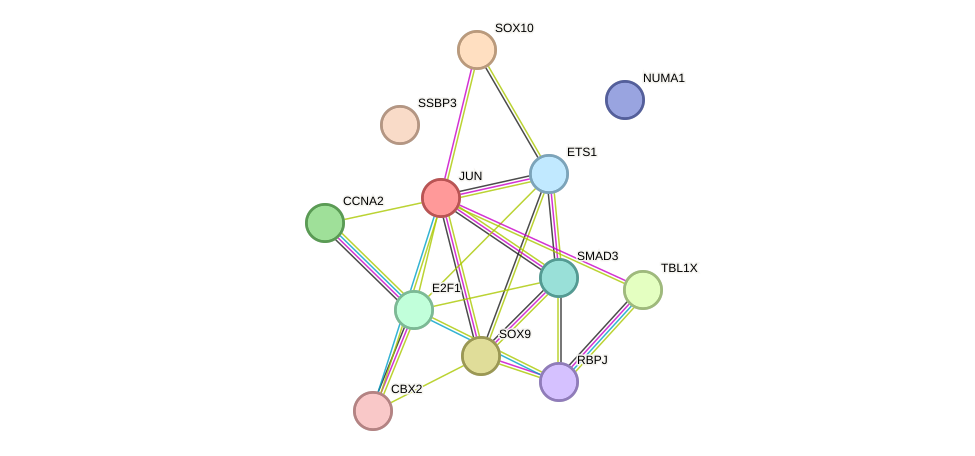
**

**Supplementary Figure 24. STRING analysis for the 12 validated *SOX10* neighbor genes.**
