# Supplementary material for: scGraph2Vec: a deep generative model for gene embedding augmented by graph neural network and single-cell omics data
Source: Gigascience. 2024 Dec 20;13:giae108. doi: 10.1093/gigascience/giae108 (PMC11659981; doi:10.1093/gigascience/giae108)
Supplement: giae108_Response_to_Reviewer_Comments_Original_Submission [file giae108_response_to_reviewer_comments_original_submission.pdf]

Author's Response To Reviewer Comments

Response to Referee #1:
The main contribution of this study lies in the proposal of a new model, scGraph2Vec, which leverages the powerful representational capabilities of Graph Neural Networks (GNNs) to capture the intricate interaction between genes, while incorporating omics information at the single-cell level. This approach facilitates a deeper understanding for researchers of gene regulation, cell type differentiation, and the molecular mechanisms underlying diseases. However, the content of this manuscript requires further enhancement, particularly in elucidating the novel biological insights or advantages offered by the gene embeddings generated by this model compared to those gene modules derived from other state-of-the-art algorithms.

Response: We thank the reviewer for the positive evaluation and suggestions of our manuscript. Following your suggestion, we compared the gene embeddings by scGraph2Vec with those derived by other state-of-the-art algorithms. In total, we compared 13 gene embedding methods published in recent years and included two additional representative gene clustering methods, SCENIC and LIGER. Additionally, we incorporated a comparison of three types of gene networks to highlight the unique advantage of scGraph2Vec in integrating gene interaction networks with single-cell omics data. We assessed the ability of each method to generate gene embeddings and gene clusters. To evaluate the biological advantages of each method, we used the MsigDB hallmark gene set as a ground truth.
The results showed that scGraph2Vec effectively combined diverse gene interaction networks with single-cell omics to generate highly informative gene embeddings. These embeddings capture both network-specific interaction features and biological context-specific expression patterns, allowing for clearer separation of gene clusters and more precise identification of co-functional gene programs.

In addition to the shortcomings in novelty and biological significance within this manuscript, here are some revision suggestions:
#1. In Fig. 2, the authors show that the gene embeddings acquired through the scGraph2Vec model achieve higher silhouette scores than those resulting from alternative algorithms. Nonetheless, they fail to expound upon the biological relevance of these gene embeddings, nor do they illustrate that the gene embeddings procured via scGraph2Vec encompass greater or more precise biological insights relative to the gene modules yielded by competing algorithms.

Response: We thank the reviewer for this valuable suggestion. During revision, we compared scGraph2Vec with 13 gene embedding methods and two representative gene clustering methods, SCENIC and LIGER. We evaluated the gene clusters using the silhouette coefficient and Davies-Bouldin Index and assessed the similarity of gene clusters using the Jaccard index. To ensure a fair evaluation of biological relevance, we introduced the MsigDB hallmark gene sets as the ground truth to measure the functions of gene clusters.
The results showed that scGraph2Vec outperformed nearly all other methods in separating gene clusters, except for siVAE, which performed better on the PBMC dataset (Fig. 2B-C and Supplementary Fig. 9). We observed significant differences in the gene clusters generated by these methods (Fig. 2D). However, when compared to the MsigDB hallmark gene sets, scGraph2Vec clustered the highest number of functionally similar genes among all competing algorithms (Fig. 2E).
Additionally, we explored the biological relevance of gene embeddings from several perspectives, including network topology, clustering of annotated gene sets, cell type specificity, and tissue specificity.
(1) Network topology. The internal closeness centrality of genes in each cluster was significantly higher than their external closeness centrality (Fig. 3A).
(2) The clustering of annotated genes. Genes with similar functions, e.g., the housekeeping genes and the hallmark genes, tend to cluster together (Fig. 3B, C and Supplementary Fig. 13).
(3) Cell type specificity. When we applied scGraph2Vec to data from six representative human tissues, gene embeddings helped us identify clusters with cell type-specific expression (Fig. 4A, B and Supplementary Fig. S15). For instance, cluster 59 was enriched in multiple excitatory and inhibitory neurons and was particularly associated with down-regulated differentially expressed genes (DEGs) in these cell types (Fig. 4A and Supplementary Fig. S15).
(4) Tissue specificity. Embeddings also captured tissue-specific functional clusters. For example, our comparison of SMARCE1's neighboring genes in the brain and lung revealed distinct functions: in brain

tissue, 14 neighboring genes were enriched in neuronal fate commitment and motor neuron differentiation (Fig. 4F), while in lung tissue, 20 neighboring genes were enriched in chromatin organization and histone H3-K9-related functions (Fig. 4G).
(5) Four applications to identify critical genes in different biological contexts. The four application cases, i.e., COVID-19, Alzheimer's disease, lung adenocarcinoma, and melanoma, illustrated that scGraph2Vec can be widely used in various scenarios and identify candidate genes critical to the biological conditions, be it disease-associated genes, cancer driver genes, or regulatory genes for cell states.
In summary, scGraph2Vec integrates various gene networks and single-cell omics data from diverse biological backgrounds to generate highly informative gene embeddings. These embeddings capture both the topological characteristics of biological networks and biologically significant gene clusters, uncovering context-specific gene regulatory programs.

#2: In Fig. 2B, the authors indicated that "housekeeping genes tended to cluster together and were enriched in a few gene modules". Does this suggest that these gene modules are related to cell maintenance functions or states?

Response: We thank the reviewer for pointing this out. We extracted gene clusters containing more than 20 housekeeping genes in the six normal human tissues and conducted functional analysis (See Table 1). Interestingly, in all tissues, the clusters with the largest number of housekeeping genes were all enriched with cytoplasmic translation and ribosome biogenesis, indicating that these clusters were involved in the basic functions of cell maintenance.

#3: The authors used module score to represent the overall expression pattern of module genes, is there mathematical proof for this? Is there biological evidence to support this claim? Is there a ground truth available to validate its reliability?

Response: We thank the reviewer for these valuable suggestions. In the first submission, we used the module eigengene to calculate the module score. This procedure followed the Weighted correlation network analysis (WGCNA) algorithm and had been well-validated in the original WGCNA study as well as in many studies citing WGCNA [1]. However, during revision, we found that the "AddModuleScore" function in the Seurat software was more frequently used in scRNA-seq data analyses, especially to summarize the expression patterns of gene sets in different cell types [2]. Thus, in the revised manuscript, we updated our analysis strategy and used the "AddModuleScore" to calculate module scores.

#4: In Fig. 4, the authors performed a GO-term analysis of the gene modules obtained from the scGraph2Vec model, and utilized the functions of these modules to investigate the functionalities of various cell types. What are the advantages of this approach compared to directly using differentially expressed genes (DEGs), or gene modules obtained from other algorithms?

Response: We thank the reviewer for this valuable suggestion. scGraph2Vec aims to identify gene clusters with coordinated expression patterns across cells. It does not use predefined cell type information during training. The relative distances among genes in the two-dimensional embedding space reflect their biological relationships, capturing not only cell type-specific expression patterns but also the topological characteristics of the gene interaction network. In contrast, directly using DEGs focuses on comparisons between cell types but does not consider the coordinated gene expression across multiple cells. In case studies such as COVID-19, Alzheimer's disease (AD), lung adenocarcinoma (LUAD), and melanoma, the density-based search algorithm allowed us to identify genes associated with candidate disease genes within the embedding space. This highlights the unique strengths of gene embeddings in uncovering additional disease-associated genes and revealing regulatory genes specific to different biological contexts.

#5: In studies on COVID-19 and Alzheimer's disease, the authors utilized disease-related variants identified through GWAS analysis, combined with information from gene modules, to screen for genes associated with these diseases. How did the authors determine that these variants in the genes would lead to changes in the RNA expression levels of these genes? Was it because these variant sites were located in promoter or enhancer regions of these genes? Is there any experimental evidence for this?

Response: We thank the reviewer for pointing this out. We used the MAGMA software to calculate gene-

based p-values and defined seed genes by considering the study-wide significant level based on the Bonferroni correction. MAGMA maps SNPs to genes based on genomic locations, assigning an SNP to a gene if the SNP's location falls inside the gene region. In addition, SNPs near a gene but outside its transcription region can be mapped to that gene using a pre-defined window. We used the default settings by MAGMA, i.e., "--annotate window=5,1.5", where SNPs located 5kb upstream and 1.5kb downstream were also mapped to a gene. Then, MAGMA implemented a multiple linear principal components regression model and an F-test to compute the gene p-values. The MAGMA pipeline has been widely used in various GWAS analysis applications.

#6: Some paragraphs and sentences in this manuscript have rather stiff language expression. The authors can use English proofreading service to prevent grammatical and semantic errors in expression.

Response: Thank you for your valuable feedback. During revision, we have carefully revised the text to improve clarity and readability. Additionally, we have considered professional English proofreading services to further enhance the manuscript and ensure that it meets the highest linguistic standards. We appreciate your suggestion and will make every effort to improve the overall quality of the writing.

Response to Referee #2:
The authors have proposed a computational method called 'scGraph2Vec' that takes a single-cell gene expression profile and a gene interaction network as input, and produces low-dimensional gene embedding as output. The links to the used datasets are either provided in the Supplementary Table S1 or mentioned in the main text. The source code has been made publicly available at https://github.com/LPH-BIG/scGraph2Vec. I highly appreciate the effort the authors have made to improve the reproducibility of their work. It would be great if the authors could address the following comments to strengthen the publication.

Response: We thank the reviewer for the positive comments on our manuscript. Each of these comments has been considered in the revised manuscript and is discussed below.

#1: The background section needs to be significantly improved. It has been written that scGraph2Vec "extends the VGAE framework". However, VGAE is a data-agnostic framework; scGraph2Vec rather utilises the VGAE framework for analysing gene expression and gene interaction network data. Therefore, the authors need to incorporate a critical review of the recent method that do the same. I am listing a few references I could find for that purpose:
https://academic.oup.com/nar/article/51/13/6578/7184155
https://isip.piconepress.com/conferences/ieee_spmb/2022/papers/p01_07.pdf
http://dr.iiserpune.ac.in:8080/xmlui/handle/123456789/7885
https://arxiv.org/abs/1802.04407
There are more references that I could not list here. I request the authors to enhance the background section by reviewing such methods.

Response: Thank you for your insightful comments. In the revised manuscript, we have reinforced a critical review of recent VGAE-based embedding methods in the background section.

#2: In addition to the critically reviewing other VGAE-based gene embedding methods, I request the authors to include them in the comparisons based on the benchmark datasets.

Response: We thank the reviewer for this valuable suggestion. Following your suggestion, during the revision process, we compared scGraph2Vec with four VGAE-based embedding methods and nine single-cell embedding methods.
Notably, we were unable to run the two methods cited in the first and second references by the due date of this revision. The first method, GenKI, was designed to capture shifting patterns in gene regulation caused by knockout (KO) perturbations. In our attempt, GenKI took days to construct the gene networks. Even after we reduced the genes to 1,865 highly variable genes in PBMC and 5,182 in the brain, we still could not obtain results using 60 CPU cores after a month. For the second reference, we were unable to test the method as its source code was unavailable.
Thus, we focused on the other VGAE-based embedding methods, including the highly cited SIG-VAE and

DGVAE. SIG-VAE extends VGAE's flexibility by using a hierarchical variational framework and a Bernoulli-Poisson decoder, allowing for better modeling of complex graph dependencies and implicit posteriors. DGVAE, on the other hand, introduces graph cluster memberships as latent factors, demonstrating strong performance in graph generation and clustering tasks. In total, we benchmarked 13 VGAE-based embedding methods (ARGA, ARGVA, SIG-VAE, DGVAE, scVI, LDVAE, siVAE, scVAE, scGNN, scapGNN, scETM, SAUCIE and SIMBA) against scGraph2Vec.
We systematically evaluated the ability of different methods to divide clusters, the similarity of gene clusters among methods, and the overlap of each cluster with the MsigDB hallmark gene sets. The results showed that scGraph2Vec could divide different gene clusters more clearly than other methods, except siVAE on the PBMC dataset (Fig. 2B-C). We found that the gene clusters identified by different methods were quite different (Fig. D). However, when compared with the MsigDB hallmark gene sets, the clusters by scGraph2Vec tended to have higher proportions of the hallmark genes, i.e., genes with common functions, than other competing algorithms (Fig. 2E). These results indicated that the clusters by scGraph2Vec tended to be more functionally correlated.

#3: The authors have utilized the gene embedding outputs of scGraph2Vec for clustering genes. Therefore, I request the authors to compare the resultant gene clusters with that of other single-cell gene expression based gene clustering methods such as SCENIC (https://www.nature.com/articles/nmeth.4463) and LIGER (https://www.cell.com/cell/pdf/S0092-8674(19)30504-5.pdf).

Response: We thank the reviewer for bringing this point up. SCENIC and LIGER generate gene clusters but do not generate gene embeddings. Thus, we applied SCENIC and LIGER using the same brain and lung datasets as we used in scGraph2Vec and generated gene clusters. Then we compared the gene clusters identified by SCENIC and LIGER in the gene embedding space identified by scGraph2Vec. Our analysis revealed that SCENIC and LIGER gene clusters were not closely distributed in our embedding space and did not overlap significantly with our scGraph2Vec clusters (Supplementary Fig. 10). By assessing the similarity of the gene clusters (by SCENIC, LIGER, and scGraph2Vec, respectively) with the MsigDB hallmark gene set, we found that the gene clusters by scGraph2Vec showed a higher proportion of the hallmark genes (Fig. 2E). Thus, we consider that scGraph2Vec can generate gene clusters more functionally correlated.

#4: The authors have used the silhouette index to compare the gene clusters produced by different methods. I would request the authors to incorporate additional evaluation metrics such as the Davies-Bouldin index (DBI) and Jaccard index.

Response: We thank the reviewer for raising this important point. During revision, we have included the Davies-Bouldin Index and the Jaccard Index to compare the gene clusters (Fig. 2).

Response to Referee #3:
The manuscript introduces a novel deep-learning framework designed to generate informative gene embeddings. This framework, scGraph2Vec, extends the variational graph autoencoder (VGAE) model and integrates single-cell data patterns along with gene interaction networks. The model's effectiveness is demonstrated through its ability to produce biologically interpretable gene embeddings that reveal functional modules of genes, which can be applied to identify disease-associated genes and understand tissue-specific cellular processes.

Response: We thank the reviewer for the positive comments on our manuscript.

However, the authors still have the following problems to solve:
#1. The manuscript compares scGraph2Vec with several existing gene embedding methods. It would be beneficial to include more recent methods, especially those with novel algorithmic approaches to network analysis.

Response: We thank the reviewer for this valuable suggestion. During the revision process, we systematically reviewed existing gene embedding methods published in recent years, especially those with novel algorithmic approaches to network analysis. In total, we selected 13 embedding methods (ARGA, ARGVA, SIG-VAE, DGVAE, scVI, LDVAE, siVAE, scVAE, scGNN, scapGNN, scETM, SAUCIE, and SIMBA) and

2 gene clustering methods (SCENC and LIGER) for comparison with scGraph2Vec.
All methods generated gene clusters. We employed three measurements to assess the gene clusters, i.e., the silhouette coefficient, the Davies-Bouldin Index, and the overrepresentation status of each cluster with the hallmark gene sets. The silhouette coefficient and the Davies-Bouldin Index measure how the clusters generated by each method can be clearly distinguished. As shown in Fig.2B-C in the revised manuscript, the clusters by scGraph2Vec outperformed nearly all other methods in the brain and PBMC tissues. The overrepresentation status of the hallmark genes measures the functional convergence of each cluster. As shown in Fig. 2E in the revised manuscript, scGraph2Vec generated gene clusters that shared the most with the hallmark genes, indicating that the clusters by scGraph2Vec were functionally convergent (Fig. 2E).

#2. The authors have used several datasets to validate the performance of scGraph2Vec. To further demonstrate the model's generalizability and robustness, testing on a broader range of datasets is recommended, especially those from different biological contexts or species.

Response: We thank the reviewer for this valuable suggestion. During the revision process, we included an additional application case by using melanoma cell lines with different states. In total, we have four applications representing three unique contexts (See Table 2). We hope these applications are satisfactory to answer your question.

#3. While the gene embeddings generated by scGraph2Vec are said to be biologically interpretable, the authors should further explore how these embeddings correspond to known biological processes and disease mechanisms. For the identified disease-associated genes, a more profound functional annotation and pathway analysis are suggested to uncover their specific roles in disease progression.

Response: We thank the reviewer for this valuable suggestion. To explore the biological relevance of gene embeddings, we conducted the following analyses.
(1) Network topology. The internal closeness centrality of genes in each cluster was significantly higher than their external closeness centrality (Fig. 3A).
(2) The clustering of annotated genes. Genes with similar functions, e.g., the housekeeping genes and the hallmark genes, tend to cluster together (Fig. 3B, C and Supplementary Fig. 13).
(3) Cell type specificity. When we applied scGraph2Vec to data from six representative human tissues, gene embeddings helped us identify clusters with cell type-specific expression (Fig. 4A, B and Supplementary Fig. S15). For instance, cluster 59 was enriched in multiple excitatory and inhibitory neurons and was particularly associated with down-regulated differentially expressed genes (DEGs) in these cell types (Fig. 4A and Supplementary Fig. S15).
(4) Tissue specificity. Embeddings also captured tissue-specific functional clusters. For example, our comparison of SMARCE1's neighboring genes in the brain and lung revealed distinct functions: in brain tissue, 14 neighboring genes were enriched in neuronal fate commitment and motor neuron differentiation (Fig. 4F), while in lung tissue, 20 neighboring genes were enriched in chromatin organization and histone H3-K9-related functions (Fig. 4G).
(5) Four applications to identify critical genes in different biological contexts. The four application cases, i.e., COVID-19, Alzheimer’s disease, lung adenocarcinoma, and melanoma, illustrated that scGraph2Vec can be widely used in various scenarios and identify candidate genes critical to the biological conditions, be it disease-associated genes, cancer driver genes, or regulatory genes for cell states.
In summary, scGraph2Vec integrates various gene networks and single-cell omics data from diverse biological backgrounds to generate highly informative gene embeddings. These embeddings capture both the topological characteristics of biological networks and biologically significant gene clusters, uncovering context-specific gene regulatory programs.

For the identified disease-related genes, we conducted in-depth functional annotation and pathway analysis. Based on pathway databases such as KEGG, WikiPathways, and Reactome, we applied the R packages clusterProfiler (v.4.6.2) and ReactomePA (v.1.42.0) to perform pathway enrichment analysis. Pathway annotation was done with ClueGO (v.2.5.10). Protein-protein interaction analysis was carried out using STRING. In COVID-19, pathway enrichment analysis revealed significant enrichment in Glycosaminoglycan degradation (PBH = 3.08 × 10-4), type I interferon induction and signaling during SARS-CoV-2 infection (PBH = 6.46 × 10-4), Interleukin-10 signaling (PBH = 3.03 × 10-3), and ABO blood

group biosynthesis (PBH = 3.29 × 10-3) (Fig. 5F). STRING analysis further demonstrated protein-protein interactions among genes involved in these pathways (Fig. 5G). In AD, our pathway annotation of 362 neighboring genes further revealed regulatory pathways involved in AD, mainly complex I biogenesis (PBH = 9.00 × 10-10), cholesterol transport (PBH = 1.07 × 10-4), regulation of cholesterol transport (PBH = 9.48 × 10-4), regulation of amyloid-beta clearance (PBH = 9.83 × 10-5) and oxidative phosphorylation (PBH = 3.67 × 10-9) (Fig. 6F, G).

#4. The manuscript mentions the optimization of model parameters through hyperparameter sweeping. The authors should provide more details on this process, including the specific ranges and tuning strategies used.

Response: We apologize for not providing the details of the hyperparameter sweeping process. In the revised manuscript, we added a detailed description of the hyperparameter selection and optimization process in the Methods section. Specifically, we considered standard VGAE parameters, i.e., learning rate, training iterations, encoding layers, and their dimensions, and new parameters that were specific to scGraph2Vec, i.e., λ, s, β, and γ. We tested λ (0.001, 0.1, 1), β (0.5, 5, 10), γ (0.1, 1), and s (2, 10). The optimal hyperparameters were determined by combining evaluation metrics, prioritizing a higher silhouette coefficient, a greater number of clusters, and a shorter running time. As a result, the optimal settings were λ=1,β=10,γ=0.1,s=10. Subsequently, we tested a two-layer encoder with dimensions 64-16, 128-16, 256-16, 128-32, and 256-32, as well as a three-layer encoder with dimensions 256-64-16 and 512-64-16. Finally, we recommended using a three-layer neural network (256-64-16) encoder. The Adam optimizer strategy and a learning rate = 1×10-4 were adopted. A maximum of 600 iterations and early stopping with patience = 50 was used. The model training was repeated 10 times in each tissue.

#5. For large-scale datasets, how efficient is scGraph2Vec in terms of computational resources? The authors are encouraged to assess and report the model's runtime and resource consumption, especially when dealing with large single-cell datasets.

Response: We apologize for not providing details about running processes. In the revised manuscript, we have added information about the computational resources. On page 20, in the last paragraph, we added:

"We used 64 logical CPUs and 512 GB RAM for all experiments. The execution time of scGraph2Vec depends on the size of the single-cell dataset and the interaction network. It ranges from 6.5 hours on the smallest dataset (PBMC: 2,638 cells and 13,714 genes) to 37.3 hours on the largest dataset (heart: 287,269 cells and 33,694 genes). For the same brain dataset, the TRRUST network (the smallest in size: 2,862 genes and 8,427 interactions) takes 0.28 hours, while the BioGRID network (the largest in size: 19,752 genes and 977,356 interactions) takes 7.6 hours."

#6. The performance of scGraph2Vec on different types of gene networks, such as metabolic or signaling networks, should be evaluated to test its universality. Discuss whether the model can be easily adapted to handle different types of omics data, such as proteomics or metabolomics.

Response: We thank the reviewer for this valuable suggestion. During the revision process, we applied scGraph2Vec to three widely used gene networks: the BioGRID network representing physical interactions, the Reactome network representing biological pathways, and the TRRUST network representing transcriptional regulatory relationships. We tested these on single-cell datasets from the brain and PBMC. The results demonstrated that scGraph2Vec can distinguish gene clusters regardless of the biological network type (Supplementary Fig. S5). Additionally, we assessed how different network features influenced gene clustering in the two-dimensional embedding space. By comparing gene clusters composed of hub genes with varying centrality levels and their neighboring genes, we found that scGraph2Vec effectively captures small, locally connected clusters, making it suitable for a range of biological networks (Supplementary Fig. S6-S7). In summary, scGraph2Vec can be broadly applied across different network types, including various omics data that provide clear gene-gene interaction relationships, such as proteomes and metabolomes.

#7. The visualizations provided are crucial for understanding the model's performance. The authors are advised to include additional visualizations to help readers more intuitively grasp the biological

characteristics and differences of gene modules.

Response: We appreciate the reviewer’s valuable suggestion regarding the visualizations. To address this, we included additional visualizations in the revised manuscript that highlight the biological characteristics and differences of the identified gene clusters. Specifically, we added Figure 2, and Supplementary Figures 9 and 10 to compare the cluster partitioning capabilities, gene cluster similarities, and overlap with known functional gene sets between scGraph2Vec and other competing algorithms. Supplementary Figures 6 and 7 were also included to assess how different characteristics of biological networks impact cluster partitioning. Additionally, we performed further functional annotation and pathway analysis for the identified disease-related genes (Fig. 5F, G and 6F, G). Furthermore, we introduced a case study applying scGraph2Vec to melanoma cell lines in different cell states, demonstrating its potential in uncovering cell state-related regulatory genes (Fig. 8).

#8. The discussion section should delve deeper into the advantages and limitations of scGraph2Vec compared to existing methods and its potential impact in practical applications.

Response: We appreciate the reviewer’s insightful suggestion. In the revised manuscript, we expanded the discussion section to provide a more in-depth comparison of the advantages and limitations of scGraph2Vec relative to existing methods and its potential impact in practical applications.

References

1. Langfelder P and Horvath S. WGCNA: an R package for weighted correlation network analysis. BMC Bioinformatics. 2008;9:559. doi:10.1186/1471-2105-9-559.
2. Tirosh I, Izar B, Prakadan SM, Wadsworth MH, 2nd, Treacy D, Trombetta JJ, et al. Dissecting the multicellular ecosystem of metastatic melanoma by single-cell RNA-seq. Science (New York, NY). 2016;352 6282:189-96. doi:10.1126/science.aad0501.
