# Supplementary material for: scGraph2Vec: a deep generative model for gene embedding augmented by graph neural network and single-cell omics data
Source: Gigascience. 2024 Dec 20;13:giae108. doi: 10.1093/gigascience/giae108 (PMC11659981; doi:10.1093/gigascience/giae108)
Supplement: giae108_Response_to_Reviewer_Comments_Revision_1 [file giae108_response_to_reviewer_comments_revision_1.pdf]

Author's Response To Reviewer Comments

Response to the comments of the editor and reviewers
Manuscript ID: GIGA-D-24-00200R2
Title: scGraph2Vec: a deep generative model for gene embedding augmented by Graph Neural Network and single-cell omics data

We appreciate very much the positive feedback and valuable comments from the editor and reviewers. Each of these comments has been considered in the revised manuscript or discussed below. Briefly, we have made the following revisions:
1. We carefully reviewed the entire bibliography and corrected any formatting issues to ensure that all citations are consistent and adhere to the required style.
2. We rephrased the sentences to improve clarity and meet scientific writing standards.
The following is an outline of our point-to-point responses to editor and reviewer's criticisms.

Response to the editor:
Comment: Your manuscript "scGraph2Vec: a deep generative model for gene embedding augmented by Graph Neural Network and single-cell omics data" (GIGA-D-24-00200R1) has been assessed by our reviewers. Based on these reports, and my own assessment as Editor, I am pleased to inform you that it is potentially acceptable for publication in GigaScience, once you have carried out some essential revisions suggested by our reviewers.
Their reports, together with any other comments, are below. Please also take a moment to check our website at https://www.editorialmanager.com/giga/ for any additional comments that were saved as attachments.
In addition, please register any new software application in the bio.tools and SciCrunch.org databases to receive RRID (Research Resource Identification Initiative ID) and biotoolsID identifiers, and include these in your manuscript. Computational workflows should be registered in workflowhub.eu and the DOIs cited in the relevant places in the manuscript. These will facilitate tracking, reproducibility and re-use of your tool.

Response: We thank the editor for giving us the opportunity to revise our manuscript. We have registered scGraph2Vec in both bio.tools and SciCrunch.org databases, and include the RRID (SCR_025322) and biotoolsID (scGraph2Vec) identifiers in our manuscript.

Response to Referee #2:
#1. In the response to comment #2 (comparing scGraph2Vec with other VGAE-based gene embedding methods), the authors have referred two methods as the 'first reference' and 'second reference'. Later they have explained that the method 'GenKI' is referred to as the 'first reference'. I request the authors to provide a citation for the GenKI in their response. Secondly, the authors did not provide any name or citation for the 'second reference'. I request the authors to provide these information as well. Finally, in the response, the authors have discussed why these methods were excluded from the comparison -- one due to its high time complexity and another due to the absence of its source code. I would request the authors to add this discussion to the main text as well. It will help the readers to decide whether they should invest time in running or implementing these methods.

Response: We thank the reviewer for bringing this point up. Here, we have added the citations of the 'first reference' GenKI [1] and the 'second reference'[2] in this response. In addition, we have also incorporated a discussion of these methods into the main text. On page 12, in the last paragraph, we added:
"Notably, to the best of our knowledge, there is currently no method specifically designed for integrating gene interaction networks with single-cell omics data to generate gene embeddings. Therefore, we selected representative VGAE-based methods capable of generating gene embeddings based on single-cell data, excluding those unable to generate embeddings for all genes within a reasonable time frame [1] or lacking available source code [2]."

#2. In the bibliography, some of the citations are not properly formatted. This is especially true for the citations newly added in the revised version. For example, "Pan S, Hu R, Long G, Jiang J, Yao L and Zhang

CJapa. Adversarially regularized graph autoencoder for graph embedding. 2018."

Response: We apologize for the oversight. We have carefully reviewed and corrected the formatting of the entire bibliography to ensure it adheres to the required style.

#3. Some sentences need to be rephrased to meet the standards of scientific/technical writing. For example, the sentence "We were surprised to find very low similarity between clusters identified by different methods (Fig. 2D)" can be rephrased as "We found that the clusters identified by different methods have low similarities (Fig. 2D). This is counter-intuitive since [please add the reason you were surprised]".

Response: We appreciate the reviewer’s valuable guidance in helping to strengthen the clarity of our writing. We have carefully rephrased some sentences to improve clarity and meet scientific/ technical writing standards. The revised version of the example sentence now reads:
“We found that the clusters identified by different methods have low similarities (Fig. 2D). This is counter-intuitive since clusters of potentially interacting genes would be expected to be consistently identified by at least some of these methods.”

References
1. Yang Y, Li G, Zhong Y, Xu Q, Chen BJ, Lin YT, et al. Gene knockout inference with variational graph autoencoder learning single-cell gene regulatory networks. Nucleic acids research. 2023;51 13:6578-92. doi:10.1093/nar/gkad450.
2. Ganeshamoorthy S, Roden L, Klepl D and He F. Gene Regulatory Network Inference through Link Prediction using Graph Neural Network. In: 2022 IEEE Signal Processing in Medicine and Biology Symposium (SPMB) 2022, pp.1-5. IEEE.
