# Supplementary material for: scGraph2Vec: a deep generative model for gene embedding augmented by graph neural network and single-cell omics data
Source: Gigascience. 2024 Dec 20;13:giae108. doi: 10.1093/gigascience/giae108 (PMC11659981; doi:10.1093/gigascience/giae108)
Supplement: giae108_Reviewer_1_Report_Original_Submission — Bin Li -- 7/12/2024 Reviewed [file giae108_reviewer_1_report_original_submission.pdf]

Reviewer Report

Title: scGraph2Vec: a deep generative model for gene embedding augmented by Graph Neural Network and single-cell omics data

Version: Original Submission **Date:** 7/12/2024

Reviewer name: Bin Li

Reviewer Comments to Author:

The main contribution of this study lies in the proposal of a new model, scGraph2Vec, which leverages the powerful representational capabilities of Graph Neural Networks (GNNs) to capture the intricate interaction between genes, while incorporating omics information at the single-cell level. This approach facilitates a deeper understanding for researchers of gene regulation, cell type differentiation, and the molecular mechanisms underlying diseases. However, the content of this manuscript requires further enhancement, particularly in elucidating the novel biological insights or advantages offered by the gene embeddings generated by this model compared to those gene modules derived from other state-of-the-art algorithms. In addition to the shortcomings in novelty and biological significance within this manuscript, here are some revision suggestions:1. In Fig. 2, the authors show that the gene embeddings acquired through the scGraph2Vec model achieve higher silhouette scores than those resulting from alternative algorithms. Nonetheless, they fail to expound upon the biological relevance of these gene embeddings, nor do they illustrate that the gene embeddings procured via scGraph2Vec encompass greater or more precise biological insights relative to the gene modules yielded by competing algorithms.2. In Fig. 2B, the authors indicated that "housekeeping genes tended to cluster together and were enriched in a few gene modules". Does this suggest that these gene modules are related to cell maintenance functions or states?3. The authors used module score to represent the overall expression pattern of module genes, is there mathematical proof for this? Is there biological evidence to support this claim? Is there a ground truth available to validate its reliability?4. In Fig. 4, the authors performed a GO-term analysis of the gene modules obtained from the scGraph2Vec model, and utilized the functions of these modules to investigate the functionalities of various cell types. What are the advantages of this approach compared to directly using differentially expressed genes (DEGs), or gene modules obtained from other algorithms?5. In studies on COVID-19 and Alzheimer's disease, the authors utilized disease-related variants identified through GWAS analysis, combined with information from gene modules, to screen for genes associated with these diseases. How did the authors determine that these variants in the genes would lead to changes in the RNA expression levels of these genes? Was it because these variant sites were located in promoter or enhancer regions of these genes? Is there any experimental evidence for this?6. Some paragraphs and sentences in this manuscript have rather stiff language expression. The authors can use English proofreading service to prevent grammatical and semantic errors in expression.

Level of Interest

Please indicate how interesting you found the manuscript: Choose an item.

Quality of Written English

Please indicate the quality of language in the manuscript: Choose an item.

Declaration of Competing Interests

Please complete a declaration of competing interests, considering the following questions:

- Have you in the past five years received reimbursements, fees, funding, or salary from an organisation that may in any way gain or lose financially from the publication of this manuscript, either now or in the future?
- Do you hold any stocks or shares in an organisation that may in any way gain or lose financially from the publication of this manuscript, either now or in the future?
- Do you hold or are you currently applying for any patents relating to the content of the manuscript?
- Have you received reimbursements, fees, funding, or salary from an organization that holds or has applied for patents relating to the content of the manuscript?
- Do you have any other financial competing interests?
- Do you have any non-financial competing interests in relation to this paper?

If you can answer no to all of the above, write 'I declare that I have no competing interests' below. If your reply is yes to any, please give details below.

I declare that I have no competing interests

I agree to the open peer review policy of the journal. I understand that my name will be included on my report to the authors and, if the manuscript is accepted for publication, my named report including any attachments I upload will be posted on the website along with the authors' responses. I agree for my report to be made available under an Open Access Creative Commons CC-BY license (http://creativecommons.org/licenses/by/4.0/). I understand that any comments which I do not wish to be included in my named report can be included as confidential comments to the editors, which will not be published.

Choose an item.

To further support our reviewers, we have joined with Publons, where you can gain additional credit to further highlight your hard work (see: https://publons.com/journal/530/gigascience). On publication of this paper, your review will be automatically added to Publons, you can then choose whether or not to claim your Publons credit. I understand this statement.

Yes Choose an item.
