# Supplementary material for: scGraph2Vec: a deep generative model for gene embedding augmented by graph neural network and single-cell omics data
Source: Gigascience. 2024 Dec 20;13:giae108. doi: 10.1093/gigascience/giae108 (PMC11659981; doi:10.1093/gigascience/giae108)
Supplement: giae108_Reviewer_2_Report_Original_Submission — Saptarshi Pyne -- 7/15/2024 Reviewed [file giae108_reviewer_2_report_original_submission.pdf]

Reviewer Report

Title: scGraph2Vec: a deep generative model for gene embedding augmented by Graph Neural Network and single-cell omics data

Version: Original Submission **Date:** 7/15/2024

Reviewer name: Saptarshi Pyne

Reviewer Comments to Author:

The authors have proposed a computational method called 'scGraph2Vec' that takes a single-cell gene expression profile and a gene interaction network as input, and produces low-dimensional gene embedding as output. The links to the used datasets are either provided in the Supplementary Table S1 or mentioned in the main text. The source code has been made publicly available at https://github.com/LPH-BIG/scGraph2Vec . I highly appreciate the effort the authors have made to improve the reproducibility of their work. It would be great if the authors could address the following comments to strengthen the publication.1. The background section needs to be significantly improved. It has been written that scGraph2Vec "extends the VGAE framework". However, VGAE is a data-agnostic framework; scGraph2Vec rather utilises the VGAE framework for analysing gene expression and gene interaction network data. Therefore, the authors need to incorporate a critical review of the recent method that do the same. I am listing a few references I could find for that purpose:https://academic.oup.com/nar/article/51/13/6578/7184155https://isip.piconepress.com/conferences/ieee_spmb/2022/papers/p01_07.pdfhttp://dr.iiserpune.ac.in:8080/xmlui/handle/123456789/7885https://arxiv.org/abs/1802.04407There are more references that I could not list here. I request the authors to enhance the background section by reviewing such methods.2. In addition to the critically reviewing other VGAE-based gene embedding methods, I request the authors to include them in the comparisons based on the benchmark datasets.3. The authors have utilised the gene embedding outputs of scGraph2Vec for clustering genes. Therefore, I request the authors to compare the resultant gene clusters with that of other single-cell gene expression based gene clustering methods such as SCENIC (https://www.nature.com/articles/nmeth.4463) and LIGER (https://www.cell.com/cell/pdf/S0092-8674(19)30504-5.pdf).4. The authors have used the silhouette index to compare the gene clusters produced by different methods. I would request the authors to incorporate additional evaluation metrics such as the Davies-Bouldin index (DBI) and Jaccard index.

Level of Interest

Please indicate how interesting you found the manuscript: Choose an item.

Quality of Written English

Please indicate the quality of language in the manuscript: Choose an item.

Declaration of Competing Interests

Please complete a declaration of competing interests, considering the following questions:

- Have you in the past five years received reimbursements, fees, funding, or salary from an organisation that may in any way gain or lose financially from the publication of this manuscript, either now or in the future?
- Do you hold any stocks or shares in an organisation that may in any way gain or lose financially from the publication of this manuscript, either now or in the future?
- Do you hold or are you currently applying for any patents relating to the content of the manuscript?
- Have you received reimbursements, fees, funding, or salary from an organization that holds or has applied for patents relating to the content of the manuscript?
- Do you have any other financial competing interests?
- Do you have any non-financial competing interests in relation to this paper?

If you can answer no to all of the above, write 'I declare that I have no competing interests' below. If your reply is yes to any, please give details below.

I declare that I have no competing interests

I agree to the open peer review policy of the journal. I understand that my name will be included on my report to the authors and, if the manuscript is accepted for publication, my named report including any attachments I upload will be posted on the website along with the authors' responses. I agree for my report to be made available under an Open Access Creative Commons CC-BY license (http://creativecommons.org/licenses/by/4.0/). I understand that any comments which I do not wish to be included in my named report can be included as confidential comments to the editors, which will not be published.

Choose an item.

To further support our reviewers, we have joined with Publons, where you can gain additional credit to further highlight your hard work (see: https://publons.com/journal/530/gigascience). On publication of this paper, your review will be automatically added to Publons, you can then choose whether or not to claim your Publons credit. I understand this statement.

Yes Choose an item.
