# Supplementary material for: scGraph2Vec: a deep generative model for gene embedding augmented by graph neural network and single-cell omics data
Source: Gigascience. 2024 Dec 20;13:giae108. doi: 10.1093/gigascience/giae108 (PMC11659981; doi:10.1093/gigascience/giae108)
Supplement: giae108_Reviewer_2_Report_Revision_1 — Saptarshi Pyne -- 11/2/2024 Reviewed [file giae108_reviewer_2_report_revision_1.pdf]

Reviewer Report

Title: scGraph2Vec: a deep generative model for gene embedding augmented by Graph Neural Network and single-cell omics data

Version: Revision 1 **Date:** 11/2/2024

Reviewer name: Saptarshi Pyne

Reviewer Comments to Author:

1. In the response to comment #2 (comparing scGraph2Vec with other VGAE-based gene embedding methods), the authors have referred two methods as the 'first reference' and 'second reference'. Later they have explained that the method 'GenKI' is referred to as the 'first reference'. I request the authors to provide a citation for the GenKI in their response. Secondly, the authors did not provide any name or citation for the 'second reference'. I request the authors to provide these information as well. Finally, in the response, the authors have discussed why these methods were excluded from the comparison -- one due to its high time complexity and another due to the absence of its source code. I would request the authors to add this discussion to the main text as well. It will help the readers to decide whether they should invest time in running or implementing these methods.2. In the bibliography, some of the citations are not properly formatted. This is especially true for the citations newly added in the revised version. For example, "Pan S, Hu R, Long G, Jiang J, Yao L and Zhang CJapa. Adversarially regularized graph autoencoder for graph embedding. 2018."3. Some sentences need to be rephrased to meet the standards of scientific/technical writing. For example, the sentence "We were surprised to find very low similarity between clusters identified by different methods (Fig. 2D)" can be rephrased as "We found that the clusters identified by different methods have low similarities (Fig. 2D). This is counter-intuitive since [please add the reason you were surprised]".

Level of Interest

Please indicate how interesting you found the manuscript: Choose an item.

Quality of Written English

Please indicate the quality of language in the manuscript: Choose an item.

Declaration of Competing Interests

Please complete a declaration of competing interests, considering the following questions:

- Have you in the past five years received reimbursements, fees, funding, or salary from an organisation that may in any way gain or lose financially from the publication of this manuscript, either now or in the future?

- Do you hold any stocks or shares in an organisation that may in any way gain or lose financially from the publication of this manuscript, either now or in the future?
- Do you hold or are you currently applying for any patents relating to the content of the manuscript?
- Have you received reimbursements, fees, funding, or salary from an organization that holds or has applied for patents relating to the content of the manuscript?
- Do you have any other financial competing interests?
- Do you have any non-financial competing interests in relation to this paper?

If you can answer no to all of the above, write 'I declare that I have no competing interests' below. If your reply is yes to any, please give details below.

I declare that I have no competing interests

I agree to the open peer review policy of the journal. I understand that my name will be included on my report to the authors and, if the manuscript is accepted for publication, my named report including any attachments I upload will be posted on the website along with the authors' responses. I agree for my report to be made available under an Open Access Creative Commons CC-BY license (http://creativecommons.org/licenses/by/4.0/). I understand that any comments which I do not wish to be included in my named report can be included as confidential comments to the editors, which will not be published.

Choose an item.

To further support our reviewers, we have joined with Publons, where you can gain additional credit to further highlight your hard work (see: https://publons.com/journal/530/gigascience). On publication of this paper, your review will be automatically added to Publons, you can then choose whether or not to claim your Publons credit. I understand this statement.

Yes Choose an item.
