# Supplementary material for: scGraph2Vec: a deep generative model for gene embedding augmented by graph neural network and single-cell omics data
Source: Gigascience. 2024 Dec 20;13:giae108. doi: 10.1093/gigascience/giae108 (PMC11659981; doi:10.1093/gigascience/giae108)
Supplement: giae108_Reviewer_3_Report_Original_Submission — Yuansong Zeng -- 7/16/2024 Reviewed [file giae108_reviewer_3_report_original_submission.pdf]

**Reviewer Report**

**Title: scGraph2Vec: a deep generative model for gene embedding augmented by Graph Neural Network and single-cell omics data**

**Version: Original Submission** **Date:** 7/16/2024

**Reviewer name: Yuansong Zeng**

**Reviewer Comments to Author:**

The manuscript introduces a novel deep-learning framework designed to generate informative gene embeddings. This framework, scGraph2Vec, extends the variational graph autoencoder (VGAE) model and integrates single-cell data patterns along with gene interaction networks. The model's effectiveness is demonstrated through its ability to produce biologically interpretable gene embeddings that reveal functional modules of genes, which can be applied to identify disease-associated genes and understand tissue-specific cellular processes. However, the authors still have the following problems to solve:1. The manuscript compares scGraph2Vec with several existing gene embedding methods. It would be beneficial to include more recent methods, especially those with novel algorithmic approaches to network analysis.2. The authors have used several datasets to validate the performance of scGraph2Vec. To further demonstrate the model's generalizability and robustness, testing on a broader range of datasets is recommended, especially those from different biological contexts or species.3. While the gene embeddings generated by scGraph2Vec are said to be biologically interpretable, the authors should further explore how these embeddings correspond to known biological processes and disease mechanisms. For the identified disease-associated genes, a more profound functional annotation and pathway analysis are suggested to uncover their specific roles in disease progression.4. The manuscript mentions the optimization of model parameters through hyperparameter sweeping. The authors should provide more details on this process, including the specific ranges and tuning strategies used.5. For large-scale datasets, how efficient is scGraph2Vec in terms of computational resources? The authors are encouraged to assess and report the model's runtime and resource consumption, especially when dealing with large single-cell datasets.6. The performance of scGraph2Vec on different types of gene networks, such as metabolic or signaling networks, should be evaluated to test its universality. Discuss whether the model can be easily adapted to handle different types of omics data, such as proteomics or metabolomics.7. The visualizations provided are crucial for understanding the model's performance. The authors are advised to include additional visualizations to help readers more intuitively grasp the biological characteristics and differences of gene modules.8. The discussion section should delve deeper into the advantages and limitations of scGraph2Vec compared to existing methods and its potential impact in practical applications.

**Level of Interest**

Please indicate how interesting you found the manuscript: Choose an item.

**Quality of Written English**

Please indicate the quality of language in the manuscript: Choose an item.

**Declaration of Competing Interests**

Please complete a declaration of competing interests, considering the following questions:

- Have you in the past five years received reimbursements, fees, funding, or salary from an organisation that may in any way gain or lose financially from the publication of this manuscript, either now or in the future?
- Do you hold any stocks or shares in an organisation that may in any way gain or lose financially from the publication of this manuscript, either now or in the future?
- Do you hold or are you currently applying for any patents relating to the content of the manuscript?
- Have you received reimbursements, fees, funding, or salary from an organization that holds or has applied for patents relating to the content of the manuscript?
- Do you have any other financial competing interests?
- Do you have any non-financial competing interests in relation to this paper?

If you can answer no to all of the above, write 'I declare that I have no competing interests' below. If your reply is yes to any, please give details below.

No competing interests

I agree to the open peer review policy of the journal. I understand that my name will be included on my report to the authors and, if the manuscript is accepted for publication, my named report including any attachments I upload will be posted on the website along with the authors' responses. I agree for my report to be made available under an Open Access Creative Commons CC-BY license (http://creativecommons.org/licenses/by/4.0/). I understand that any comments which I do not wish to be included in my named report can be included as confidential comments to the editors, which will not be published.

Choose an item.

To further support our reviewers, we have joined with Publons, where you can gain additional credit to further highlight your hard work (see: https://publons.com/journal/530/gigascience). On publication of this paper, your review will be automatically added to Publons, you can then choose whether or not to claim your Publons credit. I understand this statement.

Yes Choose an item.
